# Supplementary material for: ‘Our culture prohibits some things’: qualitative inquiry into how sociocultural context influences the scale-up of community-based injectable contraceptives in Nigeria
Source: BMJ Open. 2020 Jul 19;10(7):e035311. doi: 10.1136/bmjopen-2019-035311 (PMC7371132; doi:10.1136/bmjopen-2019-035311)
Supplement: Supplementary data [file bmjopen-2019-035311supp001.pdf]

SUPPLEMENTARY FILE 1

Interview Guide: Key informant interview with senior MOH officials and NGO programme managers

1. Let's start by you describing your role in implementing this intervention – the community-based delivery of injectable contraceptives in the pilot sites in Gombe. What was your role and how long were you involved?

2. Did you encounter any challenges during the pilot study? Please explain.
   Probes:
   - If not mentioned, ask: any resistance from the community members, from health workers, from other stakeholders, from potential users themselves?
   - How did you handle challenges / resistance?

3. We are interested in your experience with the process of taking the intervention from pilot study phase to wider implementation in Gombe itself. What was the process, from implementation to wider scale up of the intervention? Walk me through the process.
   Probes:
   - The goal, approach to implementing, key components of the process, indicators of success
   - Landscape and stakeholder assessment done – before, during or after the pilot?
   - Your role in these processes?

4. In your opinion, what factors have supported the scale up of this intervention so far?

5. Have you experienced challenges during the process of scaling up? Please explain.
   Probes:
   - What challenges? ... ***may prompt with*** "any others"
   - How did you handle challenges?

6. Was there a way of ensuring that the intervention aligns to the wants and preferences of the users you were targeting? Please tell me about it.
   Probes:
   - Any user needs / wants assessment done? By whom? When?
   - Any modifications made in order to align packaging of the innovation to user wants – either during or after the pilot?
   - Any health service modification (e.g. change service models for delivering the intervention)?

7. Have communities been engaged in the scale up process? Please describe in what ways and by whom?

8. Looking back, is there anything that you think could have been done differently to facilitate the process from pilot study to wider implementation? Please explain.

9. Is there anything else we have not asked but you would like to tell us about your experience with the process of implementation and scale-up of CBD?

Thank you for your time. This is the end of our discussion.

In-depth Interview Guides

A. Interview guide: In-depth interview with health workers (Doctors and CHEWs)

1. Let's start by you describing your role in implementing this intervention – the community-based delivery of injectable contraceptives by community health extension workers.
    a. What role did doctors / CHEWs play in designing the intervention?
    b. What about their role in introducing the intervention at community level?
    c. What was your own role?
    Probes:
    - **Doctors:** Your own (or experience of other doctors like you) with: planning, training CHEWs, providing supportive supervision to CHEWs during and after the pilot?
    - **CHEWs**: Your own (or experience of other CHEWs like you) in planning, administering the injectable contraceptives to women?

    d. Did you / other health workers experience challenges?
        i. Please explain.
        ii. How did you handle the challenges?

2. In your opinion, what factors affect wider implementation and uptake of the CBD of injectable contraceptives in the communities that you serve?
    Probes:
    - Anything else?

3. In your opinion, how supportive of this innovation are:
    a. **For doctors**: doctors in general, and your professional health group (e.g. Nigerian Medical Association) please explain.
    b. **For CHEWs**: community health extension workers in general? Please explain.

4. As far as you are aware, what role, if any, have health workers like you played in making the CBD of injectable contraceptives more acceptable to the users in your community? Please explain.

5. In your opinion, how likely are those women who have already adopted the CBD of injectables to spread information about the innovation to other women who are not using it? Please explain.

6. Looking back, is there anything that you feel might have been done differently to facilitate the process of moving from pilot study to wider implementation?

7. Is there anything else we have not discussed that you would like to tell us about your experience with implementing the CBD of injectable contraceptives?

Thank you for your time. This is the end of our discussion.

B. Interview guide: In-depth interview with community leaders

1. You were selected to participate in this study because you are aware of the government program in which CHEWs provide injectable contraceptives to women in the community, outside the health clinic setting.
   a. Please describe what you know about this program.
   b. How did you hear about it?

2. How much support do you think there is for this program in your community? Please explain

3. Have you or other community leaders like you been involved in introducing this program on injectable contraceptives into your community?
   a. ***If yes***: Describe your / or other leaders' involvement (what role)? How did you / they get involved? Who initiated?
   b. How do you feel about being involved / not being involved?

4. The government started with this program only in two places in Gombe but they are now trying to make this available to all other parts of Gombe.
   a. What challenges do you think the government might encounter when trying to get wider uptake of this programme in the community?
   b. Have you or other community leaders in this community been approached by government to assist with addressing these challenges?

5. In your opinion, what might prevent women in your community from taking up this intervention? What may encourage them to take it up?

6. In your opinion, how likely are those women who have already adopted the CBD of injectables to share information about it to other women who are not using it? Please explain?

7. Looking back, is there anything that you feel might have been done differently to make this way of providing contraceptives acceptable in your community?

8. Is there anything else we have not discussed that you would like to tell us about your experience with implementing the CBD of injectable contraceptives?

Thank you for your time. This is the end of our discussion.

Focus Group Discussion Guides

A. Focus group discussion guide: FGDs with women who have already adopted the use of injectable contraceptives through the CBD approach

1. Let us start with talking about contraceptives / family planning methods in general. Please share with me what you understand about contraception / family planning. What kinds of contraceptive methods do you know?

2. What do you think people in this community think about contraceptives and family planning in general?

   Probe:
   - Do they support the idea of contraception / family planning in general?
   - Specifically, how supportive of injectable contraceptives compared to others?

3. What about women in this community. How do you think they feel about contraceptives and family planning in general?

   Probe:
   - Support the use of contraception / family planning in general?
   - Specifically, how popular are injectable contraceptives compared to others?
   - What makes women use injectable contraceptives? What makes them not use it?

4. I am interested to know what you think women want and prefer regarding contraceptives. Which types of contraceptives do women in your community prefer? How do you think women prefer to receive contraceptives (from clinic versus other places; from doctor or nurse versus others)?

5. The government introduced a new program where community health extension workers now administer injectable contraceptives to women away from the clinic, including at homes. I know that you already receive injectable contraceptives through this approach.
   a. How did you hear about this program?
   b. What made women like you to decide to receive injectables through CHEWs?
   c. Would you encourage friends or family members to receive injectable contraceptives from CHEWs? Please explain.
   d. What do you think might prevent other women in your community from using this new way of receiving injectable contraceptives through CHEWs?

6. What suggestions do you have for how the way injectable contraceptives are administered in your community can be improved to make sure many more women receive them?

Thank you for your time. This is the end of our discussion

B. Focus group discussion guide: FGD with a group of women in the reproductive age group who have not yet adopted the CBD of injectables (may include those who do not use injectables and those who do)

1. Let us start with talking about contraceptives / family planning methods in general. How many contraceptive types do you know? Can you describe them?

2. How do you think people in this community perceive contraceptives and family planning in general?

   Probe:
   - Popularity of contraceptives / the idea of family planning in general
   - Specifically, popularity of injectable contraceptives

3. How do you think women in this community perceive contraceptives and family planning in general?

   Probe:
   - Popularity of contraceptives / family planning in general
   - Specifically, popularity of injectable contraceptives

4. What do women want - which types of contraceptives, which mode of delivery do they prefer? (clinic vs. other places, doctor/nurse versus others)

5. Let us now talk about injectable contraceptives specifically. In your opinion, what factors will make women of reproductive age use or reject injectable contraceptives? What makes them not use it?

6. The government introduced a new program where community health extension workers now administer injectable contraceptives to women away from the clinic, including at homes. I know that you have not received injectable contraceptives through this approach.
   a. How did you hear about this program?
   b. What factors make women like you decide not to receive injectables through CHEWs?
   c. What factors would encourage you to receive the injectable contraceptives through CHEWs?
   d. Willingness to encourage friends and family members of reproductive age to receive injectable contraceptives from CHEWs?
   e. What do you think might prevent other women in your community from using this new way of receiving injectable contraceptives through CHEWs?

   Probes:
   - Resistance to mode of distribution – facility vs. community based
   - Socio-cultural resistance to injectable contraceptives
   - Resistance to injectable contraceptives being administered by CHEWs rather than other health workers like nurses and doctors

7. What suggestions do you have for how the way injectable contraceptives are administered in your community can be improved to make sure many more women like you receive them?

Thank you for your time. This is the end of our discussion.

C. Focus group discussion guide: FGD with elderly women (older than reproductive age) in the community

1. Let us start with talking about contraceptives / family planning methods in general. How many contraceptive types do you know? Can you describe them?

2. How do you think people in this community perceive contraceptives and family planning in general?
   Probe:
   - Popularity of contraceptives / the idea of family planning in general
   - Specifically, popularity of injectable contraceptives
3. What role do you play in advising your married daughters/daughters-in-law and family members of reproductive age about contraception/family planning?

4. How do you think elderly women (grandmothers) in this community perceive contraceptives and family planning in general?
   Probe:
   - Popularity of contraceptives / family planning in general among elderly women (awareness and knowledge, not use)
   - Specifically, popularity of injectable contraceptives

5. What do elderly women prefer? Which types of contraceptives, mode of delivery (clinic vs. other places, doctor/nurse versus others) do they prefer for their married daughters/daughters-in-law

6. Let us now talk about injectable contraceptives specifically. In your opinion, what factors will make women of reproductive age use or reject injectable contraceptives?
7. Will you support your married daughters/daughters-in-law using injectable contraceptives? Please give reasons for your answer.
8. The government introduced a new program where community health extension workers now administer injectable contraceptives to women away from the clinic, including at homes.

   a. How did you hear about this program? In your opinion, what factors help women to decide to receive injectables through CHEWs?
   b. Willingness to encourage married daughters/daughters-in-law and family members of reproductive age to receive injectable contraceptives from CHEWs?
   c. What do you think might prevent women in your community from using injectable contraceptives through CHEWs?
   d. What kinds of challenges do you know the government has faced with the distribution of injectable contraceptives through CHEWs in your community?

   Probes:
   - Resistance to mode of distribution – facility vs. community based
   - Socio-cultural resistance to injectable contraceptives
   - Resistance to injectable contraceptives being administered by CHEWs rather than other health workers like nurses and doctors

8. What suggestions do you have for how the way injectable contraceptives are administered in your community can be improved to make sure many more women receive them?

Thank you for your time. This is the end of our discussion.

D. Focus group discussion guide: FGD with younger and older men (single and married) in the community

1. Let us start with talking about contraceptives / family planning methods in general. How many contraceptive types do you know? Can you describe them?

2. How do you think people in this community perceive contraceptives and family planning in general?
   Probe:
   - Popularity of contraceptives / the idea of family planning in general
   - Specifically, popularity of injectable contraceptives

3. How do you think men in this community perceive contraceptives and family planning in general?
   Probe:
   - Popularity of contraceptives / family planning in general among men
   - Specifically, popularity of injectable contraceptives

4. What do men want - which types of contraceptives, mode of delivery (clinic vs. other places, doctor/nurse versus others) do they prefer for their wives
5. Let us now talk about injectable contraceptives specifically. In your opinion, what factors will make women of reproductive age use or reject injectable contraceptives?
6. Will you support your wife/partner/girlfriend using injectable contraceptives? Please give reasons for your answer.
7. The government introduced a new program where community health extension workers now administer injectable contraceptives to women away from the clinic, including at homes.

   a. How did you hear about this program? In your opinion, what factors help women to decide to receive injectables through CHEWs?
   b. Willingness to encourage wife/partner/girlfriend and family members of reproductive age to receive injectable contraceptives from CHEWs?
   c. What do you think might prevent women in your community from using injectable contraceptives through CHEWs?
   d. What kinds of challenges do you know the government has faced with the distribution of injectable contraceptives through CHEWs in your community?

   Probes:
   - Resistance to mode of distribution – facility vs. community based
   - Socio-cultural resistance to injectable contraceptives
   - Resistance to injectable contraceptives being administered by CHEWs rather than other health workers like nurses and doctors

9. What suggestions do you have for how the way injectable contraceptives are administered in your community can be improved to make sure many more women receive them?

Thank you for your time. This is the end of our discussion.
