# Supplementary material for: ‘Our culture prohibits some things’: qualitative inquiry into how sociocultural context influences the scale-up of community-based injectable contraceptives in Nigeria
Source: BMJ Open. 2020 Jul 19;10(7):e035311. doi: 10.1136/bmjopen-2019-035311 (PMC7371132; doi:10.1136/bmjopen-2019-035311)
Supplement: Supplementary data [file bmjopen-2019-035311supp002.pdf]

SUPPLEMENTARY FILE 2

Participant Information Sheet for MOH officials

Study title: Scale up of community-based injectable contraceptives in Gombe State, Nigeria

Introduction:

Good day! I am Oluwaseun Akinyemi, a PhD candidate of the School of Public Health, University of the Witwatersrand, Johannesburg. I am doing research on the scale up of community-based injectable contraceptives in Gombe State, Nigeria. Research is just the process to learn the answer to a question. In this study we want to learn what the factors are that may influence the scale up of community-based distribution of injectable contraceptives in Nigeria, in order to understand possible facilitators and barriers.

Invitation to participate: I am therefore asking / inviting you to take part in this research study because you are a senior MOH official who was/is involved with this programme.

What is involved in the study – I am asking different people such as policy makers, program managers, health workers, community leaders, women who are the end-users of injectable contraceptives, and broader members of the community to participate in this study. About 100 people will participate in the study and all will be from Nigeria. If you agree to participate it means you will take part in a key informant interview. Each interview will last for about 60 minutes. In the interviews I will ask questions about your knowledge and your role in the implementation or use of injectable contraceptives as well as what you think might be the challenges and things that work well in implementing this program. There are no right and wrong answers; I am interested in understanding your opinions. Please note that I intend to audio-tape this interview.

Risks and Benefits of being involved in the study – There are no invasive procedures involved in this study, thus no physical harm is envisaged. However, you don't have to answer any question you are not comfortable with.

There are no direct benefits for participating in this study.

The participant will be given pertinent information on the study while involved in the project and after the results are available.

Participation is voluntary: Refusal to participate will involve no penalty or loss of benefits to which you are otherwise entitled. Also, you may discontinue participation at any time without penalty or loss of benefits to which you are otherwise entitled.

Reimbursements for transportation: Transportation costs incurred in order to participate in the study will be reimbursed at the rate of ₦100 per km.

Confidentiality: Efforts will be made to keep personal information confidential. Absolute confidentiality cannot be guaranteed. Personal information may be disclosed if required by law.

Organizations that may inspect and/or copy the research records for quality assurance and data analysis include groups such as the Research Ethics Committee. If results are published, may lead to individual / cohort identification.

Contact details of researcher – for further information / reporting of study related adverse events.

Dr Oluwaseun Akinyemi
Department of Health Policy and Management
College of Medicine
University of Ibadan
Ibadan, Nigeria
+234 803 502 0136
seunakinyemi@hotmail.com

Contact details of UI/UCH Ethics Committee chair (Nigeria) – for more information.

Prof. Catherine O. Falade
Chairperson, University of Ibadan/University College Hospital Ethics Committee
Institute for Advanced Medical Research and Training (IAMRAT)
College of Medicine, University of Ibadan
Ibadan, Nigeria
+234 803 326 4593
cfalade@comui.edu.ng

Contact details of REC administrator and chair (South Africa) – for more information.

Ms Zanele Ndlovu
Research Administrator
Human Research Ethics Committee (Medical)
University of the Witwatersrand
Johannesburg, South Africa
+27 11 717 1252
zanele.ndlovu@wits.ac.za

Participant Information Sheet for NGO programme managers

Study title: Scale up of community-based injectable contraceptives in Gombe State, Nigeria

Introduction:

Good day! I am Oluwaseun Akinyemi, a PhD candidate of the School of Public Health, University of the Witwatersrand, Johannesburg. I am doing research on the scale up of community-based injectable contraceptives in Gombe State, Nigeria. Research is just the process to learn the answer to a question. In this study we want to learn what the factors are that may influence the scale up of community-based distribution of injectable contraceptives in Nigeria, in order to understand possible facilitators and barriers.

Invitation to participate: I am therefore asking / inviting you to take part in this research study because you are a programme manager for an NGO involved with this programme.

What is involved in the study – I am asking different people such as policy makers, program managers, health workers, community leaders, women who are the end-users of injectable contraceptives, and broader members of the community to participate in this study. About 100 people will participate in the study and all will be from Nigeria. If you agree to participate it means you will take part in a key informant interview. Each interview will last for about 60 minutes. In the interviews I will ask questions about your knowledge and your role in the implementation or use of injectable contraceptives as well as what you think might be the challenges and things that work well in implementing this program. There are no right and wrong answers; I am interested in understanding your opinions. Please note that I intend to audio-tape this interview.

Risks and Benefits of being involved in the study – There are no invasive procedures involved in this study, thus no physical harm is envisaged. However, you don't have to answer any question you are not comfortable with.

There are no direct benefits for participating in this study.

The participant will be given pertinent information on the study while involved in the project and after the results are available.

Participation is voluntary: Refusal to participate will involve no penalty or loss of benefits to which you are otherwise entitled. Also, you may discontinue participation at any time without penalty or loss of benefits to which you are otherwise entitled.

Reimbursements for transportation: Transportation costs incurred in order to participate in the study will be reimbursed at the rate of ₦100 per km.

Confidentiality: Efforts will be made to keep personal information confidential. Absolute confidentiality cannot be guaranteed. Personal information may be disclosed if required by law.

Organizations that may inspect and/or copy the research records for quality assurance and data analysis include groups such as the Research Ethics Committee. If results are published, may lead to individual / cohort identification.

Contact details of researcher – for further information / reporting of study related adverse events.

Dr Oluwaseun Akinyemi
Department of Health Policy and Management
College of Medicine
University of Ibadan
Ibadan, Nigeria
+234 803 502 0136
seunakinyemi@hotmail.com

Contact details of UI/UCH Ethics Committee chair (Nigeria) – for more information.

Prof. Catherine O. Falade
Chairperson, University of Ibadan/University College Hospital Ethics Committee
Institute for Advanced Medical Research and Training (IAMRAT)
College of Medicine, University of Ibadan
Ibadan, Nigeria
+234 803 326 4593
cfalade@comui.edu.ng

Contact details of REC administrator and chair (South Africa) – for more information.

Ms Zanele Ndlovu
Research Administrator
Human Research Ethics Committee (Medical)
University of the Witwatersrand
Johannesburg, South Africa
+27 11 717 1252
zanele.ndlovu@wits.ac.za

Participant Information Sheet for health workers

Study title: Scale up of community-based injectable contraceptives in Gombe State, Nigeria

Introduction:

Good day! I am Oluwaseun Akinyemi, a PhD candidate of the School of Public Health, University of the Witwatersrand, Johannesburg. I am doing research on the scale up of community-based injectable contraceptives in Gombe State, Nigeria. Research is just the process to learn the answer to a question. In this study we want to learn what the factors are that may influence the scale up of community-based distribution of injectable contraceptives in Nigeria, in order to understand possible facilitators and barriers.

Invitation to participate: I am therefore asking / inviting you to take part in this research study because you are a health worker who is involved with this programme.

What is involved in the study – I am asking different people such as policy makers, program managers, health workers, community leaders, women who are the end-users of injectable contraceptives, and broader members of the community to participate in this study. About 100 people will participate in the study and all will be from Nigeria. If you agree to participate it means you will take part in a key informant interview. Each interview will last for about 60 minutes. In the interviews I will ask questions about your knowledge and your role in the implementation or use of injectable contraceptives as well as what you think might be the challenges and things that work well in implementing this program. There are no right and wrong answers; I am interested in understanding your opinions. Please note that I intend to audio-tape this interview.

Risks and Benefits of being involved in the study – There are no invasive procedures involved in this study, thus no physical harm is envisaged. However, you don’t have to answer any question you are not comfortable with.

There are no direct benefits for participating in this study.

The participant will be given pertinent information on the study while involved in the project and after the results are available.

Participation is voluntary: Refusal to participate will involve no penalty or loss of benefits to which you are otherwise entitled. Also, you may discontinue participation at any time without penalty or loss of benefits to which you are otherwise entitled.

Reimbursements for transportation: Transportation costs incurred in order to participate in the study will be reimbursed at the rate of ₦100 per km.

Confidentiality: Efforts will be made to keep personal information confidential. Absolute confidentiality cannot be guaranteed. Personal information may be disclosed if required by law.

Organizations that may inspect and/or copy the research records for quality assurance and data analysis include groups such as the Research Ethics Committee. If results are published, may lead to individual / cohort identification.

Contact details of researcher – for further information / reporting of study related adverse events.

Dr Oluwaseun Akinyemi
Department of Health Policy and Management
College of Medicine
University of Ibadan
Ibadan, Nigeria
+234 803 502 0136
seunakinyemi@hotmail.com

Contact details of UI/UCH Ethics Committee chair (Nigeria) – for more information.

Prof. Catherine O. Falade
Chairperson, University of Ibadan/University College Hospital Ethics Committee
Institute for Advanced Medical Research and Training (IAMRAT)
College of Medicine, University of Ibadan
Ibadan, Nigeria
+234 803 326 4593
cfalade@comui.edu.ng

Contact details of REC administrator and chair (South Africa) – for more information.

Ms Zanele Ndlovu
Research Administrator
Human Research Ethics Committee (Medical)
University of the Witwatersrand
Johannesburg, South Africa
+27 11 717 1252
zanele.ndlovu@wits.ac.za

Participant Information Sheet for community leaders

Study title: Scale up of community-based injectable contraceptives in Gombe State, Nigeria

Introduction:

Good day! I am Oluwaseun Akinyemi, a PhD candidate of the School of Public Health, University of the Witwatersrand, Johannesburg. I am doing research on the scale up of community-based injectable contraceptives in Gombe State, Nigeria. Research is just the process to learn the answer to a question. In this study we want to learn what the factors are that may influence the scale up of community-based distribution of injectable contraceptives in Nigeria, in order to understand possible facilitators and barriers.

Invitation to participate: I am therefore asking / inviting you to take part in this research study because you are a community leader.

What is involved in the study – I am asking different people such as policy makers, program managers, health workers, community leaders, women who are the end-users of injectable contraceptives, and broader members of the community to participate in this study. About 100 people will participate in the study and all will be from Nigeria. If you agree to participate it means you will take part in a key informant interview. Each interview will last for about 60 minutes. In the interviews I will ask questions about your knowledge and your role in the implementation or use of injectable contraceptives as well as what you think might be the challenges and things that work well in implementing this program. There are no right and wrong answers; I am interested in understanding your opinions. Please note that I intend to audio-tape this interview.

Risks and Benefits of being involved in the study – There are no invasive procedures involved in this study, thus no physical harm is envisaged. However, you don't have to answer any question you are not comfortable with.

There are no direct benefits for participating in this study.

The participant will be given pertinent information on the study while involved in the project and after the results are available.

Participation is voluntary: Refusal to participate will involve no penalty or loss of benefits to which you are otherwise entitled. Also, you may discontinue participation at any time without penalty or loss of benefits to which you are otherwise entitled.

Reimbursements for transportation: Transportation costs incurred in order to participate in the study will be reimbursed at the rate of ₦100 per km.

Confidentiality: Efforts will be made to keep personal information confidential. Absolute confidentiality cannot be guaranteed. Personal information may be disclosed if required by law.

Organizations that may inspect and/or copy the research records for quality assurance and data analysis include groups such as the Research Ethics Committee. If results are published, may lead to individual / cohort identification.

Contact details of researcher – for further information / reporting of study related adverse events.

Dr Oluwaseun Akinyemi
Department of Health Policy and Management
College of Medicine
University of Ibadan
Ibadan, Nigeria
+234 803 502 0136
seunakinyemi@hotmail.com

Contact details of UI/UCH Ethics Committee chair (Nigeria) – for more information.

Prof. Catherine O. Falade
Chairperson, University of Ibadan/University College Hospital Ethics Committee
Institute for Advanced Medical Research and Training (IAMRAT)
College of Medicine, University of Ibadan
Ibadan, Nigeria
+234 803 326 4593
cfalade@comui.edu.ng

Contact details of REC administrator and chair (South Africa) – for more information.

Ms Zanele Ndlovu
Research Administrator
Human Research Ethics Committee (Medical)
University of the Witwatersrand
Johannesburg, South Africa
+27 11 717 1252
zanele.ndlovu@wits.ac.za

Participant Information Sheet for religious leaders

Study title: Scale up of community-based injectable contraceptives in Gombe State, Nigeria

Introduction:

Good day! I am Oluwaseun Akinyemi, a PhD candidate of the School of Public Health, University of the Witwatersrand, Johannesburg. I am doing research on the scale up of community-based injectable contraceptives in Gombe State, Nigeria. Research is just the process to learn the answer to a question. In this study we want to learn what the factors are that may influence the scale up of community-based distribution of injectable contraceptives in Nigeria, in order to understand possible facilitators and barriers.

Invitation to participate: I am therefore asking / inviting you to take part in this research study because you are a religious leader in this community.

What is involved in the study – I am asking different people such as policy makers, program managers, health workers, community leaders, women who are the end-users of injectable contraceptives, and broader members of the community to participate in this study. About 100 people will participate in the study and all will be from Nigeria. If you agree to participate it means you will take part in a key informant interview. Each interview will last for about 60 minutes. In the interviews I will ask questions about your knowledge and your role in the implementation or use of injectable contraceptives as well as what you think might be the challenges and things that work well in implementing this program. There are no right and wrong answers; I am interested in understanding your opinions. Please note that I intend to audio-tape this interview.

Risks and Benefits of being involved in the study – There are no invasive procedures involved in this study, thus no physical harm is envisaged. However, you don't have to answer any question you are not comfortable with.

There are no direct benefits for participating in this study.

The participant will be given pertinent information on the study while involved in the project and after the results are available.

Participation is voluntary: Refusal to participate will involve no penalty or loss of benefits to which you are otherwise entitled. Also, you may discontinue participation at any time without penalty or loss of benefits to which you are otherwise entitled.

Reimbursements for transportation: Transportation costs incurred in order to participate in the study will be reimbursed at the rate of ₦100 per km.

Confidentiality: Efforts will be made to keep personal information confidential. Absolute confidentiality cannot be guaranteed. Personal information may be disclosed if required by law.

Organizations that may inspect and/or copy the research records for quality assurance and data analysis include groups such as the Research Ethics Committee. If results are published, may lead to individual / cohort identification.

Contact details of researcher – for further information / reporting of study related adverse events.

Dr Oluwaseun Akinyemi
Department of Health Policy and Management
College of Medicine
University of Ibadan
Ibadan, Nigeria
+234 803 502 0136
seunakinyemi@hotmail.com

Contact details of UI/UCH Ethics Committee chair (Nigeria) – for more information.

Prof. Catherine O. Falade
Chairperson, University of Ibadan/University College Hospital Ethics Committee
Institute for Advanced Medical Research and Training (IAMRAT)
College of Medicine, University of Ibadan
Ibadan, Nigeria
+234 803 326 4593
cfalade@comui.edu.ng

Contact details of REC administrator and chair (South Africa) – for more information.

Ms Zanele Ndlovu
Research Administrator
Human Research Ethics Committee (Medical)
University of the Witwatersrand
Johannesburg, South Africa
+27 11 717 1252
zanele.ndlovu@wits.ac.za

Participant Information Sheet for women who are current users of injectable contraceptives

Study title: Scale up of community-based injectable contraceptives in Gombe State, Nigeria

Introduction:

Good day! I am Oluwaseun Akinyemi, a PhD candidate of the School of Public Health, University of the Witwatersrand, Johannesburg. I am doing research on the scale up of community-based injectable contraceptives in Gombe State, Nigeria. Research is just the process to learn the answer to a question. In this study we want to learn what the factors are that may influence the scale up of community-based distribution of injectable contraceptives in Nigeria, in order to understand possible facilitators and barriers.

Invitation to participate: I am therefore asking / inviting you to take part in this research study because you use an injectable contraceptive.

What is involved in the study – I am asking different people such as policy makers, program managers, health workers, community leaders, women who are the end-users of injectable contraceptives, and broader members of the community to participate in this study. About 100 people will participate in the study and all will be from Nigeria. If you agree to participate it means you will take part in a key informant interview. Each interview will last for about 60 minutes. In the interviews I will ask questions about your knowledge and your role in the implementation or use of injectable contraceptives as well as what you think might be the challenges and things that work well in implementing this program. There are no right and wrong answers; I am interested in understanding your opinions. Please note that I intend to audio-tape this interview.

Risks and Benefits of being involved in the study – There are no invasive procedures involved in this study, thus no physical harm is envisaged. However, you don't have to answer any question you are not comfortable with.

There are no direct benefits for participating in this study.

The participant will be given pertinent information on the study while involved in the project and after the results are available.

Participation is voluntary: Refusal to participate will involve no penalty or loss of benefits to which you are otherwise entitled. Also, you may discontinue participation at any time without penalty or loss of benefits to which you are otherwise entitled.

Reimbursements for transportation: Transportation costs incurred in order to participate in the study will be reimbursed at the rate of ₦100 per km.

Confidentiality: Efforts will be made to keep personal information confidential. However, confidentiality is not guaranteed in a focus group discussion. Personal information may be disclosed if required by law.

Organizations that may inspect and/or copy the research records for quality assurance and data analysis include groups such as the Research Ethics Committee. If results are published, may lead to individual / cohort identification.

Contact details of researcher – for further information / reporting of study related adverse events.

Dr Oluwaseun Akinyemi
Department of Health Policy and Management
College of Medicine
University of Ibadan
Ibadan, Nigeria
+234 803 502 0136
seunakinyemi@hotmail.com

Contact details of UI/UCH Ethics Committee chair (Nigeria) – for more information.

Prof. Catherine O. Falade
Chairperson, University of Ibadan/University College Hospital Ethics Committee
Institute for Advanced Medical Research and Training (IAMRAT)
College of Medicine, University of Ibadan
Ibadan, Nigeria
+234 803 326 4593
cfalade@comui.edu.ng

Contact details of REC administrator and chair (South Africa– for more information.

Ms Zanele Ndlovu
Research Administrator
Human Research Ethics Committee (Medical)
University of the Witwatersrand
Johannesburg, South Africa
+27 11 717 1252
zanele.ndlovu@wits.ac.za

Participant Information Sheet for women who do not use injectable contraceptives

Study title: Scale up of community-based injectable contraceptives in Gombe State, Nigeria

Introduction:

Good day! I am Oluwaseun Akinyemi, a PhD candidate of the School of Public Health, University of the Witwatersrand, Johannesburg. I am doing research on the scale up of community-based injectable contraceptives in Gombe State, Nigeria. Research is just the process to learn the answer to a question. In this study we want to learn what the factors are that may influence the scale up of community-based distribution of injectable contraceptives in Nigeria, in order to understand possible facilitators and barriers.

Invitation to participate: I am therefore asking / inviting you to take part in this research study because you do not use an injectable contraceptive.

What is involved in the study – I am asking different people such as policy makers, program managers, health workers, community leaders, women who are the end-users of injectable contraceptives, and broader members of the community to participate in this study. About 100 people will participate in the study and all will be from Nigeria. If you agree to participate it means you will take part in a key informant interview. Each interview will last for about 60 minutes. In the interviews I will ask questions about your knowledge and your role in the implementation or use of injectable contraceptives as well as what you think might be the challenges and things that work well in implementing this program. There are no right and wrong answers; I am interested in understanding your opinions. Please note that I intend to audio-tape this interview.

Risks and Benefits of being involved in the study – There are no invasive procedures involved in this study, thus no physical harm is envisaged. However, you don't have to answer any question you are not comfortable with.

There are no direct benefits for participating in this study.

The participant will be given pertinent information on the study while involved in the project and after the results are available.

Participation is voluntary: Refusal to participate will involve no penalty or loss of benefits to which you are otherwise entitled. Also, you may discontinue participation at any time without penalty or loss of benefits to which you are otherwise entitled.

Reimbursements for transportation: Transportation costs incurred in order to participate in the study will be reimbursed at the rate of ₦100 per km.

Confidentiality: Efforts will be made to keep personal information confidential. However, confidentiality is not guaranteed in a focus group discussion. Personal information may be disclosed if required by law.

Organizations that may inspect and/or copy the research records for quality assurance and data analysis include groups such as the Research Ethics Committee. If results are published, may lead to individual / cohort identification.

Contact details of researcher – for further information / reporting of study related adverse events.

Dr Oluwaseun Akinyemi
Department of Health Policy and Management
College of Medicine
University of Ibadan
Ibadan, Nigeria
+234 803 502 0136
seunakinyemi@hotmail.com

Contact details of UI/UCH Ethics Committee chair (Nigeria) – for more information.

Prof. Catherine O. Falade
Chairperson, University of Ibadan/University College Hospital Ethics Committee
Institute for Advanced Medical Research and Training (IAMRAT)
College of Medicine, University of Ibadan
Ibadan, Nigeria
+234 803 326 4593
cfalade@comui.edu.ng

Contact details of REC administrator and chair (South Africa– for more information.

Ms Zanele Ndlovu
Research Administrator
Human Research Ethics Committee (Medical)
University of the Witwatersrand
Johannesburg, South Africa
+27 11 717 1252
zanele.ndlovu@wits.ac.za

Participant Information Sheet for married men

Study title: Scale up of community-based injectable contraceptives in Gombe State, Nigeria

Introduction:

Good day! I am Oluwaseun Akinyemi, a PhD candidate of the School of Public Health, University of the Witwatersrand, Johannesburg. I am doing research on the scale up of community-based injectable contraceptives in Gombe State, Nigeria. Research is just the process to learn the answer to a question. In this study we want to learn what the factors are that may influence the scale up of community-based distribution of injectable contraceptives in Nigeria, in order to understand possible facilitators and barriers.

Invitation to participate: I am therefore asking / inviting you to take part in this research study because you are a married man.

What is involved in the study – I am asking different people such as policy makers, program managers, health workers, community leaders, women who are the end-users of injectable contraceptives, and broader members of the community to participate in this study. About 100 people will participate in the study and all will be from Nigeria. If you agree to participate it means you will take part in a key informant interview. Each interview will last for about 60 minutes. In the interviews I will ask questions about your knowledge and your role in the implementation or use of injectable contraceptives as well as what you think might be the challenges and things that work well in implementing this program. There are no right and wrong answers; I am interested in understanding your opinions. Please note that I intend to audio-tape this interview.

Risks and Benefits of being involved in the study – There are no invasive procedures involved in this study, thus no physical harm is envisaged. However, you don't have to answer any question you are not comfortable with.

There are no direct benefits for participating in this study.

The participant will be given pertinent information on the study while involved in the project and after the results are available.

Participation is voluntary: Refusal to participate will involve no penalty or loss of benefits to which you are otherwise entitled. Also, you may discontinue participation at any time without penalty or loss of benefits to which you are otherwise entitled.

Reimbursements for transportation: Transportation costs incurred in order to participate in the study will be reimbursed at the rate of ₦100 per km.

Confidentiality: Efforts will be made to keep personal information confidential. However, confidentiality is not guaranteed in a focus group discussion. Personal information may be disclosed if required by law.

Organizations that may inspect and/or copy the research records for quality assurance and data analysis include groups such as the Research Ethics Committee. If results are published, may lead to individual / cohort identification.

Contact details of researcher – for further information / reporting of study related adverse events.

Dr Oluwaseun Akinyemi
Department of Health Policy and Management
College of Medicine
University of Ibadan
Ibadan, Nigeria
+234 803 502 0136
seunakinyemi@hotmail.com

Contact details of UI/UCH Ethics Committee chair (Nigeria) – for more information.

Prof. Catherine O. Falade
Chairperson, University of Ibadan/University College Hospital Ethics Committee
Institute for Advanced Medical Research and Training (IAMRAT)
College of Medicine, University of Ibadan
Ibadan, Nigeria
+234 803 326 4593
cfalade@comui.edu.ng

Contact details of REC administrator and chair (South Africa) – for more information.

Ms Zanele Ndlovu
Research Administrator
Human Research Ethics Committee (Medical)
University of the Witwatersrand
Johannesburg, South Africa
+27 11 717 1252
zanele.ndlovu@wits.ac.za

Participant Information Sheet for unmarried/single men

Study title: Scale up of community-based injectable contraceptives in Gombe State, Nigeria

Introduction:

Good day! I am Oluwaseun Akinyemi, a PhD candidate of the School of Public Health, University of the Witwatersrand, Johannesburg. I am doing research on the scale up of community-based injectable contraceptives in Gombe State, Nigeria. Research is just the process to learn the answer to a question. In this study we want to learn what the factors are that may influence the scale up of community-based distribution of injectable contraceptives in Nigeria, in order to understand possible facilitators and barriers.

Invitation to participate: I am therefore asking / inviting you to take part in this research study because you are an unmarried/ single man.

What is involved in the study – I am asking different people such as policy makers, program managers, health workers, community leaders, women who are the end-users of injectable contraceptives, and broader members of the community to participate in this study. About 100 people will participate in the study and all will be from Nigeria. If you agree to participate it means you will take part in a key informant interview. Each interview will last for about 60 minutes. In the interviews I will ask questions about your knowledge and your role in the implementation or use of injectable contraceptives as well as what you think might be the challenges and things that work well in implementing this program. There are no right and wrong answers; I am interested in understanding your opinions. Please note that I intend to audio-tape this interview.

Risks and Benefits of being involved in the study – There are no invasive procedures involved in this study, thus no physical harm is envisaged. However, you don't have to answer any question you are not comfortable with.

There are no direct benefits for participating in this study.

The participant will be given pertinent information on the study while involved in the project and after the results are available.

Participation is voluntary: Refusal to participate will involve no penalty or loss of benefits to which you are otherwise entitled. Also, you may discontinue participation at any time without penalty or loss of benefits to which you are otherwise entitled.

Reimbursements for transportation: Transportation costs incurred in order to participate in the study will be reimbursed at the rate of ₦100 per km.

Confidentiality: Efforts will be made to keep personal information confidential. However, confidentiality is not guaranteed in a focus group discussion. Personal information may be disclosed if required by law.

Organizations that may inspect and/or copy the research records for quality assurance and data analysis include groups such as the Research Ethics Committee. If results are published, may lead to individual / cohort identification.

Contact details of researcher – for further information / reporting of study related adverse events.

Dr Oluwaseun Akinyemi
Department of Health Policy and Management
College of Medicine
University of Ibadan
Ibadan, Nigeria
+234 803 502 0136
seunakinyemi@hotmail.com

Contact details of UI/UCH Ethics Committee chair (Nigeria) – for more information.

Prof. Catherine O. Falade
Chairperson, University of Ibadan/University College Hospital Ethics Committee
Institute for Advanced Medical Research and Training (IAMRAT)
College of Medicine, University of Ibadan
Ibadan, Nigeria
+234 803 326 4593
cfalade@comui.edu.ng

Contact details of REC administrator and chair (South Africa) – for more information.

Ms Zanele Ndlovu
Research Administrator
Human Research Ethics Committee (Medical)
University of the Witwatersrand
Johannesburg, South Africa
+27 11 717 1252
zanele.ndlovu@wits.ac.za

Informed Consent Agreement for Senior MoH officials

Study title: Scale up of community-based injectable contraceptives in Gombe State, Nigeria

SERIAL NUMBER ___________________

Background and purpose: One of the health innovations that have been piloted with significant success around the world, including Nigeria, is the community-based distribution of injectable contraceptives. This study will contribute to providing evidence towards aiding our understanding of the barriers and facilitators to the introduction, dissemination, diffusion and integration of the community-based distribution of injectable contraceptives intervention in Nigeria.

Procedures: You have been selected as one of this study's participants for a key informant interview. Each interview will last for about 60 minutes. In the interviews I will ask questions about your knowledge and your role in the implementation or use of injectable contraceptives as well as what you think might be the challenges and things that work well in implementing this program.

Potential Risks: There are no health risks associated with the interviews and discussions.

Potential benefits: The findings of this study will help enhance the scale up of injectable contraceptives in Nigeria thereby helping to promote maternal and child health as well as healthier families.

Reimbursements for "out of pocket" expenses: Transportation costs incurred in order to participate in the study will be reimbursed at the rate of ₦100 per km.

Withdrawal from participation: If you agree to take part you are free to end your participation in this study at any time you want without having to give reasons.

Additional Information: In case you have any question or concern about this study please feel free to contact Dr Oluwaseun Akinyemi, Department of Health Policy and Management, College of Medicine, University of Ibadan Ibadan, Nigeria (Tel. No.: +234 803 502 0136; Email: seunakinyemi@hotmail.com)

Informed consent record: Please read this statement carefully before you sign and if you agree on participating, please fill free to sign this form below to affirm your consent.

I, ________________________________ clearly understand the aims of the project titled **"Scale up of community-based injectable contraceptives in Gombe State, Nigeria"** which has been explained to me by the researcher. I have been given the chance to ask questions and I am satisfied with the answers to all of my questions and I agree to participate in the study. I understand that I may revoke my consent and leave the study at any stage, if I wish so, with no negative consequences.

Participant’s name: ____

Participant’s signature or thumbprint: ____________________ Date: ______________

Witness Name (As appropriate): ____

Witness signature (As appropriate): ___________________ Date: _________________

Study team member’s statement: I, the undersigned, have explained to the participant in a language that s/he understands; the procedures to be followed in the study, the risks and benefits involved, and the obligations of the study team.

___________________________ ___________________________ _______________

Name Signature Date

Informed Consent Agreement for program managers

Study title: Scale up of community-based injectable contraceptives in Gombe State, Nigeria

SERIAL NUMBER ___________________

Background and purpose: One of the health innovations that have been piloted with significant success around the world, including Nigeria, is the community-based distribution of injectable contraceptives. This study will contribute to providing evidence towards aiding our understanding of the barriers and facilitators to the introduction, dissemination, diffusion and integration of the community-based distribution of injectable contraceptives intervention in Nigeria.

Procedures: You have been selected as one of this study's participants for a key informant interview. Each interview will last for about 60 minutes. In the interviews I will ask questions about your knowledge and your role in the implementation or use of injectable contraceptives as well as what you think might be the challenges and things that work well in implementing this program.

Potential Risks: There are no health risks associated with the interviews and discussions.

Potential benefits: The findings of this study will help enhance the scale up of injectable contraceptives in Nigeria thereby helping to promote maternal and child health as well as healthier families.

Reimbursements for "out of pocket" expenses: Transportation costs incurred in order to participate in the study will be reimbursed at the rate of ₦100 per km.

Withdrawal from participation: If you agree to take part you are free to end your participation in this study at any time you want without having to give reasons.

Additional Information: In case you have any question or concern about this study please feel free to contact Dr Oluwaseun Akinyemi, Department of Health Policy and Management, College of Medicine, University of Ibadan Ibadan, Nigeria (Tel. No.: +234 803 502 0136; Email: seunakinyemi@hotmail.com)

Informed consent record: Please read this statement carefully before you sign and if you agree on participating, please fill free to sign this form below to affirm your consent.

I, ______________________________ clearly understand the aims of the project titled **"Scale up of community-based injectable contraceptives in Gombe State, Nigeria"** which has been explained to me by the researcher. I have been given the chance to ask questions and I am satisfied with the answers to all of my questions and I agree to participate in the study. I understand that I may revoke my consent and leave the study at any stage, if I wish so, with no negative consequences.

Participant's name: ____

Participant's signature or thumbprint: ____________________ Date: ______________

Witness Name (As appropriate): ____

Witness signature (As appropriate): ___________________ Date: _________________

Study team member's statement: I, the undersigned, have explained to the participant in a language that s/he understands; the procedures to be followed in the study, the risks and benefits involved, and the obligations of the study team.

___________________________ ___________________________ _______________

Name Signature Date

Informed Consent Agreement for health workers

Study title: Scale up of community-based injectable contraceptives in Gombe State, Nigeria

SERIAL NUMBER ___________________

Background and purpose: One of the health innovations that have been piloted with significant success around the world, including Nigeria, is the community-based distribution of injectable contraceptives. This study will contribute to providing evidence towards aiding our understanding of the barriers and facilitators to the introduction, dissemination, diffusion and integration of the community-based distribution of injectable contraceptives intervention in Nigeria.

Procedures: You have been selected as one of this study's participants for an in-depth interview. Each interview will last for about 60 minutes. In the interviews and discussions I will ask questions about your knowledge and your role in the implementation or use of injectable contraceptives as well as what you think might be the challenges and things that work well in implementing this program.

Potential Risks: There are no health risks associated with the interviews and discussions.

Potential benefits: The findings of this study will help enhance the scale up of injectable contraceptives in Nigeria thereby helping to promote maternal and child health as well as healthier families.

Reimbursements for "out of pocket" expenses: Transportation costs incurred in order to participate in the study will be reimbursed at the rate of ₦100 per km.

Withdrawal from participation: If you agree to take part you are free to end your participation in this study at any time you want without having to give reasons.

Additional Information: In case you have any question or concern about this study please feel free to contact Dr Oluwaseun Akinyemi, Department of Health Policy and Management, College of Medicine, University of Ibadan Ibadan, Nigeria (Tel. No.: +234 803 502 0136; Email: seunakinyemi@hotmail.com)

Informed consent record: Please read this statement carefully before you sign and if you agree on participating, please fill free to sign this form below to affirm your consent.

I, ______________________________ clearly understand the aims of the project titled **"Scale up of community-based injectable contraceptives in Gombe State, Nigeria"** which has been explained to me by the researcher. I have been given the chance to ask questions and I am satisfied with the answers to all of my questions and I agree to participate in the study. I understand that I may revoke my consent and leave the study at any stage, if I wish so, with no negative consequences.

Participant's name: ____

Participant's signature or thumbprint: ____________________ Date: ______________

Witness Name (As appropriate): ____

Witness signature (As appropriate): ___________________ Date: _________________

Study team member's statement: I, the undersigned, have explained to the participant in a language that s/he understands; the procedures to be followed in the study, the risks and benefits involved, and the obligations of the study team.

___________________________ ___________________________ _______________

Name Signature Date

Informed Consent Agreement for community leaders

Study title: Scale up of community-based injectable contraceptives in Gombe State, Nigeria

SERIAL NUMBER ___________________

Background and purpose: One of the health innovations that have been piloted with significant success around the world, including Nigeria, is the community-based distribution of injectable contraceptives. This study will contribute to providing evidence towards aiding our understanding of the barriers and facilitators to the introduction, dissemination, diffusion and integration of the community-based distribution of injectable contraceptives intervention in Nigeria.

Procedures: You have been selected as one of this study's participants for an in-depth interview. Each interview will last for about 60 minutes. In the interviews and discussions I will ask questions about your knowledge and your role in the implementation or use of injectable contraceptives as well as what you think might be the challenges and things that work well in implementing this program.

Potential Risks: There are no health risks associated with the interviews and discussions.

Potential benefits: The findings of this study will help enhance the scale up of injectable contraceptives in Nigeria thereby helping to promote maternal and child health as well as healthier families.

Reimbursements for "out of pocket" expenses: Transportation costs incurred in order to participate in the study will be reimbursed at the rate of ₦100 per km.

Withdrawal from participation: If you agree to take part you are free to end your participation in this study at any time you want without having to give reasons.

Additional Information: In case you have any question or concern about this study please feel free to contact Dr Oluwaseun Akinyemi, Department of Health Policy and Management, College of Medicine, University of Ibadan Ibadan, Nigeria (Tel. No.: +234 803 502 0136; Email: seunakinyemi@hotmail.com)

Informed consent record: Please read this statement carefully before you sign and if you agree on participating, please fill free to sign this form below to affirm your consent.

I, ______________________________ clearly understand the aims of the project titled **"Scale up of community-based injectable contraceptives in Gombe State, Nigeria"** which has been explained to me by the researcher. I have been given the chance to ask questions and I am satisfied with the answers to all of my questions and I agree to participate in the study. I understand that I may revoke my consent and leave the study at any stage, if I wish so, with no negative consequences.

Participant's name: ____

Participant's signature or thumbprint: ____________________ Date: ______________

Witness Name (As appropriate): ____

Witness signature (As appropriate): ___________________ Date: _________________

Study team member's statement: I, the undersigned, have explained to the participant in a language that s/he understands; the procedures to be followed in the study, the risks and benefits involved, and the obligations of the study team.

___________________________ ___________________________ _______________

Name Signature Date

Informed Consent Agreement for religious leaders

Study title: Scale up of community-based injectable contraceptives in Gombe State, Nigeria

SERIAL NUMBER ___________________

Background and purpose: One of the health innovations that have been piloted with significant success around the world, including Nigeria, is the community-based distribution of injectable contraceptives. This study will contribute to providing evidence towards aiding our understanding of the barriers and facilitators to the introduction, dissemination, diffusion and integration of the community-based distribution of injectable contraceptives intervention in Nigeria.

Procedures: You have been selected as one of this study's participants for an in-depth interview. Each interview will last for about 60 minutes. In the interviews and discussions I will ask questions about your knowledge and your role in the implementation or use of injectable contraceptives as well as what you think might be the challenges and things that work well in implementing this program.

Potential Risks: There are no health risks associated with the interviews and discussions.

Potential benefits: The findings of this study will help enhance the scale up of injectable contraceptives in Nigeria thereby helping to promote maternal and child health as well as healthier families.

Reimbursements for "out of pocket" expenses: Transportation costs incurred in order to participate in the study will be reimbursed at the rate of ₦100 per km.

Withdrawal from participation: If you agree to take part you are free to end your participation in this study at any time you want without having to give reasons.

Additional Information: In case you have any question or concern about this study please feel free to contact Dr Oluwaseun Akinyemi, Department of Health Policy and Management, College of Medicine, University of Ibadan Ibadan, Nigeria (Tel. No.: +234 803 502 0136; Email: seunakinyemi@hotmail.com)

Informed consent record: Please read this statement carefully before you sign and if you agree on participating, please fill free to sign this form below to affirm your consent.

I, ______________________________ clearly understand the aims of the project titled **"Scale up of community-based injectable contraceptives in Gombe State, Nigeria"** which has been explained to me by the researcher. I have been given the chance to ask questions and I am satisfied with the answers to all of my questions and I agree to participate in the study. I understand that I may revoke my consent and leave the study at any stage, if I wish so, with no negative consequences.

Participant’s name: ____

Participant’s signature or thumbprint: ____________________ Date: ______________

Witness Name (As appropriate): ____

Witness signature (As appropriate): ___________________ Date: _________________

Study team member’s statement: I, the undersigned, have explained to the participant in a language that s/he understands; the procedures to be followed in the study, the risks and benefits involved, and the obligations of the study team.

___________________________ ___________________________ _______________

Name Signature Date

Informed Consent Agreement for women who are current users of injectable contraceptives

Study title: Scale up of community-based injectable contraceptives in Gombe State, Nigeria

SERIAL NUMBER ___________________

Background and purpose: One of the health innovations that have been piloted with significant success around the world, including Nigeria, is the community-based distribution of injectable contraceptives. This study will contribute to providing evidence towards aiding our understanding of the barriers and facilitators to the introduction, dissemination, diffusion and integration of the community-based distribution of injectable contraceptives intervention in Nigeria.

Procedures: You have been selected as one of this study's participants for a focus group discussion or a questionnaire interview. Each interview or focus group discussion will last for about 60 minutes. In the interviews and discussions I will ask questions about your knowledge and your role in the implementation or use of injectable contraceptives as well as what you think might be the challenges and things that work well in implementing this program.

Potential Risks: There are no health risks associated with the interviews and discussions.

Potential benefits: The findings of this study will help enhance the scale up of injectable contraceptives in Nigeria thereby helping to promote maternal and child health as well as healthier families.

Reimbursements for "out of pocket" expenses: Transportation costs incurred in order to participate in the study will be reimbursed at the rate of ₦100 per km.

Withdrawal from participation: If you agree to take part you are free to end your participation in this study at any time you want without having to give reasons.

Additional Information: In case you have any question or concern about this study please feel free to contact Dr Oluwaseun Akinyemi, Department of Health Policy and Management, College of Medicine, University of Ibadan Ibadan, Nigeria (Tel. No.: +234 803 502 0136; Email: seunakinyemi@hotmail.com)

Informed consent record: Please read this statement carefully before you sign and if you agree on participating, please fill free to sign this form below to affirm your consent.

I, _______________________________ clearly understand the aims of the project titled **"Scale up of community-based injectable contraceptives in Gombe State, Nigeria"** which has been explained to me by the researcher. I have been given the chance to ask questions and I am satisfied with the answers to all of my questions and I agree to participate in the study. I understand

that I may revoke my consent and leave the study at any stage, if I wish so, with no negative consequences.

Participant’s name: ____

Participant’s signature or thumbprint: _________ ___________ Date: ______________

Witness Name (As appropriate): ____

Witness signature (As appropriate): ___________________ Date: _________________

Study team member’s statement: I, the undersigned, have explained to the participant in a language that s/he understands; the procedures to be followed in the study, the risks and benefits involved, and the obligations of the study team.

___________________________ ___________________________ _______________

Name Signature Date

Informed Consent Agreement for women who do not use injectable contraceptives

Study title: Scale up of community-based injectable contraceptives in Gombe State, Nigeria

SERIAL NUMBER ___________________

Background and purpose: One of the health innovations that have been piloted with significant success around the world, including Nigeria, is the community-based distribution of injectable contraceptives. This study will contribute to providing evidence towards aiding our understanding of the barriers and facilitators to the introduction, dissemination, diffusion and integration of the community-based distribution of injectable contraceptives intervention in Nigeria.

Procedures: You have been selected as one of this study's participants for a focus group discussion. Each focus group discussion will last for about 60 minutes. In the interviews and discussions I will ask questions about your knowledge and your role in the implementation or use of injectable contraceptives as well as what you think might be the challenges and things that work well in implementing this program.

Potential Risks: There are no health risks associated with the interviews and discussions.

Potential benefits: The findings of this study will help enhance the scale up of injectable contraceptives in Nigeria thereby helping to promote maternal and child health as well as healthier families.

Reimbursements for "out of pocket" expenses: Transportation costs incurred in order to participate in the study will be reimbursed at the rate of ₦100 per km.

Withdrawal from participation: If you agree to take part you are free to end your participation in this study at any time you want without having to give reasons.

Additional Information: In case you have any question or concern about this study please feel free to contact Dr Oluwaseun Akinyemi, Department of Health Policy and Management, College of Medicine, University of Ibadan Ibadan, Nigeria (Tel. No.: +234 803 502 0136; Email: seunakinyemi@hotmail.com)

Informed consent record: Please read this statement carefully before you sign and if you agree on participating, please fill free to sign this form below to affirm your consent.

I, ________________________________ clearly understand the aims of the project titled **"Scale up of community-based injectable contraceptives in Gombe State, Nigeria"** which has been explained to me by the researcher. I have been given the chance to ask questions and I am satisfied with the answers to all of my questions and I agree to participate in the study. I understand that I may revoke my consent and leave the study at any stage, if I wish so, with no negative consequences.

Participant's name: ____

Participant's signature or thumbprint: __________________ Date: ____________

Witness Name (As appropriate): _______________________________________

Witness signature (As appropriate): _________________ Date: _______________

Study team member's statement: I, the undersigned, have explained to the participant in a language that s/he understands; the procedures to be followed in the study, the risks and benefits involved, and the obligations of the study team.

________________________ ________________________ _____________

Name Signature Date

Informed Consent Agreement for married men

Study title: Scale up of community-based injectable contraceptives in Gombe State, Nigeria

SERIAL NUMBER ___________________

Background and purpose: One of the health innovations that have been piloted with significant success around the world, including Nigeria, is the community-based distribution of injectable contraceptives. This study will contribute to providing evidence towards aiding our understanding of the barriers and facilitators to the introduction, dissemination, diffusion and integration of the community-based distribution of injectable contraceptives intervention in Nigeria.

Procedures: You have been selected as one of this study's participants for a focus group discussion. Each focus group discussion will last for about 60 minutes. In the interviews and discussions I will ask questions about your knowledge and your role in the implementation or use of injectable contraceptives as well as what you think might be the challenges and things that work well in implementing this program.

Potential Risks: There are no health risks associated with the interviews and discussions.

Potential benefits: The findings of this study will help enhance the scale up of injectable contraceptives in Nigeria thereby helping to promote maternal and child health as well as healthier families.

Reimbursements for "out of pocket" expenses: Transportation costs incurred in order to participate in the study will be reimbursed at the rate of ₦100 per km.

Withdrawal from participation: If you agree to take part you are free to end your participation in this study at any time you want without having to give reasons.

Additional Information: In case you have any question or concern about this study please feel free to contact Dr Oluwaseun Akinyemi, Department of Health Policy and Management, College of Medicine, University of Ibadan Ibadan, Nigeria (Tel. No.: +234 803 502 0136; Email: seunakinyemi@hotmail.com)

Informed consent record: Please read this statement carefully before you sign and if you agree on participating, please fill free to sign this form below to affirm your consent.

I, ______________________________ clearly understand the aims of the project titled **"Scale up of community-based injectable contraceptives in Gombe State, Nigeria"** which has been explained to me by the researcher. I have been given the chance to ask questions and I am satisfied with the answers to all of my questions and I agree to participate in the study. I understand that I may revoke my consent and leave the study at any stage, if I wish so, with no negative consequences.

Participant's name: ____

Participant's signature or thumbprint: ____________________ Date: ______________

Witness Name (As appropriate): ____

Witness signature (As appropriate): ___________________ Date: _________________

Study team member's statement: I, the undersigned, have explained to the participant in a language that s/he understands; the procedures to be followed in the study, the risks and benefits involved, and the obligations of the study team.

____________________________ ____________________________ ________________

Name Signature Date

Informed Consent Agreement for unmarried men

Study title: Scale up of community-based injectable contraceptives in Gombe State, Nigeria

SERIAL NUMBER ____________________

Background and purpose: One of the health innovations that have been piloted with significant success around the world, including Nigeria, is the community-based distribution of injectable contraceptives. This study will contribute to providing evidence towards aiding our understanding of the barriers and facilitators to the introduction, dissemination, diffusion and integration of the community-based distribution of injectable contraceptives intervention in Nigeria.

Procedures: You have been selected as one of this study's participants for a focus group discussion. Each focus group discussion will last for about 60 minutes. In the interviews and discussions I will ask questions about your knowledge and your role in the implementation or use of injectable contraceptives as well as what you think might be the challenges and things that work well in implementing this program.

Potential Risks: There are no health risks associated with the interviews and discussions.

Potential benefits: The findings of this study will help enhance the scale up of injectable contraceptives in Nigeria thereby helping to promote maternal and child health as well as healthier families.

Reimbursements for "out of pocket" expenses: Transportation costs incurred in order to participate in the study will be reimbursed at the rate of ₦100 per km.

Withdrawal from participation: If you agree to take part you are free to end your participation in this study at any time you want without having to give reasons.

Additional Information: In case you have any question or concern about this study please feel free to contact Dr Oluwaseun Akinyemi, Department of Health Policy and Management, College of Medicine, University of Ibadan Ibadan, Nigeria (Tel. No.: +234 803 502 0136; Email: seunakinyemi@hotmail.com)

Informed consent record: Please read this statement carefully before you sign and if you agree on participating, please fill free to sign this form below to affirm your consent.

I, _______________________________ clearly understand the aims of the project titled **"Scale up of community-based injectable contraceptives in Gombe State, Nigeria"** which has been explained to me by the researcher. I have been given the chance to ask questions and I am satisfied with the answers to all of my questions and I agree to participate in the study. I understand that I may revoke my consent and leave the study at any stage, if I wish so, with no negative consequences.

Participant's name: ____

Participant's signature or thumbprint: ____________________ Date: ______________

Witness Name (As appropriate): ____

Witness signature (As appropriate): ___________________ Date: _________________

Study team member's statement: I, the undersigned, have explained to the participant in a language that s/he understands; the procedures to be followed in the study, the risks and benefits involved, and the obligations of the study team.

___________________________ ___________________________ _______________

Name Signature Date

Informed Consent Agreement to audio record interview senior MoH officials

Study title: Scale up of community-based injectable contraceptives in Gombe State, Nigeria

SERIAL NUMBER ___________________

Background and purpose: One of the health innovations that have been piloted with significant success around the world, including Nigeria, is the community-based distribution of injectable contraceptives.

Additional Information: In case you have any question or concern about this study please feel free to contact Dr Oluwaseun Akinyemi, Department of Health Policy and Management, College of Medicine, University of Ibadan Ibadan, Nigeria (Tel. No.: +234 803 502 0136; Email: seunakinyemi@hotmail.com)

Informed consent to record: Please read this statement carefully before you sign and if you agree that the interview / discussion with you should be audio recorded, please fill free to sign this form below to affirm your consent.

I, ______________________________ clearly understand the aims of the project titled **"Scale up of community-based injectable contraceptives in Gombe State, Nigeria"** which has been explained to me by the researcher. I have been given the chance to ask questions and I am satisfied with the answers to all of my questions and I agree to participate in the study. I understand that I may revoke my consent and leave the study at any stage, if I wish so, with no negative consequences. I agree that the interview / discussion with me should be audio recorded. I understand that the recording is for research purposes only.

Participant's name: ____

Participant's signature or thumbprint: ____________________ Date: ______________

Witness Name (As appropriate): ____

Witness signature (As appropriate): ___________________ Date: _________________

Study team member's statement: I, the undersigned, have explained to the participant in a language that s/he understands; the procedures to be followed in the study (including audio recording of the interview), the risks and benefits involved, and the obligations of the study team.

___________________________ ___________________________ _______________

Name Signature Date

Informed Consent Agreement to audio record interview with program managers

Study title: Scale up of community-based injectable contraceptives in Gombe State, Nigeria

SERIAL NUMBER ___________________

Background and purpose: One of the health innovations that have been piloted with significant success around the world, including Nigeria, is the community-based distribution of injectable contraceptives.

Additional Information: In case you have any question or concern about this study please feel free to contact Dr Oluwaseun Akinyemi, Department of Health Policy and Management, College of Medicine, University of Ibadan Ibadan, Nigeria (Tel. No.: +234 803 502 0136; Email: seunakinyemi@hotmail.com)

Informed consent to record: Please read this statement carefully before you sign and if you agree that the interview / discussion with you should be audio recorded, please fill free to sign this form below to affirm your consent.

I, ______________________________ clearly understand the aims of the project titled **"Scale up of community-based injectable contraceptives in Gombe State, Nigeria"** which has been explained to me by the researcher. I have been given the chance to ask questions and I am satisfied with the answers to all of my questions and I agree to participate in the study. I understand that I may revoke my consent and leave the study at any stage, if I wish so, with no negative consequences. I agree that the interview / discussion with me should be audio recorded. I understand that the recording is for research purposes only.

Participant's name: ____

Participant's signature or thumbprint: _____________________ Date: ______________

Witness Name (As appropriate): ____

Witness signature (As appropriate): ___________________ Date: _________________

Study team member's statement: I, the undersigned, have explained to the participant in a language that s/he understands; the procedures to be followed in the study (including audio recording of the interview), the risks and benefits involved, and the obligations of the study team.

___________________________ ___________________________ _______________

Name Signature Date

Informed Consent Agreement to audio record interview with health workers

Study title: Scale up of community-based injectable contraceptives in Gombe State, Nigeria

SERIAL NUMBER ___________________

Background and purpose: One of the health innovations that have been piloted with significant success around the world, including Nigeria, is the community-based distribution of injectable contraceptives.

Additional Information: In case you have any question or concern about this study please feel free to contact Dr Oluwaseun Akinyemi, Department of Health Policy and Management, College of Medicine, University of Ibadan Ibadan, Nigeria (Tel. No.: +234 803 502 0136; Email: seunakinyemi@hotmail.com)

Informed consent to record: Please read this statement carefully before you sign and if you agree that the interview / discussion with you should be audio recorded, please fill free to sign this form below to affirm your consent.

I, ______________________________ clearly understand the aims of the project titled **"Scale up of community-based injectable contraceptives in Gombe State, Nigeria"** which has been explained to me by the researcher. I have been given the chance to ask questions and I am satisfied with the answers to all of my questions and I agree to participate in the study. I understand that I may revoke my consent and leave the study at any stage, if I wish so, with no negative consequences. I agree that the interview / discussion with me should be audio recorded. I understand that the recording is for research purposes only.

Participant's name: ____

Participant's signature or thumbprint: ____________________ Date: ______________

Witness Name (As appropriate): ____

Witness signature (As appropriate): ___________________ Date: _________________

Study team member's statement: I, the undersigned, have explained to the participant in a language that s/he understands; the procedures to be followed in the study (including audio recording of the interview), the risks and benefits involved, and the obligations of the study team.

___________________________ ___________________________ _______________

Name Signature Date

Informed Consent Agreement to audio record interview with community leaders

Study title: Scale up of community-based injectable contraceptives in Gombe State, Nigeria

SERIAL NUMBER ___________________

Background and purpose: One of the health innovations that have been piloted with significant success around the world, including Nigeria, is the community-based distribution of injectable contraceptives.

Additional Information: In case you have any question or concern about this study please feel free to contact Dr Oluwaseun Akinyemi, Department of Health Policy and Management, College of Medicine, University of Ibadan Ibadan, Nigeria (Tel. No.: +234 803 502 0136; Email: seunakinyemi@hotmail.com)

Informed consent to record: Please read this statement carefully before you sign and if you agree that the interview / discussion with you should be audio recorded, please fill free to sign this form below to affirm your consent.

I, ______________________________ clearly understand the aims of the project titled **"Scale up of community-based injectable contraceptives in Gombe State, Nigeria"** which has been explained to me by the researcher. I have been given the chance to ask questions and I am satisfied with the answers to all of my questions and I agree to participate in the study. I understand that I may revoke my consent and leave the study at any stage, if I wish so, with no negative consequences. I agree that the interview / discussion with me should be audio recorded. I understand that the recording is for research purposes only.

Participant's name: ____

Participant's signature or thumbprint: ____________________ Date: ______________

Witness Name (As appropriate): ____

Witness signature (As appropriate): ___________________ Date: _________________

Study team member's statement: I, the undersigned, have explained to the participant in a language that s/he understands; the procedures to be followed in the study (including audio recording of the interview), the risks and benefits involved, and the obligations of the study team.

___________________________ ___________________________ _______________

Name Signature Date

Informed Consent Agreement to audio record interview with religious leaders

Study title: Scale up of community-based injectable contraceptives in Gombe State, Nigeria

SERIAL NUMBER ___________________

Background and purpose: One of the health innovations that have been piloted with significant success around the world, including Nigeria, is the community-based distribution of injectable contraceptives.

Additional Information: In case you have any question or concern about this study please feel free to contact Dr Oluwaseun Akinyemi, Department of Health Policy and Management, College of Medicine, University of Ibadan Ibadan, Nigeria (Tel. No.: +234 803 502 0136; Email: seunakinyemi@hotmail.com)

Informed consent to record: Please read this statement carefully before you sign and if you agree that the interview / discussion with you should be audio recorded, please fill free to sign this form below to affirm your consent.

I, ______________________________ clearly understand the aims of the project titled **"Scale up of community-based injectable contraceptives in Gombe State, Nigeria"** which has been explained to me by the researcher. I have been given the chance to ask questions and I am satisfied with the answers to all of my questions and I agree to participate in the study. I understand that I may revoke my consent and leave the study at any stage, if I wish so, with no negative consequences. I agree that the interview / discussion with me should be audio recorded. I understand that the recording is for research purposes only.

Participant's name: ____

Participant's signature or thumbprint: _____________________ Date: ______________

Witness Name (As appropriate): ____

Witness signature (As appropriate): ___________________ Date: _________________

Study team member's statement: I, the undersigned, have explained to the participant in a language that s/he understands; the procedures to be followed in the study (including audio recording of the interview), the risks and benefits involved, and the obligations of the study team.

___________________________ ___________________________ _______________

Name Signature Date

Informed Consent Agreement to audio record focus group discussion with women who currently use injectable contraceptive

Study title: Scale up of community-based injectable contraceptives in Gombe State, Nigeria

SERIAL NUMBER ___________________

Background and purpose: One of the health innovations that have been piloted with significant success around the world, including Nigeria, is the community-based distribution of injectable contraceptives.

Additional Information: In case you have any question or concern about this study please feel free to contact Dr Oluwaseun Akinyemi, Department of Health Policy and Management, College of Medicine, University of Ibadan Ibadan, Nigeria (Tel. No.: +234 803 502 0136; Email: seunakinyemi@hotmail.com)

Informed consent to record: Please read this statement carefully before you sign and if you agree that the interview / discussion with you should be audio recorded, please fill free to sign this form below to affirm your consent.

I, ________________________________ clearly understand the aims of the project titled **"Scale up of community-based injectable contraceptives in Gombe State, Nigeria"** which has been explained to me by the researcher. I have been given the chance to ask questions and I am satisfied with the answers to all of my questions and I agree to participate in the study. I understand that I may revoke my consent and leave the study at any stage, if I wish so, with no negative consequences. I agree that the interview / discussion with me should be audio recorded. I understand that the recording is for research purposes only.

Participant's name: ____

Participant's signature or thumbprint: _____________________ Date: _______________

Witness Name (As appropriate): ____

Witness signature (As appropriate): ____________________ Date: __________________

Study team member's statement: I, the undersigned, have explained to the participant in a language that s/he understands; the procedures to be followed in the study (including audio recording of the discussion), the risks and benefits involved, and the obligations of the study team.

_____________________________ _____________________________ ________________

Name Signature Date

Informed Consent Agreement to audio record focus group discussion with women who do not use injectable contraceptive

Study title: Scale up of community-based injectable contraceptives in Gombe State, Nigeria

SERIAL NUMBER ___________________

Background and purpose: One of the health innovations that have been piloted with significant success around the world, including Nigeria, is the community-based distribution of injectable contraceptives.

Additional Information: In case you have any question or concern about this study please feel free to contact Dr Oluwaseun Akinyemi, Department of Health Policy and Management, College of Medicine, University of Ibadan Ibadan, Nigeria (Tel. No.: +234 803 502 0136; Email: seunakinyemi@hotmail.com)

Informed consent to record: Please read this statement carefully before you sign and if you agree that the interview / discussion with you should be audio recorded, please fill free to sign this form below to affirm your consent.

I, ______________________________ clearly understand the aims of the project titled **"Scale up of community-based injectable contraceptives in Gombe State, Nigeria"** which has been explained to me by the researcher. I have been given the chance to ask questions and I am satisfied with the answers to all of my questions and I agree to participate in the study. I understand that I may revoke my consent and leave the study at any stage, if I wish so, with no negative consequences. I agree that the interview / discussion with me should be audio recorded. I understand that the recording is for research purposes only.

Participant's name: ____

Participant's signature or thumbprint: ____________________ Date: ______________

Witness Name (As appropriate): ____

Witness signature (As appropriate): ___________________ Date: _________________

Study team member's statement: I, the undersigned, have explained to the participant in a language that s/he understands; the procedures to be followed in the study (including audio recording of the discussion), the risks and benefits involved, and the obligations of the study team.

___________________________ ___________________________ _______________

Name Signature Date

Informed Consent Agreement to audio record focus group discussion with married men

Study title: Scale up of community-based injectable contraceptives in Gombe State, Nigeria

SERIAL NUMBER ___________________

Background and purpose: One of the health innovations that have been piloted with significant success around the world, including Nigeria, is the community-based distribution of injectable contraceptives.

Additional Information: In case you have any question or concern about this study please feel free to contact Dr Oluwaseun Akinyemi, Department of Health Policy and Management, College of Medicine, University of Ibadan Ibadan, Nigeria (Tel. No.: +234 803 502 0136; Email: seunakinyemi@hotmail.com)

Informed consent to record: Please read this statement carefully before you sign and if you agree that the interview / discussion with you should be audio recorded, please fill free to sign this form below to affirm your consent.

I, ______________________________ clearly understand the aims of the project titled **"Scale up of community-based injectable contraceptives in Gombe State, Nigeria"** which has been explained to me by the researcher. I have been given the chance to ask questions and I am satisfied with the answers to all of my questions and I agree to participate in the study. I understand that I may revoke my consent and leave the study at any stage, if I wish so, with no negative consequences. I agree that the interview / discussion with me should be audio recorded. I understand that the recording is for research purposes only.

Participant's name: ____

Participant's signature or thumbprint: ____________________ Date: ______________

Witness Name (As appropriate): ____

Witness signature (As appropriate): ___________________ Date: _________________

Study team member's statement: I, the undersigned, have explained to the participant in a language that s/he understands; the procedures to be followed in the study (including audio recording of the discussion), the risks and benefits involved, and the obligations of the study team.

___________________________ ___________________________ _______________

Name Signature Date

Informed Consent Agreement to audio record focus group discussion with single/unmarried men

Study title: Scale up of community-based injectable contraceptives in Gombe State, Nigeria

SERIAL NUMBER ___________________

Background and purpose: One of the health innovations that have been piloted with significant success around the world, including Nigeria, is the community-based distribution of injectable contraceptives.

Additional Information: In case you have any question or concern about this study please feel free to contact Dr Oluwaseun Akinyemi, Department of Health Policy and Management, College of Medicine, University of Ibadan Ibadan, Nigeria (Tel. No.: +234 803 502 0136; Email: seunakinyemi@hotmail.com)

Informed consent to record: Please read this statement carefully before you sign and if you agree that the interview / discussion with you should be audio recorded, please fill free to sign this form below to affirm your consent.

I, ______________________________ clearly understand the aims of the project titled **"Scale up of community-based injectable contraceptives in Gombe State, Nigeria"** which has been explained to me by the researcher. I have been given the chance to ask questions and I am satisfied with the answers to all of my questions and I agree to participate in the study. I understand that I may revoke my consent and leave the study at any stage, if I wish so, with no negative consequences. I agree that the interview / discussion with me should be audio recorded. I understand that the recording is for research purposes only.

Participant's name: ____

Participant's signature or thumbprint: ____________________ Date: ______________

Witness Name (As appropriate): ____

Witness signature (As appropriate): ___________________ Date: _________________

Study team member's statement: I, the undersigned, have explained to the participant in a language that s/he understands; the procedures to be followed in the study (including audio recording of the discussion), the risks and benefits involved, and the obligations of the study team.

___________________________ ___________________________ _______________

Name Signature Date

Distress Protocol

Distress Protocol 1: The protocol for managing distress in the context of a research focus group /interview

(Adapted from: Draucker C B, Martsolf D S and Poole C (2009) Developing Distress Protocols for research on Sensitive Topics. *Archives of Psychiatric Nursing* 23 (5) pp 343-350)

Distress

• A participant indicates they are experiencing a high level of stress or emotional distress

OR

• Exhibit behaviours suggestive that the discussion/interview is too stressful such as uncontrolled crying, shaking etc.

Stage 1 Response

• Stop the discussion/interview.

• The researcher (who is a health professional) will offer immediate support

• Assess mental status:

- Tell me what thoughts you are having?
- Tell me what you are feeling right now?
- Do you feel you are able to go on about your day?
- Do you feel safe?

Review

•If participant feels able to carry on; resume interview/discussion

•If participant is unable to carry on, Go to stage 2

Stage 2 Response

•Remove participant from discussion and accompany to quiet area or discontinue interview

•Encourage the participant to contact their GP or mental health provider

OR

•Offer, with participant consent, for a member of the research team to do so OR

•With participant consent contact a member of the health care team treating them for further advice/support

Follow up

•Follow participant up with courtesy call (if participant consents)

OR

•Encourage the participant to call either if he/she experiences increased distress in the hours/days following the focus group

Distress Protocol 2: The protocol for managing distress in the context of a research focus group /interview management

Adapted from: McCosker,H Barnard, A Gerber, R (2001). Undertaking Sensitive Research: Issues and Strategies for Meeting the Safety Needs of All. Forum: Qualitative Social Research, 2(1)

Pre-data collection

•The researcher would consider the potential physical and psychological impact on the researcher of the participants' description of life experiences.

•The researcher would consider how many interviews could be undertaken in a week.

•The researcher would be aware of the potential for emotional exhaustion.

Data collection stage

• For topics that are potentially sensitive/distressing data collection would be undertaken by two members of the research team.

•Regular scheduled debriefing sessions with the lead researcher.

•Research team would be encouraged to journal their thoughts and feelings which may then become part of fieldwork notes in some research approaches.

Analysis

•Research team would be alerted prior to transcription review of potentially "challenging" or "difficult" interviews

•The research team would have regular scheduled debriefing sessions with the lead researcher

Follow up

•Researcher will access a research mentor (supervisor) if he experiences increased distress in the hours/days following transcription.

Distress Protocol 3: The protocol for managing distress in the context of a research focus group /interview transcription

(Adapted from: Gregory, D Russell, C Phillips, L (1997). Beyond textual perfection: transcribers as vulnerable persons. Qualitative Health Research, 7(2), 294-300.)

Pre-data collection

• The transcriber would be considered in the research proposal, with a clear indication of how s/he will be provided with a "safe" working environment while also maintaining the "quality" of the research.

Ethical review stage

• The transcriber would be included in the ethical clearance process.

• The transcriber would be informed of the nature of the research and the type of data.

Pre-transcription

• The transcriber would be alerted prior to the transcription of potentially "challenging" or "difficult" interviews.

•The transcriber would have regular scheduled debriefing sessions with a named member of the research team.

During Transcription

•Transcriber would have prompt access to an appropriate person for crisis counselling.

•S/he would have a clearly documented termination from the transcription process that includes resolution of personal issues which arose as a consequence of the work.

•Transcriber would be encouraged to journal her/his thoughts and feelings which may then become part of fieldwork notes.

Follow up

•Follow transcriber up with courtesy call (if transcriber consents)

OR

•Encourage the transcriber to call if s/he experiences increased distress in the hours/days following transcription
