# Supplementary material for: ‘Our culture prohibits some things’: qualitative inquiry into how sociocultural context influences the scale-up of community-based injectable contraceptives in Nigeria
Source: BMJ Open. 2020 Jul 19;10(7):e035311. doi: 10.1136/bmjopen-2019-035311 (PMC7371132; doi:10.1136/bmjopen-2019-035311)
Supplement: Author's manuscript [file bmjopen-2019-035311.draft_revisions.pdf]

BMJ Open is committed to open peer review. As part of this commitment we make the peer review history of every article we publish publicly available.

When an article is published we post the peer reviewers' comments and the authors' responses online. We also post the versions of the paper that were used during peer review. These are the versions that the peer review comments apply to.

The versions of the paper that follow are the versions that were submitted during the peer review process. They are not the versions of record or the final published versions. They should not be cited or distributed as the published version of this manuscript.

BMJ Open is an open access journal and the full, final, typeset and author-corrected version of record of the manuscript is available on our site with no access controls, subscription charges or pay-per-view fees (http://bmjopen.bmj.com).

If you have any questions on BMJ Open's open peer review process please email info.bmjopen@bmj.com

BMJ Open

“Our culture prohibits some things”: Qualitative inquiry into how sociocultural context influences the scale up of community-based injectable contraceptives in Nigeria

Journal:	*BMJ Open*
Manuscript ID	bmjopen-2019-035311
Article Type:	Original research
Date Submitted by the Author:	27-Oct-2019
Complete List of Authors:	Akinyemi, Oluwaseun; University of Ibadan College of Medicine, Health Policy and Management; University of the Witwatersrand School of Public Health, Community Health<br>Harris, Bronwyn; Warwick Medical School, University of Warwick, Coventry, Division of Health Sciences, ; University of the Witwatersrand, Centre for Health Policy, School of Public Health<br>Kawonga, Mary; University of the Witwatersrand, School of Public Health
Keywords:	Reproductive medicine < GYNAECOLOGY, HEALTH SERVICES ADMINISTRATION & MANAGEMENT, Health policy < HEALTH SERVICES ADMINISTRATION & MANAGEMENT, PREVENTIVE MEDICINE, PUBLIC HEALTH, QUALITATIVE RESEARCH

SCHOLARONE™
Manuscripts

I, the Submitting Author has the right to grant and does grant on behalf of all authors of the Work (as defined in the below author licence), an exclusive licence and/or a non-exclusive licence for contributions from authors who are: i) UK Crown employees; ii) where BMJ has agreed a CC-BY licence shall apply, and/or iii) in accordance with the terms applicable for US Federal Government officers or employees acting as part of their official duties; on a worldwide, perpetual, irrevocable, royalty-free basis to BMJ Publishing Group Ltd ("BMJ") its licensees and where the relevant Journal is co-owned by BMJ to the co-owners of the Journal, to publish the Work in this journal and any other BMJ products and to exploit all rights, as set out in our licence.

The Submitting Author accepts and understands that any supply made under these terms is made by BMJ to the Submitting Author unless you are acting as an employee on behalf of your employer or a postgraduate student of an affiliated institution which is paying any applicable article publishing charge ("APC") for Open Access articles. Where the Submitting Author wishes to make the Work available on an Open Access basis (and intends to pay the relevant APC), the terms of reuse of such Open Access shall be governed by a Creative Commons licence – details of these licences and which Creative Commons licence will apply to this Work are set out in our licence referred to above.

Other than as permitted in any relevant BMJ Author's Self Archiving Policies, I confirm this Work has not been accepted for publication elsewhere, is not being considered for publication elsewhere and does not duplicate material already published. I confirm all authors consent to publication of this Work and authorise the granting of this licence.

For peer review only

"Our culture prohibits some things": Qualitative inquiry into how sociocultural context influences the scale up of community-based injectable contraceptives in Nigeria

Oluwaseun Akinyemi,[a,d*] (ooakinyemi@comui.edu.ng, seunakinyemi@hotmail.com)

Bronwyn Harris,[b,c] (b.harris.2@warwick.ac.uk)

Mary Kawonga,[d] (mary.kawonga@wits.ac.za)

[a] *Department of Health Policy and Management, College of Medicine, University of Ibadan*

[b] *Centre for Health Policy, School of Public Health, Faculty of Health Sciences, University of the Witwatersrand, Johannesburg*

[c] *Division of Health Sciences, Warwick Medical School, University of Warwick, Coventry*

[d] *Department of Community Health, School of Public Health, Faculty of Health Sciences, University of the Witwatersrand, Johannesburg*

Original research article

*Corresponding author: Dr O.O. Akinyemi
Department of Health Policy and Management
College of Medicine, University of Ibadan
Ibadan, Nigeria
+234 803 502 0136

Word counts:

Abstract: 298

Manuscript: 4133

Running Title:

Sociocultural context and innovation scale up

For peer review only

Abstract

Objectives To explore how sociocultural factors may support or impede the adoption of community-based distribution of injectable contraceptives in Nigeria.

Design A qualitative study based on a grounded theory approach was conducted through in-depth interviews and focus group discussions.

Setting Most participants lived in Gombe State, North East Nigeria. Other participants were from Ibadan (South West) and Abuja (Federal capital territory).

Participants Through seven key informant interviews, 15 in-depth interviews and 10 focus group discussions, 102 participants were involved in the study.

Methods This study, conducted in 2016 was part of a larger study on scale up of community-based distribution of injectable contraceptives. Qualitative data were collected from traditional and religious leaders, health workers and community members. The data were audio recorded, transcribed and analysed using a thematic framework method.

Results Sociocultural challenges to scale up included patriarchy and men's fear of losing control over their spouses, traditional and religious beliefs about fertility, and myths about contraceptives and family planning. As a result of deep-rooted beliefs that children are 'divine blessings' and that procreation should not be regulated, participants described a subtle resistance to uptake of injectable contraceptives. Since Gombe is largely a patriarchal society, male involvement emerged as important to the success of meaningful innovation uptake. Community leaders largely described their participation in the scale up process as active, although they also identified scope for further involvement and recognition.

Conclusion Scale up is more than setting up health sector implementing structures, training health workers and getting innovation supplies, but also requires preparedness which includes paying attention to complex contextual issues. This requires the health system and those who work in it, to move beyond a narrow health 'comfort zone' by actively engaging with, and learning from, those who are leading, caring for and living in, the community.

Strengths and limitations of this study

- The study participants represented a range of stakeholders - users of injectable

For peer review only - http://bmjopen.bmj.com/site/about/guidelines.xhtml

contraceptives, community members, providers and health system managers.

- Our results highlighted that scale up is influenced by several socio-cultural factors; thus, showing the importance of paying attention to complex contextual issues during innovation uptake.
- The findings of our study emphasized how health systems and communities should interact in order to ensure successful scale up of health innovations.
- As with any qualitative study, the findings of this study are not statistically generalizable.

Keywords: Nigeria; Scale up; Community-based distribution of injectable contraceptives; Community participation; Male involvement; Stakeholder collaboration; Sociocultural context

Funding

This research was supported by the Consortium for Advanced Research Training in Africa (CARTA). CARTA is jointly led by the African Population and Health Research Center and the University of the Witwatersrand and funded by the Carnegie Corporation of New York (Grant No--B 8606.R02), Sida (Grant No:54100113), the DELTAS Africa Initiative (Grant No: 107768/Z/15/Z) and Deutscher Akademischer Austauschdienst (DAAD). The DELTAS Africa Initiative is an independent funding scheme of the African Academy of Sciences (AAS)'s Alliance for Accelerating Excellence in Science in Africa (AESA) and supported by the New Partnership for Africa's Development Planning and Coordinating Agency (NEPAD Agency) with funding from the Wellcome Trust (UK) and the UK government. The statements made and views expressed are solely the responsibility of the authors.

Declarations of interest

None.

For peer review only - http://bmjopen.bmj.com/site/about/guidelines.xhtml

1. Introduction

Scale up of effective health innovations, through their increased availability to a larger population over a wider geographic area, is essential for achieving universal health coverage, particularly in developing countries.[1 2] Scale up is influenced by various factors related to the innovation, the users, and the social context in which the innovation is introduced.[3] The nature of the innovation itself, preferences and characteristics of the intended users (including their willingness to adopt the innovation), and ways that the innovation is adapted to the local context are also some of the factors affecting innovation scale up.[4]

The sociocultural context, which includes religious and cultural beliefs, gender and societal norms and attitudes, is an important predictor of adoption of a health innovation in a population.[5 6] The sociocultural context may promote health or be a barrier to health utilization.[7] For example, cultural beliefs and practices have been shown to influence the uptake of a range of health innovations including male circumcision,[8] breast cancer screening[7 9] and contraceptives.[10 11] Without due consideration of the sociocultural context, health innovation development, delivery and scale up is likely to be limited, if not dangerous![12]

Evidence highlights the influence of sociocultural context on the adoption of contraceptive innovations. For example, in a study in rural Poland, Colleran and Mace[13] reported that religious and sociocultural factors, particularly social networks, influence the uptake of contraceptive use more than individual characteristics. Dalaba and colleagues[14] in their study in northern Ghana reported that inadequate male participation led to resistance towards female reproductive freedom. Interventions at societal level, such as adequate male mobilisation coupled with appropriate community-based distribution strategies were shown to be effective in improving contraceptive uptake.[14] In Nigeria, where society is largely patriarchal, with consequences for access to and use of contraceptives, a study found that about two-thirds of rural women who had spousal approval were reported to use contraceptives while all those who were forbidden by their husbands did not use any family planning method.[15] Understanding whether and how the sociocultural context influences scale up of contraceptive innovation is thus important.

Nigeria started its family planning policy review in 2007 and successfully piloted the community-based distribution of injectable contraceptives through community health extension workers (CHEWs) as a new innovation in the health system in 2010 in Gombe

For peer review only - http://bmjopen.bmj.com/site/about/guidelines.xhtml

State, North-eastern region.[16 17] Afterwards, the scale up of this innovation started in 2014, led by a national non-governmental organization and supported by international donor agencies.[18]

According to Bradley *et al,*[3] assessing the receptivity and willingness of community members to adopt a health innovation within their environmental and sociocultural settings is one of the key first steps in the scale up process. In their conception, poor understanding by implementers of potential users' sociocultural context will limit scale up efforts, while good understanding will help program managers to recognise contextual factors that may enhance or inhibit the uptake of an innovation.[3] However, the understanding of how sociocultural contextual factors may facilitate or impede the successful scale up of the contraceptive innovation beyond the initial pilot site in Nigeria is limited. This study therefore assesses how sociocultural factors may influence the scale up of the community-based distribution of injectable contraceptives in Nigeria.

2. Methods

2.1 Study design and setting

This cross-sectional study conducted from September to November 2016, part of a larger research project on scale up of community-based distribution of injectable contraceptives, took place in Gombe (North eastern Nigeria), one of 36 States in Nigeria. Gombe State, with a population of 2,353,879 people according to the 2006 population census,[19] has one of the lowest contraceptive prevalence rates in the country (3.5% for modern methods and 4.0% for any method) and one of the highest maternal mortality rates (1,726/100,000).[16 20 21] Gombe was purposively selected because it was the setting for the national pilot test for community-based distribution of injectable contraceptives in 2010. Gombe is divided administratively into 11 Local Government Areas (LGAs). The study held in two LGAs – Gombe (A) and Yamaltu/Deba (B). Although participants were primarily based in Gombe State, one interview took place in Ibadan (South west) and 3 interviews in Abuja (Federal Capital Territory).

Participants and sample

Qualitative data were collected from a range of stakeholders, including traditional and religious leaders, health workers as well as community members, through seven key informant interviews, 15 in-depth interviews and 10 focus group discussions (see Table 1). In

For peer review only - http://bmjopen.bmj.com/site/about/guidelines.xhtml

total, 102 people participated in this study. The participants were selected through a purposive sampling method, where study respondents are recruited based on characteristics of interest, availability, and ability to provide relevant information to the research question:[22 23] how may sociocultural context influence the process of introducing, translating and integrating health innovation? The number of interviews and FGDs was determined by the principle of saturation in qualitative study, a point where no new data is being gotten.[24]

This study adopted the grounded theory approach using inductive reasoning. This approach is appropriate for this study since it is exploratory in nature and principles are being derived from specific observations.[24 25] Of the three authors, two are women, two are physician-researchers and two are domiciled in Africa.

[Table 1]

2.2 Data collection

Interview guide and focus group guide were used to conduct the interviews and focus group discussions, respectively (see Supplementary file 1). The interview guides for the discussions with community members and interview with traditional leaders were translated to the predominant local language - Hausa. The main issues covered in the interview and discussions included community perceptions about contraceptives and specifically about the innovation (community-based delivery of contraceptives), their role in the scale up process, as well as their views regarding the sociocultural challenges and facilitators of the scale up process and community engagement regarding the scale up. Interviews and discussions were tape-recorded and later translated and transcribed in full.

2.3 Data analysis

Transcripts were analyzed iteratively with the aid of the NVIVO (version 10) software using the thematic framework approach.[24 25] The pre-defined thematic areas included features of the sociocultural context that may influence scale up of the innovation. These were: community members' perceptions regarding community-based distribution of injectable contraceptives, community members' acceptance (or rejection) of contraceptives, and community engagement in the scale up process. Emerging themes were also considered and were indexed

For peer review only - http://bmjopen.bmj.com/site/about/guidelines.xhtml

and compared with themes from subsequent interviews until saturation was attained. Interviews were stopped at the point of saturation, where no new information was obtained.[24] The authors reviewed the transcripts and compared themes, before these were applied to subsequent coding of all the transcripts. The SRQR reporting guidelines for qualitative studies[26] was used in preparing this manuscript.

2.4 Ethics approval and consent to participate

Ethical approval for this study was obtained from the University of Ibadan/University College Hospital Ethical Review Board (Reference No.: UI/EC/16/0022) as well as the Human Research Ethics Committee (Medical) of the University of the Witwatersrand (Reference No.: M160737). Written informed consent were obtained from study participants before the interviews and FGDs (see Supplementary file 2). Participants were also assured of confidentiality by ensuring that all identifiers were removed from the data and that only the research team has access to the data.

3. Results

Respondents described a number of sociocultural factors that challenge the scale up process encountered right from the pilot stage (see Figure 1).

[Figure 1]

3.1 Deep rooted cultural and religious beliefs about fertility

Participants spoke about a deep-rooted belief amongst community members that children are ‘divine blessings’ and that procreation should not be regulated. Emerging from this world view, participants described a subtle resistance to the uptake of injectable contraceptives, more passive than overt in its expression by individuals and families who would feign acceptance but refuse the intervention when given.

“*…there will always be resistance to this use of contraceptives, because here, we are*

For peer review only - http://bmjopen.bmj.com/site/about/guidelines.xhtml

people that believe that children are a blessing, so you give birth to as many as possible. …The resistance may not be as a group, but when you come to individual levels… as a government, they might not show you the resistance, but when it comes to the actual usage, that's where you start having problems." (IDI, male, Doctor, SMoH)

Also, there were issues about religious beliefs influencing perception about family planning/ contraceptive use at the community level. Religiosity, that is, strong adherence to religious beliefs, was said to be one of the major factors impeding the uptake of contraceptives in general.

"*…when you look at northern Nigeria, majority are Muslims and when you look at the south eastern states, you know we have issue of religion, issue of uptake of all contraceptives, not even injectables alone. To the south eastern man, most of them are Catholics, they frown at family planning, so also here (northern Nigeria) people frown at family planning in general*" (KII, male, Senior official, SMoH).

3.2 Awareness and perceptions regarding family planning

Most interview respondents agreed that there was a need to continue to educate community members about the overall benefits of contraceptives and family planning as well as emphasize possible side effects of contraceptives use among women. Also, informants were of the opinion that lack of knowledge about benefits of contraception contributes to reluctance to adopt the innovation. In the words of a respondent:

"*…the community and people at the grassroots have little information (on contraceptives)*" (KII, female, Senior official, SMoH).

These challenges were further compounded by reports of adverse effects of the contraception from some women especially "*severe bleeding during menstruation*" (IDI, female, CHEW). Healthcare providers felt that to effectively deal with these challenges, it was important to listen sympathetically to the women and treat reported side effects, in line with the training received for the innovation scale up. Education was also given when clients had questions in order to resolve doubts about the innovation. For example, a respondent said:

"*We listen to them, and we explain to them that since it (contraceptive) is a new thing in the body, they must experience such things…*" (IDI, female, Nurse).

For peer review only - http://bmjopen.bmj.com/site/about/guidelines.xhtml
For peer review only

Focus group discussants highlighted many reasons why women in their reproductive age group used injectable contraceptives, including desire to stay attractive in order to prevent husbands from 'straying', space the children and enjoy life with fewer financial and parenting constraints. One of the discussants who currently uses injectable contraceptives described her motivation thus:

> "*...If we space our children well, we will enjoy our lives because our husbands will always desire us since we are always looking attractive. He will not stay out late because he will be thinking of you at home*" (FGD, female, current user).

Also, a participant stated that the impetus for contraceptive use was the fact that spacing her children afforded her the ability to take care of herself and the home thereby promoting hygiene and healthy living in the home. She said:

> "*…You will be able to take care of yourself as well as your house, your husband will also desire you as you will look good as well as your children. Flies will not follow you around and anyone that comes to your house will be able to eat and drink because everywhere is clean and attractive"* (FGD, female, current user).

3.3 Perceptions regarding the community-based distribution (CBD) approach

Many of the women who were current users of injectable contraceptives stated their preference for the community-based approach over the facility-based system of distributing the contraceptives. Some of their reasons for this position included convenience, affordability (saves cost as transport fare is not needed), improved privacy and focused attention from the health worker.

> "*…During home visit, it's between you and the woman (health worker) and she can explain it very well to you since she is not in a hurry; although in the hospital they can also explain but the home visit is better since the health worker will explain until you understand.*" (FGD, female, current user).

Likewise, some male discussants preferred the community-based approach because they believed it was more confidential and gave women more time to deal with domestic issues. The CBD approach was also believed to align with men's desire to be in control of 'family issues'.

> "*...Maybe the time the wife wants to go (to the health facility) the husband needed her*

For peer review only - http://bmjopen.bmj.com/site/about/guidelines.xhtml

> *for something else…Sometimes some husbands want all these things to be confidential, but when you now tell them to release their wives to the hospital, they will think you are exposing the family issues*" (FGD, married men).

However, one of the discussants who favoured the facility-based approach *'prefer[ed] to go to the hospital, so I can be checked properly, including to check my BP*" (FGD, female, current user). Similarly, some married men also preferred the facility-based approach as they believed that the community-based method is intrusive of their privacy and undermines their authority:

> *"…My advice is that the woman should come to hospital …because some husbands will query you that who gave you the permission to see their wives (at home) and advise them about child spacing. Some disagree with the community-based method*" (FGD, married men).

3.4 Spousal involvement in family planning

In addition, male involvement was highlighted as important if uptake of innovation will be meaningful since Gombe is largely a society where the women need the men's permission for doing many things including obtaining injectable contraceptives.

> "*Some men will not accept it (contraceptive) because they want plenty children…she (the wife) should continue begging the husband until he agrees if she wants to use contraceptives*" (FGD, women not using contraceptives).

Furthermore, cultural beliefs and sociocultural context were reported as fuelling poor awareness and knowledge about contraceptive use. Also, male involvement was described as important in dealing with the problem of ignorance and poor perception about family planning in the community. According to the participants in this study, once the men are well informed, it is easier to get their buy-in and cooperation in permitting their wives to get the injections.

> "*…some of the men don't know much about family planning commodities and some (people) are telling them some of the side effects. So, they prevent the women from using them…that's why we need more sensitization to the men and the youths*" (IDI, female, Nurse).

> *"The perception of men (about contraceptives) is lower than that of women. The*

For peer review only - http://bmjopen.bmj.com/site/about/guidelines.xhtml

> *women know the implications better than the men. The men in the rural areas don't mind having any number of children...*" (FGD, married men)

Also, a female health worker emphasised the importance of male involvement particularly in communities where the literacy rate is low:

> *"Honestly. we need to do more in carrying them [men] along. Male involvement is very important because you know they are our superiors. We in the North, it is only the educated that may be sometimes at loggerhead (with their husbands)… but these illiterates and native people, it is always the man that is superior. So, it is very important for us to consider male involvement, it will help a lot* (IDI, female, Nurse).

3.5 Community support for scale up

The traditional leaders interviewed believed that community support for the innovation is very high.

> "*In fact, the support our community gives to this programme, without exageration, I will say maybe 80-90%. They (the community) have been very supportive of the programme*" (IDI, male, Traditional ruler, Yemaltu-Deba).

> "*Definitely, you have 70% support from the communities. From the religious leaders, traditional rulers and even of ward heads in the community we get 100% support"* (IDI, male, Traditional leader, Gombe).

However, some cautioned that the community support is not as high as expected due to what they call low knowledge and awareness about contraceptive choices in Northern Nigeria.

> "…*Really, people have supported the program but I know some people do not have the knowledge (about injectable contraceptives) but those that have the knowledge are really in support of the program…* [you know] *the Northern culture prohibits some things, that's why I said the support for it (injectable contraceptives scale up) is not as much as we expect.*" (IDI, male, Christian religious leader)

One community leader described his role as active in the scale up process because he helps in disseminating information to all the wards under his domain. He shared his experience:

> "*Whenever the need arises, we normally call the kingmakers, district head kingmakers, they are about seven, and we have at least 54 ward heads, we use to call*

For peer review only - http://bmjopen.bmj.com/site/about/guidelines.xhtml

them from time to time and enlighten them on the programme. In fact, almost all the community leaders are fully aware of the programme" (IDI, male, Traditional leader).

Many of the leaders ascribed the progress with the scale up in their communities to the involvement of the traditional leaders in the programme. However, some leaders complained of not being officially involved in the programme, although they still offer their support.

"*There are so many traditional rulers that were involved in this program; that is the reason why it has been accepted 100%, especially in Gombe State now*" (IDI, male, Traditional leader, Gombe).

"*…My involvement is…passive involvement. Of course, we preachers, you can push people, encourage them in your own little way... We preach it, we tell people about it*… (IDI, male, Christian religious leader)

4. Discussion

In our study, there were a number of sociocultural issues alluded to as factors that may affect scale up of a contraceptive innovation in Nigeria, including patriarchy, traditional and religious norms, beliefs and myths about contraceptives and family planning. Agunbiade & Ogunleye[27] reported in their study assessing the sociocultural barriers to the scale up of exclusive breastfeeding in Nigeria, the scale up process cannot be separated from the context and the culture in the environment. Further, in a study among African American women, culture and health beliefs were found to be significant predictors of health innovation uptake.[28]

Our study indicates it is vital to secure the understanding and support of the men and significant others for scale up of the community-based distribution of injectable contraceptives to succeed in environments like our study site that are still largely patriarchal.[27] Similar to the findings of our study, male involvement and support of women's significant others, particularly the mothers-in-law, have been reported to be crucial to innovation scale up in Nigeria as these significant others have strong influence on choices made by women.[27] In other settings, increased male participation through increasing men's knowledge and reducing their opposition has been shown to improve uptake and continued use of contraceptives[29-31] and enhancement of prevention of mother to child transmission of HIV (PMTCT) scale up processes.[32] In this study, one of the reasons why women reportedly

For peer review only - http://bmjopen.bmj.com/site/about/guidelines.xhtml

use contraceptives was to look attractive and satisfy their husbands in order to prevent them from engaging in extramarital relationships. Thus, contraception is both liberating for women on one hand but also perhaps serving the very patriarchy they are trapped in on the other. Studies have shown that in many African societies, patriarchy is perpetuated by the socialisation process which starts from the family and is often preserved by women themselves.[33 34]

Also, findings from our study show passive resistance to contraceptive uptake, particularly at the individual levels. Research has shown that passive resistance is one of the major reasons for the high failure rate of innovation scale up.[35 36] Passive resistance often results from individuals' resistance to change and contentment with or lack of power to change the status quo.[36] Further, consumers will not adopt an innovation if they are not sure of its value or benefits.[37] According to Heidenreich and Kraemer, passive innovation resistance should be addressed before the introduction of new innovations into the society.[35] Strategies for reducing passive resistance to uptake of health innovations in the community include actively involving men in their uptake,[14] as well as giving adequate and correct information about the advantages of contraceptive use to all stakeholders - health workers, community and religious leaders, trained volunteers and program managers.[37]

Since the passive resistance reported in this study is largely underpinned by deep-seated issues of patriarchy, religious beliefs and traditional norms/values, it is imperative for policy makers and program managers to engage men – who are custodians, in most cases, of both religious and cultural values.[38 39] Male involvement can bring benefits for all community stakeholders,[38] as well as social change.[40] With careful management and ongoing monitoring, the involvement of men may ultimately give the scale up of this health innovation a greater chance of success.

Furthermore, religious beliefs were also found to influence scale up of the community-based injectable contraceptives and family planning in general in our study sites in Northern Nigeria. Religion has been shown to significantly influence decisions on contraceptive use,[41] as well as individual/household choices, lifestyle and even traditional values.[42] Thus, religion has great implications for the social marketing of commodities particularly contraceptives, as uptake of health products has been shown to differ significantly among adherents of major religions.[42] For example, research done in Islamic countries[43 44] highlights the need to understand Islamic religion in order to do effective social marketing, and

For peer review only - http://bmjopen.bmj.com/site/about/guidelines.xhtml

recommends that approval be obtained from religious leadership before certain sensitive products like contraceptives are marketed. Understanding religious nuances in local contexts is important in multi-faith countries such as Nigeria – for example, what 'works' in the largely-Islamic North[45] may need adaptation for Christian populations elsewhere.

Our study demonstrated the importance of strong community support and partnership with community gate-keepers in the scale up process. Research has shown that the CBD approach cannot succeed without community participation.[46] Therefore, programme implementers need to learn from the community what works (or may work) in the setting, what distribution method the community prefers or what modification to the existing methods they favour. Research has shown that there is no quick fix to scaling up.[46] It is essentially a learning process which is unique in different contexts. Therefore, scale up may not be effective without adequate contextual knowledge and willingness to learn through the process of community participation and stakeholder collaboration.[46 47]

While the focus of this paper has been on challenges as identified in the wider context of religion and patriarchy, many of the suggested solutions rely on the health systems and those working in it, to navigate these challenges and overcome them. However, this cannot be a static or reactive approach but rather needs to recognise that health systems and those working in them are also part of the sociocultural context and therefore it is important to find ways to support healthcare workers in mediating, translating and promoting the innovation in this context.

4.1 Limitations of the study

Some limitations of this study should be emphasised. As with any qualitative study, the findings are not statistically generalizable but the theories and ideas can be applied to other contexts. Thus, the findings of this study provide valuable information into how sociocultural context and user support affect the scale up of health innovations in LMICs, particularly in a patriarchal society like Nigeria.

4.2 Recommendations

Further studies are needed on the barriers and facilitators to community-based distribution of health innovations.[41] Although the pilot project[16] provided some evidence that community-based distribution is feasible in Nigeria, more understanding about what may impede or enhance this approach to health commodity distribution is needed. In addition, incorporating

For peer review only - http://bmjopen.bmj.com/site/about/guidelines.xhtml

the CBD approach with other outreach programmes, developing a more effective supervision of the approach may help make CBD more productive.

4.3 Conclusion

Our study revealed a number of sociocultural factors influencing the scale up of community-based distribution of injectable contraceptives in Nigeria. These include subtle resistance to innovation uptake as a result of deep-rooted cultural and religious beliefs about fertility and contraceptive use, influence of patriarchy and prominent role of traditional and religious leaders on innovation uptake. Findings also suggest the need for continuous community education and engagement with the significant others and community leadership structures – both traditional and religious. Thus, providing an innovation does not necessarily translate to uptake. Therefore, program implementers should also consider sociocultural and health system contexts when packaging a health innovation. Hence, supporting those in the health system to mediate the innovation in ways that are sensitive to the context but which, by working with communities and others, promotes transformation is imperative. Policy implementers should also see scale up as a learning process and be willing to move at the speed of the community.

Acknowledgements

We are grateful to Drs Mariya Saleh, Wole Adefalu, Edward Oladele and Hadiza Kamofu for expediting access to key informants. Also, we appreciate Dr Alex Eze for his feedback on the early draft of the manuscript. We are grateful for the support of Dr Busola Adebayo, Jimi Latunji and Wunmi Folajimi-Senjobi with editing the manuscript. We would like to thank all the participants in this study.

Authors' contributions

OA conceived the study; OA and MK designed it. The data was collected by OA. OA, MK and BH drafted and commented on the draft of the manuscript. OA, MK and BH read and approved the final version of the manuscript.

Data Availability Statement

Dataset for this study is available at https://doi.org/10.5061/dryad.8kprr4xj4.

For peer review only

References

1. Subramanian S, Naimoli J, Matsubayashi T, et al. Do we have the right models for scaling up health services to achieve the Millennium Development Goals? *BMC health services research* 2011;11(1):336.
2. Manham L, Hanson K. Scaling up in international health: what are the key issues? Review. *Health Policy Plan* 2010;25:85-96.
3. Bradley EH, Curry LA, Taylor LA, et al. A model for scale up of family health innovations in low-income and middle-income settings: a mixed methods study. *BMJ open* 2012;2(4):e000987.
4. Hecht R, Stover J, Bollinger L, et al. Financing of HIV/AIDS programme scale-up in low-income and middle-income countries, 2009–31. *The Lancet* 2010;376(9748):1254-60.
5. Johns B, Torres TT. Costs of scaling up health interventions: a systematic review. *Health policy and planning* 2005;20(1):1-13.
6. Mangham LJ, Hanson K. Scaling up in international health: what are the key issues? *Health policy and planning* 2010;25(2):85-96.
7. Rajaram SS, Rashidi A. Asian-Islamic women and breast cancer screening: a socio-cultural analysis. *Women & health* 1999;28(3):45-58.
8. Westercamp N, Bailey RC. Acceptability of Male Circumcision for Prevention of HIV/AIDS in Sub-Saharan Africa: A Review. *AIDS and Behavior* 2007;11(3):341-55. doi: 10.1007/s10461-006-9169-4
9. Katapodi MC, Lee KA, Facione NC, et al. Predictors of perceived breast cancer risk and the relation between perceived risk and breast cancer screening: a meta-analytic review. *Preventive medicine* 2004;38(4):388-402.
10. Muanda M, Ndongo PG, Taub LD, et al. Barriers to modern contraceptive use in Kinshasa, DRC. *PloS one* 2016;11(12):e0167560.
11. Muanda MF, Ndongo GP, Messina LJ, et al. Barriers to modern contraceptive use in rural areas in DRC. *Culture, health & sexuality* 2017;19(9):1011-23.
12. Airhihenbuwa CO. Health and culture: Beyond the Western paradigm: Sage 1995.
13. Colleran H, Mace R. Social network-and community-level influences on contraceptive use: evidence from rural Poland. *Proc R Soc B* 2015;282(1807):20150398.
14. Dalaba MA, Stone AE, Krumholz AR, et al. A qualitative analysis of the effect of a community-based primary health care programme on reproductive preferences and contraceptive use among the Kassena-Nankana of northern Ghana. *BMC health services research* 2016;16(1):80.
15. Etukudo IW. Spousal approval, communication and contraceptive behaviour in rural Nigeria. *African Journal of Midwifery and Women's Health* 2015;9(4):170-76.
16. FHI. The Effectiveness of Community-Based Access to Injectable Contraceptives in Nigeria: A Technical Report. 2010
17. Association for Reproductive and Family Health. Increasing access to contraceptives in Nigeria through task-shifting to Community Health Extension Workers (CHEWs) Abuja: Association for Reproductive and Family Health; 2015 [cited 2015 6 January]. Available from: http://www.arfh-ng.org/news/item012.php accessed 6 January 2015.
18. Association for Reproductive and Family Health. Increasing Access to Contraceptives in Nigeria Through Task-shifting to Community Health Extension Workers (CHEWS) Ibadan: Association for Reproductive and Family Health,; 2014 [cited 2015 5 November]. Available from: http://arfh-ng.org/increasing-access-contraceptives-nigeria-task-shifting-community-health-extension-workers-chews/ accessed 5 November 2015.
19. National Population Commission. Population and housing census of the Federal Republic

For peer review only - http://bmjopen.bmj.com/site/about/guidelines.xhtml

of Nigeria. *Priority tables* 2006;1
20. National Population Commission. Nigeria demographic and health survey 2008. *Nigeria and ICF Macro* 2008
21. National Population Commission, ICF International. Nigeria Demographic and Health Survey 2013. *Abuja, Nigeria* 2014
22. Vonk ME, Tripodi T, Epstein I. Research techniques for clinical social workers: Columbia University Press 2007.
23. Pope C, Mays N. Qualitative research: reaching the parts other methods cannot reach: an introduction to qualitative methods in health and health services research. *Bmj* 1995;311(6996):42-45.
24. Temple B, Edwards R. Interpreters/translators and cross-cultural research: Reflexivity and border crossing. *International Journal of Qualitative Methods* 2002;1(2)
25. Hutchison AJ, Johnston LH, Breckon JD. Using QSR-NVivo to facilitate the development of a grounded theory project: an account of a worked example. *International journal of social research methodology* 2010;13(4):283-302.
26. O'Brien BC, Harris IB, Beckman TJ, et al. Standards for reporting qualitative research: a synthesis of recommendations. *Academic Medicine* 2014;89(9):1245-51.
27. Agunbiade OM, Ogunleye OV. Constraints to exclusive breastfeeding practice among breastfeeding mothers in Southwest Nigeria: implications for scaling up. *International breastfeeding journal* 2012;7(1):5.
28. Sociocultural context of mammography screening use. Oncology nursing forum; 2006. Oncology Nursing Society.
29. Kabagenyi A, Jennings L, Reid A, et al. Barriers to male involvement in contraceptive uptake and reproductive health services: a qualitative study of men and women's perceptions in two rural districts in Uganda. *Reproductive health* 2014;11(1):21.
30. Ujuju C, Anyanti J, Adebayo S, et al. Religion, culture and male involvement in the use of the Standard Days Method: evidence from Enugu and Katsina states of Nigeria. *International nursing review* 2011;58(4):484-90.
31. Sanusi AA, Akinyemi OO, Onoviran OO. Do knowledge and cultural perceptions of modern female contraceptives predict male involvement in Ayete, Nigeria? *African journal of reproductive health* 2014;18(4):105-14.
32. Peltzer K, Jones D, Weiss SM, et al. Promoting male involvement to improve PMTCT uptake and reduce antenatal HIV infection: a cluster randomized controlled trial protocol. *BMC Public Health* 2011;11(1):778. doi: 10.1186/1471-2458-11-778
33. Florence N. Bukusu (Kenya) folktales: how women perpetuate patriarchy. *International Feminist Journal of Politics* 2013;15(3):370-90.
34. Kambarami M. Femininity, sexuality and culture: Patriarchy and female subordination in Zimbabwe. *South Africa: ARSRC* 2006
35. Heidenreich S, Kraemer T. Innovations—doomed to fail? Investigating strategies to overcome passive innovation resistance. *Journal of Product Innovation Management* 2016;33(3):277-97.
36. Heidenreich S, Spieth P. Why innovations fail—The case of passive and active innovation resistance. *International Journal of Innovation Management* 2013;17(05):1350021.
37. KISS Communications. Why Consumers Resist Innovation – part one 2016 [cited 2018 1 September]. Available from: https://www.kisscom.co.uk/news/why-consumers-resist-innovation-part-one accessed 1 September 2018.
38. Orji E, Onwudiegwu U. Contraceptive practice among married market men in Nigeria. *East African medical journal* 2003;80(7):357-60.
39. Orji E, Onwudiegwu U. Contraceptive practice among married men in Nigeria. *East Afr*

For peer review only - http://bmjopen.bmj.com/site/about/guidelines.xhtml

Med J 2008;200(80):7.
40. Onwukwe LC, Njemanze VC, Ebeh R, et al. Religiosity and Sexism as Indices of People's Perception of Women in Politics in Nigeria. *American Journal of Social Science Research* 2017;3(2):9-16.
41. Mitike G. Community-based distribution of family planning as perceived by people in the reproductive age group, North and South Gondar zones, Ethiopia. *Ethiopian Journal of Health Development* 2000;14(1):31-42.
42. Shyan Fam K, Waller DS, Zafer Erdogan B. The influence of religion on attitudes towards the advertising of controversial products. *European Journal of marketing* 2004;38(5/6):537-55.
43. Michell P, Al-Mossawi M. Religious commitment related to message contentiousness. *International Journal of Advertising* 1999;18(4):427-43.
44. Luqmani M, Yavas U, Quraeshi Z. Advertising in Saudi Arabia: content and regulation. *International Marketing Review* 1989;6(1)
45. Watts MJ. Silent violence: Food, famine, and peasantry in northern Nigeria: University of Georgia Press 2013.
46. Gilson L, Schneider H. Commentary: Managing scaling up: what are the key issues? *Health Policy and Planning* 2010;25(2):97-98.
47. Phillips JF, Greene WL. Community based distribution of family planning in Africa: lessons from operations research. 1993

For peer review only - http://bmjopen.bmj.com/site/about/guidelines.xhtml

Table and Figure

Table 1: Participants and data collection methods across study sites

Location	Type	n	Participant type
**Federal and State Level**			
	KII	4	NGO program manager, Senior official at the Federal MoH[a], NGO program managers
**Gombe State**			
	KII	3	Senior officials of State MoH.
	IDI	7	Health workers (Nurses, CHEWs[b])
LGA **A**			
	FGD	5	Current contraceptive users, older women non-users, younger women non-users (in reproductive age group), married and single men
LGA **B**	IDI	8	Health workers (Nurses, CHEWs[b])
	FGD	5	Current contraceptive users, older women non-users, younger women non-users (in reproductive age group), married and single men
**Total**		**32**	

[a]MoH – Ministry of Health; [b]CHEWs – Community Health Extension Workers

For peer review only

For peer review only - http://bmjopen.bmj.com/site/about/guidelines.xhtml

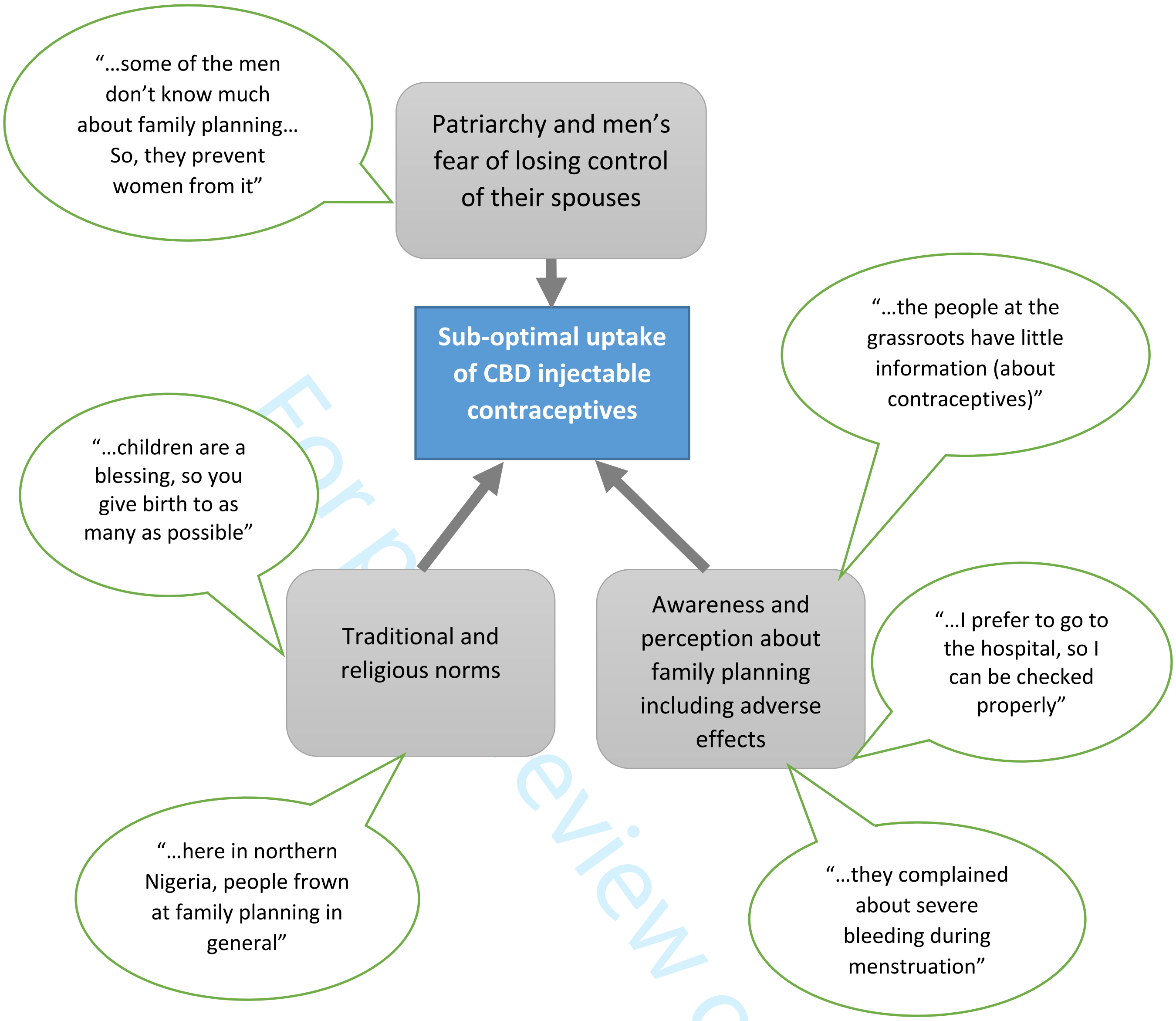

Figure 1: Socio-cultural factors influencing uptake of CBD of injectable contraceptives in Gombe State

For peer review only - http://bmjopen.bmj.com/site/about/guidelines.xhtml

APPENDIX I: Interview Guide: Key informant interview with senior MOH officials and NGO programme managers

1. Let's start by you describing your role in implementing this intervention – the community-based delivery of injectable contraceptives in the pilot sites in Gombe. What was your role and how long were you involved?

2. Did you encounter any challenges during the pilot study? Please explain.
   Probes:
   - If not mentioned, ask: any resistance from the community members, from health workers, from other stakeholders, from potential users themselves?
   - How did you handle challenges / resistance?

3. We are interested in your experience with the process of taking the intervention from pilot study phase to wider implementation in Gombe itself. What was the process, from implementation to wider scale up of the intervention? Walk me through the process.
   Probes:
   - The goal, approach to implementing, key components of the process, indicators of success
   - Landscape and stakeholder assessment done – before, during or after the pilot?
   - Your role in these processes?

4. In your opinion, what factors have supported the scale up of this intervention so far?

5. Have you experienced challenges during the process of scaling up? Please explain.
   Probes:
   - What challenges? ... ***may prompt with*** "any others"
   - How did you handle challenges?

6. Was there a way of ensuring that the intervention aligns to the wants and preferences of the users you were targeting? Please tell me about it.
   Probes:
   - Any user needs / wants assessment done? By whom? When?
   - Any modifications made in order to align packaging of the innovation to user wants – either during or after the pilot?
   - Any health service modification (e.g. change service models for delivering the intervention)?

7. Have communities been engaged in the scale up process? Please describe in what ways and by whom?

8. Looking back, is there anything that you think could have been done differently to facilitate the process from pilot study to wider implementation? Please explain.

9. Is there anything else we have not asked but you would like to tell us about your experience with the process of implementation and scale-up of CBD?

Thank you for your time. This is the end of our discussion.

For peer review only

APPENDIX II: In-depth Interview Guides

A. Interview guide: In-depth interview with health workers (Doctors and CHEWs)

1. Let's start by you describing your role in implementing this intervention – the community-based delivery of injectable contraceptives by community health extension workers.
    a. What role did doctors / CHEWs play in designing the intervention?
    b. What about their role in introducing the intervention at community level?
    c. What was your own role?
    Probes:
    - **Doctors:** Your own (or experience of other doctors like you) with: planning, training CHEWs, providing supportive supervision to CHEWs during and after the pilot?
    - **CHEWs**: Your own (or experience of other CHEWs like you) in planning, administering the injectable contraceptives to women?

    d. Did you / other health workers experience challenges?
        i. Please explain.
        ii. How did you handle the challenges?

2. In your opinion, what factors affect wider implementation and uptake of the CBD of injectable contraceptives in the communities that you serve?
    Probes:
    - Anything else?

3. In your opinion, how supportive of this innovation are:
    a. **For doctors**: doctors in general, and your professional health group (e.g. Nigerian Medical Association) please explain.
    b. **For CHEWs**: community health extension workers in general? Please explain.

4. As far as you are aware, what role, if any, have health workers like you played in making the CBD of injectable contraceptives more acceptable to the users in your community? Please explain.

5. In your opinion, how likely are those women who have already adopted the CBD of injectables to spread information about the innovation to other women who are not using it? Please explain.

6. Looking back, is there anything that you feel might have been done differently to facilitate the process of moving from pilot study to wider implementation?

7. Is there anything else we have not discussed that you would like to tell us about your experience with implementing the CBD of injectable contraceptives?

Thank you for your time. This is the end of our discussion.

For peer review only

B. Interview guide: In-depth interview with community leaders

1. You were selected to participate in this study because you are aware of the government program in which CHEWs provide injectable contraceptives to women in the community, outside the health clinic setting.
    a. Please describe what you know about this program.
    b. How did you hear about it?

2. How much support do you think there is for this program in your community? Please explain

3. Have you or other community leaders like you been involved in introducing this program on injectable contraceptives into your community?
    a. ***If yes***: Describe your / or other leaders' involvement (what role)? How did you / they get involved? Who initiated?
    b. How do you feel about being involved / not being involved?

4. The government started with this program only in two places in Gombe but they are now trying to make this available to all other parts of Gombe.
    a. What challenges do you think the government might encounter when trying to get wider uptake of this programme in the community?
    b. Have you or other community leaders in this community been approached by government to assist with addressing these challenges?

5. In your opinion, what might prevent women in your community from taking up this intervention? What may encourage them to take it up?

6. In your opinion, how likely are those women who have already adopted the CBD of injectables to share information about it to other women who are not using it? Please explain?

7. Looking back, is there anything that you feel might have been done differently to make this way of providing contraceptives acceptable in your community?

8. Is there anything else we have not discussed that you would like to tell us about your experience with implementing the CBD of injectable contraceptives?

Thank you for your time. This is the end of our discussion.

For peer review only

APPENDIX III: Focus Group Discussion Guides

A. Focus group discussion guide: FGDs with women who have already adopted the use of injectable contraceptives through the CBD approach

1. Let us start with talking about contraceptives / family planning methods in general. Please share with me what you understand about contraception / family planning. What kinds of contraceptive methods do you know?

2. What do you think people in this community think about contraceptives and family planning in general?

   Probe:
   - Do they support the idea of contraception / family planning in general?
   - Specifically, how supportive of injectable contraceptives compared to others?

3. What about women in this community. How do you think they feel about contraceptives and family planning in general?

   Probe:
   - Support the use of contraception / family planning in general?
   - Specifically, how popular are injectable contraceptives compared to others?
   - What makes women use injectable contraceptives? What makes them not use it?

4. I am interested to know what you think women want and prefer regarding contraceptives. Which types of contraceptives do women in your community prefer? How do you think women prefer to receive contraceptives (from clinic versus other places; from doctor or nurse versus others)?

5. The government introduced a new program where community health extension workers now administer injectable contraceptives to women away from the clinic, including at homes. I know that you already receive injectable contraceptives through this approach.

   a. How did you hear about this program?

   b. What made women like you to decide to receive injectables through CHEWs?

   c. Would you encourage friends or family members to receive injectable contraceptives from CHEWs? Please explain.

   d. What do you think might prevent other women in your community from using this new way of receiving injectable contraceptives through CHEWs?

6. What suggestions do you have for how the way injectable contraceptives are administered in your community can be improved to make sure many more women receive them?

Thank you for your time. This is the end of our discussion

For peer review only

B. Focus group discussion guide: FGD with a group of women in the reproductive age group who have not yet adopted the CBD of injectables (may include those who do not use injectables and those who do)

1. Let us start with talking about contraceptives / family planning methods in general. How many contraceptive types do you know? Can you describe them?

2. How do you think people in this community perceive contraceptives and family planning in general?
   Probe:
   - Popularity of contraceptives / the idea of family planning in general
   - Specifically, popularity of injectable contraceptives

3. How do you think women in this community perceive contraceptives and family planning in general?
   Probe:
   - Popularity of contraceptives / family planning in general
   - Specifically, popularity of injectable contraceptives

4. What do women want - which types of contraceptives, which mode of delivery do they prefer? (clinic vs. other places, doctor/nurse versus others)

5. Let us now talk about injectable contraceptives specifically. In your opinion, what factors will make women of reproductive age use or reject injectable contraceptives? What makes them not use it?

6. The government introduced a new program where community health extension workers now administer injectable contraceptives to women away from the clinic, including at homes. I know that you have not received injectable contraceptives through this approach.
   a. How did you hear about this program?
   b. What factors make women like you decide not to receive injectables through CHEWs?
   c. What factors would encourage you to receive the injectable contraceptives through CHEWs?
   d. Willingness to encourage friends and family members of reproductive age to receive injectable contraceptives from CHEWs?
   e. What do you think might prevent other women in your community from using this new way of receiving injectable contraceptives through CHEWs?

   Probes:
   - Resistance to mode of distribution – facility vs. community based
   - Socio-cultural resistance to injectable contraceptives
   - Resistance to injectable contraceptives being administered by CHEWs rather than other health workers like nurses and doctors

7. What suggestions do you have for how the way injectable contraceptives are administered in your community can be improved to make sure many more women like you receive them?

Thank you for your time. This is the end of our discussion.

For peer review only

C. Focus group discussion guide: FGD with elderly women (older than reproductive age) in the community

1. Let us start with talking about contraceptives / family planning methods in general. How many contraceptive types do you know? Can you describe them?

2. How do you think people in this community perceive contraceptives and family planning in general?
   Probe:
   - Popularity of contraceptives / the idea of family planning in general
   - Specifically, popularity of injectable contraceptives
3. What role do you play in advising your married daughters/daughters-in-law and family members of reproductive age about contraception/family planning?

4. How do you think elderly women (grandmothers) in this community perceive contraceptives and family planning in general?
   Probe:
   - Popularity of contraceptives / family planning in general among elderly women (awareness and knowledge, not use)
   - Specifically, popularity of injectable contraceptives

5. What do elderly women prefer? Which types of contraceptives, mode of delivery (clinic vs. other places, doctor/nurse versus others) do they prefer for their married daughters/daughters-in-law

6. Let us now talk about injectable contraceptives specifically. In your opinion, what factors will make women of reproductive age use or reject injectable contraceptives?
7. Will you support your married daughters/daughters-in-law using injectable contraceptives? Please give reasons for your answer.
8. The government introduced a new program where community health extension workers now administer injectable contraceptives to women away from the clinic, including at homes.

   a. How did you hear about this program? In your opinion, what factors help women to decide to receive injectables through CHEWs?
   b. Willingness to encourage married daughters/daughters-in-law and family members of reproductive age to receive injectable contraceptives from CHEWs?
   c. What do you think might prevent women in your community from using injectable contraceptives through CHEWs?
   d. What kinds of challenges do you know the government has faced with the distribution of injectable contraceptives through CHEWs in your community?

   Probes:
   - Resistance to mode of distribution – facility vs. community based
   - Socio-cultural resistance to injectable contraceptives
   - Resistance to injectable contraceptives being administered by CHEWs rather than other health workers like nurses and doctors

8. What suggestions do you have for how the way injectable contraceptives are administered in your community can be improved to make sure many more women receive them?

Thank you for your time. This is the end of our discussion.

For peer review only

D. Focus group discussion guide: FGD with younger and older men (single and married) in the community

1. Let us start with talking about contraceptives / family planning methods in general. How many contraceptive types do you know? Can you describe them?

2. How do you think people in this community perceive contraceptives and family planning in general?
Probe:
    - Popularity of contraceptives / the idea of family planning in general
    - Specifically, popularity of injectable contraceptives

3. How do you think men in this community perceive contraceptives and family planning in general?
Probe:
    - Popularity of contraceptives / family planning in general among men
    - Specifically, popularity of injectable contraceptives

4. What do men want - which types of contraceptives, mode of delivery (clinic vs. other places, doctor/nurse versus others) do they prefer for their wives
5. Let us now talk about injectable contraceptives specifically. In your opinion, what factors will make women of reproductive age use or reject injectable contraceptives?
6. Will you support your wife/partner/girlfriend using injectable contraceptives? Please give reasons for your answer.
7. The government introduced a new program where community health extension workers now administer injectable contraceptives to women away from the clinic, including at homes.

    a. How did you hear about this program? In your opinion, what factors help women to decide to receive injectables through CHEWs?
    b. Willingness to encourage wife/partner/girlfriend and family members of reproductive age to receive injectable contraceptives from CHEWs?
    c. What do you think might prevent women in your community from using injectable contraceptives through CHEWs?
    d. What kinds of challenges do you know the government has faced with the distribution of injectable contraceptives through CHEWs in your community?

    Probes:
    - Resistance to mode of distribution – facility vs. community based
    - Socio-cultural resistance to injectable contraceptives
    - Resistance to injectable contraceptives being administered by CHEWs rather than other health workers like nurses and doctors

9. What suggestions do you have for how the way injectable contraceptives are administered in your community can be improved to make sure many more women receive them?

Thank you for your time. This is the end of our discussion.

For peer review only

APPENDIX V: Participant Information Sheet for MOH officials

Study title: Scale up of community-based injectable contraceptives in Gombe State, Nigeria

Introduction:

Good day! I am Oluwaseun Akinyemi, a PhD candidate of the School of Public Health, University of the Witwatersrand, Johannesburg. I am doing research on the scale up of community-based injectable contraceptives in Gombe State, Nigeria. Research is just the process to learn the answer to a question. In this study we want to learn what the factors are that may influence the scale up of community-based distribution of injectable contraceptives in Nigeria, in order to understand possible facilitators and barriers.

Invitation to participate: I am therefore asking / inviting you to take part in this research study because you are a senior MOH official who was/is involved with this programme.

What is involved in the study – I am asking different people such as policy makers, program managers, health workers, community leaders, women who are the end-users of injectable contraceptives, and broader members of the community to participate in this study. About 100 people will participate in the study and all will be from Nigeria. If you agree to participate it means you will take part in a key informant interview. Each interview will last for about 60 minutes. In the interviews I will ask questions about your knowledge and your role in the implementation or use of injectable contraceptives as well as what you think might be the challenges and things that work well in implementing this program. There are no right and wrong answers; I am interested in understanding your opinions. Please note that I intend to audio-tape this interview.

Risks and Benefits of being involved in the study – There are no invasive procedures involved in this study, thus no physical harm is envisaged. However, you don't have to answer any question you are not comfortable with.

There are no direct benefits for participating in this study.

The participant will be given pertinent information on the study while involved in the project and after the results are available.

Participation is voluntary: Refusal to participate will involve no penalty or loss of benefits to which you are otherwise entitled. Also, you may discontinue participation at any time without penalty or loss of benefits to which you are otherwise entitled.

Reimbursements for transportation: Transportation costs incurred in order to participate in the study will be reimbursed at the rate of ₦100 per km.

Confidentiality: Efforts will be made to keep personal information confidential. Absolute confidentiality cannot be guaranteed. Personal information may be disclosed if required by law.

For peer review only

Organizations that may inspect and/or copy the research records for quality assurance and data analysis include groups such as the Research Ethics Committee. If results are published, may lead to individual / cohort identification.

Contact details of researcher – for further information / reporting of study related adverse events.

Dr Oluwaseun Akinyemi
Department of Health Policy and Management
College of Medicine
University of Ibadan
Ibadan, Nigeria
+234 803 502 0136
seunakinyemi@hotmail.com

Contact details of UI/UCH Ethics Committee chair (Nigeria) – for more information.

Prof. Catherine O. Falade
Chairperson, University of Ibadan/University College Hospital Ethics Committee
Institute for Advanced Medical Research and Training (IAMRAT)
College of Medicine, University of Ibadan
Ibadan, Nigeria
+234 803 326 4593
cfalade@comui.edu.ng

Contact details of REC administrator and chair (South Africa) – for more information.

Ms Zanele Ndlovu
Research Administrator
Human Research Ethics Committee (Medical)
University of the Witwatersrand
Johannesburg, South Africa
+27 11 717 1252
zanele.ndlovu@wits.ac.za

For peer review only

APPENDIX VI: Participant Information Sheet for NGO programme managers

Study title: Scale up of community-based injectable contraceptives in Gombe State, Nigeria

Introduction:

Good day! I am Oluwaseun Akinyemi, a PhD candidate of the School of Public Health, University of the Witwatersrand, Johannesburg. I am doing research on the scale up of community-based injectable contraceptives in Gombe State, Nigeria. Research is just the process to learn the answer to a question. In this study we want to learn what the factors are that may influence the scale up of community-based distribution of injectable contraceptives in Nigeria, in order to understand possible facilitators and barriers.

Invitation to participate: I am therefore asking / inviting you to take part in this research study because you are a programme manager for an NGO involved with this programme.

What is involved in the study – I am asking different people such as policy makers, program managers, health workers, community leaders, women who are the end-users of injectable contraceptives, and broader members of the community to participate in this study. About 100 people will participate in the study and all will be from Nigeria. If you agree to participate it means you will take part in a key informant interview. Each interview will last for about 60 minutes. In the interviews I will ask questions about your knowledge and your role in the implementation or use of injectable contraceptives as well as what you think might be the challenges and things that work well in implementing this program. There are no right and wrong answers; I am interested in understanding your opinions. Please note that I intend to audio-tape this interview.

Risks and Benefits of being involved in the study – There are no invasive procedures involved in this study, thus no physical harm is envisaged. However, you don't have to answer any question you are not comfortable with.

There are no direct benefits for participating in this study.

The participant will be given pertinent information on the study while involved in the project and after the results are available.

Participation is voluntary: Refusal to participate will involve no penalty or loss of benefits to which you are otherwise entitled. Also, you may discontinue participation at any time without penalty or loss of benefits to which you are otherwise entitled.

Reimbursements for transportation: Transportation costs incurred in order to participate in the study will be reimbursed at the rate of ₦100 per km.

Confidentiality: Efforts will be made to keep personal information confidential. Absolute confidentiality cannot be guaranteed. Personal information may be disclosed if required by law.

For peer review only

Organizations that may inspect and/or copy the research records for quality assurance and data analysis include groups such as the Research Ethics Committee. If results are published, may lead to individual / cohort identification.

Contact details of researcher – for further information / reporting of study related adverse events.

Dr Oluwaseun Akinyemi
Department of Health Policy and Management
College of Medicine
University of Ibadan
Ibadan, Nigeria
+234 803 502 0136
seunakinyemi@hotmail.com

Contact details of UI/UCH Ethics Committee chair (Nigeria) – for more information.

Prof. Catherine O. Falade
Chairperson, University of Ibadan/University College Hospital Ethics Committee
Institute for Advanced Medical Research and Training (IAMRAT)
College of Medicine, University of Ibadan
Ibadan, Nigeria
+234 803 326 4593
cfalade@comui.edu.ng

Contact details of REC administrator and chair (South Africa) – for more information.

Ms Zanele Ndlovu
Research Administrator
Human Research Ethics Committee (Medical)
University of the Witwatersrand
Johannesburg, South Africa
+27 11 717 1252
zanele.ndlovu@wits.ac.za

For peer review only

APPENDIX VII: Participant Information Sheet for health workers

Study title: Scale up of community-based injectable contraceptives in Gombe State, Nigeria

Introduction:

Good day! I am Oluwaseun Akinyemi, a PhD candidate of the School of Public Health, University of the Witwatersrand, Johannesburg. I am doing research on the scale up of community-based injectable contraceptives in Gombe State, Nigeria. Research is just the process to learn the answer to a question. In this study we want to learn what the factors are that may influence the scale up of community-based distribution of injectable contraceptives in Nigeria, in order to understand possible facilitators and barriers.

Invitation to participate: I am therefore asking / inviting you to take part in this research study because you are a health worker who is involved with this programme.

What is involved in the study – I am asking different people such as policy makers, program managers, health workers, community leaders, women who are the end-users of injectable contraceptives, and broader members of the community to participate in this study. About 100 people will participate in the study and all will be from Nigeria. If you agree to participate it means you will take part in a key informant interview. Each interview will last for about 60 minutes. In the interviews I will ask questions about your knowledge and your role in the implementation or use of injectable contraceptives as well as what you think might be the challenges and things that work well in implementing this program. There are no right and wrong answers; I am interested in understanding your opinions. Please note that I intend to audio-tape this interview.

Risks and Benefits of being involved in the study – There are no invasive procedures involved in this study, thus no physical harm is envisaged. However, you don't have to answer any question you are not comfortable with.

There are no direct benefits for participating in this study.

The participant will be given pertinent information on the study while involved in the project and after the results are available.

Participation is voluntary: Refusal to participate will involve no penalty or loss of benefits to which you are otherwise entitled. Also, you may discontinue participation at any time without penalty or loss of benefits to which you are otherwise entitled.

Reimbursements for transportation: Transportation costs incurred in order to participate in the study will be reimbursed at the rate of ₦100 per km.

Confidentiality: Efforts will be made to keep personal information confidential. Absolute confidentiality cannot be guaranteed. Personal information may be disclosed if required by law.

For peer review only

Organizations that may inspect and/or copy the research records for quality assurance and data analysis include groups such as the Research Ethics Committee. If results are published, may lead to individual / cohort identification.

Contact details of researcher – for further information / reporting of study related adverse events.

Dr Oluwaseun Akinyemi
Department of Health Policy and Management
College of Medicine
University of Ibadan
Ibadan, Nigeria
+234 803 502 0136
seunakinyemi@hotmail.com

Contact details of UI/UCH Ethics Committee chair (Nigeria) – for more information.

Prof. Catherine O. Falade
Chairperson, University of Ibadan/University College Hospital Ethics Committee
Institute for Advanced Medical Research and Training (IAMRAT)
College of Medicine, University of Ibadan
Ibadan, Nigeria
+234 803 326 4593
cfalade@comui.edu.ng

Contact details of REC administrator and chair (South Africa) – for more information.

Ms Zanele Ndlovu
Research Administrator
Human Research Ethics Committee (Medical)
University of the Witwatersrand
Johannesburg, South Africa
+27 11 717 1252
zanele.ndlovu@wits.ac.za

For peer review only

APPENDIX VIII: Participant Information Sheet for community leaders

Study title: Scale up of community-based injectable contraceptives in Gombe State, Nigeria

Introduction:

Good day! I am Oluwaseun Akinyemi, a PhD candidate of the School of Public Health, University of the Witwatersrand, Johannesburg. I am doing research on the scale up of community-based injectable contraceptives in Gombe State, Nigeria. Research is just the process to learn the answer to a question. In this study we want to learn what the factors are that may influence the scale up of community-based distribution of injectable contraceptives in Nigeria, in order to understand possible facilitators and barriers.

Invitation to participate: I am therefore asking / inviting you to take part in this research study because you are a community leader.

What is involved in the study – I am asking different people such as policy makers, program managers, health workers, community leaders, women who are the end-users of injectable contraceptives, and broader members of the community to participate in this study. About 100 people will participate in the study and all will be from Nigeria. If you agree to participate it means you will take part in a key informant interview. Each interview will last for about 60 minutes. In the interviews I will ask questions about your knowledge and your role in the implementation or use of injectable contraceptives as well as what you think might be the challenges and things that work well in implementing this program. There are no right and wrong answers; I am interested in understanding your opinions. Please note that I intend to audio-tape this interview.

Risks and Benefits of being involved in the study – There are no invasive procedures involved in this study, thus no physical harm is envisaged. However, you don't have to answer any question you are not comfortable with.

There are no direct benefits for participating in this study.

The participant will be given pertinent information on the study while involved in the project and after the results are available.

Participation is voluntary: Refusal to participate will involve no penalty or loss of benefits to which you are otherwise entitled. Also, you may discontinue participation at any time without penalty or loss of benefits to which you are otherwise entitled.

Reimbursements for transportation: Transportation costs incurred in order to participate in the study will be reimbursed at the rate of ₦100 per km.

Confidentiality: Efforts will be made to keep personal information confidential. Absolute confidentiality cannot be guaranteed. Personal information may be disclosed if required by law.

For peer review only

Organizations that may inspect and/or copy the research records for quality assurance and data analysis include groups such as the Research Ethics Committee. If results are published, may lead to individual / cohort identification.

Contact details of researcher – for further information / reporting of study related adverse events.

Dr Oluwaseun Akinyemi
Department of Health Policy and Management
College of Medicine
University of Ibadan
Ibadan, Nigeria
+234 803 502 0136
seunakinyemi@hotmail.com

Contact details of UI/UCH Ethics Committee chair (Nigeria) – for more information.

Prof. Catherine O. Falade
Chairperson, University of Ibadan/University College Hospital Ethics Committee
Institute for Advanced Medical Research and Training (IAMRAT)
College of Medicine, University of Ibadan
Ibadan, Nigeria
+234 803 326 4593
cfalade@comui.edu.ng

Contact details of REC administrator and chair (South Africa) – for more information.

Ms Zanele Ndlovu
Research Administrator
Human Research Ethics Committee (Medical)
University of the Witwatersrand
Johannesburg, South Africa
+27 11 717 1252
zanele.ndlovu@wits.ac.za

For peer review only

APPENDIX IX: Participant Information Sheet for religious leaders

Study title: Scale up of community-based injectable contraceptives in Gombe State, Nigeria

Introduction:

Good day! I am Oluwaseun Akinyemi, a PhD candidate of the School of Public Health, University of the Witwatersrand, Johannesburg. I am doing research on the scale up of community-based injectable contraceptives in Gombe State, Nigeria. Research is just the process to learn the answer to a question. In this study we want to learn what the factors are that may influence the scale up of community-based distribution of injectable contraceptives in Nigeria, in order to understand possible facilitators and barriers.

Invitation to participate: I am therefore asking / inviting you to take part in this research study because you are a religious leader in this community.

What is involved in the study – I am asking different people such as policy makers, program managers, health workers, community leaders, women who are the end-users of injectable contraceptives, and broader members of the community to participate in this study. About 100 people will participate in the study and all will be from Nigeria. If you agree to participate it means you will take part in a key informant interview. Each interview will last for about 60 minutes. In the interviews I will ask questions about your knowledge and your role in the implementation or use of injectable contraceptives as well as what you think might be the challenges and things that work well in implementing this program. There are no right and wrong answers; I am interested in understanding your opinions. Please note that I intend to audio-tape this interview.

Risks and Benefits of being involved in the study – There are no invasive procedures involved in this study, thus no physical harm is envisaged. However, you don't have to answer any question you are not comfortable with.

There are no direct benefits for participating in this study.

The participant will be given pertinent information on the study while involved in the project and after the results are available.

Participation is voluntary: Refusal to participate will involve no penalty or loss of benefits to which you are otherwise entitled. Also, you may discontinue participation at any time without penalty or loss of benefits to which you are otherwise entitled.

Reimbursements for transportation: Transportation costs incurred in order to participate in the study will be reimbursed at the rate of ₦100 per km.

Confidentiality: Efforts will be made to keep personal information confidential. Absolute confidentiality cannot be guaranteed. Personal information may be disclosed if required by law.

For peer review only

Organizations that may inspect and/or copy the research records for quality assurance and data analysis include groups such as the Research Ethics Committee. If results are published, may lead to individual / cohort identification.

Contact details of researcher – for further information / reporting of study related adverse events.

Dr Oluwaseun Akinyemi
Department of Health Policy and Management
College of Medicine
University of Ibadan
Ibadan, Nigeria
+234 803 502 0136
seunakinyemi@hotmail.com

Contact details of UI/UCH Ethics Committee chair (Nigeria) – for more information.

Prof. Catherine O. Falade
Chairperson, University of Ibadan/University College Hospital Ethics Committee
Institute for Advanced Medical Research and Training (IAMRAT)
College of Medicine, University of Ibadan
Ibadan, Nigeria
+234 803 326 4593
cfalade@comui.edu.ng

Contact details of REC administrator and chair (South Africa) – for more information.

Ms Zanele Ndlovu
Research Administrator
Human Research Ethics Committee (Medical)
University of the Witwatersrand
Johannesburg, South Africa
+27 11 717 1252
zanele.ndlovu@wits.ac.za

For peer review only

APPENDIX X: Participant Information Sheet for women who are current users of injectable contraceptives

Study title: Scale up of community-based injectable contraceptives in Gombe State, Nigeria

Introduction:

Good day! I am Oluwaseun Akinyemi, a PhD candidate of the School of Public Health, University of the Witwatersrand, Johannesburg. I am doing research on the scale up of community-based injectable contraceptives in Gombe State, Nigeria. Research is just the process to learn the answer to a question. In this study we want to learn what the factors are that may influence the scale up of community-based distribution of injectable contraceptives in Nigeria, in order to understand possible facilitators and barriers.

Invitation to participate: I am therefore asking / inviting you to take part in this research study because you use an injectable contraceptive.

What is involved in the study – I am asking different people such as policy makers, program managers, health workers, community leaders, women who are the end-users of injectable contraceptives, and broader members of the community to participate in this study. About 100 people will participate in the study and all will be from Nigeria. If you agree to participate it means you will take part in a key informant interview. Each interview will last for about 60 minutes. In the interviews I will ask questions about your knowledge and your role in the implementation or use of injectable contraceptives as well as what you think might be the challenges and things that work well in implementing this program. There are no right and wrong answers; I am interested in understanding your opinions. Please note that I intend to audio-tape this interview.

Risks and Benefits of being involved in the study – There are no invasive procedures involved in this study, thus no physical harm is envisaged. However, you don't have to answer any question you are not comfortable with.

There are no direct benefits for participating in this study.

The participant will be given pertinent information on the study while involved in the project and after the results are available.

Participation is voluntary: Refusal to participate will involve no penalty or loss of benefits to which you are otherwise entitled. Also, you may discontinue participation at any time without penalty or loss of benefits to which you are otherwise entitled.

Reimbursements for transportation: Transportation costs incurred in order to participate in the study will be reimbursed at the rate of ₦100 per km.

For peer review only

Confidentiality: Efforts will be made to keep personal information confidential. However, confidentiality is not guaranteed in a focus group discussion. Personal information may be disclosed if required by law.

Organizations that may inspect and/or copy the research records for quality assurance and data analysis include groups such as the Research Ethics Committee. If results are published, may lead to individual / cohort identification.

Contact details of researcher – for further information / reporting of study related adverse events.

Dr Oluwaseun Akinyemi
Department of Health Policy and Management
College of Medicine
University of Ibadan
Ibadan, Nigeria
+234 803 502 0136
seunakinyemi@hotmail.com

Contact details of UI/UCH Ethics Committee chair (Nigeria) – for more information.

Prof. Catherine O. Falade
Chairperson, University of Ibadan/University College Hospital Ethics Committee
Institute for Advanced Medical Research and Training (IAMRAT)
College of Medicine, University of Ibadan
Ibadan, Nigeria
+234 803 326 4593
cfalade@comui.edu.ng

Contact details of REC administrator and chair (South Africa– for more information.

Ms Zanele Ndlovu
Research Administrator
Human Research Ethics Committee (Medical)
University of the Witwatersrand
Johannesburg, South Africa
+27 11 717 1252
zanele.ndlovu@wits.ac.za

For peer review only

APPENDIX XI: Participant Information Sheet for women who do not use injectable contraceptives

Study title: Scale up of community-based injectable contraceptives in Gombe State, Nigeria

Introduction:

Good day! I am Oluwaseun Akinyemi, a PhD candidate of the School of Public Health, University of the Witwatersrand, Johannesburg. I am doing research on the scale up of community-based injectable contraceptives in Gombe State, Nigeria. Research is just the process to learn the answer to a question. In this study we want to learn what the factors are that may influence the scale up of community-based distribution of injectable contraceptives in Nigeria, in order to understand possible facilitators and barriers.

Invitation to participate: I am therefore asking / inviting you to take part in this research study because you do not use an injectable contraceptive.

What is involved in the study – I am asking different people such as policy makers, program managers, health workers, community leaders, women who are the end-users of injectable contraceptives, and broader members of the community to participate in this study. About 100 people will participate in the study and all will be from Nigeria. If you agree to participate it means you will take part in a key informant interview. Each interview will last for about 60 minutes. In the interviews I will ask questions about your knowledge and your role in the implementation or use of injectable contraceptives as well as what you think might be the challenges and things that work well in implementing this program. There are no right and wrong answers; I am interested in understanding your opinions. Please note that I intend to audio-tape this interview.

Risks and Benefits of being involved in the study – There are no invasive procedures involved in this study, thus no physical harm is envisaged. However, you don't have to answer any question you are not comfortable with.

There are no direct benefits for participating in this study.

The participant will be given pertinent information on the study while involved in the project and after the results are available.

Participation is voluntary: Refusal to participate will involve no penalty or loss of benefits to which you are otherwise entitled. Also, you may discontinue participation at any time without penalty or loss of benefits to which you are otherwise entitled.

Reimbursements for transportation: Transportation costs incurred in order to participate in the study will be reimbursed at the rate of ₦100 per km.

For peer review only

Confidentiality: Efforts will be made to keep personal information confidential. However, confidentiality is not guaranteed in a focus group discussion. Personal information may be disclosed if required by law.

Organizations that may inspect and/or copy the research records for quality assurance and data analysis include groups such as the Research Ethics Committee. If results are published, may lead to individual / cohort identification.

Contact details of researcher – for further information / reporting of study related adverse events.

Dr Oluwaseun Akinyemi
Department of Health Policy and Management
College of Medicine
University of Ibadan
Ibadan, Nigeria
+234 803 502 0136
seunakinyemi@hotmail.com

Contact details of UI/UCH Ethics Committee chair (Nigeria) – for more information.

Prof. Catherine O. Falade
Chairperson, University of Ibadan/University College Hospital Ethics Committee
Institute for Advanced Medical Research and Training (IAMRAT)
College of Medicine, University of Ibadan
Ibadan, Nigeria
+234 803 326 4593
cfalade@comui.edu.ng

Contact details of REC administrator and chair (South Africa– for more information.

Ms Zanele Ndlovu
Research Administrator
Human Research Ethics Committee (Medical)
University of the Witwatersrand
Johannesburg, South Africa
+27 11 717 1252
zanele.ndlovu@wits.ac.za

For peer review only

APPENDIX XII: Participant Information Sheet for married men

Study title: Scale up of community-based injectable contraceptives in Gombe State, Nigeria

Introduction:

Good day! I am Oluwaseun Akinyemi, a PhD candidate of the School of Public Health, University of the Witwatersrand, Johannesburg. I am doing research on the scale up of community-based injectable contraceptives in Gombe State, Nigeria. Research is just the process to learn the answer to a question. In this study we want to learn what the factors are that may influence the scale up of community-based distribution of injectable contraceptives in Nigeria, in order to understand possible facilitators and barriers.

Invitation to participate: I am therefore asking / inviting you to take part in this research study because you are a married man.

What is involved in the study – I am asking different people such as policy makers, program managers, health workers, community leaders, women who are the end-users of injectable contraceptives, and broader members of the community to participate in this study. About 100 people will participate in the study and all will be from Nigeria. If you agree to participate it means you will take part in a key informant interview. Each interview will last for about 60 minutes. In the interviews I will ask questions about your knowledge and your role in the implementation or use of injectable contraceptives as well as what you think might be the challenges and things that work well in implementing this program. There are no right and wrong answers; I am interested in understanding your opinions. Please note that I intend to audio-tape this interview.

Risks and Benefits of being involved in the study – There are no invasive procedures involved in this study, thus no physical harm is envisaged. However, you don't have to answer any question you are not comfortable with.

There are no direct benefits for participating in this study.

The participant will be given pertinent information on the study while involved in the project and after the results are available.

Participation is voluntary: Refusal to participate will involve no penalty or loss of benefits to which you are otherwise entitled. Also, you may discontinue participation at any time without penalty or loss of benefits to which you are otherwise entitled.

Reimbursements for transportation: Transportation costs incurred in order to participate in the study will be reimbursed at the rate of ₦100 per km.

Confidentiality: Efforts will be made to keep personal information confidential. However, confidentiality is not guaranteed in a focus group discussion. Personal information may be disclosed if required by law.

For peer review only

Organizations that may inspect and/or copy the research records for quality assurance and data analysis include groups such as the Research Ethics Committee. If results are published, may lead to individual / cohort identification.

Contact details of researcher – for further information / reporting of study related adverse events.

Dr Oluwaseun Akinyemi
Department of Health Policy and Management
College of Medicine
University of Ibadan
Ibadan, Nigeria
+234 803 502 0136
seunakinyemi@hotmail.com

Contact details of UI/UCH Ethics Committee chair (Nigeria) – for more information.

Prof. Catherine O. Falade
Chairperson, University of Ibadan/University College Hospital Ethics Committee
Institute for Advanced Medical Research and Training (IAMRAT)
College of Medicine, University of Ibadan
Ibadan, Nigeria
+234 803 326 4593
cfalade@comui.edu.ng

Contact details of REC administrator and chair (South Africa) – for more information.

Ms Zanele Ndlovu
Research Administrator
Human Research Ethics Committee (Medical)
University of the Witwatersrand
Johannesburg, South Africa
+27 11 717 1252
zanele.ndlovu@wits.ac.za

For peer review only

APPENDIX XIII: Participant Information Sheet for unmarried/single men

Study title: Scale up of community-based injectable contraceptives in Gombe State, Nigeria

Introduction:

Good day! I am Oluwaseun Akinyemi, a PhD candidate of the School of Public Health, University of the Witwatersrand, Johannesburg. I am doing research on the scale up of community-based injectable contraceptives in Gombe State, Nigeria. Research is just the process to learn the answer to a question. In this study we want to learn what the factors are that may influence the scale up of community-based distribution of injectable contraceptives in Nigeria, in order to understand possible facilitators and barriers.

Invitation to participate: I am therefore asking / inviting you to take part in this research study because you are an unmarried/ single man.

What is involved in the study – I am asking different people such as policy makers, program managers, health workers, community leaders, women who are the end-users of injectable contraceptives, and broader members of the community to participate in this study. About 100 people will participate in the study and all will be from Nigeria. If you agree to participate it means you will take part in a key informant interview. Each interview will last for about 60 minutes. In the interviews I will ask questions about your knowledge and your role in the implementation or use of injectable contraceptives as well as what you think might be the challenges and things that work well in implementing this program. There are no right and wrong answers; I am interested in understanding your opinions. Please note that I intend to audio-tape this interview.

Risks and Benefits of being involved in the study – There are no invasive procedures involved in this study, thus no physical harm is envisaged. However, you don't have to answer any question you are not comfortable with.

There are no direct benefits for participating in this study.

The participant will be given pertinent information on the study while involved in the project and after the results are available.

Participation is voluntary: Refusal to participate will involve no penalty or loss of benefits to which you are otherwise entitled. Also, you may discontinue participation at any time without penalty or loss of benefits to which you are otherwise entitled.

Reimbursements for transportation: Transportation costs incurred in order to participate in the study will be reimbursed at the rate of ₦100 per km.

Confidentiality: Efforts will be made to keep personal information confidential. However, confidentiality is not guaranteed in a focus group discussion. Personal information may be disclosed if required by law.

For peer review only

Organizations that may inspect and/or copy the research records for quality assurance and data analysis include groups such as the Research Ethics Committee. If results are published, may lead to individual / cohort identification.

Contact details of researcher – for further information / reporting of study related adverse events.

Dr Oluwaseun Akinyemi
Department of Health Policy and Management
College of Medicine
University of Ibadan
Ibadan, Nigeria
+234 803 502 0136
seunakinyemi@hotmail.com

Contact details of UI/UCH Ethics Committee chair (Nigeria) – for more information.

Prof. Catherine O. Falade
Chairperson, University of Ibadan/University College Hospital Ethics Committee
Institute for Advanced Medical Research and Training (IAMRAT)
College of Medicine, University of Ibadan
Ibadan, Nigeria
+234 803 326 4593
cfalade@comui.edu.ng

Contact details of REC administrator and chair (South Africa) – for more information.

Ms Zanele Ndlovu
Research Administrator
Human Research Ethics Committee (Medical)
University of the Witwatersrand
Johannesburg, South Africa
+27 11 717 1252
zanele.ndlovu@wits.ac.za

For peer review only

APPENDIX XIV: Informed Consent Agreement for Senior MoH officials

Study title: Scale up of community-based injectable contraceptives in Gombe State, Nigeria

SERIAL NUMBER ___________________

Background and purpose: One of the health innovations that have been piloted with significant success around the world, including Nigeria, is the community-based distribution of injectable contraceptives. This study will contribute to providing evidence towards aiding our understanding of the barriers and facilitators to the introduction, dissemination, diffusion and integration of the community-based distribution of injectable contraceptives intervention in Nigeria.

Procedures: You have been selected as one of this study's participants for a key informant interview. Each interview will last for about 60 minutes. In the interviews I will ask questions about your knowledge and your role in the implementation or use of injectable contraceptives as well as what you think might be the challenges and things that work well in implementing this program.

Potential Risks: There are no health risks associated with the interviews and discussions.

Potential benefits: The findings of this study will help enhance the scale up of injectable contraceptives in Nigeria thereby helping to promote maternal and child health as well as healthier families.

Reimbursements for "out of pocket" expenses: Transportation costs incurred in order to participate in the study will be reimbursed at the rate of ₦100 per km.

Withdrawal from participation: If you agree to take part you are free to end your participation in this study at any time you want without having to give reasons.

Additional Information: In case you have any question or concern about this study please feel free to contact Dr Oluwaseun Akinyemi, Department of Health Policy and Management, College of Medicine, University of Ibadan Ibadan, Nigeria (Tel. No.: +234 803 502 0136; Email: seunakinyemi@hotmail.com)

Informed consent record: Please read this statement carefully before you sign and if you agree on participating, please fill free to sign this form below to affirm your consent.

I, _________________________________ clearly understand the aims of the project titled **"Scale up of community-based injectable contraceptives in Gombe State, Nigeria"** which has been explained to me by the researcher. I have been given the chance to ask questions and I am satisfied with the answers to all of my questions and I agree to participate in the study. I understand that I may revoke my consent and leave the study at any stage, if I wish so, with no negative consequences.

For peer review only

Participant's name: ____

Participant's signature or thumbprint: __________________ Date: _____________

Witness Name (As appropriate): _______________________________________

Witness signature (As appropriate): _________________ Date: _______________

Study team member's statement: I, the undersigned, have explained to the participant in a language that s/he understands; the procedures to be followed in the study, the risks and benefits involved, and the obligations of the study team.

________________________ ________________________ _____________

Name Signature Date

For peer review only

APPENDIX XV: Informed Consent Agreement for program managers

Study title: Scale up of community-based injectable contraceptives in Gombe State, Nigeria

SERIAL NUMBER ___________________

Background and purpose: One of the health innovations that have been piloted with significant success around the world, including Nigeria, is the community-based distribution of injectable contraceptives. This study will contribute to providing evidence towards aiding our understanding of the barriers and facilitators to the introduction, dissemination, diffusion and integration of the community-based distribution of injectable contraceptives intervention in Nigeria.

Procedures: You have been selected as one of this study's participants for a key informant interview. Each interview will last for about 60 minutes. In the interviews I will ask questions about your knowledge and your role in the implementation or use of injectable contraceptives as well as what you think might be the challenges and things that work well in implementing this program.

Potential Risks: There are no health risks associated with the interviews and discussions.

Potential benefits: The findings of this study will help enhance the scale up of injectable contraceptives in Nigeria thereby helping to promote maternal and child health as well as healthier families.

Reimbursements for "out of pocket" expenses: Transportation costs incurred in order to participate in the study will be reimbursed at the rate of ₦100 per km.

Withdrawal from participation: If you agree to take part you are free to end your participation in this study at any time you want without having to give reasons.

Additional Information: In case you have any question or concern about this study please feel free to contact Dr Oluwaseun Akinyemi, Department of Health Policy and Management, College of Medicine, University of Ibadan Ibadan, Nigeria (Tel. No.: +234 803 502 0136; Email: seunakinyemi@hotmail.com)

Informed consent record: Please read this statement carefully before you sign and if you agree on participating, please fill free to sign this form below to affirm your consent.

I, ________________________________ clearly understand the aims of the project titled **"Scale up of community-based injectable contraceptives in Gombe State, Nigeria"** which has been explained to me by the researcher. I have been given the chance to ask questions and I am satisfied with the answers to all of my questions and I agree to participate in the study. I understand that I may revoke my consent and leave the study at any stage, if I wish so, with no negative consequences.

For peer review only

Participant's name: ____

Participant's signature or thumbprint: ____________________ Date: ______________

Witness Name (As appropriate): ____

Witness signature (As appropriate): ___________________ Date: _________________

Study team member's statement: I, the undersigned, have explained to the participant in a language that s/he understands; the procedures to be followed in the study, the risks and benefits involved, and the obligations of the study team.

___________________________ ___________________________ _______________

Name Signature Date

For peer review only

APPENDIX XVI: Informed Consent Agreement for health workers

Study title: Scale up of community-based injectable contraceptives in Gombe State, Nigeria

SERIAL NUMBER ___________________

Background and purpose: One of the health innovations that have been piloted with significant success around the world, including Nigeria, is the community-based distribution of injectable contraceptives. This study will contribute to providing evidence towards aiding our understanding of the barriers and facilitators to the introduction, dissemination, diffusion and integration of the community-based distribution of injectable contraceptives intervention in Nigeria.

Procedures: You have been selected as one of this study's participants for an in-depth interview. Each interview will last for about 60 minutes. In the interviews and discussions I will ask questions about your knowledge and your role in the implementation or use of injectable contraceptives as well as what you think might be the challenges and things that work well in implementing this program.

Potential Risks: There are no health risks associated with the interviews and discussions.

Potential benefits: The findings of this study will help enhance the scale up of injectable contraceptives in Nigeria thereby helping to promote maternal and child health as well as healthier families.

Reimbursements for "out of pocket" expenses: Transportation costs incurred in order to participate in the study will be reimbursed at the rate of ₦100 per km.

Withdrawal from participation: If you agree to take part you are free to end your participation in this study at any time you want without having to give reasons.

Additional Information: In case you have any question or concern about this study please feel free to contact Dr Oluwaseun Akinyemi, Department of Health Policy and Management, College of Medicine, University of Ibadan Ibadan, Nigeria (Tel. No.: +234 803 502 0136; Email: seunakinyemi@hotmail.com)

Informed consent record: Please read this statement carefully before you sign and if you agree on participating, please fill free to sign this form below to affirm your consent.

I, ________________________________ clearly understand the aims of the project titled **"Scale up of community-based injectable contraceptives in Gombe State, Nigeria"** which has been explained to me by the researcher. I have been given the chance to ask questions and I am satisfied with the answers to all of my questions and I agree to participate in the study. I understand that I may revoke my consent and leave the study at any stage, if I wish so, with no negative consequences.

For peer review only

Participant's name: ____

Participant's signature or thumbprint: ____________________ Date: ______________

Witness Name (As appropriate): ____

Witness signature (As appropriate): ___________________ Date: _________________

Study team member's statement: I, the undersigned, have explained to the participant in a language that s/he understands; the procedures to be followed in the study, the risks and benefits involved, and the obligations of the study team.

___________________________ ___________________________ _______________

Name Signature Date

For peer review only

APPENDIX XVII: Informed Consent Agreement for community leaders

Study title: Scale up of community-based injectable contraceptives in Gombe State, Nigeria

SERIAL NUMBER ___________________

Background and purpose: One of the health innovations that have been piloted with significant success around the world, including Nigeria, is the community-based distribution of injectable contraceptives. This study will contribute to providing evidence towards aiding our understanding of the barriers and facilitators to the introduction, dissemination, diffusion and integration of the community-based distribution of injectable contraceptives intervention in Nigeria.

Procedures: You have been selected as one of this study's participants for an in-depth interview. Each interview will last for about 60 minutes. In the interviews and discussions I will ask questions about your knowledge and your role in the implementation or use of injectable contraceptives as well as what you think might be the challenges and things that work well in implementing this program.

Potential Risks: There are no health risks associated with the interviews and discussions.

Potential benefits: The findings of this study will help enhance the scale up of injectable contraceptives in Nigeria thereby helping to promote maternal and child health as well as healthier families.

Reimbursements for "out of pocket" expenses: Transportation costs incurred in order to participate in the study will be reimbursed at the rate of ₦100 per km.

Withdrawal from participation: If you agree to take part you are free to end your participation in this study at any time you want without having to give reasons.

Additional Information: In case you have any question or concern about this study please feel free to contact Dr Oluwaseun Akinyemi, Department of Health Policy and Management, College of Medicine, University of Ibadan Ibadan, Nigeria (Tel. No.: +234 803 502 0136; Email: seunakinyemi@hotmail.com)

Informed consent record: Please read this statement carefully before you sign and if you agree on participating, please fill free to sign this form below to affirm your consent.

I, _______________________________ clearly understand the aims of the project titled **"Scale up of community-based injectable contraceptives in Gombe State, Nigeria"** which has been explained to me by the researcher. I have been given the chance to ask questions and I am satisfied with the answers to all of my questions and I agree to participate in the study. I understand that I may revoke my consent and leave the study at any stage, if I wish so, with no negative consequences.

For peer review only

Participant's name: ____

Participant's signature or thumbprint: ____________________ Date: ______________

Witness Name (As appropriate): ____

Witness signature (As appropriate): ___________________ Date: _________________

Study team member's statement: I, the undersigned, have explained to the participant in a language that s/he understands; the procedures to be followed in the study, the risks and benefits involved, and the obligations of the study team.

___________________________ ___________________________ _______________

Name Signature Date

For peer review only

APPENDIX XVIII: Informed Consent Agreement for religious leaders

Study title: Scale up of community-based injectable contraceptives in Gombe State, Nigeria

SERIAL NUMBER ___________________

Background and purpose: One of the health innovations that have been piloted with significant success around the world, including Nigeria, is the community-based distribution of injectable contraceptives. This study will contribute to providing evidence towards aiding our understanding of the barriers and facilitators to the introduction, dissemination, diffusion and integration of the community-based distribution of injectable contraceptives intervention in Nigeria.

Procedures: You have been selected as one of this study's participants for an in-depth interview. Each interview will last for about 60 minutes. In the interviews and discussions I will ask questions about your knowledge and your role in the implementation or use of injectable contraceptives as well as what you think might be the challenges and things that work well in implementing this program.

Potential Risks: There are no health risks associated with the interviews and discussions.

Potential benefits: The findings of this study will help enhance the scale up of injectable contraceptives in Nigeria thereby helping to promote maternal and child health as well as healthier families.

Reimbursements for "out of pocket" expenses: Transportation costs incurred in order to participate in the study will be reimbursed at the rate of ₦100 per km.

Withdrawal from participation: If you agree to take part you are free to end your participation in this study at any time you want without having to give reasons.

Additional Information: In case you have any question or concern about this study please feel free to contact Dr Oluwaseun Akinyemi, Department of Health Policy and Management, College of Medicine, University of Ibadan Ibadan, Nigeria (Tel. No.: +234 803 502 0136; Email: seunakinyemi@hotmail.com)

Informed consent record: Please read this statement carefully before you sign and if you agree on participating, please fill free to sign this form below to affirm your consent.

I, _________________________________ clearly understand the aims of the project titled **"Scale up of community-based injectable contraceptives in Gombe State, Nigeria"** which has been explained to me by the researcher. I have been given the chance to ask questions and I am satisfied with the answers to all of my questions and I agree to participate in the study. I understand that I may revoke my consent and leave the study at any stage, if I wish so, with no negative consequences.

For peer review only

Participant's name: ____

Participant's signature or thumbprint: ____________________ Date: ______________

Witness Name (As appropriate): ____

Witness signature (As appropriate): ___________________ Date: _________________

Study team member's statement: I, the undersigned, have explained to the participant in a language that s/he understands; the procedures to be followed in the study, the risks and benefits involved, and the obligations of the study team.

___________________________ ___________________________ _______________

Name Signature Date

For peer review only

APPENDIX XIX: Informed Consent Agreement for women who are current users of injectable contraceptives

Study title: Scale up of community-based injectable contraceptives in Gombe State, Nigeria

SERIAL NUMBER ___________________

Background and purpose: One of the health innovations that have been piloted with significant success around the world, including Nigeria, is the community-based distribution of injectable contraceptives. This study will contribute to providing evidence towards aiding our understanding of the barriers and facilitators to the introduction, dissemination, diffusion and integration of the community-based distribution of injectable contraceptives intervention in Nigeria.

Procedures: You have been selected as one of this study's participants for a focus group discussion or a questionnaire interview. Each interview or focus group discussion will last for about 60 minutes. In the interviews and discussions I will ask questions about your knowledge and your role in the implementation or use of injectable contraceptives as well as what you think might be the challenges and things that work well in implementing this program.

Potential Risks: There are no health risks associated with the interviews and discussions.

Potential benefits: The findings of this study will help enhance the scale up of injectable contraceptives in Nigeria thereby helping to promote maternal and child health as well as healthier families.

Reimbursements for "out of pocket" expenses: Transportation costs incurred in order to participate in the study will be reimbursed at the rate of ₦100 per km.

Withdrawal from participation: If you agree to take part you are free to end your participation in this study at any time you want without having to give reasons.

Additional Information: In case you have any question or concern about this study please feel free to contact Dr Oluwaseun Akinyemi, Department of Health Policy and Management, College of Medicine, University of Ibadan Ibadan, Nigeria (Tel. No.: +234 803 502 0136; Email: seunakinyemi@hotmail.com)

Informed consent record: Please read this statement carefully before you sign and if you agree on participating, please fill free to sign this form below to affirm your consent.

I, ________________________________ clearly understand the aims of the project titled **"Scale up of community-based injectable contraceptives in Gombe State, Nigeria"** which has been explained to me by the researcher. I have been given the chance to ask questions and I am satisfied with the answers to all of my questions and I agree to participate in the study. I understand

that I may revoke my consent and leave the study at any stage, if I wish so, with no negative consequences.

Participant's name: ____

Participant's signature or thumbprint: ____________________ Date: ______________

Witness Name (As appropriate): ____

Witness signature (As appropriate): ___________________ Date: _________________

Study team member's statement: I, the undersigned, have explained to the participant in a language that s/he understands; the procedures to be followed in the study, the risks and benefits involved, and the obligations of the study team.

___________________________ ___________________________ _______________

Name Signature Date

For peer review only

APPENDIX XX: Informed Consent Agreement for women who do not use injectable contraceptives

Study title: Scale up of community-based injectable contraceptives in Gombe State, Nigeria

SERIAL NUMBER ___________________

Background and purpose: One of the health innovations that have been piloted with significant success around the world, including Nigeria, is the community-based distribution of injectable contraceptives. This study will contribute to providing evidence towards aiding our understanding of the barriers and facilitators to the introduction, dissemination, diffusion and integration of the community-based distribution of injectable contraceptives intervention in Nigeria.

Procedures: You have been selected as one of this study's participants for a focus group discussion. Each focus group discussion will last for about 60 minutes. In the interviews and discussions I will ask questions about your knowledge and your role in the implementation or use of injectable contraceptives as well as what you think might be the challenges and things that work well in implementing this program.

Potential Risks: There are no health risks associated with the interviews and discussions.

Potential benefits: The findings of this study will help enhance the scale up of injectable contraceptives in Nigeria thereby helping to promote maternal and child health as well as healthier families.

Reimbursements for "out of pocket" expenses: Transportation costs incurred in order to participate in the study will be reimbursed at the rate of ₦100 per km.

Withdrawal from participation: If you agree to take part you are free to end your participation in this study at any time you want without having to give reasons.

Additional Information: In case you have any question or concern about this study please feel free to contact Dr Oluwaseun Akinyemi, Department of Health Policy and Management, College of Medicine, University of Ibadan Ibadan, Nigeria (Tel. No.: +234 803 502 0136; Email: seunakinyemi@hotmail.com)

Informed consent record: Please read this statement carefully before you sign and if you agree on participating, please fill free to sign this form below to affirm your consent.

I, _______________________________ clearly understand the aims of the project titled **"Scale up of community-based injectable contraceptives in Gombe State, Nigeria"** which has been explained to me by the researcher. I have been given the chance to ask questions and I am satisfied with the answers to all of my questions and I agree to participate in the study. I understand

For peer review only

that I may revoke my consent and leave the study at any stage, if I wish so, with no negative consequences.

Participant's name: ____

Participant's signature or thumbprint: ____________________ Date: ______________

Witness Name (As appropriate): ____

Witness signature (As appropriate): ___________________ Date: _________________

Study team member's statement: I, the undersigned, have explained to the participant in a language that s/he understands; the procedures to be followed in the study, the risks and benefits involved, and the obligations of the study team.

___________________________ ___________________________ _______________

Name Signature Date

For peer review only

APPENDIX XXI: Informed Consent Agreement for married men

Study title: Scale up of community-based injectable contraceptives in Gombe State, Nigeria

SERIAL NUMBER ___________________

Background and purpose: One of the health innovations that have been piloted with significant success around the world, including Nigeria, is the community-based distribution of injectable contraceptives. This study will contribute to providing evidence towards aiding our understanding of the barriers and facilitators to the introduction, dissemination, diffusion and integration of the community-based distribution of injectable contraceptives intervention in Nigeria.

Procedures: You have been selected as one of this study's participants for a focus group discussion. Each focus group discussion will last for about 60 minutes. In the interviews and discussions I will ask questions about your knowledge and your role in the implementation or use of injectable contraceptives as well as what you think might be the challenges and things that work well in implementing this program.

Potential Risks: There are no health risks associated with the interviews and discussions.

Potential benefits: The findings of this study will help enhance the scale up of injectable contraceptives in Nigeria thereby helping to promote maternal and child health as well as healthier families.

Reimbursements for "out of pocket" expenses: Transportation costs incurred in order to participate in the study will be reimbursed at the rate of ₦100 per km.

Withdrawal from participation: If you agree to take part you are free to end your participation in this study at any time you want without having to give reasons.

Additional Information: In case you have any question or concern about this study please feel free to contact Dr Oluwaseun Akinyemi, Department of Health Policy and Management, College of Medicine, University of Ibadan Ibadan, Nigeria (Tel. No.: +234 803 502 0136; Email: seunakinyemi@hotmail.com)

Informed consent record: Please read this statement carefully before you sign and if you agree on participating, please fill free to sign this form below to affirm your consent.

I, _______________________________ clearly understand the aims of the project titled **"Scale up of community-based injectable contraceptives in Gombe State, Nigeria"** which has been explained to me by the researcher. I have been given the chance to ask questions and I am satisfied with the answers to all of my questions and I agree to participate in the study. I understand that I may revoke my consent and leave the study at any stage, if I wish so, with no negative consequences.

For peer review only

Participant's name: ____

Participant's signature or thumbprint: ____________________ Date: ______________

Witness Name (As appropriate): ____

Witness signature (As appropriate): ___________________ Date: _________________

Study team member's statement: I, the undersigned, have explained to the participant in a language that s/he understands; the procedures to be followed in the study, the risks and benefits involved, and the obligations of the study team.

___________________________ ___________________________ _______________

Name Signature Date

For peer review only

APPENDIX XXII: Informed Consent Agreement for unmarried men

Study title: Scale up of community-based injectable contraceptives in Gombe State, Nigeria

SERIAL NUMBER ___________________

Background and purpose: One of the health innovations that have been piloted with significant success around the world, including Nigeria, is the community-based distribution of injectable contraceptives. This study will contribute to providing evidence towards aiding our understanding of the barriers and facilitators to the introduction, dissemination, diffusion and integration of the community-based distribution of injectable contraceptives intervention in Nigeria.

Procedures: You have been selected as one of this study's participants for a focus group discussion. Each focus group discussion will last for about 60 minutes. In the interviews and discussions I will ask questions about your knowledge and your role in the implementation or use of injectable contraceptives as well as what you think might be the challenges and things that work well in implementing this program.

Potential Risks: There are no health risks associated with the interviews and discussions.

Potential benefits: The findings of this study will help enhance the scale up of injectable contraceptives in Nigeria thereby helping to promote maternal and child health as well as healthier families.

Reimbursements for "out of pocket" expenses: Transportation costs incurred in order to participate in the study will be reimbursed at the rate of ₦100 per km.

Withdrawal from participation: If you agree to take part you are free to end your participation in this study at any time you want without having to give reasons.

Additional Information: In case you have any question or concern about this study please feel free to contact Dr Oluwaseun Akinyemi, Department of Health Policy and Management, College of Medicine, University of Ibadan Ibadan, Nigeria (Tel. No.: +234 803 502 0136; Email: seunakinyemi@hotmail.com)

Informed consent record: Please read this statement carefully before you sign and if you agree on participating, please fill free to sign this form below to affirm your consent.

I, ________________________________ clearly understand the aims of the project titled **"Scale up of community-based injectable contraceptives in Gombe State, Nigeria"** which has been explained to me by the researcher. I have been given the chance to ask questions and I am satisfied with the answers to all of my questions and I agree to participate in the study. I understand that I may revoke my consent and leave the study at any stage, if I wish so, with no negative consequences.

For peer review only

Participant's name: ____

Participant's signature or thumbprint: ____________________ Date: ______________

Witness Name (As appropriate): ____

Witness signature (As appropriate): ___________________ Date: _________________

Study team member's statement: I, the undersigned, have explained to the participant in a language that s/he understands; the procedures to be followed in the study, the risks and benefits involved, and the obligations of the study team.

___________________________ ___________________________ _______________

Name Signature Date

For peer review only

APPENDIX XXIII: Informed Consent Agreement to audio record interview senior MoH officials

Study title: Scale up of community-based injectable contraceptives in Gombe State, Nigeria

SERIAL NUMBER ___________________

Background and purpose: One of the health innovations that have been piloted with significant success around the world, including Nigeria, is the community-based distribution of injectable contraceptives.

Additional Information: In case you have any question or concern about this study please feel free to contact Dr Oluwaseun Akinyemi, Department of Health Policy and Management, College of Medicine, University of Ibadan Ibadan, Nigeria (Tel. No.: +234 803 502 0136; Email: seunakinyemi@hotmail.com)

Informed consent to record: Please read this statement carefully before you sign and if you agree that the interview / discussion with you should be audio recorded, please fill free to sign this form below to affirm your consent.

I, ______________________________ clearly understand the aims of the project titled **"Scale up of community-based injectable contraceptives in Gombe State, Nigeria"** which has been explained to me by the researcher. I have been given the chance to ask questions and I am satisfied with the answers to all of my questions and I agree to participate in the study. I understand that I may revoke my consent and leave the study at any stage, if I wish so, with no negative consequences. I agree that the interview / discussion with me should be audio recorded. I understand that the recording is for research purposes only.

Participant's name: ____

Participant's signature or thumbprint: ____________________ Date: ______________

Witness Name (As appropriate): ____

Witness signature (As appropriate): ___________________ Date: _________________

Study team member's statement: I, the undersigned, have explained to the participant in a language that s/he understands; the procedures to be followed in the study (including audio recording of the interview), the risks and benefits involved, and the obligations of the study team.

___________________________ ___________________________ _______________

Name Signature Date

For peer review only

APPENDIX XXIV: Informed Consent Agreement to audio record interview with program managers

Study title: Scale up of community-based injectable contraceptives in Gombe State, Nigeria

SERIAL NUMBER ___________________

Background and purpose: One of the health innovations that have been piloted with significant success around the world, including Nigeria, is the community-based distribution of injectable contraceptives.

Additional Information: In case you have any question or concern about this study please feel free to contact Dr Oluwaseun Akinyemi, Department of Health Policy and Management, College of Medicine, University of Ibadan Ibadan, Nigeria (Tel. No.: +234 803 502 0136; Email: seunakinyemi@hotmail.com)

Informed consent to record: Please read this statement carefully before you sign and if you agree that the interview / discussion with you should be audio recorded, please fill free to sign this form below to affirm your consent.

I, ______________________________ clearly understand the aims of the project titled **"Scale up of community-based injectable contraceptives in Gombe State, Nigeria"** which has been explained to me by the researcher. I have been given the chance to ask questions and I am satisfied with the answers to all of my questions and I agree to participate in the study. I understand that I may revoke my consent and leave the study at any stage, if I wish so, with no negative consequences. I agree that the interview / discussion with me should be audio recorded. I understand that the recording is for research purposes only.

Participant's name: ____

Participant's signature or thumbprint: ____________________ Date: ______________

Witness Name (As appropriate): ____

Witness signature (As appropriate): ___________________ Date: _________________

Study team member's statement: I, the undersigned, have explained to the participant in a language that s/he understands; the procedures to be followed in the study (including audio recording of the interview), the risks and benefits involved, and the obligations of the study team.

___________________________ ___________________________ _______________

Name Signature Date

For peer review only

APPENDIX XXV: Informed Consent Agreement to audio record interview with health workers

Study title: Scale up of community-based injectable contraceptives in Gombe State, Nigeria

SERIAL NUMBER ___________________

Background and purpose: One of the health innovations that have been piloted with significant success around the world, including Nigeria, is the community-based distribution of injectable contraceptives.

Additional Information: In case you have any question or concern about this study please feel free to contact Dr Oluwaseun Akinyemi, Department of Health Policy and Management, College of Medicine, University of Ibadan Ibadan, Nigeria (Tel. No.: +234 803 502 0136; Email: seunakinyemi@hotmail.com)

Informed consent to record: Please read this statement carefully before you sign and if you agree that the interview / discussion with you should be audio recorded, please fill free to sign this form below to affirm your consent.

I, ______________________________ clearly understand the aims of the project titled **"Scale up of community-based injectable contraceptives in Gombe State, Nigeria"** which has been explained to me by the researcher. I have been given the chance to ask questions and I am satisfied with the answers to all of my questions and I agree to participate in the study. I understand that I may revoke my consent and leave the study at any stage, if I wish so, with no negative consequences. I agree that the interview / discussion with me should be audio recorded. I understand that the recording is for research purposes only.

Participant's name: ____

Participant's signature or thumbprint: ____________________ Date: ______________

Witness Name (As appropriate): ____

Witness signature (As appropriate): ___________________ Date: _________________

Study team member's statement: I, the undersigned, have explained to the participant in a language that s/he understands; the procedures to be followed in the study (including audio recording of the interview), the risks and benefits involved, and the obligations of the study team.

___________________________ ___________________________ _______________

Name Signature Date

For peer review only

APPENDIX XXVI: Informed Consent Agreement to audio record interview with community leaders

Study title: Scale up of community-based injectable contraceptives in Gombe State, Nigeria

SERIAL NUMBER ___________________

Background and purpose: One of the health innovations that have been piloted with significant success around the world, including Nigeria, is the community-based distribution of injectable contraceptives.

Additional Information: In case you have any question or concern about this study please feel free to contact Dr Oluwaseun Akinyemi, Department of Health Policy and Management, College of Medicine, University of Ibadan Ibadan, Nigeria (Tel. No.: +234 803 502 0136; Email: seunakinyemi@hotmail.com)

Informed consent to record: Please read this statement carefully before you sign and if you agree that the interview / discussion with you should be audio recorded, please fill free to sign this form below to affirm your consent.

I, ______________________________ clearly understand the aims of the project titled **"Scale up of community-based injectable contraceptives in Gombe State, Nigeria"** which has been explained to me by the researcher. I have been given the chance to ask questions and I am satisfied with the answers to all of my questions and I agree to participate in the study. I understand that I may revoke my consent and leave the study at any stage, if I wish so, with no negative consequences. I agree that the interview / discussion with me should be audio recorded. I understand that the recording is for research purposes only.

Participant's name: ____

Participant's signature or thumbprint: ____________________ Date: ______________

Witness Name (As appropriate): ____

Witness signature (As appropriate): ___________________ Date: _________________

Study team member's statement: I, the undersigned, have explained to the participant in a language that s/he understands; the procedures to be followed in the study (including audio recording of the interview), the risks and benefits involved, and the obligations of the study team.

___________________________ ___________________________ _______________

Name Signature Date

For peer review only

APPENDIX XXVII: Informed Consent Agreement to audio record interview with religious leaders

Study title: Scale up of community-based injectable contraceptives in Gombe State, Nigeria

SERIAL NUMBER ___________________

Background and purpose: One of the health innovations that have been piloted with significant success around the world, including Nigeria, is the community-based distribution of injectable contraceptives.

Additional Information: In case you have any question or concern about this study please feel free to contact Dr Oluwaseun Akinyemi, Department of Health Policy and Management, College of Medicine, University of Ibadan Ibadan, Nigeria (Tel. No.: +234 803 502 0136; Email: seunakinyemi@hotmail.com)

Informed consent to record: Please read this statement carefully before you sign and if you agree that the interview / discussion with you should be audio recorded, please fill free to sign this form below to affirm your consent.

I, ______________________________ clearly understand the aims of the project titled **"Scale up of community-based injectable contraceptives in Gombe State, Nigeria"** which has been explained to me by the researcher. I have been given the chance to ask questions and I am satisfied with the answers to all of my questions and I agree to participate in the study. I understand that I may revoke my consent and leave the study at any stage, if I wish so, with no negative consequences. I agree that the interview / discussion with me should be audio recorded. I understand that the recording is for research purposes only.

Participant's name: ____

Participant's signature or thumbprint: ____________________ Date: ______________

Witness Name (As appropriate): ____

Witness signature (As appropriate): ___________________ Date: _________________

Study team member's statement: I, the undersigned, have explained to the participant in a language that s/he understands; the procedures to be followed in the study (including audio recording of the interview), the risks and benefits involved, and the obligations of the study team.

___________________________ ___________________________ _______________

Name Signature Date

For peer review only

APPENDIX XXVIII: Informed Consent Agreement to audio record focus group discussion with women who currently use injectable contraceptive

Study title: Scale up of community-based injectable contraceptives in Gombe State, Nigeria

SERIAL NUMBER ___________________

Background and purpose: One of the health innovations that have been piloted with significant success around the world, including Nigeria, is the community-based distribution of injectable contraceptives.

Additional Information: In case you have any question or concern about this study please feel free to contact Dr Oluwaseun Akinyemi, Department of Health Policy and Management, College of Medicine, University of Ibadan Ibadan, Nigeria (Tel. No.: +234 803 502 0136; Email: seunakinyemi@hotmail.com)

Informed consent to record: Please read this statement carefully before you sign and if you agree that the interview / discussion with you should be audio recorded, please fill free to sign this form below to affirm your consent.

I, ______________________________ clearly understand the aims of the project titled **"Scale up of community-based injectable contraceptives in Gombe State, Nigeria"** which has been explained to me by the researcher. I have been given the chance to ask questions and I am satisfied with the answers to all of my questions and I agree to participate in the study. I understand that I may revoke my consent and leave the study at any stage, if I wish so, with no negative consequences. I agree that the interview / discussion with me should be audio recorded. I understand that the recording is for research purposes only.

Participant's name: ____

Participant's signature or thumbprint: ____________________ Date: ______________

Witness Name (As appropriate): ____

Witness signature (As appropriate): ___________________ Date: _________________

Study team member's statement: I, the undersigned, have explained to the participant in a language that s/he understands; the procedures to be followed in the study (including audio recording of the discussion), the risks and benefits involved, and the obligations of the study team.

___________________________ ___________________________ _______________

Name Signature Date

For peer review only

APPENDIX XXIX: Informed Consent Agreement to audio record focus group discussion with women who do not use injectable contraceptive

Study title: Scale up of community-based injectable contraceptives in Gombe State, Nigeria

SERIAL NUMBER ___________________

Background and purpose: One of the health innovations that have been piloted with significant success around the world, including Nigeria, is the community-based distribution of injectable contraceptives.

Additional Information: In case you have any question or concern about this study please feel free to contact Dr Oluwaseun Akinyemi, Department of Health Policy and Management, College of Medicine, University of Ibadan Ibadan, Nigeria (Tel. No.: +234 803 502 0136; Email: seunakinyemi@hotmail.com)

Informed consent to record: Please read this statement carefully before you sign and if you agree that the interview / discussion with you should be audio recorded, please fill free to sign this form below to affirm your consent.

I, ______________________________ clearly understand the aims of the project titled **"Scale up of community-based injectable contraceptives in Gombe State, Nigeria"** which has been explained to me by the researcher. I have been given the chance to ask questions and I am satisfied with the answers to all of my questions and I agree to participate in the study. I understand that I may revoke my consent and leave the study at any stage, if I wish so, with no negative consequences. I agree that the interview / discussion with me should be audio recorded. I understand that the recording is for research purposes only.

Participant's name: ____

Participant's signature or thumbprint: ____________________ Date: ______________

Witness Name (As appropriate): ____

Witness signature (As appropriate): ___________________ Date: ________________

Study team member's statement: I, the undersigned, have explained to the participant in a language that s/he understands; the procedures to be followed in the study (including audio recording of the discussion), the risks and benefits involved, and the obligations of the study team.

___________________________ ___________________________ _______________

Name Signature Date

For peer review only

APPENDIX XXX: Informed Consent Agreement to audio record focus group discussion with married men

Study title: Scale up of community-based injectable contraceptives in Gombe State, Nigeria

SERIAL NUMBER ___________________

Background and purpose: One of the health innovations that have been piloted with significant success around the world, including Nigeria, is the community-based distribution of injectable contraceptives.

Additional Information: In case you have any question or concern about this study please feel free to contact Dr Oluwaseun Akinyemi, Department of Health Policy and Management, College of Medicine, University of Ibadan Ibadan, Nigeria (Tel. No.: +234 803 502 0136; Email: seunakinyemi@hotmail.com)

Informed consent to record: Please read this statement carefully before you sign and if you agree that the interview / discussion with you should be audio recorded, please fill free to sign this form below to affirm your consent.

I, ______________________________ clearly understand the aims of the project titled **"Scale up of community-based injectable contraceptives in Gombe State, Nigeria"** which has been explained to me by the researcher. I have been given the chance to ask questions and I am satisfied with the answers to all of my questions and I agree to participate in the study. I understand that I may revoke my consent and leave the study at any stage, if I wish so, with no negative consequences. I agree that the interview / discussion with me should be audio recorded. I understand that the recording is for research purposes only.

Participant's name: ____

Participant's signature or thumbprint: ____________________ Date: ______________

Witness Name (As appropriate): ____

Witness signature (As appropriate): ___________________ Date: _________________

Study team member's statement: I, the undersigned, have explained to the participant in a language that s/he understands; the procedures to be followed in the study (including audio recording of the discussion), the risks and benefits involved, and the obligations of the study team.

___________________________ ___________________________ _______________

Name Signature Date

For peer review only

APPENDIX XXXI: Informed Consent Agreement to audio record focus group discussion with single/unmarried men

Study title: Scale up of community-based injectable contraceptives in Gombe State, Nigeria

SERIAL NUMBER ___________________

Background and purpose: One of the health innovations that have been piloted with significant success around the world, including Nigeria, is the community-based distribution of injectable contraceptives.

Additional Information: In case you have any question or concern about this study please feel free to contact Dr Oluwaseun Akinyemi, Department of Health Policy and Management, College of Medicine, University of Ibadan Ibadan, Nigeria (Tel. No.: +234 803 502 0136; Email: seunakinyemi@hotmail.com)

Informed consent to record: Please read this statement carefully before you sign and if you agree that the interview / discussion with you should be audio recorded, please fill free to sign this form below to affirm your consent.

I, ______________________________ clearly understand the aims of the project titled **"Scale up of community-based injectable contraceptives in Gombe State, Nigeria"** which has been explained to me by the researcher. I have been given the chance to ask questions and I am satisfied with the answers to all of my questions and I agree to participate in the study. I understand that I may revoke my consent and leave the study at any stage, if I wish so, with no negative consequences. I agree that the interview / discussion with me should be audio recorded. I understand that the recording is for research purposes only.

Participant's name: ____

Participant's signature or thumbprint: ____________________ Date: ______________

Witness Name (As appropriate): ____

Witness signature (As appropriate): ___________________ Date: _________________

Study team member's statement: I, the undersigned, have explained to the participant in a language that s/he understands; the procedures to be followed in the study (including audio recording of the discussion), the risks and benefits involved, and the obligations of the study team.

___________________________ ___________________________ _______________

Name Signature Date

For peer review only

APPENDIX XXXII: Distress Protocol

Distress Protocol 1: The protocol for managing distress in the context of a research focus group /interview

(Adapted from: Draucker C B, Martsolf D S and Poole C (2009) Developing Distress Protocols for research on Sensitive Topics. *Archives of Psychiatric Nursing* 23 (5) pp 343-350)

Distress

• A participant indicates they are experiencing a high level of stress or emotional distress

OR

• Exhibit behaviours suggestive that the discussion/interview is too stressful such as uncontrolled crying, shaking etc.

Stage 1 Response

• Stop the discussion/interview.

• The researcher (who is a health professional) will offer immediate support

• Assess mental status:

  - Tell me what thoughts you are having?
  - Tell me what you are feeling right now?
  - Do you feel you are able to go on about your day?
  - Do you feel safe?

Review

•If participant feels able to carry on; resume interview/discussion

•If participant is unable to carry on, Go to stage 2

Stage 2 Response

•Remove participant from discussion and accompany to quiet area or discontinue interview

•Encourage the participant to contact their GP or mental health provider

OR

•Offer, with participant consent, for a member of the research team to do so OR

•With participant consent contact a member of the health care team treating them for further advice/support

Follow up

•Follow participant up with courtesy call (if participant consents)

OR

•Encourage the participant to call either if he/she experiences increased distress in the hours/days following the focus group

For peer review only

Distress Protocol 2: The protocol for managing distress in the context of a research focus group /interview management

Adapted from: McCosker,H Barnard, A Gerber, R (2001). Undertaking Sensitive Research: Issues and Strategies for Meeting the Safety Needs of All. Forum: Qualitative Social Research, 2(1)

Pre-data collection

•The researcher would consider the potential physical and psychological impact on the researcher of the participants' description of life experiences.

•The researcher would consider how many interviews could be undertaken in a week.

•The researcher would be aware of the potential for emotional exhaustion.

Data collection stage

• For topics that are potentially sensitive/distressing data collection would be undertaken by two members of the research team.

•Regular scheduled debriefing sessions with the lead researcher.

•Research team would be encouraged to journal their thoughts and feelings which may then become part of fieldwork notes in some research approaches.

Analysis

•Research team would be alerted prior to transcription review of potentially "challenging" or "difficult" interviews

•The research team would have regular scheduled debriefing sessions with the lead researcher

Follow up

•Researcher will access a research mentor (supervisor) if he experiences increased distress in the hours/days following transcription.

For peer review only

Distress Protocol 3: The protocol for managing distress in the context of a research focus group /interview transcription

(Adapted from: Gregory, D Russell, C Phillips, L (1997). Beyond textual perfection: transcribers as vulnerable persons. Qualitative Health Research, 7(2), 294-300.)

Pre-data collection

• The transcriber would be considered in the research proposal, with a clear indication of how s/he will be provided with a "safe" working environment while also maintaining the "quality" of the research.

Ethical review stage

• The transcriber would be included in the ethical clearance process.

• The transcriber would be informed of the nature of the research and the type of data.

Pre-transcription

• The transcriber would be alerted prior to the transcription of potentially "challenging" or "difficult" interviews.

•The transcriber would have regular scheduled debriefing sessions with a named member of the research team.

During Transcription

•Transcriber would have prompt access to an appropriate person for crisis counselling.

•S/he would have a clearly documented termination from the transcription process that includes resolution of personal issues which arose as a consequence of the work.

•Transcriber would be encouraged to journal her/his thoughts and feelings which may then become part of fieldwork notes.

Follow up

•Follow transcriber up with courtesy call (if transcriber consents)

OR

•Encourage the transcriber to call if s/he experiences increased distress in the hours/days following transcription

For peer review only

Reporting checklist for qualitative study.

Based on the SRQR guidelines.

Instructions to authors

Complete this checklist by entering the page numbers from your manuscript where readers will find each of the items listed below.

Your article may not currently address all the items on the checklist. Please modify your text to include the missing information. If you are certain that an item does not apply, please write "n/a" and provide a short explanation.

Upload your completed checklist as an extra file when you submit to a journal.

In your methods section, say that you used the SRQRreporting guidelines, and cite them as:

O'Brien BC, Harris IB, Beckman TJ, Reed DA, Cook DA. Standards for reporting qualitative research: a synthesis of recommendations. Acad Med. 2014;89(9):1245-1251.

		Reporting Item	Page Number
**Title**			
	#1	Concise description of the nature and topic of the study identifying the study as qualitative or indicating the approach (e.g. ethnography, grounded theory) or data collection methods (e.g. interview, focus group) is recommended	1

For peer review only

**Abstract**			
	#2	Summary of the key elements of the study using the abstract format of the intended publication; typically includes background, purpose, methods, results and conclusions	2
**Introduction**			
Problem formulation	#3	Description and signifcance of the problem / phenomenon studied: review of relevant theory and empirical work; problem statement	4-5
Purpose or research question	#4	Purpose of the study and specific objectives or questions	5
**Methods**			
Qualitative approach and research paradigm	#5	Qualitative approach (e.g. ethnography, grounded theory, case study, phenomenolgy, narrative research) and guiding theory if appropriate; identifying the research paradigm (e.g. postpositivist, constructivist / interpretivist) is also recommended; rationale. The rationale should briefly discuss the justification for choosing that theory, approach, method or technique rather than other options available; the assumptions and limitations implicit in those choices and how those choices influence study conclusions and transferability.	6

For peer review only

		As appropriate the rationale for several items might be discussed together.	
Researcher characteristics and reflexivity	#6	Researchers' characteristics that may influence the research, including personal attributes, qualifications / experience, relationship with participants, assumptions and / or presuppositions; potential or actual interaction between researchers' characteristics and the research questions, approach, methods, results and / or transferability	6
Context	#7	Setting / site and salient contextual factors; rationale	5
Sampling strategy	#8	How and why research participants, documents, or events were selected; criteria for deciding when no further sampling was necessary (e.g. sampling saturation); rationale	5-6
Ethical issues pertaining to human subjects	#9	Documentation of approval by an appropriate ethics review board and participant consent, or explanation for lack thereof; other confidentiality and data security issues	7
Data collection methods	#10	Types of data collected; details of data collection procedures including (as appropriate) start and stop dates of data collection and analysis, iterative process, triangulation of sources / methods, and modification of procedures in response to evolving study findings; rationale	5-6

For peer review only

Data collection instruments and technologies	#11	Description of instruments (e.g. interview guides, questionnaires) and devices (e.g. audio recorders) used for data collection; if / how the instruments(s) changed over the course of the study	6
Units of study	#12	Number and relevant characteristics of participants, documents, or events included in the study; level of participation (could be reported in results)	5-6
Data processing	#13	Methods for processing data prior to and during analysis, including transcription, data entry, data management and security, verification of data integrity, data coding, and anonymisation / deidentification of excerpts	6
Data analysis	#14	Process by which inferences, themes, etc. were identified and developed, including the researchers involved in data analysis; usually references a specific paradigm or approach; rationale	6-7
Techniques to enhance trustworthiness	#15	Techniques to enhance trustworthiness and credibility of data analysis (e.g. member checking, audit trail, triangulation); rationale	7
**Results/findings**			
Syntheses and interpretation	#16	Main findings (e.g. interpretations, inferences, and themes); might include development of a theory or model, or integration with prior research or theory	7-12

For peer review only

Links to empirical data	#17	Evidence (e.g. quotes, field notes, text excerpts, photographs) to substantiate analytic findings	7-12
**Discussion**			
Intergration with prior work, implications, transferability and contribution(s) to the field	#18	Short summary of main findings; explanation of how findings and conclusions connect to, support, elaborate on, or challenge conclusions of earlier scholarship; discussion of scope of application / generalizability; identification of unique contributions(s) to scholarship in a discipline or field	12-14
Limitations	#19	Trustworthiness and limitations of findings	14
**Other**			
Conflicts of interest	#20	Potential sources of influence of perceived influence on study conduct and conclusions; how these were managed	3
Funding	#21	Sources of funding and other support; role of funders in data collection, interpretation and reporting	3

None The SRQR checklist is distributed with permission of Wolters Kluwer © 2014 by the Association of American Medical Colleges. This checklist can be completed online using https://www.goodreports.org/, a tool made by the EQUATOR Network in collaboration with Penelope.ai

For peer review only

BMJ Open

"Our culture prohibits some things": Qualitative inquiry into how sociocultural context influences the scale up of community-based injectable contraceptives in Nigeria

Journal:	*BMJ Open*
Manuscript ID	bmjopen-2019-035311.R1
Article Type:	Original research
Date Submitted by the Author:	04-Feb-2020
Complete List of Authors:	Akinyemi, Oluwaseun; University of Ibadan College of Medicine, Health Policy and Management; University of the Witwatersrand School of Public Health, Community Health<br>Harris, Bronwyn; Warwick Medical School, University of Warwick, Coventry, Division of Health Sciences, ; University of the Witwatersrand, Centre for Health Policy, School of Public Health<br>Kawonga, Mary; University of the Witwatersrand, School of Public Health
**Primary Subject Heading**:	Health policy
Secondary Subject Heading:	Evidence based practice, Health policy, Qualitative research, Public health, Reproductive medicine
Keywords:	Reproductive medicine < GYNAECOLOGY, HEALTH SERVICES ADMINISTRATION & MANAGEMENT, Health policy < HEALTH SERVICES ADMINISTRATION & MANAGEMENT, PREVENTIVE MEDICINE, PUBLIC HEALTH, QUALITATIVE RESEARCH

SCHOLARONE™
Manuscripts

I, the Submitting Author has the right to grant and does grant on behalf of all authors of the Work (as defined in the below author licence), an exclusive licence and/or a non-exclusive licence for contributions from authors who are: i) UK Crown employees; ii) where BMJ has agreed a CC-BY licence shall apply, and/or iii) in accordance with the terms applicable for US Federal Government officers or employees acting as part of their official duties; on a worldwide, perpetual, irrevocable, royalty-free basis to BMJ Publishing Group Ltd ("BMJ") its licensees and where the relevant Journal is co-owned by BMJ to the co-owners of the Journal, to publish the Work in this journal and any other BMJ products and to exploit all rights, as set out in our licence.

The Submitting Author accepts and understands that any supply made under these terms is made by BMJ to the Submitting Author unless you are acting as an employee on behalf of your employer or a postgraduate student of an affiliated institution which is paying any applicable article publishing charge ("APC") for Open Access articles. Where the Submitting Author wishes to make the Work available on an Open Access basis (and intends to pay the relevant APC), the terms of reuse of such Open Access shall be governed by a Creative Commons licence – details of these licences and which Creative Commons licence will apply to this Work are set out in our licence referred to above.

Other than as permitted in any relevant BMJ Author's Self Archiving Policies, I confirm this Work has not been accepted for publication elsewhere, is not being considered for publication elsewhere and does not duplicate material already published. I confirm all authors consent to publication of this Work and authorise the granting of this licence.

For peer review only

"Our culture prohibits some things": Qualitative inquiry into how sociocultural context influences the scale up of community-based injectable contraceptives in Nigeria

Oluwaseun Akinyemi,[a,d*] (ooakinyemi@comui.edu.ng, seunakinyemi@hotmail.com)

Bronwyn Harris,[b,c] (b.harris.2@warwick.ac.uk)

Mary Kawonga,[d] (mary.kawonga@wits.ac.za)

[a] *Department of Health Policy and Management, College of Medicine, University of Ibadan*

[b] *Centre for Health Policy, School of Public Health, Faculty of Health Sciences, University of the Witwatersrand, Johannesburg*

[c] *Division of Health Sciences, Warwick Medical School, University of Warwick, Coventry*

[d] *Department of Community Health, School of Public Health, Faculty of Health Sciences, University of the Witwatersrand, Johannesburg*

Original research article

*Corresponding author: Dr O.O. Akinyemi
Department of Health Policy and Management
College of Medicine, University of Ibadan
Ibadan, Nigeria
+234 803 502 0136

Word counts:

Abstract: 283

Manuscript: 4697

Running Title:

Sociocultural context and innovation scale up

For peer review only

Abstract

Objectives To explore how sociocultural factors may support or impede the adoption of community-based distribution of injectable contraceptives in Nigeria.

Design A qualitative study based on a grounded theory approach was conducted through in-depth interviews and focus group discussions.

Setting Most participants lived in Gombe State, North East Nigeria. Other participants were from Ibadan (South West) and Abuja (Federal capital territory).

Participants Through seven key informant interviews, 15 in-depth interviews and 10 focus group discussions, 102 participants were involved in the study.

Methods This study, conducted in 2016 was part of a larger study on scale up of community-based distribution of injectable contraceptives. Qualitative data were collected from traditional and religious leaders, health workers and community members. The data were audio recorded, transcribed and analysed using a thematic framework method.

Results Sociocultural challenges to scale up included patriarchy and men's fear of losing control over their spouses, traditional and religious beliefs about fertility, and myths about contraceptives and family planning. As a result of deep-rooted beliefs that children are 'divine blessings' and that procreation should not be regulated, participants described a subtle resistance to uptake of injectable contraceptives. Since Gombe is largely a patriarchal society, male involvement emerged as important to the success of meaningful innovation uptake. Community leaders largely described their participation in the scale up process as active, although they also identified scope for further involvement and recognition.

Conclusion Scale up is more than setting up health sector implementing structures, training health workers and getting innovation supplies, but also requires preparedness which includes paying attention to complex contextual issues. Policy implementers should also see scale up as a learning process and be willing to move at the speed of the community.

.

Strengths and limitations of this study

- The study participants represented a range of stakeholders - users of injectable contraceptives, community members, providers and health system managers.

For peer review only - http://bmjopen.bmj.com/site/about/guidelines.xhtml

- The study provided stakeholders who participated a distinct experience and some observations to reflect upon since the study was based on a current program, rather than speaking merely about theoretical responses they or their community members may have to a health intervention.
- Our results highlighted that scale up is influenced by several socio-cultural factors; thus, showing the importance of paying attention to complex contextual issues during innovation uptake.
- The findings of our study emphasized how health systems and communities should interact in order to ensure successful scale up of health innovations.
- Social desirability bias is a possibility as some of the participants in this study were involved in the community-based distribution intervention

Keywords: Nigeria; Scale up; Community-based distribution of injectable contraceptives; Community participation; Male involvement; Stakeholder collaboration; Sociocultural context

Funding

This research was supported by the Consortium for Advanced Research Training in Africa (CARTA). CARTA is jointly led by the African Population and Health Research Center and the University of the Witwatersrand and funded by the Carnegie Corporation of New York (Grant No--B 8606.R02), Sida (Grant No:54100113), the DELTAS Africa Initiative (Grant No: 107768/Z/15/Z) and Deutscher Akademischer Austauschdienst (DAAD). The DELTAS Africa Initiative is an independent funding scheme of the African Academy of Sciences (AAS)'s Alliance for Accelerating Excellence in Science in Africa (AESA) and supported by the New Partnership for Africa's Development Planning and Coordinating Agency (NEPAD Agency) with funding from the Wellcome Trust (UK) and the UK government. The statements made and views expressed are solely the responsibility of the authors.

Declarations of interest

None.

For peer review only - http://bmjopen.bmj.com/site/about/guidelines.xhtml

1. Introduction

Scale up of effective health innovations, through their increased availability to a larger population over a wider geographic area, is essential for achieving universal health coverage, particularly in developing countries.[1 2] Scale up is influenced by various factors related to the innovation, the users, and the social context in which the innovation is introduced.[3] The nature of the innovation itself, preferences and characteristics of the intended users (including their willingness to adopt the innovation), and ways that the innovation is adapted to the local context are also some of the factors affecting innovation scale up.[4]

The sociocultural context, which includes religious and cultural beliefs, gender and societal norms and attitudes, is an important predictor of adoption of a health innovation in a population.[5 6] The sociocultural context may promote health or be a barrier to healthcare utilization.[7] For example, cultural beliefs and practices have been shown to influence the uptake of a range of health innovations including male circumcision,[8] breast cancer screening[7 9] and contraceptives.[10 11] Without due consideration of the sociocultural context, health innovation development, delivery and scale up is likely to be limited, and this may lead to waste of scarce health resources including time.[12] According to Bradley *et al,*[3] assessing the receptivity and willingness of community members to adopt a health innovation within their environmental and sociocultural settings is one of the key first steps in the scale up process. In their conception, poor understanding by implementers of potential users' sociocultural context will limit scale up efforts, while good understanding will help program managers to recognise contextual factors that may enhance or inhibit the uptake of an innovation.[3]

Evidence highlights the influence of sociocultural context on the adoption of contraceptive innovations. For example, in a study in rural Poland, Colleran and Mace[13] reported that religious and sociocultural factors, particularly social networks, influence the uptake of contraceptive use more than individual characteristics. Dalaba and colleagues[14] in their study in northern Ghana reported that inadequate male participation led to resistance towards female reproductive freedom. Interventions at societal level, such as adequate male mobilisation coupled with appropriate community-based distribution strategies were shown to be effective in improving contraceptive uptake.[14] In Nigeria, where society is largely patriarchal, with consequences for access to and use of contraceptives, a study found that about two-thirds of rural women who had spousal approval were reported to use

For peer review only - http://bmjopen.bmj.com/site/about/guidelines.xhtml

contraceptives while all those who were forbidden by their husbands did not use any family planning method.[15] According to the last Demographic and Health Survey in Nigeria, contraceptive prevalence rate was 14.3% while only 2.4% of women of reproductive age group use injectable contraceptive. Understanding whether and how the sociocultural context influences scale up of contraceptive innovation is thus important.

Evidence suggests that community-based distribution of contraceptives is still needed in developing countries particularly rural or isolated communities in order to increase contraceptive prevalence rate.[16-18] The full potential of CBD to improve CPR has not been fully realized in Sub-Saharan Africa.[19] Nigeria started its family planning policy review in 2007 and successfully piloted the community-based distribution of injectable contraceptives through community health extension workers (CHEWs) as a new innovation in the health system in 2010 in Gombe State, North-eastern region.[20 21] Afterwards, the scale up of this innovation started in 2014, led by a national non-governmental organization and supported by international donor agencies.[22]

However, the understanding of how sociocultural contextual factors may facilitate or impede the successful scale up of the contraceptive innovation beyond the initial pilot site in Nigeria is limited. This study took place after scale up started, thus it adopted both a retrospective slant to assess factors that affected scale up and forward-looking approach to what may affect future scale up effort (see study timeline in Figure 1). The objective of this study is therefore to explore how sociocultural factors may influence the scale up of the community-based distribution of injectable contraceptives in Nigeria.

[Figure 1]

2. Methods

2.1 Study design and setting

This cross-sectional study conducted from September to November 2016, was part of a larger research project[23 24] which explored the interplay between barriers and facilitators of scale up of the community-based distribution of injectable contraceptives guided by the AIDED model[3] in order to inform wider scale up of this innovation in Nigeria. The project took place

For peer review only - http://bmjopen.bmj.com/site/about/guidelines.xhtml

in Gombe (North eastern Nigeria), one of 36 States in Nigeria and a predominantly Muslim community. Gombe State, with a population of 2,353,879 people according to the 2006 population census,[25] has one of the lowest contraceptive prevalence rates in the country (3.5% for modern methods and 4.0% for any method) and one of the highest maternal mortality rates (1,726/100,000).[20 26 27] Gombe was purposively selected because it was the setting for the national pilot test for community-based distribution of injectable contraceptives in 2010. Gombe is divided administratively into 11 Local Government Areas (LGAs). The study was conducted in two LGAs – Gombe (A) and Yamaltu/Deba (B). Although participants were primarily based in Gombe State, one interview took place in another state (Ibadan in the South west) and 3 interviews in Abuja at national level (Federal Capital Territory).

Participants and sample

In total, 102 people participated in this study (see Table 1). The participants were selected through a purposive sampling method, where study respondents were recruited based on characteristics of interest, availability, and ability to provide relevant information[28 29] about the research question: how may sociocultural context influence the process of introducing, translating and integrating health innovation? Participants included a range of stakeholders, including traditional and religious leaders, health workers as well as community members. One FGD each was conducted with different types of participants who were involved and could give an account based on their experiences of the program with the aim of exploring qualitatively the views, concerns from this range of key role players, not aiming to quantify, compare or rank frequency of views. Each of the FGDs had eight participants. The selected participants were therefore deemed appropriate role players to share their own experience and views as well as reflect on and summarize the views of others about the implementation of the community-based distribution of injectable contraceptives within their sociocultural context. The number of interviews and FGDs was determined by the principles of saturation, a point where no new information is emerging,[30 31] and availability of resources as well as study time.[32] The interviews and FGDs were conducted by the first author assisted by three trained research assistants.

This study adopted the inductive thematic analysis. This approach was guided by available literature as well as insights from the participants – codes were generated from key themes identified from transcripts.[30 33-35]

For peer review only - http://bmjopen.bmj.com/site/about/guidelines.xhtml

[Table 1]

2.2 Data collection

Data were collected through seven key informant interviews (KIIs), 15 in-depth interviews (IDIs) and 10 focus group discussions (FGDs). KIIS, IDIs and FGDs were conducted with senior stakeholders, healthcare workers and community members, respectively. Guides were used to conduct the interviews and focus group discussions (see Supplementary file 1). All guides were translated to the predominant local language - Hausa. The main issues covered during the interviews and discussions included community perceptions about contraceptives and specifically about the innovation (community-based delivery of contraceptives), their role in the scale up process, as well as their views regarding the sociocultural challenges and facilitators of the scale up process and community engagement regarding the scale up. Interviews and discussions were tape-recorded and later transcribed in full and translated where necessary. Transcription was done using a protocol which emphasizes verbatim transcription of recordings. Both transcription and translation were done by the same research associate who is vast in both Hausa and English languages. Back translation was subsequently done by another associate vast in the two languages; the back translations were afterwards compared with the original transcripts in order to ensure that the original meaning is retained.[36]

2.3 Data analysis

Transcripts were analyzed iteratively with the aid of the NVIVO (version 10) software using the inductive thematic approach.[30 31 34 35] Coding were done by the first and last authors independently at first before analysis started; these codes were then compared and harmonized. Emerging codes from the transcripts were added to pre-defined themes: community members' perceptions regarding community-based distribution of injectable contraceptives, community members' acceptance (or rejection) of contraceptives, and community engagement in the scale up process. Emerging themes were indexed and compared with themes from subsequent interviews until saturation was attained. A codebook was developed after all the data were collected; this was then applied to the transcripts. Although number of interviews and FGDs were set at the outset based on empirical guidance,

For peer review only - http://bmjopen.bmj.com/site/about/guidelines.xhtml

however during analysis, it was confirmed that saturation,[30] where no new information was obtained, was actually reached before the cap was reached in both interviews and FGDs. Analysis progressed alongside data collection. The SRQR reporting guidelines for qualitative studies[37] was used in preparing this manuscript.

2.4 Ethics approval and consent to participate

Ethical approval for this study was obtained from the University of Ibadan/University College Hospital Ethical Review Board (Reference No.: UI/EC/16/0022) as well as the Human Research Ethics Committee (Medical) of the University of the Witwatersrand (Reference No.: M160737). Written informed consent were obtained from study participants before the interviews and FGDs (see Supplementary file 2). Participants were also assured of confidentiality by ensuring that all identifiers were removed from the data and that only the research team has access to the data.

2.5 Patient and Public Involvement

The study participants were involved in shaping the study particularly during the pre-test which led to the modification of some ambiguous questions in the guides. Also, some participants helped with the recruitment of other respondents through a snowball sampling[38] procedure. Furthermore, findings from this study and the larger research project have been presented in conferences with useful feedback; a policy brief has been developed to be disseminated to policy makers.

3. Results

Of the 102 study participants, 59 (57.8%) were female (Table 1) with a mean age of 42.8 ± 14.2 years. Similarly, mean ages of FGD and interview participants were 42.7 ± 14.6 and 46.7 ± 6.0 years, respectively. Six of the 10 FGDs were made up of women participants, each FGD comprising of eight respondents. Participants described a number of sociocultural factors that challenged the scale up process right from the pilot stage; these include traditional and religious norms about fertility, perception about family planning including adverse effects as well as patriarchy and men's fear of losing control of their spouses (Figure 2).

[Figure 2]

For peer review only

3.1 Deep rooted cultural and religious beliefs about fertility

Interview participants spoke about a deep-rooted belief amongst community members that children are 'divine blessings' and that procreation should not be regulated. Related to this world view, participants described a subtle resistance to the uptake of injectable contraceptives, more passive than overt in its expression by individuals and families who would feign acceptance but refuse the intervention when given.

> "*…there will always be resistance to this use of contraceptives, because here, we are people that believe that children are a blessing, so you give birth to as many as possible. …The resistance may not be as a group, but when you come to individual levels… as a government, they might not show you the resistance, but when it comes to the actual usage, that's where you start having problems*." (IDI, male, Doctor, SMoH)

Also, there were issues about religious beliefs influencing perception about family planning/ contraceptive use at the community level. Religiosity, that is, strong adherence to religious beliefs, was said to be one of the major factors impeding the uptake of contraceptives in general.

> "*…when you look at northern Nigeria, majority are Muslims and when you look at the south eastern states, you know we have issue of religion, issue of uptake of all contraceptives, not even injectables alone. To the south eastern man, most of them are Catholics, they frown at family planning, so also here (northern Nigeria) people frown at family planning in general*" (KII, male, Senior official, SMoH).

3.2 Awareness and perceptions regarding family planning

Most interview respondents agreed that there was a need to continue to educate community members about the overall benefits of contraceptives and family planning as well as emphasize possible side effects of contraceptives use among women. Also, informants were of the opinion that lack of knowledge about benefits of contraception contributes to reluctance to adopt the innovation. In the words of a respondent:

> "*…the community and people at the grassroots have little information (on contraceptives)*" (KII, female, Senior official, SMoH).

For peer review only - http://bmjopen.bmj.com/site/about/guidelines.xhtml

These challenges were further compounded by reports of adverse effects of the contraception from some women especially "*severe bleeding during menstruation*" (IDI, female, CHEW). Healthcare providers felt that to effectively deal with these challenges, it was important to listen sympathetically to the women and treat reported side effects, in line with the training received for the innovation scale up. Education was also given when clients had questions in order to resolve doubts about the innovation. For example, a respondent said:

> "*We listen to them, and we explain to them that since it (contraceptive) is a new thing in the body, they must experience such things…*" (IDI, female, Nurse).

Focus group discussants highlighted many reasons why women in their reproductive age group used injectable contraceptives, including desire to stay attractive in order to prevent husbands from 'straying', space the children and enjoy life with fewer financial and parenting constraints. One of the discussants who currently uses injectable contraceptives described her motivation thus:

> *"…If we space our children well, we will enjoy our lives because our husbands will always desire us since we are always looking attractive. He will not stay out late because he will be thinking of you at home*" (FGD, female, current user).

Also, a participant stated that the impetus for contraceptive use was the fact that spacing her children afforded her the ability to take care of herself and the home thereby promoting hygiene and healthy living in the home. She said:

> "*…You will be able to take care of yourself as well as your house, your husband will also desire you as you will look good as well as your children. Flies will not follow you around and anyone that comes to your house will be able to eat and drink because everywhere is clean and attractive"* (FGD, female, current user).

3.3 Perceptions regarding the community-based distribution (CBD) approach

During the FGDs, women who were current users of injectable contraceptives stated their preference for the community-based approach over the facility-based system of distributing the contraceptives. Some of their reasons for this position included convenience, affordability (saves cost as transport fare is not needed), improved privacy and focused attention from the health worker.

For peer review only - http://bmjopen.bmj.com/site/about/guidelines.xhtml

"*…During home visit, it's between you and the woman (health worker) and she can explain it very well to you since she is not in a hurry; although in the hospital they can also explain but the home visit is better since the health worker will explain until you understand.*" (FGD, female, current user).

Likewise, some male discussants preferred the community-based approach because they believed it was more confidential and gave women more time to deal with domestic issues. The CBD approach was also believed to align with men's desire to be in control of 'family issues'.

"…Maybe the time the wife wants to go (to the health facility) the husband needed her for something else…Sometimes some husbands want all these things to be confidential, but when you now tell them to release their wives to the hospital, they will think you are exposing the family issues" (FGD, married men).

However, one of the discussants who favoured the facility-based approach *'prefer[ed] to go to the hospital, so I can be checked properly, including to check my BP*" (FGD, female, current user). Similarly, some married men also preferred the facility-based approach as they believed that the community-based method is intrusive of their privacy and undermines their authority:

"…My advice is that the woman should come to hospital …because some husbands will query you that who gave you the permission to see their wives (at home) and advise them about child spacing. Some disagree with the community-based method" (FGD, married men).

3.4 Spousal involvement in family planning

In addition, male involvement was highlighted as important if uptake of innovation will be meaningful since Gombe is largely a society where the women need the men's permission for doing many things including obtaining injectable contraceptives.

"*Some men will not accept it (contraceptive) because they want plenty children…she (the wife) should continue begging the husband until he agrees if she wants to use contraceptives*" (FGD, women not using contraceptives).

Furthermore, cultural beliefs and sociocultural context were reported as fuelling poor awareness and knowledge about contraceptive use. Also, male involvement was described as

For peer review only - http://bmjopen.bmj.com/site/about/guidelines.xhtml

important in dealing with the problem of ignorance and poor perception about family planning in the community. According to the participants in this study, once the men are well informed, it is easier to get their buy-in and cooperation in permitting their wives to get the injections.

"*…some of the men don't know much about family planning commodities and some (people) are telling them some of the side effects. So, they prevent the women from using them…that's why we need more sensitization to the men and the youths*" (IDI, female, Nurse).

"The perception of men (about contraceptives) is lower than that of women. The women know the implications better than the men. The men in the rural areas don't mind having any number of children..." (FGD, married men)

Also, a female health worker emphasised the importance of male involvement particularly in communities where the literacy rate is low:

"Honestly. we need to do more in carrying them [men] along. Male involvement is very important because you know they are our superiors. We in the North, it is only the educated that may be sometimes at loggerhead (with their husbands) … but these illiterates and native people, it is always the man that is superior. So, it is very important for us to consider male involvement, it will help a lot (IDI, female, Nurse).

3.5 Community support for scale up

The issue of community support for scale up was solely explored with community and religious leaders. The traditional leaders interviewed believed that community support for the innovation is very high.

"*In fact, the support our community gives to this programme, without exaggeration, I will say maybe 80-90%. They (the community) have been very supportive of the programme*" (IDI, male, Traditional ruler, Yemaltu-Deba).

"*Definitely, you have 70% support from the communities. From the religious leaders, traditional rulers and even of ward heads in the community we get 100% support"* (IDI, male, Traditional leader, Gombe).

However, some cautioned that the community support is not as high as expected due to what

For peer review only - http://bmjopen.bmj.com/site/about/guidelines.xhtml

they call low knowledge and awareness about contraceptive choices in Northern Nigeria.

> "*…Really, people have supported the program but I know some people do not have the knowledge (about injectable contraceptives) but those that have the knowledge are really in support of the program…* [you know] *the Northern culture prohibits some things, that's why I said the support for it (injectable contraceptives scale up) is not as much as we expect*." (IDI, male, Christian religious leader)

One community leader described his role as active in the scale up process because he helps in disseminating information to all the wards under his domain. He shared his experience:

> "*Whenever the need arises, we normally call the kingmakers, district head kingmakers, they are about seven, and we have at least 54 ward heads, we use to call them from time to time and enlighten them on the programme. In fact, almost all the community leaders are fully aware of the programme*" (IDI, male, Traditional leader).

Many of the leaders ascribed the progress with the scale up in their communities to the involvement of the traditional leaders in the programme. However, some leaders complained of not being officially involved in the programme, although they still offer their support.

> "*There are so many traditional rulers that were involved in this program; that is the reason why it has been accepted 100%, especially in Gombe State now*" (IDI, male, Traditional leader, Gombe).

> "*…My involvement is…passive involvement. Of course, we preachers, you can push people, encourage them in your own little way... We preach it, we tell people about it…* (IDI, male, Christian religious leader)

4. Discussion

In our study, there were a number of sociocultural issues alluded to as factors that may affect scale up of a contraceptive innovation in Nigeria, including patriarchy, traditional and religious norms, beliefs and myths about contraceptives and family planning. Agunbiade & Ogunleye[39] reported in their study assessing the sociocultural barriers to the scale up of exclusive breastfeeding in Nigeria, the scale up process cannot be separated from the context and the culture in the environment. Further, in a study among African American women, culture and health beliefs were found to be significant predictors of health innovation

For peer review only - http://bmjopen.bmj.com/site/about/guidelines.xhtml

uptake.[40]

Our study indicates it is vital to secure the understanding and support of the men and significant others for scale up of the community-based distribution of injectable contraceptives to succeed in environments like our study site that are still largely patriarchal.[39] Similar to the findings of our study, male involvement and support of women's significant others, particularly the mothers-in-law, have been reported to be crucial to innovation scale up in Nigeria as these significant others have strong influence on choices made by women.[39] In other settings, increased male participation through increasing men's knowledge and reducing their opposition has been shown to improve uptake and continued use of contraceptives[41-43] and enhancement of prevention of mother to child transmission of HIV (PMTCT) scale up processes.[44] In this study, one of the reasons why women reportedly use contraceptives was to look attractive and satisfy their husbands in order to prevent them from engaging in extramarital relationships. Thus, contraception is both liberating for women on one hand but also perhaps serving the very patriarchy they are trapped in on the other. Studies have shown that in many African societies, patriarchy is perpetuated by the socialisation process which starts from the family and is often preserved by women themselves.[45 46]

Also, findings from our study show passive resistance to contraceptive uptake, particularly at the individual levels, by both women and their spouses. Research has shown that passive resistance is one of the major reasons for the high failure rate of innovation scale up.[47 48] Passive resistance often results from individuals' resistance to change and contentment with or lack of power to change the status quo.[48] Further, consumers will not adopt an innovation if they are not sure of its value or benefits.[49] According to Heidenreich and Kraemer, passive innovation resistance should be addressed before the introduction of new innovations into the society.[47] Strategies for reducing passive resistance to uptake of health innovations in the community include actively involving men in their uptake,[14] as well as giving adequate and correct information about the advantages of contraceptive use to all stakeholders - health workers, community and religious leaders, trained volunteers and program managers.[49]

Since the passive resistance reported in this study is largely underpinned by deep-seated issues of patriarchy, religious beliefs and traditional norms/values, it is imperative for policy makers and program managers to engage men – who are custodians, in most cases, of both religious and cultural values.[50 51] Male involvement can bring benefits for all community

For peer review only - http://bmjopen.bmj.com/site/about/guidelines.xhtml

stakeholders,[50] as well as social change.[52] With careful management and ongoing monitoring, the involvement of men may ultimately give the scale up of this health innovation a greater chance of success.

Furthermore, religious beliefs were also found to influence scale up of the community-based injectable contraceptives and family planning in general in our study sites in Northern Nigeria. Religion has been shown to significantly influence decisions on contraceptive use,[53] as well as individual/household choices, lifestyle and even traditional values.[54] Thus, religion has great implications for the social marketing of commodities particularly contraceptives, as uptake of health products has been shown to differ significantly among adherents of major religions.[54] For example, research done in Islamic countries[55 56] highlights the need to understand Islamic religion in order to do effective social marketing, and recommends that approval be obtained from religious leadership before certain sensitive products like contraceptives are marketed. Understanding religious nuances in local contexts is important in multi-faith countries such as Nigeria – for example, what 'works' in the largely-Islamic North[57] may need adaptation for Christian populations elsewhere.

Our study demonstrated the importance of strong community support and partnership with community gate-keepers in the scale up process. Research has shown that the CBD approach cannot succeed without community participation.[58] Therefore, programme implementers need to learn from the community what works (or may work) in the setting, what distribution method the community prefers or what modification to the existing methods they favour. Research has shown that there is no quick fix to scaling up.[58] It is essentially a learning process which is unique in different contexts. Therefore, scale up may not be effective without adequate contextual knowledge and willingness to learn through the process of community participation and stakeholder collaboration.[58 59]

While the focus of this paper has been on challenges as identified in the wider context of religion and patriarchy, many of the suggested solutions rely on the health systems and those working in it, to navigate these challenges and overcome them. However, this cannot be a static or reactive approach but rather needs to recognise that health systems and those working in them are also part of the sociocultural context and therefore it is important to find ways to support healthcare workers in mediating, translating and promoting the innovation in this context.

For peer review only - http://bmjopen.bmj.com/site/about/guidelines.xhtml

4.1 Limitations of the study

Some limitations of this study should be emphasised. As with any qualitative study, the findings are not statistically generalizable but the theories and ideas can be applied to other contexts. Also, some of the participants in this study were involved in the community-based distribution intervention, therefore they may provide more socially desirable answers. However, a strength of this study is that it provided stakeholders who participated a distinct experience and some observations to reflect upon since the study was based on a current program, rather than speaking merely about theoretical responses they or their community members may have to a health intervention. Thus, the findings of this research provide valuable information into how sociocultural context and user support affect the scale up of health innovations in LMICs, particularly in a patriarchal society like Nigeria.

4.3 Conclusion

Our study suggests the need for continuous community education and engagement with the significant others and community leadership structures – both traditional and religious. Thus, providing an innovation does not necessarily translate to uptake. Therefore, program implementers should also consider sociocultural and health system contexts when packaging a health innovation. Hence, supporting those in the health system to mediate the innovation in ways that are sensitive to the context but which, by working with communities and others, promotes transformation is imperative. In addition, incorporating the CBD approach with other outreach programmes, developing a more effective supervision of the approach may help make CBD more productive. Interventions to improve male participation will likewise go a long way to improve the scale up process. Policy implementers should also see scale up as a learning process and be willing to move at the speed of the community.

Acknowledgements

We are grateful to Drs Mariya Saleh, Wole Adefalu, Edward Oladele and Hadiza Kamofu for expediting access to key informants. Also, we appreciate Dr Alex Eze for his feedback on the early draft of the manuscript. We are grateful for the support of Dr Busola Adebayo, Jimi Latunji and Wunmi Folajimi-Senjobi with editing the manuscript. We would like to thank all the participants in this study.

Authors' contributions

OA conceived the study; OA and MK designed it. The data was collected by OA. OA, MK and

For peer review only - http://bmjopen.bmj.com/site/about/guidelines.xhtml

BH drafted and commented on the draft of the manuscript. OA, MK and BH read and approved the final version of the manuscript.

Data Availability Statement

Dataset for this study is available at https://doi.org/10.5061/dryad.8kprr4xj4.

For peer review only

For peer review only - http://bmjopen.bmj.com/site/about/guidelines.xhtml

References

1. Subramanian S, Naimoli J, Matsubayashi T, et al. Do we have the right models for scaling up health services to achieve the Millennium Development Goals? *BMC health services research* 2011;11(1):336.
2. Manham L, Hanson K. Scaling up in international health: what are the key issues? Review. *Health Policy Plan* 2010;25:85-96.
3. Bradley EH, Curry LA, Taylor LA, et al. A model for scale up of family health innovations in low-income and middle-income settings: a mixed methods study. *BMJ open* 2012;2(4):e000987.
4. Hecht R, Stover J, Bollinger L, et al. Financing of HIV/AIDS programme scale-up in low-income and middle-income countries, 2009–31. *The Lancet* 2010;376(9748):1254-60.
5. Johns B, Torres TT. Costs of scaling up health interventions: a systematic review. *Health policy and planning* 2005;20(1):1-13.
6. Mangham LJ, Hanson K. Scaling up in international health: what are the key issues? *Health policy and planning* 2010;25(2):85-96.
7. Rajaram SS, Rashidi A. Asian-Islamic women and breast cancer screening: a socio-cultural analysis. *Women & health* 1999;28(3):45-58.
8. Westercamp N, Bailey RC. Acceptability of Male Circumcision for Prevention of HIV/AIDS in Sub-Saharan Africa: A Review. *AIDS and Behavior* 2007;11(3):341-55. doi: 10.1007/s10461-006-9169-4
9. Katapodi MC, Lee KA, Facione NC, et al. Predictors of perceived breast cancer risk and the relation between perceived risk and breast cancer screening: a meta-analytic review. *Preventive medicine* 2004;38(4):388-402.
10. Muanda M, Ndongo PG, Taub LD, et al. Barriers to modern contraceptive use in Kinshasa, DRC. *PloS one* 2016;11(12):e0167560.
11. Muanda MF, Ndongo GP, Messina LJ, et al. Barriers to modern contraceptive use in rural areas in DRC. *Culture, health & sexuality* 2017;19(9):1011-23.
12. Airhihenbuwa CO. Health and culture: Beyond the Western paradigm: Sage 1995.
13. Colleran H, Mace R. Social network-and community-level influences on contraceptive use: evidence from rural Poland. *Proc R Soc B* 2015;282(1807):20150398.
14. Dalaba MA, Stone AE, Krumholz AR, et al. A qualitative analysis of the effect of a community-based primary health care programme on reproductive preferences and contraceptive use among the Kassena-Nankana of northern Ghana. *BMC health services research* 2016;16(1):80.
15. Etukudo IW. Spousal approval, communication and contraceptive behaviour in rural Nigeria. *African Journal of Midwifery and Women's Health* 2015;9(4):170-76.
16. Prata N, Gessessew A, Cartwright A, et al. Provision of injectable contraceptives in Ethiopia through community-based reproductive health agents. *Bulletin of the World Health Organization* 2011;89:556-64.
17. Prata N, Vahidnia F, Potts M, et al. Revisiting community-based distribution programs: are they still needed? *Contraception* 2005;72(6):402-07.
18. Gichane MW, Mutesa M, Chowa G. Translating Evidence into Policy Change: Advocacy for Community-Based Distribution of Injectable Contraceptives in Zambia. *Global Social Welfare* 2019;6(1):41-47.
19. Hoke T, Brunie A, Krueger K, et al. Community-based distribution of injectable contraceptives: introduction strategies in four sub-Saharan African countries. *International perspectives on sexual and reproductive health* 2012;38(4):214-19.
20. FHI. The Effectiveness of Community-Based Access to Injectable Contraceptives in Nigeria: A Technical Report. 2010

For peer review only - http://bmjopen.bmj.com/site/about/guidelines.xhtml

21. Association for Reproductive and Family Health. Increasing access to contraceptives in Nigeria through task-shifting to Community Health Extension Workers (CHEWs) Abuja: Association for Reproductive and Family Health; 2015 [cited 2015 6 January]. Available from: http://www.arfh-ng.org/news/item012.php accessed 6 January 2015.
22. Association for Reproductive and Family Health. Increasing Access to Contraceptives in Nigeria Through Task-shifting to Community Health Extension Workers (CHEWS) Ibadan: Association for Reproductive and Family Health,; 2014 [cited 2015 5 November]. Available from: http://arfh-ng.org/increasing-access-contraceptives-nigeria-task-shifting-community-health-extension-workers-chews/ accessed 5 November 2015.
23. Akinyemi O, Harris B, Kawonga M. Health system readiness for innovation scale-up: the experience of community-based distribution of injectable contraceptives in Nigeria. *BMC Health Services Research* 2019;19(1):1-11.
24. Akinyemi O, Harris B, Kawonga M. Innovation diffusion: how homogenous networks influence the uptake of community-based injectable contraceptives. *BMC public health* 2019;19(1):1520.
25. National Population Commission. Population and housing census of the Federal Republic of Nigeria. *Priority tables* 2006;1
26. National Population Commission. Nigeria demographic and health survey 2008. *Nigeria and ICF Macro* 2008
27. National Population Commission, ICF International. Nigeria Demographic and Health Survey 2013. *Abuja, Nigeria* 2014
28. Vonk ME, Tripodi T, Epstein I. Research techniques for clinical social workers: Columbia University Press 2007.
29. Pope C, Mays N. Qualitative research: reaching the parts other methods cannot reach: an introduction to qualitative methods in health and health services research. *Bmj* 1995;311(6996):42-45.
30. Temple B, Edwards R. Interpreters/translators and cross-cultural research: Reflexivity and border crossing. *International Journal of Qualitative Methods* 2002;1(2)
31. Guest G, Bunce A, Johnson L. How many interviews are enough? An experiment with data saturation and variability. *Field methods* 2006;18(1):59-82.
32. Namey E, Guest G, McKenna K, et al. Evaluating bang for the buck: a cost-effectiveness comparison between individual interviews and focus groups based on thematic saturation levels. *American Journal of Evaluation* 2016;37(3):425-40.
33. Guest G, MacQueen KM, Namey EE. Introduction to applied thematic analysis. *Applied thematic analysis* 2012;3:20.
34. Bernard HR. Research methods in anthropology: Qualitative and quantitative approaches: Rowman & Littlefield 2017.
35. Hutchison AJ, Johnston LH, Breckon JD. Using QSR-NVivo to facilitate the development of a grounded theory project: an account of a worked example. *International journal of social research methodology* 2010;13(4):283-302.
36. Van Nes F, Abma T, Jonsson H, et al. Language differences in qualitative research: is meaning lost in translation? *European journal of ageing* 2010;7(4):313-16.
37. O'Brien BC, Harris IB, Beckman TJ, et al. Standards for reporting qualitative research: a synthesis of recommendations. *Academic Medicine* 2014;89(9):1245-51.
38. Johnson TP. Snowball sampling: introduction. *Wiley StatsRef: Statistics Reference Online* 2014
39. Agunbiade OM, Ogunleye OV. Constraints to exclusive breastfeeding practice among breastfeeding mothers in Southwest Nigeria: implications for scaling up. *International breastfeeding journal* 2012;7(1):5.

For peer review only - http://bmjopen.bmj.com/site/about/guidelines.xhtml

40. Sociocultural context of mammography screening use. Oncology nursing forum; 2006. Oncology Nursing Society.
41. Kabagenyi A, Jennings L, Reid A, et al. Barriers to male involvement in contraceptive uptake and reproductive health services: a qualitative study of men and women's perceptions in two rural districts in Uganda. *Reproductive health* 2014;11(1):21.
42. Ujuju C, Anyanti J, Adebayo S, et al. Religion, culture and male involvement in the use of the Standard Days Method: evidence from Enugu and Katsina states of Nigeria. *International nursing review* 2011;58(4):484-90.
43. Sanusi AA, Akinyemi OO, Onoviran OO. Do knowledge and cultural perceptions of modern female contraceptives predict male involvement in Ayete, Nigeria? *African journal of reproductive health* 2014;18(4):105-14.
44. Peltzer K, Jones D, Weiss SM, et al. Promoting male involvement to improve PMTCT uptake and reduce antenatal HIV infection: a cluster randomized controlled trial protocol. *BMC Public Health* 2011;11(1):778. doi: 10.1186/1471-2458-11-778
45. Florence N. Bukusu (Kenya) folktales: how women perpetuate patriarchy. *International Feminist Journal of Politics* 2013;15(3):370-90.
46. Kambarami M. Femininity, sexuality and culture: Patriarchy and female subordination in Zimbabwe. *South Africa: ARSRC* 2006
47. Heidenreich S, Kraemer T. Innovations—doomed to fail? Investigating strategies to overcome passive innovation resistance. *Journal of Product Innovation Management* 2016;33(3):277-97.
48. Heidenreich S, Spieth P. Why innovations fail—The case of passive and active innovation resistance. *International Journal of Innovation Management* 2013;17(05):1350021.
49. KISS Communications. Why Consumers Resist Innovation – part one 2016 [cited 2018 1 September]. Available from: https://www.kisscom.co.uk/news/why-consumers-resist-innovation-part-one accessed 1 September 2018.
50. Orji E, Onwudiegwu U. Contraceptive practice among married market men in Nigeria. *East African medical journal* 2003;80(7):357-60.
51. Orji E, Onwudiegwu U. Contraceptive practice among married men in Nigeria. *East Afr Med J* 2008;200(80):7.
52. Onwukwe LC, Njemanze VC, Ebeh R, et al. Religiosity and Sexism as Indices of People's Perception of Women in Politics in Nigeria. *American Journal of Social Science Research* 2017;3(2):9-16.
53. Mitike G. Community-based distribution of family planning as perceived by people in the reproductive age group, North and South Gondar zones, Ethiopia. *Ethiopian Journal of Health Development* 2000;14(1):31-42.
54. Shyan Fam K, Waller DS, Zafer Erdogan B. The influence of religion on attitudes towards the advertising of controversial products. *European Journal of marketing* 2004;38(5/6):537-55.
55. Michell P, Al-Mossawi M. Religious commitment related to message contentiousness. *International Journal of Advertising* 1999;18(4):427-43.
56. Luqmani M, Yavas U, Quraeshi Z. Advertising in Saudi Arabia: content and regulation. *International Marketing Review* 1989;6(1)
57. Watts MJ. Silent violence: Food, famine, and peasantry in northern Nigeria: University of Georgia Press 2013.
58. Gilson L, Schneider H. Commentary: Managing scaling up: what are the key issues? *Health Policy and Planning* 2010;25(2):97-98.
59. Phillips JF, Greene WL. Community based distribution of family planning in Africa: lessons from operations research. 1993

For peer review only - http://bmjopen.bmj.com/site/about/guidelines.xhtml

Table

Table 1: Participants' sociodemographic characteristics and data collection methods across study sites

Location	Type	No. of interviews/ FGDs	No. of participants	Gender M n (%)	Gender F n (%)	Participant type
**Federal and State Level**						
	KII	4	4	2 (50.0)	2 (50.0)	NGO program manager, Senior official at the Federal MoH[a], NGO program managers
**Gombe State**						
	KII	3	3	2 (66.7)	1 (33.3)	Senior officials of State MoH.
LGA **A**	IDI	7	7	3 (42.9)	4 (57.1)	Health workers (Nurses, CHEWs[b]), traditional and religious leaders
	FGD	5	40	16 (40.0)	24 (60.0)	Current contraceptive users, older women non-users, younger women non-users (in reproductive age group), married and single men
LGA **B**	IDI	8	8	4 (50.0)	4 (50.0)	Health workers (Nurses, CHEWs[b]), traditional and religious leaders
	FGD	5	40	16 (40.0)	24 (60.0)	Current contraceptive users, older women non-users, younger women non-users (in reproductive age group), married and single men
**Total**		**32**	**102**	**43 (42.2)**	**59 (57.8)**	

[a]MoH – Ministry of Health; [b]CHEWs – Community Health Extension Workers

For peer review only - http://bmjopen.bmj.com/site/about/guidelines.xhtml

Figure legends/captions

Figure 1: Situating data collection within the timeline for pilot study and scale up

Figure 2: Socio-cultural factors influencing uptake of CBD of injectable contraceptives in Gombe State

For peer review only

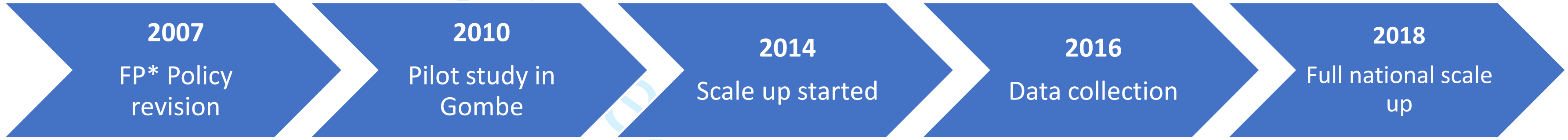

*Family planning

Figure 1: Situating data collection within the timeline for pilot study and scale up

For peer review only

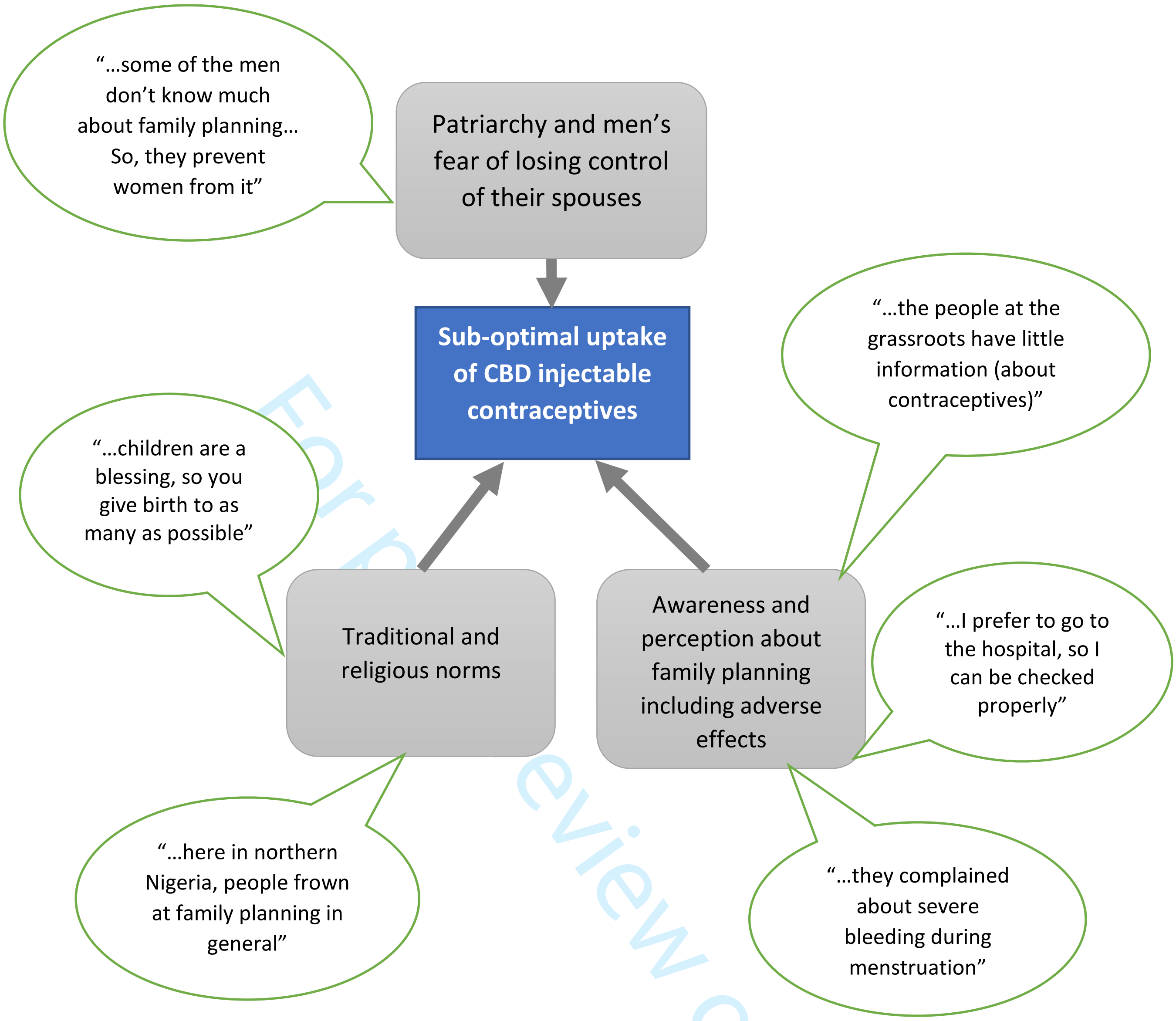

Figure 2: Sociocultural factors influencing uptake of CBD of injectable contraceptives in Gombe State

For peer review only

SUPPLEMENTARY FILE 1

Interview Guide: Key informant interview with senior MOH officials and NGO programme managers

1. Let's start by you describing your role in implementing this intervention – the community-based delivery of injectable contraceptives in the pilot sites in Gombe. What was your role and how long were you involved?

2. Did you encounter any challenges during the pilot study? Please explain.
   Probes:
   - If not mentioned, ask: any resistance from the community members, from health workers, from other stakeholders, from potential users themselves?
   - How did you handle challenges / resistance?

3. We are interested in your experience with the process of taking the intervention from pilot study phase to wider implementation in Gombe itself. What was the process, from implementation to wider scale up of the intervention? Walk me through the process.
   Probes:
   - The goal, approach to implementing, key components of the process, indicators of success
   - Landscape and stakeholder assessment done – before, during or after the pilot?
   - Your role in these processes?

4. In your opinion, what factors have supported the scale up of this intervention so far?

5. Have you experienced challenges during the process of scaling up? Please explain.
   Probes:
   - What challenges? ... ***may prompt with*** "any others"
   - How did you handle challenges?

6. Was there a way of ensuring that the intervention aligns to the wants and preferences of the users you were targeting? Please tell me about it.
   Probes:
   - Any user needs / wants assessment done? By whom? When?
   - Any modifications made in order to align packaging of the innovation to user wants – either during or after the pilot?
   - Any health service modification (e.g. change service models for delivering the intervention)?

7. Have communities been engaged in the scale up process? Please describe in what ways and by whom?

8. Looking back, is there anything that you think could have been done differently to facilitate the process from pilot study to wider implementation? Please explain.

9. Is there anything else we have not asked but you would like to tell us about your experience with the process of implementation and scale-up of CBD?

Thank you for your time. This is the end of our discussion.

For peer review only

In-depth Interview Guides

A. Interview guide: In-depth interview with health workers (Doctors and CHEWs)

1. Let's start by you describing your role in implementing this intervention – the community-based delivery of injectable contraceptives by community health extension workers.
    a. What role did doctors / CHEWs play in designing the intervention?
    b. What about their role in introducing the intervention at community level?
    c. What was your own role?
    Probes:
    - **Doctors:** Your own (or experience of other doctors like you) with: planning, training CHEWs, providing supportive supervision to CHEWs during and after the pilot?
    - **CHEWs**: Your own (or experience of other CHEWs like you) in planning, administering the injectable contraceptives to women?

    d. Did you / other health workers experience challenges?
        i. Please explain.
        ii. How did you handle the challenges?

2. In your opinion, what factors affect wider implementation and uptake of the CBD of injectable contraceptives in the communities that you serve?
    Probes:
    - Anything else?

3. In your opinion, how supportive of this innovation are:
    a. **For doctors**: doctors in general, and your professional health group (e.g. Nigerian Medical Association) please explain.
    b. **For CHEWs**: community health extension workers in general? Please explain.

4. As far as you are aware, what role, if any, have health workers like you played in making the CBD of injectable contraceptives more acceptable to the users in your community? Please explain.

5. In your opinion, how likely are those women who have already adopted the CBD of injectables to spread information about the innovation to other women who are not using it? Please explain.

6. Looking back, is there anything that you feel might have been done differently to facilitate the process of moving from pilot study to wider implementation?

7. Is there anything else we have not discussed that you would like to tell us about your experience with implementing the CBD of injectable contraceptives?

Thank you for your time. This is the end of our discussion.

For peer review only

B. Interview guide: In-depth interview with community leaders

1. You were selected to participate in this study because you are aware of the government program in which CHEWs provide injectable contraceptives to women in the community, outside the health clinic setting.
    a. Please describe what you know about this program.
    b. How did you hear about it?

2. How much support do you think there is for this program in your community? Please explain

3. Have you or other community leaders like you been involved in introducing this program on injectable contraceptives into your community?
    a. ***If yes***: Describe your / or other leaders' involvement (what role)? How did you / they get involved? Who initiated?
    b. How do you feel about being involved / not being involved?

4. The government started with this program only in two places in Gombe but they are now trying to make this available to all other parts of Gombe.
    a. What challenges do you think the government might encounter when trying to get wider uptake of this programme in the community?
    b. Have you or other community leaders in this community been approached by government to assist with addressing these challenges?

5. In your opinion, what might prevent women in your community from taking up this intervention? What may encourage them to take it up?

6. In your opinion, how likely are those women who have already adopted the CBD of injectables to share information about it to other women who are not using it? Please explain?

7. Looking back, is there anything that you feel might have been done differently to make this way of providing contraceptives acceptable in your community?

8. Is there anything else we have not discussed that you would like to tell us about your experience with implementing the CBD of injectable contraceptives?

Thank you for your time. This is the end of our discussion.

For peer review only

Focus Group Discussion Guides

A. Focus group discussion guide: FGDs with women who have already adopted the use of injectable contraceptives through the CBD approach

1. Let us start with talking about contraceptives / family planning methods in general. Please share with me what you understand about contraception / family planning. What kinds of contraceptive methods do you know?

2. What do you think people in this community think about contraceptives and family planning in general?

   Probe:
   - Do they support the idea of contraception / family planning in general?
   - Specifically, how supportive of injectable contraceptives compared to others?

3. What about women in this community. How do you think they feel about contraceptives and family planning in general?

   Probe:
   - Support the use of contraception / family planning in general?
   - Specifically, how popular are injectable contraceptives compared to others?
   - What makes women use injectable contraceptives? What makes them not use it?

4. I am interested to know what you think women want and prefer regarding contraceptives. Which types of contraceptives do women in your community prefer? How do you think women prefer to receive contraceptives (from clinic versus other places; from doctor or nurse versus others)?

5. The government introduced a new program where community health extension workers now administer injectable contraceptives to women away from the clinic, including at homes. I know that you already receive injectable contraceptives through this approach.

   a. How did you hear about this program?

   b. What made women like you to decide to receive injectables through CHEWs?

   c. Would you encourage friends or family members to receive injectable contraceptives from CHEWs? Please explain.

   d. What do you think might prevent other women in your community from using this new way of receiving injectable contraceptives through CHEWs?

6. What suggestions do you have for how the way injectable contraceptives are administered in your community can be improved to make sure many more women receive them?

Thank you for your time. This is the end of our discussion

For peer review only

B. Focus group discussion guide: FGD with a group of women in the reproductive age group who have not yet adopted the CBD of injectables (may include those who do not use injectables and those who do)

1. Let us start with talking about contraceptives / family planning methods in general. How many contraceptive types do you know? Can you describe them?

2. How do you think people in this community perceive contraceptives and family planning in general?

   Probe:
   - Popularity of contraceptives / the idea of family planning in general
   - Specifically, popularity of injectable contraceptives

3. How do you think women in this community perceive contraceptives and family planning in general?

   Probe:
   - Popularity of contraceptives / family planning in general
   - Specifically, popularity of injectable contraceptives

4. What do women want - which types of contraceptives, which mode of delivery do they prefer? (clinic vs. other places, doctor/nurse versus others)

5. Let us now talk about injectable contraceptives specifically. In your opinion, what factors will make women of reproductive age use or reject injectable contraceptives? What makes them not use it?

6. The government introduced a new program where community health extension workers now administer injectable contraceptives to women away from the clinic, including at homes. I know that you have not received injectable contraceptives through this approach.
   a. How did you hear about this program?
   b. What factors make women like you decide not to receive injectables through CHEWs?
   c. What factors would encourage you to receive the injectable contraceptives through CHEWs?
   d. Willingness to encourage friends and family members of reproductive age to receive injectable contraceptives from CHEWs?
   e. What do you think might prevent other women in your community from using this new way of receiving injectable contraceptives through CHEWs?

   Probes:
   - Resistance to mode of distribution – facility vs. community based
   - Socio-cultural resistance to injectable contraceptives
   - Resistance to injectable contraceptives being administered by CHEWs rather than other health workers like nurses and doctors

7. What suggestions do you have for how the way injectable contraceptives are administered in your community can be improved to make sure many more women like you receive them?

Thank you for your time. This is the end of our discussion.

For peer review only

C. Focus group discussion guide: FGD with elderly women (older than reproductive age) in the community

1. Let us start with talking about contraceptives / family planning methods in general. How many contraceptive types do you know? Can you describe them?

2. How do you think people in this community perceive contraceptives and family planning in general?
   Probe:
   - Popularity of contraceptives / the idea of family planning in general
   - Specifically, popularity of injectable contraceptives
3. What role do you play in advising your married daughters/daughters-in-law and family members of reproductive age about contraception/family planning?

4. How do you think elderly women (grandmothers) in this community perceive contraceptives and family planning in general?
   Probe:
   - Popularity of contraceptives / family planning in general among elderly women (awareness and knowledge, not use)
   - Specifically, popularity of injectable contraceptives

5. What do elderly women prefer? Which types of contraceptives, mode of delivery (clinic vs. other places, doctor/nurse versus others) do they prefer for their married daughters/daughters-in-law

6. Let us now talk about injectable contraceptives specifically. In your opinion, what factors will make women of reproductive age use or reject injectable contraceptives?
7. Will you support your married daughters/daughters-in-law using injectable contraceptives? Please give reasons for your answer.
8. The government introduced a new program where community health extension workers now administer injectable contraceptives to women away from the clinic, including at homes.

   a. How did you hear about this program? In your opinion, what factors help women to decide to receive injectables through CHEWs?
   b. Willingness to encourage married daughters/daughters-in-law and family members of reproductive age to receive injectable contraceptives from CHEWs?
   c. What do you think might prevent women in your community from using injectable contraceptives through CHEWs?
   d. What kinds of challenges do you know the government has faced with the distribution of injectable contraceptives through CHEWs in your community?

   Probes:
   - Resistance to mode of distribution – facility vs. community based
   - Socio-cultural resistance to injectable contraceptives
   - Resistance to injectable contraceptives being administered by CHEWs rather than other health workers like nurses and doctors

8. What suggestions do you have for how the way injectable contraceptives are administered in your community can be improved to make sure many more women receive them?

Thank you for your time. This is the end of our discussion.

For peer review only

D. Focus group discussion guide: FGD with younger and older men (single and married) in the community

1. Let us start with talking about contraceptives / family planning methods in general. How many contraceptive types do you know? Can you describe them?

2. How do you think people in this community perceive contraceptives and family planning in general?
   Probe:
   - Popularity of contraceptives / the idea of family planning in general
   - Specifically, popularity of injectable contraceptives

3. How do you think men in this community perceive contraceptives and family planning in general?
   Probe:
   - Popularity of contraceptives / family planning in general among men
   - Specifically, popularity of injectable contraceptives

4. What do men want - which types of contraceptives, mode of delivery (clinic vs. other places, doctor/nurse versus others) do they prefer for their wives
5. Let us now talk about injectable contraceptives specifically. In your opinion, what factors will make women of reproductive age use or reject injectable contraceptives?
6. Will you support your wife/partner/girlfriend using injectable contraceptives? Please give reasons for your answer.
7. The government introduced a new program where community health extension workers now administer injectable contraceptives to women away from the clinic, including at homes.

   a. How did you hear about this program? In your opinion, what factors help women to decide to receive injectables through CHEWs?
   b. Willingness to encourage wife/partner/girlfriend and family members of reproductive age to receive injectable contraceptives from CHEWs?
   c. What do you think might prevent women in your community from using injectable contraceptives through CHEWs?
   d. What kinds of challenges do you know the government has faced with the distribution of injectable contraceptives through CHEWs in your community?

   Probes:
   - Resistance to mode of distribution – facility vs. community based
   - Socio-cultural resistance to injectable contraceptives
   - Resistance to injectable contraceptives being administered by CHEWs rather than other health workers like nurses and doctors

9. What suggestions do you have for how the way injectable contraceptives are administered in your community can be improved to make sure many more women receive them?

Thank you for your time. This is the end of our discussion.

For peer review only

SUPPLEMENTARY FILE 2

Participant Information Sheet for MOH officials

Study title: Scale up of community-based injectable contraceptives in Gombe State, Nigeria

Introduction:

Good day! I am Oluwaseun Akinyemi, a PhD candidate of the School of Public Health, University of the Witwatersrand, Johannesburg. I am doing research on the scale up of community-based injectable contraceptives in Gombe State, Nigeria. Research is just the process to learn the answer to a question. In this study we want to learn what the factors are that may influence the scale up of community-based distribution of injectable contraceptives in Nigeria, in order to understand possible facilitators and barriers.

Invitation to participate: I am therefore asking / inviting you to take part in this research study because you are a senior MOH official who was/is involved with this programme.

What is involved in the study – I am asking different people such as policy makers, program managers, health workers, community leaders, women who are the end-users of injectable contraceptives, and broader members of the community to participate in this study. About 100 people will participate in the study and all will be from Nigeria. If you agree to participate it means you will take part in a key informant interview. Each interview will last for about 60 minutes. In the interviews I will ask questions about your knowledge and your role in the implementation or use of injectable contraceptives as well as what you think might be the challenges and things that work well in implementing this program. There are no right and wrong answers; I am interested in understanding your opinions. Please note that I intend to audio-tape this interview.

Risks and Benefits of being involved in the study – There are no invasive procedures involved in this study, thus no physical harm is envisaged. However, you don't have to answer any question you are not comfortable with.

There are no direct benefits for participating in this study.

The participant will be given pertinent information on the study while involved in the project and after the results are available.

Participation is voluntary: Refusal to participate will involve no penalty or loss of benefits to which you are otherwise entitled. Also, you may discontinue participation at any time without penalty or loss of benefits to which you are otherwise entitled.

Reimbursements for transportation: Transportation costs incurred in order to participate in the study will be reimbursed at the rate of ₦100 per km.

For peer review only

Confidentiality: Efforts will be made to keep personal information confidential. Absolute confidentiality cannot be guaranteed. Personal information may be disclosed if required by law.

Organizations that may inspect and/or copy the research records for quality assurance and data analysis include groups such as the Research Ethics Committee. If results are published, may lead to individual / cohort identification.

Contact details of researcher – for further information / reporting of study related adverse events.

Dr Oluwaseun Akinyemi
Department of Health Policy and Management
College of Medicine
University of Ibadan
Ibadan, Nigeria
+234 803 502 0136
seunakinyemi@hotmail.com

Contact details of UI/UCH Ethics Committee chair (Nigeria) – for more information.

Prof. Catherine O. Falade
Chairperson, University of Ibadan/University College Hospital Ethics Committee
Institute for Advanced Medical Research and Training (IAMRAT)
College of Medicine, University of Ibadan
Ibadan, Nigeria
+234 803 326 4593
cfalade@comui.edu.ng

Contact details of REC administrator and chair (South Africa) – for more information.

Ms Zanele Ndlovu
Research Administrator
Human Research Ethics Committee (Medical)
University of the Witwatersrand
Johannesburg, South Africa
+27 11 717 1252
zanele.ndlovu@wits.ac.za

For peer review only

Participant Information Sheet for NGO programme managers

Study title: Scale up of community-based injectable contraceptives in Gombe State, Nigeria

Introduction:

Good day! I am Oluwaseun Akinyemi, a PhD candidate of the School of Public Health, University of the Witwatersrand, Johannesburg. I am doing research on the scale up of community-based injectable contraceptives in Gombe State, Nigeria. Research is just the process to learn the answer to a question. In this study we want to learn what the factors are that may influence the scale up of community-based distribution of injectable contraceptives in Nigeria, in order to understand possible facilitators and barriers.

Invitation to participate: I am therefore asking / inviting you to take part in this research study because you are a programme manager for an NGO involved with this programme.

What is involved in the study – I am asking different people such as policy makers, program managers, health workers, community leaders, women who are the end-users of injectable contraceptives, and broader members of the community to participate in this study. About 100 people will participate in the study and all will be from Nigeria. If you agree to participate it means you will take part in a key informant interview. Each interview will last for about 60 minutes. In the interviews I will ask questions about your knowledge and your role in the implementation or use of injectable contraceptives as well as what you think might be the challenges and things that work well in implementing this program. There are no right and wrong answers; I am interested in understanding your opinions. Please note that I intend to audio-tape this interview.

Risks and Benefits of being involved in the study – There are no invasive procedures involved in this study, thus no physical harm is envisaged. However, you don't have to answer any question you are not comfortable with.

There are no direct benefits for participating in this study.

The participant will be given pertinent information on the study while involved in the project and after the results are available.

Participation is voluntary: Refusal to participate will involve no penalty or loss of benefits to which you are otherwise entitled. Also, you may discontinue participation at any time without penalty or loss of benefits to which you are otherwise entitled.

Reimbursements for transportation: Transportation costs incurred in order to participate in the study will be reimbursed at the rate of ₦100 per km.

Confidentiality: Efforts will be made to keep personal information confidential. Absolute confidentiality cannot be guaranteed. Personal information may be disclosed if required by law.

For peer review only

Organizations that may inspect and/or copy the research records for quality assurance and data analysis include groups such as the Research Ethics Committee. If results are published, may lead to individual / cohort identification.

Contact details of researcher – for further information / reporting of study related adverse events.

Dr Oluwaseun Akinyemi
Department of Health Policy and Management
College of Medicine
University of Ibadan
Ibadan, Nigeria
+234 803 502 0136
seunakinyemi@hotmail.com

Contact details of UI/UCH Ethics Committee chair (Nigeria) – for more information.

Prof. Catherine O. Falade
Chairperson, University of Ibadan/University College Hospital Ethics Committee
Institute for Advanced Medical Research and Training (IAMRAT)
College of Medicine, University of Ibadan
Ibadan, Nigeria
+234 803 326 4593
cfalade@comui.edu.ng

Contact details of REC administrator and chair (South Africa) – for more information.

Ms Zanele Ndlovu
Research Administrator
Human Research Ethics Committee (Medical)
University of the Witwatersrand
Johannesburg, South Africa
+27 11 717 1252
zanele.ndlovu@wits.ac.za

For peer review only

Participant Information Sheet for health workers

Study title: Scale up of community-based injectable contraceptives in Gombe State, Nigeria

Introduction:

Good day! I am Oluwaseun Akinyemi, a PhD candidate of the School of Public Health, University of the Witwatersrand, Johannesburg. I am doing research on the scale up of community-based injectable contraceptives in Gombe State, Nigeria. Research is just the process to learn the answer to a question. In this study we want to learn what the factors are that may influence the scale up of community-based distribution of injectable contraceptives in Nigeria, in order to understand possible facilitators and barriers.

Invitation to participate: I am therefore asking / inviting you to take part in this research study because you are a health worker who is involved with this programme.

What is involved in the study – I am asking different people such as policy makers, program managers, health workers, community leaders, women who are the end-users of injectable contraceptives, and broader members of the community to participate in this study. About 100 people will participate in the study and all will be from Nigeria. If you agree to participate it means you will take part in a key informant interview. Each interview will last for about 60 minutes. In the interviews I will ask questions about your knowledge and your role in the implementation or use of injectable contraceptives as well as what you think might be the challenges and things that work well in implementing this program. There are no right and wrong answers; I am interested in understanding your opinions. Please note that I intend to audio-tape this interview.

Risks and Benefits of being involved in the study – There are no invasive procedures involved in this study, thus no physical harm is envisaged. However, you don't have to answer any question you are not comfortable with.

There are no direct benefits for participating in this study.

The participant will be given pertinent information on the study while involved in the project and after the results are available.

Participation is voluntary: Refusal to participate will involve no penalty or loss of benefits to which you are otherwise entitled. Also, you may discontinue participation at any time without penalty or loss of benefits to which you are otherwise entitled.

Reimbursements for transportation: Transportation costs incurred in order to participate in the study will be reimbursed at the rate of ₦100 per km.

Confidentiality: Efforts will be made to keep personal information confidential. Absolute confidentiality cannot be guaranteed. Personal information may be disclosed if required by law.

Organizations that may inspect and/or copy the research records for quality assurance and data analysis include groups such as the Research Ethics Committee. If results are published, may lead to individual / cohort identification.

Contact details of researcher – for further information / reporting of study related adverse events.

Dr Oluwaseun Akinyemi
Department of Health Policy and Management
College of Medicine
University of Ibadan
Ibadan, Nigeria
+234 803 502 0136
seunakinyemi@hotmail.com

Contact details of UI/UCH Ethics Committee chair (Nigeria) – for more information.

Prof. Catherine O. Falade
Chairperson, University of Ibadan/University College Hospital Ethics Committee
Institute for Advanced Medical Research and Training (IAMRAT)
College of Medicine, University of Ibadan
Ibadan, Nigeria
+234 803 326 4593
cfalade@comui.edu.ng

Contact details of REC administrator and chair (South Africa) – for more information.

Ms Zanele Ndlovu
Research Administrator
Human Research Ethics Committee (Medical)
University of the Witwatersrand
Johannesburg, South Africa
+27 11 717 1252
zanele.ndlovu@wits.ac.za

For peer review only

Participant Information Sheet for community leaders

Study title: Scale up of community-based injectable contraceptives in Gombe State, Nigeria

Introduction:

Good day! I am Oluwaseun Akinyemi, a PhD candidate of the School of Public Health, University of the Witwatersrand, Johannesburg. I am doing research on the scale up of community-based injectable contraceptives in Gombe State, Nigeria. Research is just the process to learn the answer to a question. In this study we want to learn what the factors are that may influence the scale up of community-based distribution of injectable contraceptives in Nigeria, in order to understand possible facilitators and barriers.

Invitation to participate: I am therefore asking / inviting you to take part in this research study because you are a community leader.

What is involved in the study – I am asking different people such as policy makers, program managers, health workers, community leaders, women who are the end-users of injectable contraceptives, and broader members of the community to participate in this study. About 100 people will participate in the study and all will be from Nigeria. If you agree to participate it means you will take part in a key informant interview. Each interview will last for about 60 minutes. In the interviews I will ask questions about your knowledge and your role in the implementation or use of injectable contraceptives as well as what you think might be the challenges and things that work well in implementing this program. There are no right and wrong answers; I am interested in understanding your opinions. Please note that I intend to audio-tape this interview.

Risks and Benefits of being involved in the study – There are no invasive procedures involved in this study, thus no physical harm is envisaged. However, you don't have to answer any question you are not comfortable with.

There are no direct benefits for participating in this study.

The participant will be given pertinent information on the study while involved in the project and after the results are available.

Participation is voluntary: Refusal to participate will involve no penalty or loss of benefits to which you are otherwise entitled. Also, you may discontinue participation at any time without penalty or loss of benefits to which you are otherwise entitled.

Reimbursements for transportation: Transportation costs incurred in order to participate in the study will be reimbursed at the rate of ₦100 per km.

Confidentiality: Efforts will be made to keep personal information confidential. Absolute confidentiality cannot be guaranteed. Personal information may be disclosed if required by law.

For peer review only

Organizations that may inspect and/or copy the research records for quality assurance and data analysis include groups such as the Research Ethics Committee. If results are published, may lead to individual / cohort identification.

Contact details of researcher – for further information / reporting of study related adverse events.

Dr Oluwaseun Akinyemi
Department of Health Policy and Management
College of Medicine
University of Ibadan
Ibadan, Nigeria
+234 803 502 0136
seunakinyemi@hotmail.com

Contact details of UI/UCH Ethics Committee chair (Nigeria) – for more information.

Prof. Catherine O. Falade
Chairperson, University of Ibadan/University College Hospital Ethics Committee
Institute for Advanced Medical Research and Training (IAMRAT)
College of Medicine, University of Ibadan
Ibadan, Nigeria
+234 803 326 4593
cfalade@comui.edu.ng

Contact details of REC administrator and chair (South Africa) – for more information.

Ms Zanele Ndlovu
Research Administrator
Human Research Ethics Committee (Medical)
University of the Witwatersrand
Johannesburg, South Africa
+27 11 717 1252
zanele.ndlovu@wits.ac.za

For peer review only

Participant Information Sheet for religious leaders

Study title: Scale up of community-based injectable contraceptives in Gombe State, Nigeria

Introduction:

Good day! I am Oluwaseun Akinyemi, a PhD candidate of the School of Public Health, University of the Witwatersrand, Johannesburg. I am doing research on the scale up of community-based injectable contraceptives in Gombe State, Nigeria. Research is just the process to learn the answer to a question. In this study we want to learn what the factors are that may influence the scale up of community-based distribution of injectable contraceptives in Nigeria, in order to understand possible facilitators and barriers.

Invitation to participate: I am therefore asking / inviting you to take part in this research study because you are a religious leader in this community.

What is involved in the study – I am asking different people such as policy makers, program managers, health workers, community leaders, women who are the end-users of injectable contraceptives, and broader members of the community to participate in this study. About 100 people will participate in the study and all will be from Nigeria. If you agree to participate it means you will take part in a key informant interview. Each interview will last for about 60 minutes. In the interviews I will ask questions about your knowledge and your role in the implementation or use of injectable contraceptives as well as what you think might be the challenges and things that work well in implementing this program. There are no right and wrong answers; I am interested in understanding your opinions. Please note that I intend to audio-tape this interview.

Risks and Benefits of being involved in the study – There are no invasive procedures involved in this study, thus no physical harm is envisaged. However, you don't have to answer any question you are not comfortable with.

There are no direct benefits for participating in this study.

The participant will be given pertinent information on the study while involved in the project and after the results are available.

Participation is voluntary: Refusal to participate will involve no penalty or loss of benefits to which you are otherwise entitled. Also, you may discontinue participation at any time without penalty or loss of benefits to which you are otherwise entitled.

Reimbursements for transportation: Transportation costs incurred in order to participate in the study will be reimbursed at the rate of ₦100 per km.

Confidentiality: Efforts will be made to keep personal information confidential. Absolute confidentiality cannot be guaranteed. Personal information may be disclosed if required by law.

For peer review only

Organizations that may inspect and/or copy the research records for quality assurance and data analysis include groups such as the Research Ethics Committee. If results are published, may lead to individual / cohort identification.

Contact details of researcher – for further information / reporting of study related adverse events.

Dr Oluwaseun Akinyemi
Department of Health Policy and Management
College of Medicine
University of Ibadan
Ibadan, Nigeria
+234 803 502 0136
seunakinyemi@hotmail.com

Contact details of UI/UCH Ethics Committee chair (Nigeria) – for more information.

Prof. Catherine O. Falade
Chairperson, University of Ibadan/University College Hospital Ethics Committee
Institute for Advanced Medical Research and Training (IAMRAT)
College of Medicine, University of Ibadan
Ibadan, Nigeria
+234 803 326 4593
cfalade@comui.edu.ng

Contact details of REC administrator and chair (South Africa) – for more information.

Ms Zanele Ndlovu
Research Administrator
Human Research Ethics Committee (Medical)
University of the Witwatersrand
Johannesburg, South Africa
+27 11 717 1252
zanele.ndlovu@wits.ac.za

For peer review only

Participant Information Sheet for women who are current users of injectable contraceptives

Study title: Scale up of community-based injectable contraceptives in Gombe State, Nigeria

Introduction:

Good day! I am Oluwaseun Akinyemi, a PhD candidate of the School of Public Health, University of the Witwatersrand, Johannesburg. I am doing research on the scale up of community-based injectable contraceptives in Gombe State, Nigeria. Research is just the process to learn the answer to a question. In this study we want to learn what the factors are that may influence the scale up of community-based distribution of injectable contraceptives in Nigeria, in order to understand possible facilitators and barriers.

Invitation to participate: I am therefore asking / inviting you to take part in this research study because you use an injectable contraceptive.

What is involved in the study – I am asking different people such as policy makers, program managers, health workers, community leaders, women who are the end-users of injectable contraceptives, and broader members of the community to participate in this study. About 100 people will participate in the study and all will be from Nigeria. If you agree to participate it means you will take part in a key informant interview. Each interview will last for about 60 minutes. In the interviews I will ask questions about your knowledge and your role in the implementation or use of injectable contraceptives as well as what you think might be the challenges and things that work well in implementing this program. There are no right and wrong answers; I am interested in understanding your opinions. Please note that I intend to audio-tape this interview.

Risks and Benefits of being involved in the study – There are no invasive procedures involved in this study, thus no physical harm is envisaged. However, you don't have to answer any question you are not comfortable with.

There are no direct benefits for participating in this study.

The participant will be given pertinent information on the study while involved in the project and after the results are available.

Participation is voluntary: Refusal to participate will involve no penalty or loss of benefits to which you are otherwise entitled. Also, you may discontinue participation at any time without penalty or loss of benefits to which you are otherwise entitled.

Reimbursements for transportation: Transportation costs incurred in order to participate in the study will be reimbursed at the rate of ₦100 per km.

For peer review only

Confidentiality: Efforts will be made to keep personal information confidential. However, confidentiality is not guaranteed in a focus group discussion. Personal information may be disclosed if required by law.

Organizations that may inspect and/or copy the research records for quality assurance and data analysis include groups such as the Research Ethics Committee. If results are published, may lead to individual / cohort identification.

Contact details of researcher – for further information / reporting of study related adverse events.

Dr Oluwaseun Akinyemi
Department of Health Policy and Management
College of Medicine
University of Ibadan
Ibadan, Nigeria
+234 803 502 0136
seunakinyemi@hotmail.com

Contact details of UI/UCH Ethics Committee chair (Nigeria) – for more information.

Prof. Catherine O. Falade
Chairperson, University of Ibadan/University College Hospital Ethics Committee
Institute for Advanced Medical Research and Training (IAMRAT)
College of Medicine, University of Ibadan
Ibadan, Nigeria
+234 803 326 4593
cfalade@comui.edu.ng

Contact details of REC administrator and chair (South Africa– for more information.

Ms Zanele Ndlovu
Research Administrator
Human Research Ethics Committee (Medical)
University of the Witwatersrand
Johannesburg, South Africa
+27 11 717 1252
zanele.ndlovu@wits.ac.za

For peer review only

Participant Information Sheet for women who do not use injectable contraceptives

Study title: Scale up of community-based injectable contraceptives in Gombe State, Nigeria

Introduction:

Good day! I am Oluwaseun Akinyemi, a PhD candidate of the School of Public Health, University of the Witwatersrand, Johannesburg. I am doing research on the scale up of community-based injectable contraceptives in Gombe State, Nigeria. Research is just the process to learn the answer to a question. In this study we want to learn what the factors are that may influence the scale up of community-based distribution of injectable contraceptives in Nigeria, in order to understand possible facilitators and barriers.

Invitation to participate: I am therefore asking / inviting you to take part in this research study because you do not use an injectable contraceptive.

What is involved in the study – I am asking different people such as policy makers, program managers, health workers, community leaders, women who are the end-users of injectable contraceptives, and broader members of the community to participate in this study. About 100 people will participate in the study and all will be from Nigeria. If you agree to participate it means you will take part in a key informant interview. Each interview will last for about 60 minutes. In the interviews I will ask questions about your knowledge and your role in the implementation or use of injectable contraceptives as well as what you think might be the challenges and things that work well in implementing this program. There are no right and wrong answers; I am interested in understanding your opinions. Please note that I intend to audio-tape this interview.

Risks and Benefits of being involved in the study – There are no invasive procedures involved in this study, thus no physical harm is envisaged. However, you don't have to answer any question you are not comfortable with.

There are no direct benefits for participating in this study.

The participant will be given pertinent information on the study while involved in the project and after the results are available.

Participation is voluntary: Refusal to participate will involve no penalty or loss of benefits to which you are otherwise entitled. Also, you may discontinue participation at any time without penalty or loss of benefits to which you are otherwise entitled.

Reimbursements for transportation: Transportation costs incurred in order to participate in the study will be reimbursed at the rate of ₦100 per km.

Confidentiality: Efforts will be made to keep personal information confidential. However, confidentiality is not guaranteed in a focus group discussion. Personal information may be disclosed if required by law.

For peer review only

Organizations that may inspect and/or copy the research records for quality assurance and data analysis include groups such as the Research Ethics Committee. If results are published, may lead to individual / cohort identification.

Contact details of researcher – for further information / reporting of study related adverse events.

Dr Oluwaseun Akinyemi
Department of Health Policy and Management
College of Medicine
University of Ibadan
Ibadan, Nigeria
+234 803 502 0136
seunakinyemi@hotmail.com

Contact details of UI/UCH Ethics Committee chair (Nigeria) – for more information.

Prof. Catherine O. Falade
Chairperson, University of Ibadan/University College Hospital Ethics Committee
Institute for Advanced Medical Research and Training (IAMRAT)
College of Medicine, University of Ibadan
Ibadan, Nigeria
+234 803 326 4593
cfalade@comui.edu.ng

Contact details of REC administrator and chair (South Africa– for more information.

Ms Zanele Ndlovu
Research Administrator
Human Research Ethics Committee (Medical)
University of the Witwatersrand
Johannesburg, South Africa
+27 11 717 1252
zanele.ndlovu@wits.ac.za

For peer review only

Participant Information Sheet for married men

Study title: Scale up of community-based injectable contraceptives in Gombe State, Nigeria

Introduction:

Good day! I am Oluwaseun Akinyemi, a PhD candidate of the School of Public Health, University of the Witwatersrand, Johannesburg. I am doing research on the scale up of community-based injectable contraceptives in Gombe State, Nigeria. Research is just the process to learn the answer to a question. In this study we want to learn what the factors are that may influence the scale up of community-based distribution of injectable contraceptives in Nigeria, in order to understand possible facilitators and barriers.

Invitation to participate: I am therefore asking / inviting you to take part in this research study because you are a married man.

What is involved in the study – I am asking different people such as policy makers, program managers, health workers, community leaders, women who are the end-users of injectable contraceptives, and broader members of the community to participate in this study. About 100 people will participate in the study and all will be from Nigeria. If you agree to participate it means you will take part in a key informant interview. Each interview will last for about 60 minutes. In the interviews I will ask questions about your knowledge and your role in the implementation or use of injectable contraceptives as well as what you think might be the challenges and things that work well in implementing this program. There are no right and wrong answers; I am interested in understanding your opinions. Please note that I intend to audio-tape this interview.

Risks and Benefits of being involved in the study – There are no invasive procedures involved in this study, thus no physical harm is envisaged. However, you don't have to answer any question you are not comfortable with.

There are no direct benefits for participating in this study.

The participant will be given pertinent information on the study while involved in the project and after the results are available.

Participation is voluntary: Refusal to participate will involve no penalty or loss of benefits to which you are otherwise entitled. Also, you may discontinue participation at any time without penalty or loss of benefits to which you are otherwise entitled.

Reimbursements for transportation: Transportation costs incurred in order to participate in the study will be reimbursed at the rate of ₦100 per km.

Confidentiality: Efforts will be made to keep personal information confidential. However, confidentiality is not guaranteed in a focus group discussion. Personal information may be disclosed if required by law.

For peer review only

Organizations that may inspect and/or copy the research records for quality assurance and data analysis include groups such as the Research Ethics Committee. If results are published, may lead to individual / cohort identification.

Contact details of researcher – for further information / reporting of study related adverse events.

Dr Oluwaseun Akinyemi
Department of Health Policy and Management
College of Medicine
University of Ibadan
Ibadan, Nigeria
+234 803 502 0136
seunakinyemi@hotmail.com

Contact details of UI/UCH Ethics Committee chair (Nigeria) – for more information.

Prof. Catherine O. Falade
Chairperson, University of Ibadan/University College Hospital Ethics Committee
Institute for Advanced Medical Research and Training (IAMRAT)
College of Medicine, University of Ibadan
Ibadan, Nigeria
+234 803 326 4593
cfalade@comui.edu.ng

Contact details of REC administrator and chair (South Africa) – for more information.

Ms Zanele Ndlovu
Research Administrator
Human Research Ethics Committee (Medical)
University of the Witwatersrand
Johannesburg, South Africa
+27 11 717 1252
zanele.ndlovu@wits.ac.za

For peer review only

Participant Information Sheet for unmarried/single men

Study title: Scale up of community-based injectable contraceptives in Gombe State, Nigeria

Introduction:

Good day! I am Oluwaseun Akinyemi, a PhD candidate of the School of Public Health, University of the Witwatersrand, Johannesburg. I am doing research on the scale up of community-based injectable contraceptives in Gombe State, Nigeria. Research is just the process to learn the answer to a question. In this study we want to learn what the factors are that may influence the scale up of community-based distribution of injectable contraceptives in Nigeria, in order to understand possible facilitators and barriers.

Invitation to participate: I am therefore asking / inviting you to take part in this research study because you are an unmarried/ single man.

What is involved in the study – I am asking different people such as policy makers, program managers, health workers, community leaders, women who are the end-users of injectable contraceptives, and broader members of the community to participate in this study. About 100 people will participate in the study and all will be from Nigeria. If you agree to participate it means you will take part in a key informant interview. Each interview will last for about 60 minutes. In the interviews I will ask questions about your knowledge and your role in the implementation or use of injectable contraceptives as well as what you think might be the challenges and things that work well in implementing this program. There are no right and wrong answers; I am interested in understanding your opinions. Please note that I intend to audio-tape this interview.

Risks and Benefits of being involved in the study – There are no invasive procedures involved in this study, thus no physical harm is envisaged. However, you don't have to answer any question you are not comfortable with.

There are no direct benefits for participating in this study.

The participant will be given pertinent information on the study while involved in the project and after the results are available.

Participation is voluntary: Refusal to participate will involve no penalty or loss of benefits to which you are otherwise entitled. Also, you may discontinue participation at any time without penalty or loss of benefits to which you are otherwise entitled.

Reimbursements for transportation: Transportation costs incurred in order to participate in the study will be reimbursed at the rate of ₦100 per km.

Confidentiality: Efforts will be made to keep personal information confidential. However, confidentiality is not guaranteed in a focus group discussion. Personal information may be disclosed if required by law.

For peer review only

Organizations that may inspect and/or copy the research records for quality assurance and data analysis include groups such as the Research Ethics Committee. If results are published, may lead to individual / cohort identification.

Contact details of researcher – for further information / reporting of study related adverse events.

Dr Oluwaseun Akinyemi
Department of Health Policy and Management
College of Medicine
University of Ibadan
Ibadan, Nigeria
+234 803 502 0136
seunakinyemi@hotmail.com

Contact details of UI/UCH Ethics Committee chair (Nigeria) – for more information.

Prof. Catherine O. Falade
Chairperson, University of Ibadan/University College Hospital Ethics Committee
Institute for Advanced Medical Research and Training (IAMRAT)
College of Medicine, University of Ibadan
Ibadan, Nigeria
+234 803 326 4593
cfalade@comui.edu.ng

Contact details of REC administrator and chair (South Africa) – for more information.

Ms Zanele Ndlovu
Research Administrator
Human Research Ethics Committee (Medical)
University of the Witwatersrand
Johannesburg, South Africa
+27 11 717 1252
zanele.ndlovu@wits.ac.za

For peer review only

Informed Consent Agreement for Senior MoH officials

Study title: Scale up of community-based injectable contraceptives in Gombe State, Nigeria

SERIAL NUMBER ___________________

Background and purpose: One of the health innovations that have been piloted with significant success around the world, including Nigeria, is the community-based distribution of injectable contraceptives. This study will contribute to providing evidence towards aiding our understanding of the barriers and facilitators to the introduction, dissemination, diffusion and integration of the community-based distribution of injectable contraceptives intervention in Nigeria.

Procedures: You have been selected as one of this study's participants for a key informant interview. Each interview will last for about 60 minutes. In the interviews I will ask questions about your knowledge and your role in the implementation or use of injectable contraceptives as well as what you think might be the challenges and things that work well in implementing this program.

Potential Risks: There are no health risks associated with the interviews and discussions.

Potential benefits: The findings of this study will help enhance the scale up of injectable contraceptives in Nigeria thereby helping to promote maternal and child health as well as healthier families.

Reimbursements for "out of pocket" expenses: Transportation costs incurred in order to participate in the study will be reimbursed at the rate of ₦100 per km.

Withdrawal from participation: If you agree to take part you are free to end your participation in this study at any time you want without having to give reasons.

Additional Information: In case you have any question or concern about this study please feel free to contact Dr Oluwaseun Akinyemi, Department of Health Policy and Management, College of Medicine, University of Ibadan Ibadan, Nigeria (Tel. No.: +234 803 502 0136; Email: seunakinyemi@hotmail.com)

Informed consent record: Please read this statement carefully before you sign and if you agree on participating, please fill free to sign this form below to affirm your consent.

I, ________________________________ clearly understand the aims of the project titled **"Scale up of community-based injectable contraceptives in Gombe State, Nigeria"** which has been explained to me by the researcher. I have been given the chance to ask questions and I am satisfied with the answers to all of my questions and I agree to participate in the study. I understand that I may revoke my consent and leave the study at any stage, if I wish so, with no negative consequences.

For peer review only

Participant’s name: ____

Participant’s signature or thumbprint: ____________________ Date: ______________

Witness Name (As appropriate): ____

Witness signature (As appropriate): ___________________ Date: _________________

Study team member’s statement: I, the undersigned, have explained to the participant in a language that s/he understands; the procedures to be followed in the study, the risks and benefits involved, and the obligations of the study team.

___________________________ ___________________________ _______________

Name Signature Date

For peer review only

Informed Consent Agreement for program managers

Study title: Scale up of community-based injectable contraceptives in Gombe State, Nigeria

SERIAL NUMBER ___________________

Background and purpose: One of the health innovations that have been piloted with significant success around the world, including Nigeria, is the community-based distribution of injectable contraceptives. This study will contribute to providing evidence towards aiding our understanding of the barriers and facilitators to the introduction, dissemination, diffusion and integration of the community-based distribution of injectable contraceptives intervention in Nigeria.

Procedures: You have been selected as one of this study's participants for a key informant interview. Each interview will last for about 60 minutes. In the interviews I will ask questions about your knowledge and your role in the implementation or use of injectable contraceptives as well as what you think might be the challenges and things that work well in implementing this program.

Potential Risks: There are no health risks associated with the interviews and discussions.

Potential benefits: The findings of this study will help enhance the scale up of injectable contraceptives in Nigeria thereby helping to promote maternal and child health as well as healthier families.

Reimbursements for "out of pocket" expenses: Transportation costs incurred in order to participate in the study will be reimbursed at the rate of ₦100 per km.

Withdrawal from participation: If you agree to take part you are free to end your participation in this study at any time you want without having to give reasons.

Additional Information: In case you have any question or concern about this study please feel free to contact Dr Oluwaseun Akinyemi, Department of Health Policy and Management, College of Medicine, University of Ibadan Ibadan, Nigeria (Tel. No.: +234 803 502 0136; Email: seunakinyemi@hotmail.com)

Informed consent record: Please read this statement carefully before you sign and if you agree on participating, please fill free to sign this form below to affirm your consent.

I, ________________________________ clearly understand the aims of the project titled **"Scale up of community-based injectable contraceptives in Gombe State, Nigeria"** which has been explained to me by the researcher. I have been given the chance to ask questions and I am satisfied with the answers to all of my questions and I agree to participate in the study. I understand that I may revoke my consent and leave the study at any stage, if I wish so, with no negative consequences.

For peer review only

Participant's name: ____

Participant's signature or thumbprint: ____________________ Date: ______________

Witness Name (As appropriate): ____

Witness signature (As appropriate): ___________________ Date: _________________

Study team member's statement: I, the undersigned, have explained to the participant in a language that s/he understands; the procedures to be followed in the study, the risks and benefits involved, and the obligations of the study team.

___________________________ ___________________________ _______________

Name Signature Date

For peer review only

Informed Consent Agreement for health workers

Study title: Scale up of community-based injectable contraceptives in Gombe State, Nigeria

SERIAL NUMBER ___________________

Background and purpose: One of the health innovations that have been piloted with significant success around the world, including Nigeria, is the community-based distribution of injectable contraceptives. This study will contribute to providing evidence towards aiding our understanding of the barriers and facilitators to the introduction, dissemination, diffusion and integration of the community-based distribution of injectable contraceptives intervention in Nigeria.

Procedures: You have been selected as one of this study's participants for an in-depth interview. Each interview will last for about 60 minutes. In the interviews and discussions I will ask questions about your knowledge and your role in the implementation or use of injectable contraceptives as well as what you think might be the challenges and things that work well in implementing this program.

Potential Risks: There are no health risks associated with the interviews and discussions.

Potential benefits: The findings of this study will help enhance the scale up of injectable contraceptives in Nigeria thereby helping to promote maternal and child health as well as healthier families.

Reimbursements for "out of pocket" expenses: Transportation costs incurred in order to participate in the study will be reimbursed at the rate of ₦100 per km.

Withdrawal from participation: If you agree to take part you are free to end your participation in this study at any time you want without having to give reasons.

Additional Information: In case you have any question or concern about this study please feel free to contact Dr Oluwaseun Akinyemi, Department of Health Policy and Management, College of Medicine, University of Ibadan Ibadan, Nigeria (Tel. No.: +234 803 502 0136; Email: seunakinyemi@hotmail.com)

Informed consent record: Please read this statement carefully before you sign and if you agree on participating, please fill free to sign this form below to affirm your consent.

I, _________________________________ clearly understand the aims of the project titled **"Scale up of community-based injectable contraceptives in Gombe State, Nigeria"** which has been explained to me by the researcher. I have been given the chance to ask questions and I am satisfied with the answers to all of my questions and I agree to participate in the study. I understand that I may revoke my consent and leave the study at any stage, if I wish so, with no negative consequences.

For peer review only

Participant's name: ____

Participant's signature or thumbprint: ____________________ Date: ______________

Witness Name (As appropriate): ____

Witness signature (As appropriate): ___________________ Date: _________________

Study team member's statement: I, the undersigned, have explained to the participant in a language that s/he understands; the procedures to be followed in the study, the risks and benefits involved, and the obligations of the study team.

___________________________ ___________________________ _______________

Name Signature Date

For peer review only

Informed Consent Agreement for community leaders

Study title: Scale up of community-based injectable contraceptives in Gombe State, Nigeria

SERIAL NUMBER ___________________

Background and purpose: One of the health innovations that have been piloted with significant success around the world, including Nigeria, is the community-based distribution of injectable contraceptives. This study will contribute to providing evidence towards aiding our understanding of the barriers and facilitators to the introduction, dissemination, diffusion and integration of the community-based distribution of injectable contraceptives intervention in Nigeria.

Procedures: You have been selected as one of this study's participants for an in-depth interview. Each interview will last for about 60 minutes. In the interviews and discussions I will ask questions about your knowledge and your role in the implementation or use of injectable contraceptives as well as what you think might be the challenges and things that work well in implementing this program.

Potential Risks: There are no health risks associated with the interviews and discussions.

Potential benefits: The findings of this study will help enhance the scale up of injectable contraceptives in Nigeria thereby helping to promote maternal and child health as well as healthier families.

Reimbursements for "out of pocket" expenses: Transportation costs incurred in order to participate in the study will be reimbursed at the rate of ₦100 per km.

Withdrawal from participation: If you agree to take part you are free to end your participation in this study at any time you want without having to give reasons.

Additional Information: In case you have any question or concern about this study please feel free to contact Dr Oluwaseun Akinyemi, Department of Health Policy and Management, College of Medicine, University of Ibadan Ibadan, Nigeria (Tel. No.: +234 803 502 0136; Email: seunakinyemi@hotmail.com)

Informed consent record: Please read this statement carefully before you sign and if you agree on participating, please fill free to sign this form below to affirm your consent.

I, _________________________________ clearly understand the aims of the project titled **"Scale up of community-based injectable contraceptives in Gombe State, Nigeria"** which has been explained to me by the researcher. I have been given the chance to ask questions and I am satisfied with the answers to all of my questions and I agree to participate in the study. I understand that I may revoke my consent and leave the study at any stage, if I wish so, with no negative consequences.

For peer review only

Participant's name: ____

Participant's signature or thumbprint: ____________________ Date: ______________

Witness Name (As appropriate): ____

Witness signature (As appropriate): ___________________ Date: ________________

Study team member's statement: I, the undersigned, have explained to the participant in a language that s/he understands; the procedures to be followed in the study, the risks and benefits involved, and the obligations of the study team.

___________________________ ___________________________ _______________

Name Signature Date

For peer review only

Informed Consent Agreement for religious leaders

Study title: Scale up of community-based injectable contraceptives in Gombe State, Nigeria

SERIAL NUMBER ____________________

Background and purpose: One of the health innovations that have been piloted with significant success around the world, including Nigeria, is the community-based distribution of injectable contraceptives. This study will contribute to providing evidence towards aiding our understanding of the barriers and facilitators to the introduction, dissemination, diffusion and integration of the community-based distribution of injectable contraceptives intervention in Nigeria.

Procedures: You have been selected as one of this study's participants for an in-depth interview. Each interview will last for about 60 minutes. In the interviews and discussions I will ask questions about your knowledge and your role in the implementation or use of injectable contraceptives as well as what you think might be the challenges and things that work well in implementing this program.

Potential Risks: There are no health risks associated with the interviews and discussions.

Potential benefits: The findings of this study will help enhance the scale up of injectable contraceptives in Nigeria thereby helping to promote maternal and child health as well as healthier families.

Reimbursements for "out of pocket" expenses: Transportation costs incurred in order to participate in the study will be reimbursed at the rate of ₦100 per km.

Withdrawal from participation: If you agree to take part you are free to end your participation in this study at any time you want without having to give reasons.

Additional Information: In case you have any question or concern about this study please feel free to contact Dr Oluwaseun Akinyemi, Department of Health Policy and Management, College of Medicine, University of Ibadan Ibadan, Nigeria (Tel. No.: +234 803 502 0136; Email: seunakinyemi@hotmail.com)

Informed consent record: Please read this statement carefully before you sign and if you agree on participating, please fill free to sign this form below to affirm your consent.

I, ________________________________ clearly understand the aims of the project titled **"Scale up of community-based injectable contraceptives in Gombe State, Nigeria"** which has been explained to me by the researcher. I have been given the chance to ask questions and I am satisfied with the answers to all of my questions and I agree to participate in the study. I understand that I may revoke my consent and leave the study at any stage, if I wish so, with no negative consequences.

For peer review only

Participant's name: ____

Participant's signature or thumbprint: ____________________ Date: ______________

Witness Name (As appropriate): ____

Witness signature (As appropriate): ___________________ Date: _________________

Study team member's statement: I, the undersigned, have explained to the participant in a language that s/he understands; the procedures to be followed in the study, the risks and benefits involved, and the obligations of the study team.

___________________________ ___________________________ _______________

Name Signature Date

For peer review only

Informed Consent Agreement for women who are current users of injectable contraceptives

Study title: Scale up of community-based injectable contraceptives in Gombe State, Nigeria

SERIAL NUMBER ___________________

Background and purpose: One of the health innovations that have been piloted with significant success around the world, including Nigeria, is the community-based distribution of injectable contraceptives. This study will contribute to providing evidence towards aiding our understanding of the barriers and facilitators to the introduction, dissemination, diffusion and integration of the community-based distribution of injectable contraceptives intervention in Nigeria.

Procedures: You have been selected as one of this study's participants for a focus group discussion or a questionnaire interview. Each interview or focus group discussion will last for about 60 minutes. In the interviews and discussions I will ask questions about your knowledge and your role in the implementation or use of injectable contraceptives as well as what you think might be the challenges and things that work well in implementing this program.

Potential Risks: There are no health risks associated with the interviews and discussions.

Potential benefits: The findings of this study will help enhance the scale up of injectable contraceptives in Nigeria thereby helping to promote maternal and child health as well as healthier families.

Reimbursements for "out of pocket" expenses: Transportation costs incurred in order to participate in the study will be reimbursed at the rate of ₦100 per km.

Withdrawal from participation: If you agree to take part you are free to end your participation in this study at any time you want without having to give reasons.

Additional Information: In case you have any question or concern about this study please feel free to contact Dr Oluwaseun Akinyemi, Department of Health Policy and Management, College of Medicine, University of Ibadan Ibadan, Nigeria (Tel. No.: +234 803 502 0136; Email: seunakinyemi@hotmail.com)

Informed consent record: Please read this statement carefully before you sign and if you agree on participating, please fill free to sign this form below to affirm your consent.

I, ________________________________ clearly understand the aims of the project titled **"Scale up of community-based injectable contraceptives in Gombe State, Nigeria"** which has been explained to me by the researcher. I have been given the chance to ask questions and I am satisfied with the answers to all of my questions and I agree to participate in the study. I understand

that I may revoke my consent and leave the study at any stage, if I wish so, with no negative consequences.

Participant's name: ____

Participant's signature or thumbprint: _____________________ Date: ______________

Witness Name (As appropriate): ____

Witness signature (As appropriate): ___________________ Date: _________________

Study team member's statement: I, the undersigned, have explained to the participant in a language that s/he understands; the procedures to be followed in the study, the risks and benefits involved, and the obligations of the study team.

___________________________ ___________________________ _______________

Name Signature Date

For peer review only

Informed Consent Agreement for women who do not use injectable contraceptives

Study title: Scale up of community-based injectable contraceptives in Gombe State, Nigeria

SERIAL NUMBER ___________________

Background and purpose: One of the health innovations that have been piloted with significant success around the world, including Nigeria, is the community-based distribution of injectable contraceptives. This study will contribute to providing evidence towards aiding our understanding of the barriers and facilitators to the introduction, dissemination, diffusion and integration of the community-based distribution of injectable contraceptives intervention in Nigeria.

Procedures: You have been selected as one of this study's participants for a focus group discussion. Each focus group discussion will last for about 60 minutes. In the interviews and discussions I will ask questions about your knowledge and your role in the implementation or use of injectable contraceptives as well as what you think might be the challenges and things that work well in implementing this program.

Potential Risks: There are no health risks associated with the interviews and discussions.

Potential benefits: The findings of this study will help enhance the scale up of injectable contraceptives in Nigeria thereby helping to promote maternal and child health as well as healthier families.

Reimbursements for "out of pocket" expenses: Transportation costs incurred in order to participate in the study will be reimbursed at the rate of ₦100 per km.

Withdrawal from participation: If you agree to take part you are free to end your participation in this study at any time you want without having to give reasons.

Additional Information: In case you have any question or concern about this study please feel free to contact Dr Oluwaseun Akinyemi, Department of Health Policy and Management, College of Medicine, University of Ibadan Ibadan, Nigeria (Tel. No.: +234 803 502 0136; Email: seunakinyemi@hotmail.com)

Informed consent record: Please read this statement carefully before you sign and if you agree on participating, please fill free to sign this form below to affirm your consent.

I, _________________________________ clearly understand the aims of the project titled **"Scale up of community-based injectable contraceptives in Gombe State, Nigeria"** which has been explained to me by the researcher. I have been given the chance to ask questions and I am satisfied with the answers to all of my questions and I agree to participate in the study. I understand that I may revoke my consent and leave the study at any stage, if I wish so, with no negative consequences.

For peer review only

Participant's name: ____

Participant's signature or thumbprint: ____________________ Date: ______________

Witness Name (As appropriate): ____

Witness signature (As appropriate): ___________________ Date: _________________

Study team member's statement: I, the undersigned, have explained to the participant in a language that s/he understands; the procedures to be followed in the study, the risks and benefits involved, and the obligations of the study team.

___________________________ ___________________________ _______________

Name Signature Date

For peer review only

Informed Consent Agreement for married men

Study title: Scale up of community-based injectable contraceptives in Gombe State, Nigeria

SERIAL NUMBER ___________________

Background and purpose: One of the health innovations that have been piloted with significant success around the world, including Nigeria, is the community-based distribution of injectable contraceptives. This study will contribute to providing evidence towards aiding our understanding of the barriers and facilitators to the introduction, dissemination, diffusion and integration of the community-based distribution of injectable contraceptives intervention in Nigeria.

Procedures: You have been selected as one of this study's participants for a focus group discussion. Each focus group discussion will last for about 60 minutes. In the interviews and discussions I will ask questions about your knowledge and your role in the implementation or use of injectable contraceptives as well as what you think might be the challenges and things that work well in implementing this program.

Potential Risks: There are no health risks associated with the interviews and discussions.

Potential benefits: The findings of this study will help enhance the scale up of injectable contraceptives in Nigeria thereby helping to promote maternal and child health as well as healthier families.

Reimbursements for "out of pocket" expenses: Transportation costs incurred in order to participate in the study will be reimbursed at the rate of ₦100 per km.

Withdrawal from participation: If you agree to take part you are free to end your participation in this study at any time you want without having to give reasons.

Additional Information: In case you have any question or concern about this study please feel free to contact Dr Oluwaseun Akinyemi, Department of Health Policy and Management, College of Medicine, University of Ibadan Ibadan, Nigeria (Tel. No.: +234 803 502 0136; Email: seunakinyemi@hotmail.com)

Informed consent record: Please read this statement carefully before you sign and if you agree on participating, please fill free to sign this form below to affirm your consent.

I, ________________________________ clearly understand the aims of the project titled **"Scale up of community-based injectable contraceptives in Gombe State, Nigeria"** which has been explained to me by the researcher. I have been given the chance to ask questions and I am satisfied with the answers to all of my questions and I agree to participate in the study. I understand that I may revoke my consent and leave the study at any stage, if I wish so, with no negative consequences.

For peer review only

Participant’s name: ____

Participant’s signature or thumbprint: ______________________ Date: ________________

Witness Name (As appropriate): ____

Witness signature (As appropriate): _____________________ Date: __________________

Study team member’s statement: I, the undersigned, have explained to the participant in a language that s/he understands; the procedures to be followed in the study, the risks and benefits involved, and the obligations of the study team.

______________________________ ______________________________ _________________

Name Signature Date

For peer review only

Informed Consent Agreement for unmarried men

Study title: Scale up of community-based injectable contraceptives in Gombe State, Nigeria

SERIAL NUMBER ___________________

Background and purpose: One of the health innovations that have been piloted with significant success around the world, including Nigeria, is the community-based distribution of injectable contraceptives. This study will contribute to providing evidence towards aiding our understanding of the barriers and facilitators to the introduction, dissemination, diffusion and integration of the community-based distribution of injectable contraceptives intervention in Nigeria.

Procedures: You have been selected as one of this study's participants for a focus group discussion. Each focus group discussion will last for about 60 minutes. In the interviews and discussions I will ask questions about your knowledge and your role in the implementation or use of injectable contraceptives as well as what you think might be the challenges and things that work well in implementing this program.

Potential Risks: There are no health risks associated with the interviews and discussions.

Potential benefits: The findings of this study will help enhance the scale up of injectable contraceptives in Nigeria thereby helping to promote maternal and child health as well as healthier families.

Reimbursements for "out of pocket" expenses: Transportation costs incurred in order to participate in the study will be reimbursed at the rate of ₦100 per km.

Withdrawal from participation: If you agree to take part you are free to end your participation in this study at any time you want without having to give reasons.

Additional Information: In case you have any question or concern about this study please feel free to contact Dr Oluwaseun Akinyemi, Department of Health Policy and Management, College of Medicine, University of Ibadan Ibadan, Nigeria (Tel. No.: +234 803 502 0136; Email: seunakinyemi@hotmail.com)

Informed consent record: Please read this statement carefully before you sign and if you agree on participating, please fill free to sign this form below to affirm your consent.

I, _________________________________ clearly understand the aims of the project titled **"Scale up of community-based injectable contraceptives in Gombe State, Nigeria"** which has been explained to me by the researcher. I have been given the chance to ask questions and I am satisfied with the answers to all of my questions and I agree to participate in the study. I understand that I may revoke my consent and leave the study at any stage, if I wish so, with no negative consequences.

For peer review only

Participant's name: ____

Participant's signature or thumbprint: __________________ Date: _____________

Witness Name (As appropriate): ____

Witness signature (As appropriate): _________________ Date: ________________

Study team member's statement: I, the undersigned, have explained to the participant in a language that s/he understands; the procedures to be followed in the study, the risks and benefits involved, and the obligations of the study team.

________________________ ________________________ _____________

Name Signature Date

For peer review only

Informed Consent Agreement to audio record interview senior MoH officials

Study title: Scale up of community-based injectable contraceptives in Gombe State, Nigeria

SERIAL NUMBER ___________________

Background and purpose: One of the health innovations that have been piloted with significant success around the world, including Nigeria, is the community-based distribution of injectable contraceptives.

Additional Information: In case you have any question or concern about this study please feel free to contact Dr Oluwaseun Akinyemi, Department of Health Policy and Management, College of Medicine, University of Ibadan Ibadan, Nigeria (Tel. No.: +234 803 502 0136; Email: seunakinyemi@hotmail.com)

Informed consent to record: Please read this statement carefully before you sign and if you agree that the interview / discussion with you should be audio recorded, please fill free to sign this form below to affirm your consent.

I, ______________________________ clearly understand the aims of the project titled **"Scale up of community-based injectable contraceptives in Gombe State, Nigeria"** which has been explained to me by the researcher. I have been given the chance to ask questions and I am satisfied with the answers to all of my questions and I agree to participate in the study. I understand that I may revoke my consent and leave the study at any stage, if I wish so, with no negative consequences. I agree that the interview / discussion with me should be audio recorded. I understand that the recording is for research purposes only.

Participant's name: ____

Participant's signature or thumbprint: ___________________ Date: ______________

Witness Name (As appropriate): ____

Witness signature (As appropriate): ___________________ Date: _________________

Study team member's statement: I, the undersigned, have explained to the participant in a language that s/he understands; the procedures to be followed in the study (including audio recording of the interview), the risks and benefits involved, and the obligations of the study team.

___________________________ ___________________________ _______________

Name Signature Date

For peer review only

Informed Consent Agreement to audio record interview with program managers

Study title: Scale up of community-based injectable contraceptives in Gombe State, Nigeria

SERIAL NUMBER ____________________

Background and purpose: One of the health innovations that have been piloted with significant success around the world, including Nigeria, is the community-based distribution of injectable contraceptives.

Additional Information: In case you have any question or concern about this study please feel free to contact Dr Oluwaseun Akinyemi, Department of Health Policy and Management, College of Medicine, University of Ibadan Ibadan, Nigeria (Tel. No.: +234 803 502 0136; Email: seunakinyemi@hotmail.com)

Informed consent to record: Please read this statement carefully before you sign and if you agree that the interview / discussion with you should be audio recorded, please fill free to sign this form below to affirm your consent.

I, ________________________________ clearly understand the aims of the project titled **"Scale up of community-based injectable contraceptives in Gombe State, Nigeria"** which has been explained to me by the researcher. I have been given the chance to ask questions and I am satisfied with the answers to all of my questions and I agree to participate in the study. I understand that I may revoke my consent and leave the study at any stage, if I wish so, with no negative consequences. I agree that the interview / discussion with me should be audio recorded. I understand that the recording is for research purposes only.

Participant's name: ____

Participant's signature or thumbprint: _____________________ Date: _______________

Witness Name (As appropriate): ____

Witness signature (As appropriate): ____________________ Date: __________________

Study team member's statement: I, the undersigned, have explained to the participant in a language that s/he understands; the procedures to be followed in the study (including audio recording of the interview), the risks and benefits involved, and the obligations of the study team.

_____________________________ _____________________________ ________________

Name Signature Date

For peer review only

Informed Consent Agreement to audio record interview with health workers

Study title: Scale up of community-based injectable contraceptives in Gombe State, Nigeria

SERIAL NUMBER ____________________

Background and purpose: One of the health innovations that have been piloted with significant success around the world, including Nigeria, is the community-based distribution of injectable contraceptives.

Additional Information: In case you have any question or concern about this study please feel free to contact Dr Oluwaseun Akinyemi, Department of Health Policy and Management, College of Medicine, University of Ibadan Ibadan, Nigeria (Tel. No.: +234 803 502 0136; Email: seunakinyemi@hotmail.com)

Informed consent to record: Please read this statement carefully before you sign and if you agree that the interview / discussion with you should be audio recorded, please fill free to sign this form below to affirm your consent.

I, ________________________________ clearly understand the aims of the project titled **"Scale up of community-based injectable contraceptives in Gombe State, Nigeria"** which has been explained to me by the researcher. I have been given the chance to ask questions and I am satisfied with the answers to all of my questions and I agree to participate in the study. I understand that I may revoke my consent and leave the study at any stage, if I wish so, with no negative consequences. I agree that the interview / discussion with me should be audio recorded. I understand that the recording is for research purposes only.

Participant's name: ____

Participant's signature or thumbprint: _____________________ Date: _______________

Witness Name (As appropriate): ____

Witness signature (As appropriate): ____________________ Date: __________________

Study team member's statement: I, the undersigned, have explained to the participant in a language that s/he understands; the procedures to be followed in the study (including audio recording of the interview), the risks and benefits involved, and the obligations of the study team.

_____________________________ _____________________________ ________________

Name Signature Date

For peer review only

Informed Consent Agreement to audio record interview with community leaders

Study title: Scale up of community-based injectable contraceptives in Gombe State, Nigeria

SERIAL NUMBER ____________________

Background and purpose: One of the health innovations that have been piloted with significant success around the world, including Nigeria, is the community-based distribution of injectable contraceptives.

Additional Information: In case you have any question or concern about this study please feel free to contact Dr Oluwaseun Akinyemi, Department of Health Policy and Management, College of Medicine, University of Ibadan Ibadan, Nigeria (Tel. No.: +234 803 502 0136; Email: seunakinyemi@hotmail.com)

Informed consent to record: Please read this statement carefully before you sign and if you agree that the interview / discussion with you should be audio recorded, please fill free to sign this form below to affirm your consent.

I, ________________________________ clearly understand the aims of the project titled **"Scale up of community-based injectable contraceptives in Gombe State, Nigeria"** which has been explained to me by the researcher. I have been given the chance to ask questions and I am satisfied with the answers to all of my questions and I agree to participate in the study. I understand that I may revoke my consent and leave the study at any stage, if I wish so, with no negative consequences. I agree that the interview / discussion with me should be audio recorded. I understand that the recording is for research purposes only.

Participant's name: ____

Participant's signature or thumbprint: _____________________ Date: _______________

Witness Name (As appropriate): ____

Witness signature (As appropriate): ____________________ Date: __________________

Study team member's statement: I, the undersigned, have explained to the participant in a language that s/he understands; the procedures to be followed in the study (including audio recording of the interview), the risks and benefits involved, and the obligations of the study team.

_____________________________ _____________________________ ________________

Name Signature Date

For peer review only

Informed Consent Agreement to audio record interview with religious leaders

Study title: Scale up of community-based injectable contraceptives in Gombe State, Nigeria

SERIAL NUMBER ___________________

Background and purpose: One of the health innovations that have been piloted with significant success around the world, including Nigeria, is the community-based distribution of injectable contraceptives.

Additional Information: In case you have any question or concern about this study please feel free to contact Dr Oluwaseun Akinyemi, Department of Health Policy and Management, College of Medicine, University of Ibadan Ibadan, Nigeria (Tel. No.: +234 803 502 0136; Email: seunakinyemi@hotmail.com)

Informed consent to record: Please read this statement carefully before you sign and if you agree that the interview / discussion with you should be audio recorded, please fill free to sign this form below to affirm your consent.

I, ______________________________ clearly understand the aims of the project titled **"Scale up of community-based injectable contraceptives in Gombe State, Nigeria"** which has been explained to me by the researcher. I have been given the chance to ask questions and I am satisfied with the answers to all of my questions and I agree to participate in the study. I understand that I may revoke my consent and leave the study at any stage, if I wish so, with no negative consequences. I agree that the interview / discussion with me should be audio recorded. I understand that the recording is for research purposes only.

Participant's name: ____

Participant's signature or thumbprint: _____________________ Date: ______________

Witness Name (As appropriate): ____

Witness signature (As appropriate): ___________________ Date: _________________

Study team member's statement: I, the undersigned, have explained to the participant in a language that s/he understands; the procedures to be followed in the study (including audio recording of the interview), the risks and benefits involved, and the obligations of the study team.

___________________________ ___________________________ _______________

Name Signature Date

For peer review only

Informed Consent Agreement to audio record focus group discussion with women who currently use injectable contraceptive

Study title: Scale up of community-based injectable contraceptives in Gombe State, Nigeria

SERIAL NUMBER ___________________

Background and purpose: One of the health innovations that have been piloted with significant success around the world, including Nigeria, is the community-based distribution of injectable contraceptives.

Additional Information: In case you have any question or concern about this study please feel free to contact Dr Oluwaseun Akinyemi, Department of Health Policy and Management, College of Medicine, University of Ibadan Ibadan, Nigeria (Tel. No.: +234 803 502 0136; Email: seunakinyemi@hotmail.com)

Informed consent to record: Please read this statement carefully before you sign and if you agree that the interview / discussion with you should be audio recorded, please fill free to sign this form below to affirm your consent.

I, ______________________________ clearly understand the aims of the project titled **"Scale up of community-based injectable contraceptives in Gombe State, Nigeria"** which has been explained to me by the researcher. I have been given the chance to ask questions and I am satisfied with the answers to all of my questions and I agree to participate in the study. I understand that I may revoke my consent and leave the study at any stage, if I wish so, with no negative consequences. I agree that the interview / discussion with me should be audio recorded. I understand that the recording is for research purposes only.

Participant's name: ____

Participant's signature or thumbprint: ____________________ Date: ______________

Witness Name (As appropriate): ____

Witness signature (As appropriate): ___________________ Date: _________________

Study team member's statement: I, the undersigned, have explained to the participant in a language that s/he understands; the procedures to be followed in the study (including audio recording of the discussion), the risks and benefits involved, and the obligations of the study team.

___________________________ ___________________________ _______________

Name Signature Date

For peer review only

Informed Consent Agreement to audio record focus group discussion with women who do not use injectable contraceptive

Study title: Scale up of community-based injectable contraceptives in Gombe State, Nigeria

SERIAL NUMBER ___________________

Background and purpose: One of the health innovations that have been piloted with significant success around the world, including Nigeria, is the community-based distribution of injectable contraceptives.

Additional Information: In case you have any question or concern about this study please feel free to contact Dr Oluwaseun Akinyemi, Department of Health Policy and Management, College of Medicine, University of Ibadan Ibadan, Nigeria (Tel. No.: +234 803 502 0136; Email: seunakinyemi@hotmail.com)

Informed consent to record: Please read this statement carefully before you sign and if you agree that the interview / discussion with you should be audio recorded, please fill free to sign this form below to affirm your consent.

I, ______________________________ clearly understand the aims of the project titled **"Scale up of community-based injectable contraceptives in Gombe State, Nigeria"** which has been explained to me by the researcher. I have been given the chance to ask questions and I am satisfied with the answers to all of my questions and I agree to participate in the study. I understand that I may revoke my consent and leave the study at any stage, if I wish so, with no negative consequences. I agree that the interview / discussion with me should be audio recorded. I understand that the recording is for research purposes only.

Participant's name: ____

Participant's signature or thumbprint: ____________________ Date: ______________

Witness Name (As appropriate): ____

Witness signature (As appropriate): ___________________ Date: _________________

Study team member's statement: I, the undersigned, have explained to the participant in a language that s/he understands; the procedures to be followed in the study (including audio recording of the discussion), the risks and benefits involved, and the obligations of the study team.

___________________________ ___________________________ _______________

Name Signature Date

For peer review only

Informed Consent Agreement to audio record focus group discussion with married men

Study title: Scale up of community-based injectable contraceptives in Gombe State, Nigeria

SERIAL NUMBER ____________________

Background and purpose: One of the health innovations that have been piloted with significant success around the world, including Nigeria, is the community-based distribution of injectable contraceptives.

Additional Information: In case you have any question or concern about this study please feel free to contact Dr Oluwaseun Akinyemi, Department of Health Policy and Management, College of Medicine, University of Ibadan Ibadan, Nigeria (Tel. No.: +234 803 502 0136; Email: seunakinyemi@hotmail.com)

Informed consent to record: Please read this statement carefully before you sign and if you agree that the interview / discussion with you should be audio recorded, please fill free to sign this form below to affirm your consent.

I, ________________________________ clearly understand the aims of the project titled **"Scale up of community-based injectable contraceptives in Gombe State, Nigeria"** which has been explained to me by the researcher. I have been given the chance to ask questions and I am satisfied with the answers to all of my questions and I agree to participate in the study. I understand that I may revoke my consent and leave the study at any stage, if I wish so, with no negative consequences. I agree that the interview / discussion with me should be audio recorded. I understand that the recording is for research purposes only.

Participant's name: ____

Participant's signature or thumbprint: _____________________ Date: _______________

Witness Name (As appropriate): ____

Witness signature (As appropriate): ____________________ Date: __________________

Study team member's statement: I, the undersigned, have explained to the participant in a language that s/he understands; the procedures to be followed in the study (including audio recording of the discussion), the risks and benefits involved, and the obligations of the study team.

_____________________________ _____________________________ ________________

Name Signature Date

For peer review only

Informed Consent Agreement to audio record focus group discussion with single/unmarried men

Study title: Scale up of community-based injectable contraceptives in Gombe State, Nigeria

SERIAL NUMBER ___________________

Background and purpose: One of the health innovations that have been piloted with significant success around the world, including Nigeria, is the community-based distribution of injectable contraceptives.

Additional Information: In case you have any question or concern about this study please feel free to contact Dr Oluwaseun Akinyemi, Department of Health Policy and Management, College of Medicine, University of Ibadan Ibadan, Nigeria (Tel. No.: +234 803 502 0136; Email: seunakinyemi@hotmail.com)

Informed consent to record: Please read this statement carefully before you sign and if you agree that the interview / discussion with you should be audio recorded, please fill free to sign this form below to affirm your consent.

I, ______________________________ clearly understand the aims of the project titled **"Scale up of community-based injectable contraceptives in Gombe State, Nigeria"** which has been explained to me by the researcher. I have been given the chance to ask questions and I am satisfied with the answers to all of my questions and I agree to participate in the study. I understand that I may revoke my consent and leave the study at any stage, if I wish so, with no negative consequences. I agree that the interview / discussion with me should be audio recorded. I understand that the recording is for research purposes only.

Participant's name: ____

Participant's signature or thumbprint: ____________________ Date: ______________

Witness Name (As appropriate): ____

Witness signature (As appropriate): ___________________ Date: _________________

Study team member's statement: I, the undersigned, have explained to the participant in a language that s/he understands; the procedures to be followed in the study (including audio recording of the discussion), the risks and benefits involved, and the obligations of the study team.

___________________________ ___________________________ _______________

Name Signature Date

For peer review only

Distress Protocol

Distress Protocol 1: The protocol for managing distress in the context of a research focus group /interview

(Adapted from: Draucker C B, Martsolf D S and Poole C (2009) Developing Distress Protocols for research on Sensitive Topics. *Archives of Psychiatric Nursing* 23 (5) pp 343-350)

Distress

• A participant indicates they are experiencing a high level of stress or emotional distress

OR

• Exhibit behaviours suggestive that the discussion/interview is too stressful such as uncontrolled crying, shaking etc.

Stage 1 Response

• Stop the discussion/interview.

• The researcher (who is a health professional) will offer immediate support

• Assess mental status:

- Tell me what thoughts you are having?
- Tell me what you are feeling right now?
- Do you feel you are able to go on about your day?
- Do you feel safe?

Review

•If participant feels able to carry on; resume interview/discussion

•If participant is unable to carry on, Go to stage 2

Stage 2 Response

•Remove participant from discussion and accompany to quiet area or discontinue interview

•Encourage the participant to contact their GP or mental health provider

OR

•Offer, with participant consent, for a member of the research team to do so OR

•With participant consent contact a member of the health care team treating them for further advice/support

Follow up

•Follow participant up with courtesy call (if participant consents)

OR

•Encourage the participant to call either if he/she experiences increased distress in the hours/days following the focus group

For peer review only

Distress Protocol 2: The protocol for managing distress in the context of a research focus group /interview management

Adapted from: McCosker,H Barnard, A Gerber, R (2001). Undertaking Sensitive Research: Issues and Strategies for Meeting the Safety Needs of All. Forum: Qualitative Social Research, 2(1)

Pre-data collection

•The researcher would consider the potential physical and psychological impact on the researcher of the participants' description of life experiences.

•The researcher would consider how many interviews could be undertaken in a week.

•The researcher would be aware of the potential for emotional exhaustion.

Data collection stage

• For topics that are potentially sensitive/distressing data collection would be undertaken by two members of the research team.

•Regular scheduled debriefing sessions with the lead researcher.

•Research team would be encouraged to journal their thoughts and feelings which may then become part of fieldwork notes in some research approaches.

Analysis

•Research team would be alerted prior to transcription review of potentially "challenging" or "difficult" interviews

•The research team would have regular scheduled debriefing sessions with the lead researcher

Follow up

•Researcher will access a research mentor (supervisor) if he experiences increased distress in the hours/days following transcription.

For peer review only

Distress Protocol 3: The protocol for managing distress in the context of a research focus group /interview transcription

(Adapted from: Gregory, D Russell, C Phillips, L (1997). Beyond textual perfection: transcribers as vulnerable persons. Qualitative Health Research, 7(2), 294-300.)

Pre-data collection

• The transcriber would be considered in the research proposal, with a clear indication of how s/he will be provided with a "safe" working environment while also maintaining the "quality" of the research.

Ethical review stage

• The transcriber would be included in the ethical clearance process.

• The transcriber would be informed of the nature of the research and the type of data.

Pre-transcription

• The transcriber would be alerted prior to the transcription of potentially "challenging" or "difficult" interviews.

•The transcriber would have regular scheduled debriefing sessions with a named member of the research team.

During Transcription

•Transcriber would have prompt access to an appropriate person for crisis counselling.

•S/he would have a clearly documented termination from the transcription process that includes resolution of personal issues which arose as a consequence of the work.

•Transcriber would be encouraged to journal her/his thoughts and feelings which may then become part of fieldwork notes.

Follow up

•Follow transcriber up with courtesy call (if transcriber consents)

OR

•Encourage the transcriber to call if s/he experiences increased distress in the hours/days following transcription

For peer review only

Reporting checklist for qualitative study.

Based on the SRQR guidelines.

Instructions to authors

Complete this checklist by entering the page numbers from your manuscript where readers will find each of the items listed below.

Your article may not currently address all the items on the checklist. Please modify your text to include the missing information. If you are certain that an item does not apply, please write "n/a" and provide a short explanation.

Upload your completed checklist as an extra file when you submit to a journal.

In your methods section, say that you used the SRQRreporting guidelines, and cite them as:

O'Brien BC, Harris IB, Beckman TJ, Reed DA, Cook DA. Standards for reporting qualitative research: a synthesis of recommendations. Acad Med. 2014;89(9):1245-1251.

		Reporting Item	Page Number
**Title**			
	#1	Concise description of the nature and topic of the study identifying the study as qualitative or indicating the approach (e.g. ethnography, grounded theory) or data collection methods (e.g. interview, focus group) is recommended	1
**Abstract**			
	#2	Summary of the key elements of the study using the abstract format of the intended publication; typically includes background, purpose, methods, results and conclusions	2
**Introduction**			
Problem formulation	#3	Description and signifcance of the problem / phenomenon studied: review of relevant theory and empirical work; problem statement	4-5

For peer review only

Purpose or research question	#4	Purpose of the study and specific objectives or questions	5
**Methods**			
Qualitative approach and research paradigm	#5	Qualitative approach (e.g. ethnography, grounded theory, case study, phenomenolgy, narrative research) and guiding theory if appropriate; identifying the research paradigm (e.g. postpositivist, constructivist / interpretivist) is also recommended; rationale. The rationale should briefly discuss the justification for choosing that theory, approach, method or technique rather than other options available; the assumptions and limitations implicit in those choices and how those choices influence study conclusions and transferability. As appropriate the rationale for several items might be discussed together.	6
Researcher characteristics and reflexivity	#6	Researchers' characteristics that may influence the research, including personal attributes, qualifications / experience, relationship with participants, assumptions and / or presuppositions; potential or actual interaction between researchers' characteristics and the research questions, approach, methods, results and / or transferability	6
Context	#7	Setting / site and salient contextual factors; rationale	5
Sampling strategy	#8	How and why research participants, documents, or events were selected; criteria for deciding when no further sampling was necessary (e.g. sampling saturation); rationale	5-6
Ethical issues pertaining to human subjects	#9	Documentation of approval by an appropriate ethics review board and participant consent, or explanation for lack thereof; other confidentiality and data security issues	7
Data collection methods	#10	Types of data collected; details of data collection procedures including (as appropriate) start and stop dates of data collection and analysis, iterative	5-6

For peer review only

		process, triangulation of sources / methods, and modification of procedures in response to evolving study findings; rationale	
Data collection instruments and technologies	#11	Description of instruments (e.g. interview guides, questionnaires) and devices (e.g. audio recorders) used for data collection; if / how the instruments(s) changed over the course of the study	6
Units of study	#12	Number and relevant characteristics of participants, documents, or events included in the study; level of participation (could be reported in results)	5-6
Data processing	#13	Methods for processing data prior to and during analysis, including transcription, data entry, data management and security, verification of data integrity, data coding, and anonymisation / deidentification of excerpts	6
Data analysis	#14	Process by which inferences, themes, etc. were identified and developed, including the researchers involved in data analysis; usually references a specific paradigm or approach; rationale	6-7
Techniques to enhance trustworthiness	#15	Techniques to enhance trustworthiness and credibility of data analysis (e.g. member checking, audit trail, triangulation); rationale	7
**Results/findings**			
Syntheses and interpretation	#16	Main findings (e.g. interpretations, inferences, and themes); might include development of a theory or model, or integration with prior research or theory	7-12
Links to empirical data	#17	Evidence (e.g. quotes, field notes, text excerpts, photographs) to substantiate analytic findings	7-12
**Discussion**			
Intergration with prior work, implications, transferability and contribution(s) to the field	#18	Short summary of main findings; explanation of how findings and conclusions connect to, support, elaborate on, or challenge conclusions of earlier scholarship; discussion of scope of application /	12-14

For peer review only

		generalizability; identification of unique contributions(s) to scholarship in a discipline or field	
Limitations	#19	Trustworthiness and limitations of findings	14
**Other**			
Conflicts of interest	#20	Potential sources of influence of perceived influence on study conduct and conclusions; how these were managed	3
Funding	#21	Sources of funding and other support; role of funders in data collection, interpretation and reporting	3

None The SRQR checklist is distributed with permission of Wolters Kluwer © 2014 by the Association of American Medical Colleges. This checklist can be completed online using https://www.goodreports.org/, a tool made by the EQUATOR Network in collaboration with Penelope.ai

For peer review only

BMJ Open

"Our culture prohibits some things": Qualitative inquiry into how sociocultural context influences the scale up of community-based injectable contraceptives in Nigeria

Journal:	*BMJ Open*
Manuscript ID	bmjopen-2019-035311.R2
Article Type:	Original research
Date Submitted by the Author:	03-Apr-2020
Complete List of Authors:	Akinyemi, Oluwaseun; University of Ibadan College of Medicine, Health Policy and Management; University of the Witwatersrand School of Public Health, Community Health<br>Harris, Bronwyn; Warwick Medical School, University of Warwick, Coventry, Division of Health Sciences, ; University of the Witwatersrand, Centre for Health Policy, School of Public Health<br>Kawonga, Mary; University of the Witwatersrand, School of Public Health
**Primary Subject Heading**:	Health policy
Secondary Subject Heading:	Evidence based practice, Health policy, Qualitative research, Public health, Reproductive medicine
Keywords:	Reproductive medicine < GYNAECOLOGY, HEALTH SERVICES ADMINISTRATION & MANAGEMENT, Health policy < HEALTH SERVICES ADMINISTRATION & MANAGEMENT, PREVENTIVE MEDICINE, PUBLIC HEALTH, QUALITATIVE RESEARCH

SCHOLARONE™
Manuscripts

I, the Submitting Author has the right to grant and does grant on behalf of all authors of the Work (as defined in the below author licence), an exclusive licence and/or a non-exclusive licence for contributions from authors who are: i) UK Crown employees; ii) where BMJ has agreed a CC-BY licence shall apply, and/or iii) in accordance with the terms applicable for US Federal Government officers or employees acting as part of their official duties; on a worldwide, perpetual, irrevocable, royalty-free basis to BMJ Publishing Group Ltd ("BMJ") its licensees and where the relevant Journal is co-owned by BMJ to the co-owners of the Journal, to publish the Work in this journal and any other BMJ products and to exploit all rights, as set out in our licence.

The Submitting Author accepts and understands that any supply made under these terms is made by BMJ to the Submitting Author unless you are acting as an employee on behalf of your employer or a postgraduate student of an affiliated institution which is paying any applicable article publishing charge ("APC") for Open Access articles. Where the Submitting Author wishes to make the Work available on an Open Access basis (and intends to pay the relevant APC), the terms of reuse of such Open Access shall be governed by a Creative Commons licence – details of these licences and which Creative Commons licence will apply to this Work are set out in our licence referred to above.

Other than as permitted in any relevant BMJ Author's Self Archiving Policies, I confirm this Work has not been accepted for publication elsewhere, is not being considered for publication elsewhere and does not duplicate material already published. I confirm all authors consent to publication of this Work and authorise the granting of this licence.

For peer review only

"Our culture prohibits some things": Qualitative inquiry into how sociocultural context influences the scale up of community-based injectable contraceptives in Nigeria

Oluwaseun Akinyemi,[a,d*] (ooakinyemi@comui.edu.ng, seunakinyemi@hotmail.com)

Bronwyn Harris,[b,c] (b.harris.2@warwick.ac.uk)

Mary Kawonga,[d] (mary.kawonga@wits.ac.za)

[a] *Department of Health Policy and Management, College of Medicine, University of Ibadan*

[b] *Centre for Health Policy, School of Public Health, Faculty of Health Sciences, University of the Witwatersrand, Johannesburg*

[c] *Division of Health Sciences, Warwick Medical School, University of Warwick, Coventry*

[d] *Department of Community Health, School of Public Health, Faculty of Health Sciences, University of the Witwatersrand, Johannesburg*

Original research article

*Corresponding author: Dr O.O. Akinyemi
Department of Health Policy and Management
College of Medicine, University of Ibadan
Ibadan, Nigeria
+234 803 502 0136

Word counts:

Abstract: 282

Manuscript: 4691

Running Title:

Sociocultural context and innovation scale up

For peer review only

Abstract

Objectives To explore how sociocultural factors may support or impede the adoption of community-based distribution of injectable contraceptives in Nigeria.

Design A qualitative study based on inductive thematic analysis was conducted through in-depth interviews and focus group discussions.

Setting Most participants lived in Gombe State, North East Nigeria. Other participants were from Ibadan (South West) and Abuja (Federal capital territory).

Participants Through seven key informant interviews, 15 in-depth interviews and 10 focus group discussions, 102 participants were involved in the study.

Methods This study, conducted in 2016 was part of a larger study on scale up of community-based distribution of injectable contraceptives. Qualitative data were collected from traditional and religious leaders, health workers and community members. The data were audio recorded, transcribed and analysed using a thematic framework method.

Results Sociocultural challenges to scale up included patriarchy and men's fear of losing control over their spouses, traditional and religious beliefs about fertility, and myths about contraceptives and family planning. As a result of deep-rooted beliefs that children are 'divine blessings' and that procreation should not be regulated, participants described a subtle resistance to uptake of injectable contraceptives. Since Gombe is largely a patriarchal society, male involvement emerged as important to the success of meaningful innovation uptake. Community leaders largely described their participation in the scale up process as active, although they also identified scope for further involvement and recognition.

Conclusion Scale up is more than setting up health sector implementing structures, training health workers and getting innovation supplies, but also requires preparedness which includes paying attention to complex contextual issues. Policy implementers should also see scale up as a learning process and be willing to move at the speed of the community.

.

Strengths and limitations of this study

- The study participants represented a range of stakeholders - users of injectable contraceptives, community members, providers and health system managers.

For peer review only - http://bmjopen.bmj.com/site/about/guidelines.xhtml

- The study provided stakeholders who participated a distinct experience and some observations to reflect upon since the study was based on a current program, rather than speaking merely about theoretical responses they or their community members may have to a health intervention.
- Our results highlighted that scale up is influenced by several socio-cultural factors; thus, showing the importance of paying attention to complex contextual issues during innovation uptake.
- The findings of our study emphasized how health systems and communities should interact in order to ensure successful scale up of health innovations.
- Social desirability bias is a possibility as some of the participants in this study were involved in the community-based distribution intervention

Keywords: Nigeria; Scale up; Community-based distribution of injectable contraceptives; Community participation; Male involvement; Stakeholder collaboration; Sociocultural context

Funding

This research was supported by the Consortium for Advanced Research Training in Africa (CARTA). CARTA is jointly led by the African Population and Health Research Center and the University of the Witwatersrand and funded by the Carnegie Corporation of New York (Grant No--B 8606.R02), Sida (Grant No:54100113), the DELTAS Africa Initiative (Grant No: 107768/Z/15/Z) and Deutscher Akademischer Austauschdienst (DAAD). The DELTAS Africa Initiative is an independent funding scheme of the African Academy of Sciences (AAS)'s Alliance for Accelerating Excellence in Science in Africa (AESA) and supported by the New Partnership for Africa's Development Planning and Coordinating Agency (NEPAD Agency) with funding from the Wellcome Trust (UK) and the UK government. The statements made and views expressed are solely the responsibility of the authors.

Declarations of interest

None.

For peer review only - http://bmjopen.bmj.com/site/about/guidelines.xhtml

1. Introduction

Scale up of effective health innovations, through their increased availability to a larger population over a wider geographic area, is essential for achieving universal health coverage, particularly in developing countries.[1 2] Scale up is influenced by various factors related to the innovation, the users, and the social context in which the innovation is introduced.[3] The nature of the innovation itself, preferences and characteristics of the intended users (including their willingness to adopt the innovation), and ways that the innovation is adapted to the local context are also some of the factors affecting innovation scale up.[4]

The sociocultural context, which includes religious and cultural beliefs, gender and societal norms and attitudes, is an important predictor of adoption of a health innovation in a population.[5 6] The sociocultural context may promote health or be a barrier to healthcare utilization.[7] For example, cultural beliefs and practices have been shown to influence the uptake of a range of health innovations including male circumcision,[8] breast cancer screening[7 9] and contraceptives.[10 11] Without due consideration of the sociocultural context, health innovation development, delivery and scale up is likely to be limited, and this may lead to waste of scarce health resources including time.[12] According to Bradley *et al,*[3] assessing the receptivity and willingness of community members to adopt a health innovation within their environmental and sociocultural settings is one of the key first steps in the scale up process. In their conception, poor understanding by implementers of potential users' sociocultural context will limit scale up efforts, while good understanding will help program managers to recognise contextual factors that may enhance or inhibit the uptake of an innovation.[3]

Evidence highlights the influence of sociocultural context on the adoption of contraceptive innovations. For example, in a study in rural Poland, Colleran and Mace[13] reported that religious and sociocultural factors, particularly social networks, influence the uptake of contraceptive use more than individual characteristics. Dalaba and colleagues[14] in their study in northern Ghana reported that inadequate male participation led to resistance towards female reproductive freedom. Interventions at societal level, such as adequate male mobilisation coupled with appropriate community-based distribution strategies were shown to be effective in improving contraceptive uptake.[14] In Nigeria, where society is largely patriarchal, with consequences for access to and use of contraceptives, a study found that about two-thirds of rural women who had spousal approval were reported to use

For peer review only - http://bmjopen.bmj.com/site/about/guidelines.xhtml

contraceptives while all those who were forbidden by their husbands did not use any family planning method.[15] According to the last Demographic and Health Survey in Nigeria, contraceptive prevalence rate was 14.3% while only 2.4% of women of reproductive age group use injectable contraceptive. Understanding whether and how the sociocultural context influences scale up of contraceptive innovation is thus important.

Evidence suggests that community-based distribution of contraceptives is still needed in developing countries particularly rural or isolated communities in order to increase contraceptive prevalence rate.[16-18] The full potential of CBD to improve CPR has not been fully realized in Sub-Saharan Africa.[19] Nigeria started its family planning policy review in 2007 and successfully piloted the community-based distribution of injectable contraceptives through community health extension workers (CHEWs) as a new innovation in the health system in 2010 in Gombe State, North-eastern region.[20 21] Afterwards, the scale up of this innovation started in 2014, led by a national non-governmental organization and supported by international donor agencies.[22]

For peer review only

However, the understanding of how sociocultural contextual factors may facilitate or impede the successful scale up of the contraceptive innovation beyond the initial pilot site in Nigeria is limited. This study took place after scale up started, thus it adopted both a retrospective slant to assess factors that affected scale up and forward-looking approach to what may affect future scale up effort (see study timeline in Figure 1). The objective of this study is therefore to explore how sociocultural factors may influence the scale up of the community-based distribution of injectable contraceptives in Nigeria.

[Figure 1]

2. Methods

2.1 Study design and setting

This cross-sectional study conducted from September to November 2016, was part of a larger research project[23 24] which explored the interplay between barriers and facilitators of scale up of the community-based distribution of injectable contraceptives guided by the AIDED model[3] in order to inform wider scale up of this innovation in Nigeria. The project took place

For peer review only - http://bmjopen.bmj.com/site/about/guidelines.xhtml

in Gombe (North eastern Nigeria), one of 36 States in Nigeria and a predominantly Muslim community. Gombe State, with a population of 2,353,879 people according to the 2006 population census,[25] has one of the lowest contraceptive prevalence rates in the country (3.5% for modern methods and 4.0% for any method) and one of the highest maternal mortality rates (1,726/100,000).[20 26 27] Gombe was purposively selected because it was the setting for the national pilot test for community-based distribution of injectable contraceptives in 2010. Gombe is divided administratively into 11 Local Government Areas (LGAs). The study was conducted in two LGAs – Gombe (A) and Yamaltu/Deba (B). Although participants were primarily based in Gombe State, one interview took place in another state (Ibadan in the South west) and 3 interviews in Abuja at national level (Federal Capital Territory).

Participants and sample

In total, 102 people participated in this study (see Table 1). The participants were selected through a purposive sampling method, where study respondents were recruited based on characteristics of interest, availability, and ability to provide relevant information[28 29] about the research question: how may sociocultural context influence the process of introducing, translating and integrating health innovation? Participants included a range of stakeholders, including traditional and religious leaders, health workers as well as community members. One FGD each was conducted with different types of participants who were involved and could give an account based on their experiences of the program with the aim of exploring qualitatively the views, concerns from this range of key role players, not aiming to quantify, compare or rank frequency of views. Each of the FGDs had eight participants. The selected participants were therefore deemed appropriate role players to share their own experience and views as well as reflect on and summarize the views of others about the implementation of the community-based distribution of injectable contraceptives within their sociocultural context. The number of interviews and FGDs was determined by the principles of saturation, a point where no new information is emerging,[30 31] and availability of resources as well as study time.[32] The interviews and FGDs were conducted by the first author assisted by three trained research assistants.

This study adopted the inductive thematic analysis. This approach was guided by available literature as well as insights from the participants – codes were generated from key themes identified from transcripts.[30 33-35]

For peer review only - http://bmjopen.bmj.com/site/about/guidelines.xhtml

[Table 1]

2.2 Data collection

Data were collected through seven key informant interviews (KIIs), 15 in-depth interviews (IDIs) and 10 focus group discussions (FGDs). KIIS, IDIs and FGDs were conducted with senior stakeholders, healthcare workers and community members, respectively. Guides were used to conduct the interviews and focus group discussions (see Supplementary file 1). All guides were translated to the predominant local language - Hausa. Interviews with key stakeholders covered issues of program planning and design, community entry, and resolution of challenges with implementation. However, FGDs dealt with issues of program implementation, community members preferences with innovation delivery and packaging. Nonetheless, some issues covered in the interviews and discussions (FGDs) were similar. Interviews and discussions were tape-recorded and later transcribed in full and translated where necessary. Transcription was done using a protocol which emphasizes verbatim transcription of recordings. Both transcription and translation were done by the same research associate who is vast in both Hausa and English languages. Back translation was subsequently done by another associate vast in the two languages; the back translations were afterwards compared with the original transcripts in order to ensure that the original meaning is retained.[36]

2.3 Data analysis

Transcripts were analyzed iteratively with the aid of the NVIVO (version 10) software using the inductive thematic approach.[30 31 34 35] Coding were done by the first and last authors independently at first before analysis started; these codes were then compared and harmonized. Emerging codes from the transcripts were added to pre-defined themes: community members' perceptions regarding community-based distribution of injectable contraceptives, community members' acceptance (or rejection) of contraceptives, and community engagement in the scale up process. Emerging themes were indexed and compared with themes from subsequent interviews until saturation was attained. A codebook was developed after all the data were collected; this was then applied to the transcripts. Although number of interviews and FGDs were set at the outset based on empirical guidance,

For peer review only - http://bmjopen.bmj.com/site/about/guidelines.xhtml

however during analysis, it was confirmed that saturation,[30] where no new information was obtained, was actually reached before the cap was reached in both interviews and FGDs. Analysis progressed alongside data collection. The SRQR reporting guidelines for qualitative studies[37] was used in preparing this manuscript.

2.4 Ethics approval and consent to participate

Ethical approval for this study was obtained from the University of Ibadan/University College Hospital Ethical Review Board (Reference No.: UI/EC/16/0022) as well as the Human Research Ethics Committee (Medical) of the University of the Witwatersrand (Reference No.: M160737). Written informed consent were obtained from study participants before the interviews and FGDs (see Supplementary file 2). Participants were also assured of confidentiality by ensuring that all identifiers were removed from the data and that only the research team has access to the data.

2.5 Patient and Public Involvement

The study participants were involved in shaping the study particularly during the pre-test which led to the modification of some ambiguous questions in the guides. Also, some participants helped with the recruitment of other respondents through a snowball sampling[38] procedure. Furthermore, findings from this study and the larger research project have been presented in conferences with useful feedback; a policy brief has been developed to be disseminated to policy makers.

3. Results

Of the 102 study participants, 59 (57.8%) were female (Table 1) with a mean age of 42.8 ± 14.2 years. Similarly, mean ages of FGD and interview participants were 42.7 ± 14.6 and 46.7 ± 6.0 years, respectively. Six of the 10 FGDs were made up of women participants, each FGD comprising of eight respondents. Participants described a number of sociocultural factors that challenged the scale up process right from the pilot stage; these include traditional and religious norms about fertility, perception about family planning including adverse effects as well as patriarchy and men’s fear of losing control of their spouses (Figure 2).

[Figure 2]

For peer review only - http://bmjopen.bmj.com/site/about/guidelines.xhtml

3.1 Deep rooted cultural and religious beliefs about fertility

Interview participants spoke about a deep-rooted belief amongst community members that children are 'divine blessings' and that procreation should not be regulated. Related to this world view, participants described a subtle resistance to the uptake of injectable contraceptives, more passive than overt in its expression by individuals and families who would feign acceptance but refuse the intervention when given.

> "*…there will always be resistance to this use of contraceptives, because here, we are people that believe that children are a blessing, so you give birth to as many as possible. …The resistance may not be as a group, but when you come to individual levels… as a government, they might not show you the resistance, but when it comes to the actual usage, that's where you start having problems*." (IDI, male, Doctor, SMoH)

Also, there were issues about religious beliefs influencing perception about family planning/ contraceptive use at the community level. Religiosity, that is, strong adherence to religious beliefs, was said to be one of the major factors impeding the uptake of contraceptives in general.

> "*…when you look at northern Nigeria, majority are Muslims and when you look at the south eastern states, you know we have issue of religion, issue of uptake of all contraceptives, not even injectables alone. To the south eastern man, most of them are Catholics, they frown at family planning, so also here (northern Nigeria) people frown at family planning in general*" (KII, male, Senior official, SMoH).

3.2 Awareness and perceptions regarding family planning

Most interview respondents agreed that there was a need to continue to educate community members about the overall benefits of contraceptives and family planning as well as emphasize possible side effects of contraceptives use among women. Also, informants were of the opinion that lack of knowledge about benefits of contraception contributes to reluctance to adopt the innovation. In the words of a respondent:

> "*…the community and people at the grassroots have little information (on contraceptives)*" (KII, female, Senior official, SMoH).

For peer review only - http://bmjopen.bmj.com/site/about/guidelines.xhtml

These challenges were further compounded by reports of adverse effects of the contraception from some women especially "*severe bleeding during menstruation*" (IDI, female, CHEW). Healthcare providers felt that to effectively deal with these challenges, it was important to listen sympathetically to the women and treat reported side effects, in line with the training received for the innovation scale up. Education was also given when clients had questions in order to resolve doubts about the innovation. For example, a respondent said:

> "*We listen to them, and we explain to them that since it (contraceptive) is a new thing in the body, they must experience such things…*" (IDI, female, Nurse).

Focus group discussants highlighted many reasons why women in their reproductive age group used injectable contraceptives, including desire to stay attractive in order to prevent husbands from 'straying', space the children and enjoy life with fewer financial and parenting constraints. One of the discussants who currently uses injectable contraceptives described her motivation thus:

> *"…If we space our children well, we will enjoy our lives because our husbands will always desire us since we are always looking attractive. He will not stay out late because he will be thinking of you at home*" (FGD, female, current user).

Also, a participant stated that the impetus for contraceptive use was the fact that spacing her children afforded her the ability to take care of herself and the home thereby promoting hygiene and healthy living in the home. She said:

> "*…You will be able to take care of yourself as well as your house, your husband will also desire you as you will look good as well as your children. Flies will not follow you around and anyone that comes to your house will be able to eat and drink because everywhere is clean and attractive"* (FGD, female, current user).

3.3 Perceptions regarding the community-based distribution (CBD) approach

During the FGDs, women who were current users of injectable contraceptives stated their preference for the community-based approach over the facility-based system of distributing the contraceptives. Some of their reasons for this position included convenience, affordability (saves cost as transport fare is not needed), improved privacy and focused attention from the health worker.

For peer review only - http://bmjopen.bmj.com/site/about/guidelines.xhtml

> "*…During home visit, it's between you and the woman (health worker) and she can explain it very well to you since she is not in a hurry; although in the hospital they can also explain but the home visit is better since the health worker will explain until you understand.*" (FGD, female, current user).

Likewise, some male discussants preferred the community-based approach because they believed it was more confidential and gave women more time to deal with domestic issues. The CBD approach was also believed to align with men's desire to be in control of 'family issues'.

> *"…Maybe the time the wife wants to go (to the health facility) the husband needed her for something else…Sometimes some husbands want all these things to be confidential, but when you now tell them to release their wives to the hospital, they will think you are exposing the family issues*" (FGD, married men).

However, one of the discussants who favoured the facility-based approach *'prefer[ed] to go to the hospital, so I can be checked properly, including to check my BP*" (FGD, female, current user). Similarly, some married men also preferred the facility-based approach as they believed that the community-based method is intrusive of their privacy and undermines their authority:

> *"…My advice is that the woman should come to hospital …because some husbands will query you that who gave you the permission to see their wives (at home) and advise them about child spacing. Some disagree with the community-based method*" (FGD, married men).

3.4 Spousal involvement in family planning

In addition, male involvement was highlighted as important if uptake of innovation will be meaningful since Gombe is largely a society where the women need the men's permission for doing many things including obtaining injectable contraceptives.

> "*Some men will not accept it (contraceptive) because they want plenty children…she (the wife) should continue begging the husband until he agrees if she wants to use contraceptives*" (FGD, women not using contraceptives).

Furthermore, cultural beliefs and sociocultural context were reported as fuelling poor awareness and knowledge about contraceptive use. Also, male involvement was described as

For peer review only - http://bmjopen.bmj.com/site/about/guidelines.xhtml

For peer review only

important in dealing with the problem of ignorance and poor perception about family planning in the community. According to the participants in this study, once the men are well informed, it is easier to get their buy-in and cooperation in permitting their wives to get the injections.

> "*…some of the men don't know much about family planning commodities and some (people) are telling them some of the side effects. So, they prevent the women from using them…that's why we need more sensitization to the men and the youths*" (IDI, female, Nurse).

> *"The perception of men (about contraceptives) is lower than that of women. The women know the implications better than the men. The men in the rural areas don't mind having any number of children...*" (FGD, married men)

Also, a female health worker emphasised the importance of male involvement particularly in communities where the literacy rate is low:

> *"Honestly. we need to do more in carrying them [men] along. Male involvement is very important because you know they are our superiors. We in the North, it is only the educated that may be sometimes at loggerhead (with their husbands) … but these illiterates and native people, it is always the man that is superior. So, it is very important for us to consider male involvement, it will help a lot* (IDI, female, Nurse).

3.5 Community support for scale up

The issue of community support for scale up was solely explored with community and religious leaders. The traditional leaders interviewed believed that community support for the innovation is very high.

> "*In fact, the support our community gives to this programme, without exaggeration, I will say maybe 80-90%. They (the community) have been very supportive of the programme*" (IDI, male, Traditional ruler, Yemaltu-Deba).

> "*Definitely, you have 70% support from the communities. From the religious leaders, traditional rulers and even of ward heads in the community we get 100% support"* (IDI, male, Traditional leader, Gombe).

However, some cautioned that the community support is not as high as expected due to what

For peer review only - http://bmjopen.bmj.com/site/about/guidelines.xhtml

For peer review only

they call low knowledge and awareness about contraceptive choices in Northern Nigeria.

> "*…Really, people have supported the program but I know some people do not have the knowledge (about injectable contraceptives) but those that have the knowledge are really in support of the program…* [you know] *the Northern culture prohibits some things, that's why I said the support for it (injectable contraceptives scale up) is not as much as we expect*." (IDI, male, Christian religious leader)

One community leader described his role as active in the scale up process because he helps in disseminating information to all the wards under his domain. He shared his experience:

> "*Whenever the need arises, we normally call the kingmakers, district head kingmakers, they are about seven, and we have at least 54 ward heads, we use to call them from time to time and enlighten them on the programme. In fact, almost all the community leaders are fully aware of the programme*" (IDI, male, Traditional leader).

Many of the leaders ascribed the progress with the scale up in their communities to the involvement of the traditional leaders in the programme. However, some leaders complained of not being officially involved in the programme, although they still offer their support.

> "*There are so many traditional rulers that were involved in this program; that is the reason why it has been accepted 100%, especially in Gombe State now*" (IDI, male, Traditional leader, Gombe).

> "*…My involvement is…passive involvement. Of course, we preachers, you can push people, encourage them in your own little way... We preach it, we tell people about it…* (IDI, male, Christian religious leader)

4. Discussion

In our study, there were a number of sociocultural issues alluded to as factors that may affect scale up of a contraceptive innovation in Nigeria, including patriarchy, traditional and religious norms, beliefs and myths about contraceptives and family planning. Agunbiade & Ogunleye[39] reported in their study assessing the sociocultural barriers to the scale up of exclusive breastfeeding in Nigeria, the scale up process cannot be separated from the context and the culture in the environment. Further, in a study among African American women, culture and health beliefs were found to be significant predictors of health innovation

For peer review only - http://bmjopen.bmj.com/site/about/guidelines.xhtml

uptake.[40]

Our study indicates it is vital to secure the understanding and support of the men and significant others for scale up of the community-based distribution of injectable contraceptives to succeed in environments like our study site that are still largely patriarchal.[39] Similar to the findings of our study, male involvement and support of women's significant others, particularly the mothers-in-law, have been reported to be crucial to innovation scale up in Nigeria as these significant others have strong influence on choices made by women.[39] In other settings, increased male participation through increasing men's knowledge and reducing their opposition has been shown to improve uptake and continued use of contraceptives[41-43] and enhancement of prevention of mother to child transmission of HIV (PMTCT) scale up processes.[44] In this study, one of the reasons why women reportedly use contraceptives was to look attractive and satisfy their husbands in order to prevent them from engaging in extramarital relationships. Thus, contraception is both liberating for women on one hand but also perhaps serving the very patriarchy they are trapped in on the other. Studies have shown that in many African societies, patriarchy is perpetuated by the socialisation process which starts from the family and is often preserved by women themselves.[45 46]

Also, findings from our study show passive resistance to contraceptive uptake, particularly at the individual levels, by both women and their spouses. Research has shown that passive resistance is one of the major reasons for the high failure rate of innovation scale up.[47 48] Passive resistance often results from individuals' resistance to change and contentment with or lack of power to change the status quo.[48] Further, consumers will not adopt an innovation if they are not sure of its value or benefits.[49] According to Heidenreich and Kraemer, passive innovation resistance should be addressed before the introduction of new innovations into the society.[47] Strategies for reducing passive resistance to uptake of health innovations in the community include actively involving men in their uptake,[14] as well as giving adequate and correct information about the advantages of contraceptive use to all stakeholders - health workers, community and religious leaders, trained volunteers and program managers.[49]

Since the passive resistance reported in this study is largely underpinned by deep-seated issues of patriarchy, religious beliefs and traditional norms/values, it is imperative for policy makers and program managers to engage men – who are custodians, in most cases, of both religious and cultural values.[50 51] Male involvement can bring benefits for all community

For peer review only - http://bmjopen.bmj.com/site/about/guidelines.xhtml

stakeholders,[50] as well as social change.[52] With careful management and ongoing monitoring, the involvement of men may ultimately give the scale up of this health innovation a greater chance of success.

Furthermore, religious beliefs were also found to influence scale up of the community-based injectable contraceptives and family planning in general in our study sites in Northern Nigeria. Religion has been shown to significantly influence decisions on contraceptive use,[53] as well as individual/household choices, lifestyle and even traditional values.[54] Thus, religion has great implications for the social marketing of commodities particularly contraceptives, as uptake of health products has been shown to differ significantly among adherents of major religions.[54] For example, research done in Islamic countries[55 56] highlights the need to understand Islamic religion in order to do effective social marketing, and recommends that approval be obtained from religious leadership before certain sensitive products like contraceptives are marketed. Understanding religious nuances in local contexts is important in multi-faith countries such as Nigeria – for example, what 'works' in the largely-Islamic North[57] may need adaptation for Christian populations elsewhere.

Our study demonstrated the importance of strong community support and partnership with community gate-keepers in the scale up process. Research has shown that the CBD approach cannot succeed without community participation.[58] Therefore, programme implementers need to learn from the community what works (or may work) in the setting, what distribution method the community prefers or what modification to the existing methods they favour. Research has shown that there is no quick fix to scaling up.[58] It is essentially a learning process which is unique in different contexts. Therefore, scale up may not be effective without adequate contextual knowledge and willingness to learn through the process of community participation and stakeholder collaboration.[58 59]

While the focus of this paper has been on challenges as identified in the wider context of religion and patriarchy, many of the suggested solutions rely on the health systems and those working in it, to navigate these challenges and overcome them. However, this cannot be a static or reactive approach but rather needs to recognise that health systems and those working in them are also part of the sociocultural context and therefore it is important to find ways to support healthcare workers in mediating, translating and promoting the innovation in this context.

For peer review only - http://bmjopen.bmj.com/site/about/guidelines.xhtml

4.1 Limitations of the study

Some limitations of this study should be emphasised. As with any qualitative study, the findings are not statistically generalizable but the theories and ideas can be applied to other contexts. Also, some of the participants in this study were involved in the community-based distribution intervention, therefore they may provide more socially desirable answers. However, a strength of this study is that it provided stakeholders who participated a distinct experience and some observations to reflect upon since the study was based on a current program, rather than speaking merely about theoretical responses they or their community members may have to a health intervention. Thus, the findings of this research provide valuable information into how sociocultural context and user support affect the scale up of health innovations in LMICs, particularly in a patriarchal society like Nigeria.

4.3 Conclusion

Our study suggests the need for continuous community education and engagement with the significant others and community leadership structures – both traditional and religious. Thus, providing an innovation does not necessarily translate to uptake. Therefore, program implementers should also consider sociocultural and health system contexts when packaging a health innovation. Hence, supporting those in the health system to mediate the innovation in ways that are sensitive to the context but which, by working with communities and others, promotes transformation is imperative. In addition, incorporating the CBD approach with other outreach programmes, developing a more effective supervision of the approach may help make CBD more productive. Interventions to improve male participation will likewise go a long way to improve the scale up process. Policy implementers should also see scale up as a learning process and be willing to move at the speed of the community.

Acknowledgements

We are grateful to Drs Mariya Saleh, Wole Adefalu, Edward Oladele and Hadiza Kamofu for expediting access to key informants. Also, we appreciate Dr Alex Eze for his feedback on the early draft of the manuscript. We are grateful for the support of Dr Busola Adebayo, Jimi Latunji and Wunmi Folajimi-Senjobi with editing the manuscript. We would like to thank all the participants in this study.

Authors' contributions

OA conceived the study; OA and MK designed it. The data was collected by OA. OA, MK and

For peer review only - http://bmjopen.bmj.com/site/about/guidelines.xhtml

BH drafted and commented on the draft of the manuscript. OA, MK and BH read and approved the final version of the manuscript.

Data Availability Statement

Extra data can be accessed via the Dryad data repository at http://datadryad.org/ with the doi: 10.5061/dryad.6q573n5w8

For peer review only

References

1. Subramanian S, Naimoli J, Matsubayashi T, et al. Do we have the right models for scaling up health services to achieve the Millennium Development Goals? *BMC health services research* 2011;11(1):336.
2. Manham L, Hanson K. Scaling up in international health: what are the key issues? Review. *Health Policy Plan* 2010;25:85-96.
3. Bradley EH, Curry LA, Taylor LA, et al. A model for scale up of family health innovations in low-income and middle-income settings: a mixed methods study. *BMJ open* 2012;2(4):e000987.
4. Hecht R, Stover J, Bollinger L, et al. Financing of HIV/AIDS programme scale-up in low-income and middle-income countries, 2009–31. *The Lancet* 2010;376(9748):1254-60.
5. Johns B, Torres TT. Costs of scaling up health interventions: a systematic review. *Health policy and planning* 2005;20(1):1-13.
6. Mangham LJ, Hanson K. Scaling up in international health: what are the key issues? *Health policy and planning* 2010;25(2):85-96.
7. Rajaram SS, Rashidi A. Asian-Islamic women and breast cancer screening: a socio-cultural analysis. *Women & health* 1999;28(3):45-58.
8. Westercamp N, Bailey RC. Acceptability of Male Circumcision for Prevention of HIV/AIDS in Sub-Saharan Africa: A Review. *AIDS and Behavior* 2007;11(3):341-55. doi: 10.1007/s10461-006-9169-4
9. Katapodi MC, Lee KA, Facione NC, et al. Predictors of perceived breast cancer risk and the relation between perceived risk and breast cancer screening: a meta-analytic review. *Preventive medicine* 2004;38(4):388-402.
10. Muanda M, Ndongo PG, Taub LD, et al. Barriers to modern contraceptive use in Kinshasa, DRC. *PloS one* 2016;11(12):e0167560.
11. Muanda MF, Ndongo GP, Messina LJ, et al. Barriers to modern contraceptive use in rural areas in DRC. *Culture, health & sexuality* 2017;19(9):1011-23.
12. Airhihenbuwa CO. Health and culture: Beyond the Western paradigm: Sage 1995.
13. Colleran H, Mace R. Social network-and community-level influences on contraceptive use: evidence from rural Poland. *Proc R Soc B* 2015;282(1807):20150398.
14. Dalaba MA, Stone AE, Krumholz AR, et al. A qualitative analysis of the effect of a community-based primary health care programme on reproductive preferences and contraceptive use among the Kassena-Nankana of northern Ghana. *BMC health services research* 2016;16(1):80.
15. Etukudo IW. Spousal approval, communication and contraceptive behaviour in rural Nigeria. *African Journal of Midwifery and Women's Health* 2015;9(4):170-76.
16. Prata N, Gessessew A, Cartwright A, et al. Provision of injectable contraceptives in Ethiopia through community-based reproductive health agents. *Bulletin of the World Health Organization* 2011;89:556-64.
17. Prata N, Vahidnia F, Potts M, et al. Revisiting community-based distribution programs: are they still needed? *Contraception* 2005;72(6):402-07.
18. Gichane MW, Mutesa M, Chowa G. Translating Evidence into Policy Change: Advocacy for Community-Based Distribution of Injectable Contraceptives in Zambia. *Global Social Welfare* 2019;6(1):41-47.
19. Hoke T, Brunie A, Krueger K, et al. Community-based distribution of injectable contraceptives: introduction strategies in four sub-Saharan African countries. *International perspectives on sexual and reproductive health* 2012;38(4):214-19.
20. FHI. The Effectiveness of Community-Based Access to Injectable Contraceptives in Nigeria: A Technical Report. 2010

For peer review only - http://bmjopen.bmj.com/site/about/guidelines.xhtml

21. Association for Reproductive and Family Health. Increasing access to contraceptives in Nigeria through task-shifting to Community Health Extension Workers (CHEWs) Abuja: Association for Reproductive and Family Health; 2015 [cited 2015 6 January]. Available from: http://www.arfh-ng.org/news/item012.php accessed 6 January 2015.
22. Association for Reproductive and Family Health. Increasing Access to Contraceptives in Nigeria Through Task-shifting to Community Health Extension Workers (CHEWS) Ibadan: Association for Reproductive and Family Health,; 2014 [cited 2015 5 November]. Available from: http://arfh-ng.org/increasing-access-contraceptives-nigeria-task-shifting-community-health-extension-workers-chews/ accessed 5 November 2015.
23. Akinyemi O, Harris B, Kawonga M. Health system readiness for innovation scale-up: the experience of community-based distribution of injectable contraceptives in Nigeria. *BMC Health Services Research* 2019;19(1):1-11.
24. Akinyemi O, Harris B, Kawonga M. Innovation diffusion: how homogenous networks influence the uptake of community-based injectable contraceptives. *BMC public health* 2019;19(1):1520.
25. National Population Commission. Population and housing census of the Federal Republic of Nigeria. *Priority tables* 2006;1
26. National Population Commission. Nigeria demographic and health survey 2008. *Nigeria and ICF Macro* 2008
27. National Population Commission, ICF International. Nigeria Demographic and Health Survey 2013. *Abuja, Nigeria* 2014
28. Vonk ME, Tripodi T, Epstein I. Research techniques for clinical social workers: Columbia University Press 2007.
29. Pope C, Mays N. Qualitative research: reaching the parts other methods cannot reach: an introduction to qualitative methods in health and health services research. *Bmj* 1995;311(6996):42-45.
30. Temple B, Edwards R. Interpreters/translators and cross-cultural research: Reflexivity and border crossing. *International Journal of Qualitative Methods* 2002;1(2)
31. Guest G, Bunce A, Johnson L. How many interviews are enough? An experiment with data saturation and variability. *Field methods* 2006;18(1):59-82.
32. Namey E, Guest G, McKenna K, et al. Evaluating bang for the buck: a cost-effectiveness comparison between individual interviews and focus groups based on thematic saturation levels. *American Journal of Evaluation* 2016;37(3):425-40.
33. Guest G, MacQueen KM, Namey EE. Introduction to applied thematic analysis. *Applied thematic analysis* 2012;3:20.
34. Bernard HR. Research methods in anthropology: Qualitative and quantitative approaches: Rowman & Littlefield 2017.
35. Hutchison AJ, Johnston LH, Breckon JD. Using QSR-NVivo to facilitate the development of a grounded theory project: an account of a worked example. *International journal of social research methodology* 2010;13(4):283-302.
36. Van Nes F, Abma T, Jonsson H, et al. Language differences in qualitative research: is meaning lost in translation? *European journal of ageing* 2010;7(4):313-16.
37. O'Brien BC, Harris IB, Beckman TJ, et al. Standards for reporting qualitative research: a synthesis of recommendations. *Academic Medicine* 2014;89(9):1245-51.
38. Johnson TP. Snowball sampling: introduction. *Wiley StatsRef: Statistics Reference Online* 2014
39. Agunbiade OM, Ogunleye OV. Constraints to exclusive breastfeeding practice among breastfeeding mothers in Southwest Nigeria: implications for scaling up. *International breastfeeding journal* 2012;7(1):5.

For peer review only - http://bmjopen.bmj.com/site/about/guidelines.xhtml

40. Sociocultural context of mammography screening use. Oncology nursing forum; 2006. Oncology Nursing Society.
41. Kabagenyi A, Jennings L, Reid A, et al. Barriers to male involvement in contraceptive uptake and reproductive health services: a qualitative study of men and women's perceptions in two rural districts in Uganda. *Reproductive health* 2014;11(1):21.
42. Ujuju C, Anyanti J, Adebayo S, et al. Religion, culture and male involvement in the use of the Standard Days Method: evidence from Enugu and Katsina states of Nigeria. *International nursing review* 2011;58(4):484-90.
43. Sanusi AA, Akinyemi OO, Onoviran OO. Do knowledge and cultural perceptions of modern female contraceptives predict male involvement in Ayete, Nigeria? *African journal of reproductive health* 2014;18(4):105-14.
44. Peltzer K, Jones D, Weiss SM, et al. Promoting male involvement to improve PMTCT uptake and reduce antenatal HIV infection: a cluster randomized controlled trial protocol. *BMC Public Health* 2011;11(1):778. doi: 10.1186/1471-2458-11-778
45. Florence N. Bukusu (Kenya) folktales: how women perpetuate patriarchy. *International Feminist Journal of Politics* 2013;15(3):370-90.
46. Kambarami M. Femininity, sexuality and culture: Patriarchy and female subordination in Zimbabwe. *South Africa: ARSRC* 2006
47. Heidenreich S, Kraemer T. Innovations—doomed to fail? Investigating strategies to overcome passive innovation resistance. *Journal of Product Innovation Management* 2016;33(3):277-97.
48. Heidenreich S, Spieth P. Why innovations fail—The case of passive and active innovation resistance. *International Journal of Innovation Management* 2013;17(05):1350021.
49. KISS Communications. Why Consumers Resist Innovation – part one 2016 [cited 2018 1 September]. Available from: https://www.kisscom.co.uk/news/why-consumers-resist-innovation-part-one accessed 1 September 2018.
50. Orji E, Onwudiegwu U. Contraceptive practice among married market men in Nigeria. *East African medical journal* 2003;80(7):357-60.
51. Orji E, Onwudiegwu U. Contraceptive practice among married men in Nigeria. *East Afr Med J* 2008;200(80):7.
52. Onwukwe LC, Njemanze VC, Ebeh R, et al. Religiosity and Sexism as Indices of People's Perception of Women in Politics in Nigeria. *American Journal of Social Science Research* 2017;3(2):9-16.
53. Mitike G. Community-based distribution of family planning as perceived by people in the reproductive age group, North and South Gondar zones, Ethiopia. *Ethiopian Journal of Health Development* 2000;14(1):31-42.
54. Shyan Fam K, Waller DS, Zafer Erdogan B. The influence of religion on attitudes towards the advertising of controversial products. *European Journal of marketing* 2004;38(5/6):537-55.
55. Michell P, Al-Mossawi M. Religious commitment related to message contentiousness. *International Journal of Advertising* 1999;18(4):427-43.
56. Luqmani M, Yavas U, Quraeshi Z. Advertising in Saudi Arabia: content and regulation. *International Marketing Review* 1989;6(1)
57. Watts MJ. Silent violence: Food, famine, and peasantry in northern Nigeria: University of Georgia Press 2013.
58. Gilson L, Schneider H. Commentary: Managing scaling up: what are the key issues? *Health Policy and Planning* 2010;25(2):97-98.
59. Phillips JF, Greene WL. Community based distribution of family planning in Africa: lessons from operations research. 1993

For peer review only - http://bmjopen.bmj.com/site/about/guidelines.xhtml

Table

Table 1: Participants' sociodemographic characteristics and data collection methods across study sites

Location	Type	No. of interviews/ FGDs	No. of participants	Gender		Participant type
				M n (%)	F n (%)	
**Federal and State Level**						
	KII	4	4	2 (50.0)	2 (50.0)	NGO program manager, Senior official at the Federal MoH[a], NGO program managers
**Gombe State**						
	KII	3	3	2 (66.7)	1 (33.3)	Senior officials of State MoH.
LGA **A**	IDI	7	7	3 (42.9)	4 (57.1)	Health workers (Nurses, CHEWs[b]), traditional and religious leaders
	FGD	5	40	16 (40.0)	24 (60.0)	Current contraceptive users, older women non-users, younger women non-users (in reproductive age group), married and single men
LGA **B**	IDI	8	8	4 (50.0)	4 (50.0)	Health workers (Nurses, CHEWs[b]), traditional and religious leaders
	FGD	5	40	16 (40.0)	24 (60.0)	Current contraceptive users, older women non-users, younger women non-users (in reproductive age group), married and single men
**Total**		**32**	**102**	**43 (42.2)**	**59 (57.8)**	

[a]MoH – Ministry of Health; [b]CHEWs – Community Health Extension Workers

For peer review only - http://bmjopen.bmj.com/site/about/guidelines.xhtml

Figure legends/captions

Figure 1: Situating data collection within the timeline for pilot study and scale up

Figure 2: Socio-cultural factors influencing uptake of CBD of injectable contraceptives in Gombe State

For peer review only

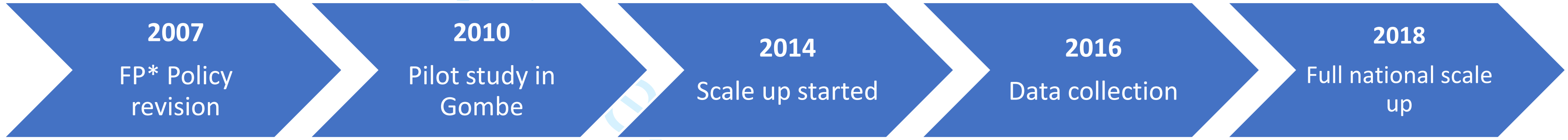

**Family planning*

Figure 1: Situating data collection within the timeline for pilot study and scale up

For peer review only

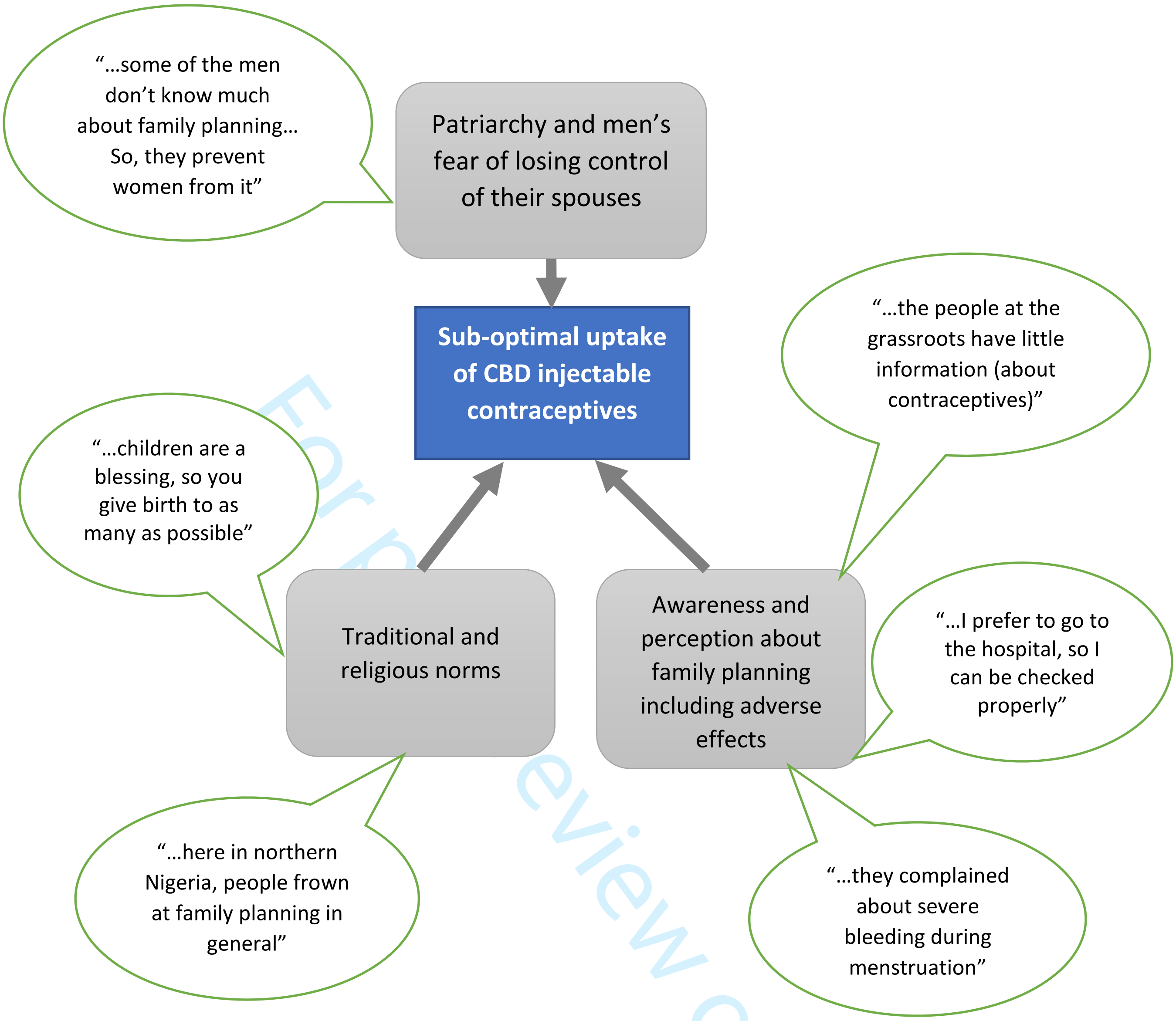

Figure 2: Sociocultural factors influencing uptake of CBD of injectable contraceptives in Gombe State

SUPPLEMENTARY FILE 1

Interview Guide: Key informant interview with senior MOH officials and NGO programme managers

1. Let's start by you describing your role in implementing this intervention – the community-based delivery of injectable contraceptives in the pilot sites in Gombe. What was your role and how long were you involved?

2. Did you encounter any challenges during the pilot study? Please explain.
   Probes:
   - If not mentioned, ask: any resistance from the community members, from health workers, from other stakeholders, from potential users themselves?
   - How did you handle challenges / resistance?

3. We are interested in your experience with the process of taking the intervention from pilot study phase to wider implementation in Gombe itself. What was the process, from implementation to wider scale up of the intervention? Walk me through the process.
   Probes:
   - The goal, approach to implementing, key components of the process, indicators of success
   - Landscape and stakeholder assessment done – before, during or after the pilot?
   - Your role in these processes?

4. In your opinion, what factors have supported the scale up of this intervention so far?

5. Have you experienced challenges during the process of scaling up? Please explain.
   Probes:
   - What challenges? ... ***may prompt with*** "any others"
   - How did you handle challenges?

6. Was there a way of ensuring that the intervention aligns to the wants and preferences of the users you were targeting? Please tell me about it.
   Probes:
   - Any user needs / wants assessment done? By whom? When?
   - Any modifications made in order to align packaging of the innovation to user wants – either during or after the pilot?
   - Any health service modification (e.g. change service models for delivering the intervention)?

7. Have communities been engaged in the scale up process? Please describe in what ways and by whom?

8. Looking back, is there anything that you think could have been done differently to facilitate the process from pilot study to wider implementation? Please explain.

9. Is there anything else we have not asked but you would like to tell us about your experience with the process of implementation and scale-up of CBD?

Thank you for your time. This is the end of our discussion.

For peer review only

In-depth Interview Guides

A. Interview guide: In-depth interview with health workers (Doctors and CHEWs)

1. Let's start by you describing your role in implementing this intervention – the community-based delivery of injectable contraceptives by community health extension workers.
    a. What role did doctors / CHEWs play in designing the intervention?
    b. What about their role in introducing the intervention at community level?
    c. What was your own role?
    Probes:
    - **Doctors:** Your own (or experience of other doctors like you) with: planning, training CHEWs, providing supportive supervision to CHEWs during and after the pilot?
    - **CHEWs**: Your own (or experience of other CHEWs like you) in planning, administering the injectable contraceptives to women?

    d. Did you / other health workers experience challenges?
        i. Please explain.
        ii. How did you handle the challenges?

2. In your opinion, what factors affect wider implementation and uptake of the CBD of injectable contraceptives in the communities that you serve?
    Probes:
    - Anything else?

3. In your opinion, how supportive of this innovation are:
    a. **For doctors**: doctors in general, and your professional health group (e.g. Nigerian Medical Association) please explain.
    b. **For CHEWs**: community health extension workers in general? Please explain.

4. As far as you are aware, what role, if any, have health workers like you played in making the CBD of injectable contraceptives more acceptable to the users in your community? Please explain.

5. In your opinion, how likely are those women who have already adopted the CBD of injectables to spread information about the innovation to other women who are not using it? Please explain.

6. Looking back, is there anything that you feel might have been done differently to facilitate the process of moving from pilot study to wider implementation?

7. Is there anything else we have not discussed that you would like to tell us about your experience with implementing the CBD of injectable contraceptives?

Thank you for your time. This is the end of our discussion.

For peer review only

B. Interview guide: In-depth interview with community leaders

1. You were selected to participate in this study because you are aware of the government program in which CHEWs provide injectable contraceptives to women in the community, outside the health clinic setting.
    a. Please describe what you know about this program.
    b. How did you hear about it?

2. How much support do you think there is for this program in your community? Please explain

3. Have you or other community leaders like you been involved in introducing this program on injectable contraceptives into your community?
    a. ***If yes***: Describe your / or other leaders' involvement (what role)? How did you / they get involved? Who initiated?
    b. How do you feel about being involved / not being involved?

4. The government started with this program only in two places in Gombe but they are now trying to make this available to all other parts of Gombe.
    a. What challenges do you think the government might encounter when trying to get wider uptake of this programme in the community?
    b. Have you or other community leaders in this community been approached by government to assist with addressing these challenges?

5. In your opinion, what might prevent women in your community from taking up this intervention? What may encourage them to take it up?

6. In your opinion, how likely are those women who have already adopted the CBD of injectables to share information about it to other women who are not using it? Please explain?

7. Looking back, is there anything that you feel might have been done differently to make this way of providing contraceptives acceptable in your community?

8. Is there anything else we have not discussed that you would like to tell us about your experience with implementing the CBD of injectable contraceptives?

Thank you for your time. This is the end of our discussion.

For peer review only - http://bmjopen.bmj.com/site/about/guidelines.xhtml

Focus Group Discussion Guides

A. Focus group discussion guide: FGDs with women who have already adopted the use of injectable contraceptives through the CBD approach

1. Let us start with talking about contraceptives / family planning methods in general. Please share with me what you understand about contraception / family planning. What kinds of contraceptive methods do you know?

2. What do you think people in this community think about contraceptives and family planning in general?
   Probe:
   - Do they support the idea of contraception / family planning in general?
   - Specifically, how supportive of injectable contraceptives compared to others?

3. What about women in this community. How do you think they feel about contraceptives and family planning in general?
   Probe:
   - Support the use of contraception / family planning in general?
   - Specifically, how popular are injectable contraceptives compared to others?
   - What makes women use injectable contraceptives? What makes them not use it?

4. I am interested to know what you think women want and prefer regarding contraceptives. Which types of contraceptives do women in your community prefer? How do you think women prefer to receive contraceptives (from clinic versus other places; from doctor or nurse versus others)?

5. The government introduced a new program where community health extension workers now administer injectable contraceptives to women away from the clinic, including at homes. I know that you already receive injectable contraceptives through this approach.
   a. How did you hear about this program?
   b. What made women like you to decide to receive injectables through CHEWs?
   c. Would you encourage friends or family members to receive injectable contraceptives from CHEWs? Please explain.
   d. What do you think might prevent other women in your community from using this new way of receiving injectable contraceptives through CHEWs?

6. What suggestions do you have for how the way injectable contraceptives are administered in your community can be improved to make sure many more women receive them?

Thank you for your time. This is the end of our discussion

For peer review only

B. Focus group discussion guide: FGD with a group of women in the reproductive age group who have not yet adopted the CBD of injectables (may include those who do not use injectables and those who do)

1. Let us start with talking about contraceptives / family planning methods in general. How many contraceptive types do you know? Can you describe them?

2. How do you think people in this community perceive contraceptives and family planning in general?

   Probe:
   - Popularity of contraceptives / the idea of family planning in general
   - Specifically, popularity of injectable contraceptives

3. How do you think women in this community perceive contraceptives and family planning in general?

   Probe:
   - Popularity of contraceptives / family planning in general
   - Specifically, popularity of injectable contraceptives

4. What do women want - which types of contraceptives, which mode of delivery do they prefer? (clinic vs. other places, doctor/nurse versus others)

5. Let us now talk about injectable contraceptives specifically. In your opinion, what factors will make women of reproductive age use or reject injectable contraceptives? What makes them not use it?

6. The government introduced a new program where community health extension workers now administer injectable contraceptives to women away from the clinic, including at homes. I know that you have not received injectable contraceptives through this approach.
   a. How did you hear about this program?
   b. What factors make women like you decide not to receive injectables through CHEWs?
   c. What factors would encourage you to receive the injectable contraceptives through CHEWs?
   d. Willingness to encourage friends and family members of reproductive age to receive injectable contraceptives from CHEWs?
   e. What do you think might prevent other women in your community from using this new way of receiving injectable contraceptives through CHEWs?

   Probes:
   - Resistance to mode of distribution – facility vs. community based
   - Socio-cultural resistance to injectable contraceptives
   - Resistance to injectable contraceptives being administered by CHEWs rather than other health workers like nurses and doctors

7. What suggestions do you have for how the way injectable contraceptives are administered in your community can be improved to make sure many more women like you receive them?

Thank you for your time. This is the end of our discussion.

For peer review only

C. Focus group discussion guide: FGD with elderly women (older than reproductive age) in the community

1. Let us start with talking about contraceptives / family planning methods in general. How many contraceptive types do you know? Can you describe them?

2. How do you think people in this community perceive contraceptives and family planning in general?
   Probe:
   - Popularity of contraceptives / the idea of family planning in general
   - Specifically, popularity of injectable contraceptives
3. What role do you play in advising your married daughters/daughters-in-law and family members of reproductive age about contraception/family planning?

4. How do you think elderly women (grandmothers) in this community perceive contraceptives and family planning in general?
   Probe:
   - Popularity of contraceptives / family planning in general among elderly women (awareness and knowledge, not use)
   - Specifically, popularity of injectable contraceptives

5. What do elderly women prefer? Which types of contraceptives, mode of delivery (clinic vs. other places, doctor/nurse versus others) do they prefer for their married daughters/daughters-in-law

6. Let us now talk about injectable contraceptives specifically. In your opinion, what factors will make women of reproductive age use or reject injectable contraceptives?
7. Will you support your married daughters/daughters-in-law using injectable contraceptives? Please give reasons for your answer.
8. The government introduced a new program where community health extension workers now administer injectable contraceptives to women away from the clinic, including at homes.

   a. How did you hear about this program? In your opinion, what factors help women to decide to receive injectables through CHEWs?
   b. Willingness to encourage married daughters/daughters-in-law and family members of reproductive age to receive injectable contraceptives from CHEWs?
   c. What do you think might prevent women in your community from using injectable contraceptives through CHEWs?
   d. What kinds of challenges do you know the government has faced with the distribution of injectable contraceptives through CHEWs in your community?

   Probes:
   - Resistance to mode of distribution – facility vs. community based
   - Socio-cultural resistance to injectable contraceptives
   - Resistance to injectable contraceptives being administered by CHEWs rather than other health workers like nurses and doctors

8. What suggestions do you have for how the way injectable contraceptives are administered in your community can be improved to make sure many more women receive them?

Thank you for your time. This is the end of our discussion.

For peer review only

D. Focus group discussion guide: FGD with younger and older men (single and married) in the community

1. Let us start with talking about contraceptives / family planning methods in general. How many contraceptive types do you know? Can you describe them?

2. How do you think people in this community perceive contraceptives and family planning in general?
   Probe:
   - Popularity of contraceptives / the idea of family planning in general
   - Specifically, popularity of injectable contraceptives

3. How do you think men in this community perceive contraceptives and family planning in general?
   Probe:
   - Popularity of contraceptives / family planning in general among men
   - Specifically, popularity of injectable contraceptives

4. What do men want - which types of contraceptives, mode of delivery (clinic vs. other places, doctor/nurse versus others) do they prefer for their wives
5. Let us now talk about injectable contraceptives specifically. In your opinion, what factors will make women of reproductive age use or reject injectable contraceptives?
6. Will you support your wife/partner/girlfriend using injectable contraceptives? Please give reasons for your answer.
7. The government introduced a new program where community health extension workers now administer injectable contraceptives to women away from the clinic, including at homes.

   a. How did you hear about this program? In your opinion, what factors help women to decide to receive injectables through CHEWs?
   b. Willingness to encourage wife/partner/girlfriend and family members of reproductive age to receive injectable contraceptives from CHEWs?
   c. What do you think might prevent women in your community from using injectable contraceptives through CHEWs?
   d. What kinds of challenges do you know the government has faced with the distribution of injectable contraceptives through CHEWs in your community?

   Probes:
   - Resistance to mode of distribution – facility vs. community based
   - Socio-cultural resistance to injectable contraceptives
   - Resistance to injectable contraceptives being administered by CHEWs rather than other health workers like nurses and doctors

9. What suggestions do you have for how the way injectable contraceptives are administered in your community can be improved to make sure many more women receive them?

Thank you for your time. This is the end of our discussion.

For peer review only

SUPPLEMENTARY FILE 2

Participant Information Sheet for MOH officials

Study title: Scale up of community-based injectable contraceptives in Gombe State, Nigeria

Introduction:

Good day! I am Oluwaseun Akinyemi, a PhD candidate of the School of Public Health, University of the Witwatersrand, Johannesburg. I am doing research on the scale up of community-based injectable contraceptives in Gombe State, Nigeria. Research is just the process to learn the answer to a question. In this study we want to learn what the factors are that may influence the scale up of community-based distribution of injectable contraceptives in Nigeria, in order to understand possible facilitators and barriers.

Invitation to participate: I am therefore asking / inviting you to take part in this research study because you are a senior MOH official who was/is involved with this programme.

What is involved in the study – I am asking different people such as policy makers, program managers, health workers, community leaders, women who are the end-users of injectable contraceptives, and broader members of the community to participate in this study. About 100 people will participate in the study and all will be from Nigeria. If you agree to participate it means you will take part in a key informant interview. Each interview will last for about 60 minutes. In the interviews I will ask questions about your knowledge and your role in the implementation or use of injectable contraceptives as well as what you think might be the challenges and things that work well in implementing this program. There are no right and wrong answers; I am interested in understanding your opinions. Please note that I intend to audio-tape this interview.

Risks and Benefits of being involved in the study – There are no invasive procedures involved in this study, thus no physical harm is envisaged. However, you don't have to answer any question you are not comfortable with.

There are no direct benefits for participating in this study.

The participant will be given pertinent information on the study while involved in the project and after the results are available.

Participation is voluntary: Refusal to participate will involve no penalty or loss of benefits to which you are otherwise entitled. Also, you may discontinue participation at any time without penalty or loss of benefits to which you are otherwise entitled.

Reimbursements for transportation: Transportation costs incurred in order to participate in the study will be reimbursed at the rate of ₦100 per km.

For peer review only

Confidentiality: Efforts will be made to keep personal information confidential. Absolute confidentiality cannot be guaranteed. Personal information may be disclosed if required by law.

Organizations that may inspect and/or copy the research records for quality assurance and data analysis include groups such as the Research Ethics Committee. If results are published, may lead to individual / cohort identification.

Contact details of researcher – for further information / reporting of study related adverse events.

Dr Oluwaseun Akinyemi
Department of Health Policy and Management
College of Medicine
University of Ibadan
Ibadan, Nigeria
+234 803 502 0136
seunakinyemi@hotmail.com

Contact details of UI/UCH Ethics Committee chair (Nigeria) – for more information.

Prof. Catherine O. Falade
Chairperson, University of Ibadan/University College Hospital Ethics Committee
Institute for Advanced Medical Research and Training (IAMRAT)
College of Medicine, University of Ibadan
Ibadan, Nigeria
+234 803 326 4593
cfalade@comui.edu.ng

Contact details of REC administrator and chair (South Africa) – for more information.

Ms Zanele Ndlovu
Research Administrator
Human Research Ethics Committee (Medical)
University of the Witwatersrand
Johannesburg, South Africa
+27 11 717 1252
zanele.ndlovu@wits.ac.za

For peer review only

Participant Information Sheet for NGO programme managers

Study title: Scale up of community-based injectable contraceptives in Gombe State, Nigeria

Introduction:

Good day! I am Oluwaseun Akinyemi, a PhD candidate of the School of Public Health, University of the Witwatersrand, Johannesburg. I am doing research on the scale up of community-based injectable contraceptives in Gombe State, Nigeria. Research is just the process to learn the answer to a question. In this study we want to learn what the factors are that may influence the scale up of community-based distribution of injectable contraceptives in Nigeria, in order to understand possible facilitators and barriers.

Invitation to participate: I am therefore asking / inviting you to take part in this research study because you are a programme manager for an NGO involved with this programme.

What is involved in the study – I am asking different people such as policy makers, program managers, health workers, community leaders, women who are the end-users of injectable contraceptives, and broader members of the community to participate in this study. About 100 people will participate in the study and all will be from Nigeria. If you agree to participate it means you will take part in a key informant interview. Each interview will last for about 60 minutes. In the interviews I will ask questions about your knowledge and your role in the implementation or use of injectable contraceptives as well as what you think might be the challenges and things that work well in implementing this program. There are no right and wrong answers; I am interested in understanding your opinions. Please note that I intend to audio-tape this interview.

Risks and Benefits of being involved in the study – There are no invasive procedures involved in this study, thus no physical harm is envisaged. However, you don't have to answer any question you are not comfortable with.

There are no direct benefits for participating in this study.

The participant will be given pertinent information on the study while involved in the project and after the results are available.

Participation is voluntary: Refusal to participate will involve no penalty or loss of benefits to which you are otherwise entitled. Also, you may discontinue participation at any time without penalty or loss of benefits to which you are otherwise entitled.

Reimbursements for transportation: Transportation costs incurred in order to participate in the study will be reimbursed at the rate of ₦100 per km.

Confidentiality: Efforts will be made to keep personal information confidential. Absolute confidentiality cannot be guaranteed. Personal information may be disclosed if required by law.

For peer review only

Organizations that may inspect and/or copy the research records for quality assurance and data analysis include groups such as the Research Ethics Committee. If results are published, may lead to individual / cohort identification.

Contact details of researcher – for further information / reporting of study related adverse events.

Dr Oluwaseun Akinyemi
Department of Health Policy and Management
College of Medicine
University of Ibadan
Ibadan, Nigeria
+234 803 502 0136
seunakinyemi@hotmail.com

Contact details of UI/UCH Ethics Committee chair (Nigeria) – for more information.

Prof. Catherine O. Falade
Chairperson, University of Ibadan/University College Hospital Ethics Committee
Institute for Advanced Medical Research and Training (IAMRAT)
College of Medicine, University of Ibadan
Ibadan, Nigeria
+234 803 326 4593
cfalade@comui.edu.ng

Contact details of REC administrator and chair (South Africa) – for more information.

Ms Zanele Ndlovu
Research Administrator
Human Research Ethics Committee (Medical)
University of the Witwatersrand
Johannesburg, South Africa
+27 11 717 1252
zanele.ndlovu@wits.ac.za

For peer review only

Participant Information Sheet for health workers

Study title: Scale up of community-based injectable contraceptives in Gombe State, Nigeria

Introduction:

Good day! I am Oluwaseun Akinyemi, a PhD candidate of the School of Public Health, University of the Witwatersrand, Johannesburg. I am doing research on the scale up of community-based injectable contraceptives in Gombe State, Nigeria. Research is just the process to learn the answer to a question. In this study we want to learn what the factors are that may influence the scale up of community-based distribution of injectable contraceptives in Nigeria, in order to understand possible facilitators and barriers.

Invitation to participate: I am therefore asking / inviting you to take part in this research study because you are a health worker who is involved with this programme.

What is involved in the study – I am asking different people such as policy makers, program managers, health workers, community leaders, women who are the end-users of injectable contraceptives, and broader members of the community to participate in this study. About 100 people will participate in the study and all will be from Nigeria. If you agree to participate it means you will take part in a key informant interview. Each interview will last for about 60 minutes. In the interviews I will ask questions about your knowledge and your role in the implementation or use of injectable contraceptives as well as what you think might be the challenges and things that work well in implementing this program. There are no right and wrong answers; I am interested in understanding your opinions. Please note that I intend to audio-tape this interview.

Risks and Benefits of being involved in the study – There are no invasive procedures involved in this study, thus no physical harm is envisaged. However, you don't have to answer any question you are not comfortable with.

There are no direct benefits for participating in this study.

The participant will be given pertinent information on the study while involved in the project and after the results are available.

Participation is voluntary: Refusal to participate will involve no penalty or loss of benefits to which you are otherwise entitled. Also, you may discontinue participation at any time without penalty or loss of benefits to which you are otherwise entitled.

Reimbursements for transportation: Transportation costs incurred in order to participate in the study will be reimbursed at the rate of ₦100 per km.

Confidentiality: Efforts will be made to keep personal information confidential. Absolute confidentiality cannot be guaranteed. Personal information may be disclosed if required by law.

For peer review only

Organizations that may inspect and/or copy the research records for quality assurance and data analysis include groups such as the Research Ethics Committee. If results are published, may lead to individual / cohort identification.

Contact details of researcher – for further information / reporting of study related adverse events.

Dr Oluwaseun Akinyemi
Department of Health Policy and Management
College of Medicine
University of Ibadan
Ibadan, Nigeria
+234 803 502 0136
seunakinyemi@hotmail.com

Contact details of UI/UCH Ethics Committee chair (Nigeria) – for more information.

Prof. Catherine O. Falade
Chairperson, University of Ibadan/University College Hospital Ethics Committee
Institute for Advanced Medical Research and Training (IAMRAT)
College of Medicine, University of Ibadan
Ibadan, Nigeria
+234 803 326 4593
cfalade@comui.edu.ng

Contact details of REC administrator and chair (South Africa) – for more information.

Ms Zanele Ndlovu
Research Administrator
Human Research Ethics Committee (Medical)
University of the Witwatersrand
Johannesburg, South Africa
+27 11 717 1252
zanele.ndlovu@wits.ac.za

For peer review only

Participant Information Sheet for community leaders

Study title: Scale up of community-based injectable contraceptives in Gombe State, Nigeria

Introduction:

Good day! I am Oluwaseun Akinyemi, a PhD candidate of the School of Public Health, University of the Witwatersrand, Johannesburg. I am doing research on the scale up of community-based injectable contraceptives in Gombe State, Nigeria. Research is just the process to learn the answer to a question. In this study we want to learn what the factors are that may influence the scale up of community-based distribution of injectable contraceptives in Nigeria, in order to understand possible facilitators and barriers.

Invitation to participate: I am therefore asking / inviting you to take part in this research study because you are a community leader.

What is involved in the study – I am asking different people such as policy makers, program managers, health workers, community leaders, women who are the end-users of injectable contraceptives, and broader members of the community to participate in this study. About 100 people will participate in the study and all will be from Nigeria. If you agree to participate it means you will take part in a key informant interview. Each interview will last for about 60 minutes. In the interviews I will ask questions about your knowledge and your role in the implementation or use of injectable contraceptives as well as what you think might be the challenges and things that work well in implementing this program. There are no right and wrong answers; I am interested in understanding your opinions. Please note that I intend to audio-tape this interview.

Risks and Benefits of being involved in the study – There are no invasive procedures involved in this study, thus no physical harm is envisaged. However, you don't have to answer any question you are not comfortable with.

There are no direct benefits for participating in this study.

The participant will be given pertinent information on the study while involved in the project and after the results are available.

Participation is voluntary: Refusal to participate will involve no penalty or loss of benefits to which you are otherwise entitled. Also, you may discontinue participation at any time without penalty or loss of benefits to which you are otherwise entitled.

Reimbursements for transportation: Transportation costs incurred in order to participate in the study will be reimbursed at the rate of ₦100 per km.

Confidentiality: Efforts will be made to keep personal information confidential. Absolute confidentiality cannot be guaranteed. Personal information may be disclosed if required by law.

Organizations that may inspect and/or copy the research records for quality assurance and data analysis include groups such as the Research Ethics Committee. If results are published, may lead to individual / cohort identification.

Contact details of researcher – for further information / reporting of study related adverse events.

Dr Oluwaseun Akinyemi
Department of Health Policy and Management
College of Medicine
University of Ibadan
Ibadan, Nigeria
+234 803 502 0136
seunakinyemi@hotmail.com

Contact details of UI/UCH Ethics Committee chair (Nigeria) – for more information.

Prof. Catherine O. Falade
Chairperson, University of Ibadan/University College Hospital Ethics Committee
Institute for Advanced Medical Research and Training (IAMRAT)
College of Medicine, University of Ibadan
Ibadan, Nigeria
+234 803 326 4593
cfalade@comui.edu.ng

Contact details of REC administrator and chair (South Africa) – for more information.

Ms Zanele Ndlovu
Research Administrator
Human Research Ethics Committee (Medical)
University of the Witwatersrand
Johannesburg, South Africa
+27 11 717 1252
zanele.ndlovu@wits.ac.za

For peer review only

Participant Information Sheet for religious leaders

Study title: Scale up of community-based injectable contraceptives in Gombe State, Nigeria

Introduction:

Good day! I am Oluwaseun Akinyemi, a PhD candidate of the School of Public Health, University of the Witwatersrand, Johannesburg. I am doing research on the scale up of community-based injectable contraceptives in Gombe State, Nigeria. Research is just the process to learn the answer to a question. In this study we want to learn what the factors are that may influence the scale up of community-based distribution of injectable contraceptives in Nigeria, in order to understand possible facilitators and barriers.

Invitation to participate: I am therefore asking / inviting you to take part in this research study because you are a religious leader in this community.

What is involved in the study – I am asking different people such as policy makers, program managers, health workers, community leaders, women who are the end-users of injectable contraceptives, and broader members of the community to participate in this study. About 100 people will participate in the study and all will be from Nigeria. If you agree to participate it means you will take part in a key informant interview. Each interview will last for about 60 minutes. In the interviews I will ask questions about your knowledge and your role in the implementation or use of injectable contraceptives as well as what you think might be the challenges and things that work well in implementing this program. There are no right and wrong answers; I am interested in understanding your opinions. Please note that I intend to audio-tape this interview.

Risks and Benefits of being involved in the study – There are no invasive procedures involved in this study, thus no physical harm is envisaged. However, you don't have to answer any question you are not comfortable with.

There are no direct benefits for participating in this study.

The participant will be given pertinent information on the study while involved in the project and after the results are available.

Participation is voluntary: Refusal to participate will involve no penalty or loss of benefits to which you are otherwise entitled. Also, you may discontinue participation at any time without penalty or loss of benefits to which you are otherwise entitled.

Reimbursements for transportation: Transportation costs incurred in order to participate in the study will be reimbursed at the rate of ₦100 per km.

Confidentiality: Efforts will be made to keep personal information confidential. Absolute confidentiality cannot be guaranteed. Personal information may be disclosed if required by law.

For peer review only

Organizations that may inspect and/or copy the research records for quality assurance and data analysis include groups such as the Research Ethics Committee. If results are published, may lead to individual / cohort identification.

Contact details of researcher – for further information / reporting of study related adverse events.

Dr Oluwaseun Akinyemi
Department of Health Policy and Management
College of Medicine
University of Ibadan
Ibadan, Nigeria
+234 803 502 0136
seunakinyemi@hotmail.com

Contact details of UI/UCH Ethics Committee chair (Nigeria) – for more information.

Prof. Catherine O. Falade
Chairperson, University of Ibadan/University College Hospital Ethics Committee
Institute for Advanced Medical Research and Training (IAMRAT)
College of Medicine, University of Ibadan
Ibadan, Nigeria
+234 803 326 4593
cfalade@comui.edu.ng

Contact details of REC administrator and chair (South Africa) – for more information.

Ms Zanele Ndlovu
Research Administrator
Human Research Ethics Committee (Medical)
University of the Witwatersrand
Johannesburg, South Africa
+27 11 717 1252
zanele.ndlovu@wits.ac.za

For peer review only

Participant Information Sheet for women who are current users of injectable contraceptives

Study title: Scale up of community-based injectable contraceptives in Gombe State, Nigeria

Introduction:

Good day! I am Oluwaseun Akinyemi, a PhD candidate of the School of Public Health, University of the Witwatersrand, Johannesburg. I am doing research on the scale up of community-based injectable contraceptives in Gombe State, Nigeria. Research is just the process to learn the answer to a question. In this study we want to learn what the factors are that may influence the scale up of community-based distribution of injectable contraceptives in Nigeria, in order to understand possible facilitators and barriers.

Invitation to participate: I am therefore asking / inviting you to take part in this research study because you use an injectable contraceptive.

What is involved in the study – I am asking different people such as policy makers, program managers, health workers, community leaders, women who are the end-users of injectable contraceptives, and broader members of the community to participate in this study. About 100 people will participate in the study and all will be from Nigeria. If you agree to participate it means you will take part in a key informant interview. Each interview will last for about 60 minutes. In the interviews I will ask questions about your knowledge and your role in the implementation or use of injectable contraceptives as well as what you think might be the challenges and things that work well in implementing this program. There are no right and wrong answers; I am interested in understanding your opinions. Please note that I intend to audio-tape this interview.

Risks and Benefits of being involved in the study – There are no invasive procedures involved in this study, thus no physical harm is envisaged. However, you don't have to answer any question you are not comfortable with.

There are no direct benefits for participating in this study.

The participant will be given pertinent information on the study while involved in the project and after the results are available.

Participation is voluntary: Refusal to participate will involve no penalty or loss of benefits to which you are otherwise entitled. Also, you may discontinue participation at any time without penalty or loss of benefits to which you are otherwise entitled.

Reimbursements for transportation: Transportation costs incurred in order to participate in the study will be reimbursed at the rate of ₦100 per km.

For peer review only

Confidentiality: Efforts will be made to keep personal information confidential. However, confidentiality is not guaranteed in a focus group discussion. Personal information may be disclosed if required by law.

Organizations that may inspect and/or copy the research records for quality assurance and data analysis include groups such as the Research Ethics Committee. If results are published, may lead to individual / cohort identification.

Contact details of researcher – for further information / reporting of study related adverse events.

Dr Oluwaseun Akinyemi
Department of Health Policy and Management
College of Medicine
University of Ibadan
Ibadan, Nigeria
+234 803 502 0136
seunakinyemi@hotmail.com

Contact details of UI/UCH Ethics Committee chair (Nigeria) – for more information.

Prof. Catherine O. Falade
Chairperson, University of Ibadan/University College Hospital Ethics Committee
Institute for Advanced Medical Research and Training (IAMRAT)
College of Medicine, University of Ibadan
Ibadan, Nigeria
+234 803 326 4593
cfalade@comui.edu.ng

Contact details of REC administrator and chair (South Africa– for more information.

Ms Zanele Ndlovu
Research Administrator
Human Research Ethics Committee (Medical)
University of the Witwatersrand
Johannesburg, South Africa
+27 11 717 1252
zanele.ndlovu@wits.ac.za

For peer review only

Participant Information Sheet for women who do not use injectable contraceptives

Study title: Scale up of community-based injectable contraceptives in Gombe State, Nigeria

Introduction:

Good day! I am Oluwaseun Akinyemi, a PhD candidate of the School of Public Health, University of the Witwatersrand, Johannesburg. I am doing research on the scale up of community-based injectable contraceptives in Gombe State, Nigeria. Research is just the process to learn the answer to a question. In this study we want to learn what the factors are that may influence the scale up of community-based distribution of injectable contraceptives in Nigeria, in order to understand possible facilitators and barriers.

Invitation to participate: I am therefore asking / inviting you to take part in this research study because you do not use an injectable contraceptive.

What is involved in the study – I am asking different people such as policy makers, program managers, health workers, community leaders, women who are the end-users of injectable contraceptives, and broader members of the community to participate in this study. About 100 people will participate in the study and all will be from Nigeria. If you agree to participate it means you will take part in a key informant interview. Each interview will last for about 60 minutes. In the interviews I will ask questions about your knowledge and your role in the implementation or use of injectable contraceptives as well as what you think might be the challenges and things that work well in implementing this program. There are no right and wrong answers; I am interested in understanding your opinions. Please note that I intend to audio-tape this interview.

Risks and Benefits of being involved in the study – There are no invasive procedures involved in this study, thus no physical harm is envisaged. However, you don't have to answer any question you are not comfortable with.

There are no direct benefits for participating in this study.

The participant will be given pertinent information on the study while involved in the project and after the results are available.

Participation is voluntary: Refusal to participate will involve no penalty or loss of benefits to which you are otherwise entitled. Also, you may discontinue participation at any time without penalty or loss of benefits to which you are otherwise entitled.

Reimbursements for transportation: Transportation costs incurred in order to participate in the study will be reimbursed at the rate of ₦100 per km.

Confidentiality: Efforts will be made to keep personal information confidential. However, confidentiality is not guaranteed in a focus group discussion. Personal information may be disclosed if required by law.

For peer review only

Organizations that may inspect and/or copy the research records for quality assurance and data analysis include groups such as the Research Ethics Committee. If results are published, may lead to individual / cohort identification.

Contact details of researcher – for further information / reporting of study related adverse events.

Dr Oluwaseun Akinyemi
Department of Health Policy and Management
College of Medicine
University of Ibadan
Ibadan, Nigeria
+234 803 502 0136
seunakinyemi@hotmail.com

Contact details of UI/UCH Ethics Committee chair (Nigeria) – for more information.

Prof. Catherine O. Falade
Chairperson, University of Ibadan/University College Hospital Ethics Committee
Institute for Advanced Medical Research and Training (IAMRAT)
College of Medicine, University of Ibadan
Ibadan, Nigeria
+234 803 326 4593
cfalade@comui.edu.ng

Contact details of REC administrator and chair (South Africa– for more information.

Ms Zanele Ndlovu
Research Administrator
Human Research Ethics Committee (Medical)
University of the Witwatersrand
Johannesburg, South Africa
+27 11 717 1252
zanele.ndlovu@wits.ac.za

For peer review only

Participant Information Sheet for married men

Study title: Scale up of community-based injectable contraceptives in Gombe State, Nigeria

Introduction:

Good day! I am Oluwaseun Akinyemi, a PhD candidate of the School of Public Health, University of the Witwatersrand, Johannesburg. I am doing research on the scale up of community-based injectable contraceptives in Gombe State, Nigeria. Research is just the process to learn the answer to a question. In this study we want to learn what the factors are that may influence the scale up of community-based distribution of injectable contraceptives in Nigeria, in order to understand possible facilitators and barriers.

Invitation to participate: I am therefore asking / inviting you to take part in this research study because you are a married man.

What is involved in the study – I am asking different people such as policy makers, program managers, health workers, community leaders, women who are the end-users of injectable contraceptives, and broader members of the community to participate in this study. About 100 people will participate in the study and all will be from Nigeria. If you agree to participate it means you will take part in a key informant interview. Each interview will last for about 60 minutes. In the interviews I will ask questions about your knowledge and your role in the implementation or use of injectable contraceptives as well as what you think might be the challenges and things that work well in implementing this program. There are no right and wrong answers; I am interested in understanding your opinions. Please note that I intend to audio-tape this interview.

Risks and Benefits of being involved in the study – There are no invasive procedures involved in this study, thus no physical harm is envisaged. However, you don't have to answer any question you are not comfortable with.

There are no direct benefits for participating in this study.

The participant will be given pertinent information on the study while involved in the project and after the results are available.

Participation is voluntary: Refusal to participate will involve no penalty or loss of benefits to which you are otherwise entitled. Also, you may discontinue participation at any time without penalty or loss of benefits to which you are otherwise entitled.

Reimbursements for transportation: Transportation costs incurred in order to participate in the study will be reimbursed at the rate of ₦100 per km.

Confidentiality: Efforts will be made to keep personal information confidential. However, confidentiality is not guaranteed in a focus group discussion. Personal information may be disclosed if required by law.

For peer review only

Organizations that may inspect and/or copy the research records for quality assurance and data analysis include groups such as the Research Ethics Committee. If results are published, may lead to individual / cohort identification.

Contact details of researcher – for further information / reporting of study related adverse events.

Dr Oluwaseun Akinyemi
Department of Health Policy and Management
College of Medicine
University of Ibadan
Ibadan, Nigeria
+234 803 502 0136
seunakinyemi@hotmail.com

Contact details of UI/UCH Ethics Committee chair (Nigeria) – for more information.

Prof. Catherine O. Falade
Chairperson, University of Ibadan/University College Hospital Ethics Committee
Institute for Advanced Medical Research and Training (IAMRAT)
College of Medicine, University of Ibadan
Ibadan, Nigeria
+234 803 326 4593
cfalade@comui.edu.ng

Contact details of REC administrator and chair (South Africa) – for more information.

Ms Zanele Ndlovu
Research Administrator
Human Research Ethics Committee (Medical)
University of the Witwatersrand
Johannesburg, South Africa
+27 11 717 1252
zanele.ndlovu@wits.ac.za

For peer review only

Participant Information Sheet for unmarried/single men

Study title: Scale up of community-based injectable contraceptives in Gombe State, Nigeria

Introduction:

Good day! I am Oluwaseun Akinyemi, a PhD candidate of the School of Public Health, University of the Witwatersrand, Johannesburg. I am doing research on the scale up of community-based injectable contraceptives in Gombe State, Nigeria. Research is just the process to learn the answer to a question. In this study we want to learn what the factors are that may influence the scale up of community-based distribution of injectable contraceptives in Nigeria, in order to understand possible facilitators and barriers.

Invitation to participate: I am therefore asking / inviting you to take part in this research study because you are an unmarried/ single man.

What is involved in the study – I am asking different people such as policy makers, program managers, health workers, community leaders, women who are the end-users of injectable contraceptives, and broader members of the community to participate in this study. About 100 people will participate in the study and all will be from Nigeria. If you agree to participate it means you will take part in a key informant interview. Each interview will last for about 60 minutes. In the interviews I will ask questions about your knowledge and your role in the implementation or use of injectable contraceptives as well as what you think might be the challenges and things that work well in implementing this program. There are no right and wrong answers; I am interested in understanding your opinions. Please note that I intend to audio-tape this interview.

Risks and Benefits of being involved in the study – There are no invasive procedures involved in this study, thus no physical harm is envisaged. However, you don't have to answer any question you are not comfortable with.

There are no direct benefits for participating in this study.

The participant will be given pertinent information on the study while involved in the project and after the results are available.

Participation is voluntary: Refusal to participate will involve no penalty or loss of benefits to which you are otherwise entitled. Also, you may discontinue participation at any time without penalty or loss of benefits to which you are otherwise entitled.

Reimbursements for transportation: Transportation costs incurred in order to participate in the study will be reimbursed at the rate of ₦100 per km.

Confidentiality: Efforts will be made to keep personal information confidential. However, confidentiality is not guaranteed in a focus group discussion. Personal information may be disclosed if required by law.

For peer review only

Organizations that may inspect and/or copy the research records for quality assurance and data analysis include groups such as the Research Ethics Committee. If results are published, may lead to individual / cohort identification.

Contact details of researcher – for further information / reporting of study related adverse events.

Dr Oluwaseun Akinyemi
Department of Health Policy and Management
College of Medicine
University of Ibadan
Ibadan, Nigeria
+234 803 502 0136
seunakinyemi@hotmail.com

Contact details of UI/UCH Ethics Committee chair (Nigeria) – for more information.

Prof. Catherine O. Falade
Chairperson, University of Ibadan/University College Hospital Ethics Committee
Institute for Advanced Medical Research and Training (IAMRAT)
College of Medicine, University of Ibadan
Ibadan, Nigeria
+234 803 326 4593
cfalade@comui.edu.ng

Contact details of REC administrator and chair (South Africa) – for more information.

Ms Zanele Ndlovu
Research Administrator
Human Research Ethics Committee (Medical)
University of the Witwatersrand
Johannesburg, South Africa
+27 11 717 1252
zanele.ndlovu@wits.ac.za

For peer review only

Informed Consent Agreement for Senior MoH officials

Study title: Scale up of community-based injectable contraceptives in Gombe State, Nigeria

SERIAL NUMBER ___________________

Background and purpose: One of the health innovations that have been piloted with significant success around the world, including Nigeria, is the community-based distribution of injectable contraceptives. This study will contribute to providing evidence towards aiding our understanding of the barriers and facilitators to the introduction, dissemination, diffusion and integration of the community-based distribution of injectable contraceptives intervention in Nigeria.

Procedures: You have been selected as one of this study's participants for a key informant interview. Each interview will last for about 60 minutes. In the interviews I will ask questions about your knowledge and your role in the implementation or use of injectable contraceptives as well as what you think might be the challenges and things that work well in implementing this program.

Potential Risks: There are no health risks associated with the interviews and discussions.

Potential benefits: The findings of this study will help enhance the scale up of injectable contraceptives in Nigeria thereby helping to promote maternal and child health as well as healthier families.

Reimbursements for "out of pocket" expenses: Transportation costs incurred in order to participate in the study will be reimbursed at the rate of ₦100 per km.

Withdrawal from participation: If you agree to take part you are free to end your participation in this study at any time you want without having to give reasons.

Additional Information: In case you have any question or concern about this study please feel free to contact Dr Oluwaseun Akinyemi, Department of Health Policy and Management, College of Medicine, University of Ibadan Ibadan, Nigeria (Tel. No.: +234 803 502 0136; Email: seunakinyemi@hotmail.com)

Informed consent record: Please read this statement carefully before you sign and if you agree on participating, please fill free to sign this form below to affirm your consent.

I, _________________________________ clearly understand the aims of the project titled **"Scale up of community-based injectable contraceptives in Gombe State, Nigeria"** which has been explained to me by the researcher. I have been given the chance to ask questions and I am satisfied with the answers to all of my questions and I agree to participate in the study. I understand that I may revoke my consent and leave the study at any stage, if I wish so, with no negative consequences.

For peer review only

Participant's name: ____

Participant's signature or thumbprint: ____________________ Date: ______________

Witness Name (As appropriate): ____

Witness signature (As appropriate): ___________________ Date: _________________

Study team member's statement: I, the undersigned, have explained to the participant in a language that s/he understands; the procedures to be followed in the study, the risks and benefits involved, and the obligations of the study team.

_____________________________ ___________________________ _______________

Name Signature Date

For peer review only

Informed Consent Agreement for program managers

Study title: Scale up of community-based injectable contraceptives in Gombe State, Nigeria

SERIAL NUMBER ___________________

Background and purpose: One of the health innovations that have been piloted with significant success around the world, including Nigeria, is the community-based distribution of injectable contraceptives. This study will contribute to providing evidence towards aiding our understanding of the barriers and facilitators to the introduction, dissemination, diffusion and integration of the community-based distribution of injectable contraceptives intervention in Nigeria.

Procedures: You have been selected as one of this study's participants for a key informant interview. Each interview will last for about 60 minutes. In the interviews I will ask questions about your knowledge and your role in the implementation or use of injectable contraceptives as well as what you think might be the challenges and things that work well in implementing this program.

Potential Risks: There are no health risks associated with the interviews and discussions.

Potential benefits: The findings of this study will help enhance the scale up of injectable contraceptives in Nigeria thereby helping to promote maternal and child health as well as healthier families.

Reimbursements for "out of pocket" expenses: Transportation costs incurred in order to participate in the study will be reimbursed at the rate of ₦100 per km.

Withdrawal from participation: If you agree to take part you are free to end your participation in this study at any time you want without having to give reasons.

Additional Information: In case you have any question or concern about this study please feel free to contact Dr Oluwaseun Akinyemi, Department of Health Policy and Management, College of Medicine, University of Ibadan Ibadan, Nigeria (Tel. No.: +234 803 502 0136; Email: seunakinyemi@hotmail.com)

Informed consent record: Please read this statement carefully before you sign and if you agree on participating, please fill free to sign this form below to affirm your consent.

I, _________________________________ clearly understand the aims of the project titled **"Scale up of community-based injectable contraceptives in Gombe State, Nigeria"** which has been explained to me by the researcher. I have been given the chance to ask questions and I am satisfied with the answers to all of my questions and I agree to participate in the study. I understand that I may revoke my consent and leave the study at any stage, if I wish so, with no negative consequences.

For peer review only

Participant's name: ____

Participant's signature or thumbprint: _____________________ Date: _______________

Witness Name (As appropriate): ____

Witness signature (As appropriate): ____________________ Date: __________________

Study team member's statement: I, the undersigned, have explained to the participant in a language that s/he understands; the procedures to be followed in the study, the risks and benefits involved, and the obligations of the study team.

____________________________ ____________________________ ________________

Name Signature Date

For peer review only

Informed Consent Agreement for health workers

Study title: Scale up of community-based injectable contraceptives in Gombe State, Nigeria

SERIAL NUMBER ___________________

Background and purpose: One of the health innovations that have been piloted with significant success around the world, including Nigeria, is the community-based distribution of injectable contraceptives. This study will contribute to providing evidence towards aiding our understanding of the barriers and facilitators to the introduction, dissemination, diffusion and integration of the community-based distribution of injectable contraceptives intervention in Nigeria.

Procedures: You have been selected as one of this study's participants for an in-depth interview. Each interview will last for about 60 minutes. In the interviews and discussions I will ask questions about your knowledge and your role in the implementation or use of injectable contraceptives as well as what you think might be the challenges and things that work well in implementing this program.

Potential Risks: There are no health risks associated with the interviews and discussions.

Potential benefits: The findings of this study will help enhance the scale up of injectable contraceptives in Nigeria thereby helping to promote maternal and child health as well as healthier families.

Reimbursements for "out of pocket" expenses: Transportation costs incurred in order to participate in the study will be reimbursed at the rate of ₦100 per km.

Withdrawal from participation: If you agree to take part you are free to end your participation in this study at any time you want without having to give reasons.

Additional Information: In case you have any question or concern about this study please feel free to contact Dr Oluwaseun Akinyemi, Department of Health Policy and Management, College of Medicine, University of Ibadan Ibadan, Nigeria (Tel. No.: +234 803 502 0136; Email: seunakinyemi@hotmail.com)

Informed consent record: Please read this statement carefully before you sign and if you agree on participating, please fill free to sign this form below to affirm your consent.

I, _________________________________ clearly understand the aims of the project titled **"Scale up of community-based injectable contraceptives in Gombe State, Nigeria"** which has been explained to me by the researcher. I have been given the chance to ask questions and I am satisfied with the answers to all of my questions and I agree to participate in the study. I understand that I may revoke my consent and leave the study at any stage, if I wish so, with no negative consequences.

For peer review only

Participant's name: ____

Participant's signature or thumbprint: ______________________ Date: ________________

Witness Name (As appropriate): ____

Witness signature (As appropriate): _____________________ Date: __________________

Study team member's statement: I, the undersigned, have explained to the participant in a language that s/he understands; the procedures to be followed in the study, the risks and benefits involved, and the obligations of the study team.

______________________________ ______________________________ ________________

Name Signature Date

For peer review only

Informed Consent Agreement for community leaders

Study title: Scale up of community-based injectable contraceptives in Gombe State, Nigeria

SERIAL NUMBER ___________________

Background and purpose: One of the health innovations that have been piloted with significant success around the world, including Nigeria, is the community-based distribution of injectable contraceptives. This study will contribute to providing evidence towards aiding our understanding of the barriers and facilitators to the introduction, dissemination, diffusion and integration of the community-based distribution of injectable contraceptives intervention in Nigeria.

Procedures: You have been selected as one of this study's participants for an in-depth interview. Each interview will last for about 60 minutes. In the interviews and discussions I will ask questions about your knowledge and your role in the implementation or use of injectable contraceptives as well as what you think might be the challenges and things that work well in implementing this program.

Potential Risks: There are no health risks associated with the interviews and discussions.

Potential benefits: The findings of this study will help enhance the scale up of injectable contraceptives in Nigeria thereby helping to promote maternal and child health as well as healthier families.

Reimbursements for "out of pocket" expenses: Transportation costs incurred in order to participate in the study will be reimbursed at the rate of ₦100 per km.

Withdrawal from participation: If you agree to take part you are free to end your participation in this study at any time you want without having to give reasons.

Additional Information: In case you have any question or concern about this study please feel free to contact Dr Oluwaseun Akinyemi, Department of Health Policy and Management, College of Medicine, University of Ibadan Ibadan, Nigeria (Tel. No.: +234 803 502 0136; Email: seunakinyemi@hotmail.com)

Informed consent record: Please read this statement carefully before you sign and if you agree on participating, please fill free to sign this form below to affirm your consent.

I, _________________________________ clearly understand the aims of the project titled **"Scale up of community-based injectable contraceptives in Gombe State, Nigeria"** which has been explained to me by the researcher. I have been given the chance to ask questions and I am satisfied with the answers to all of my questions and I agree to participate in the study. I understand that I may revoke my consent and leave the study at any stage, if I wish so, with no negative consequences.

For peer review only

Participant’s name: ____

Participant’s signature or thumbprint: ____________________ Date: ______________

Witness Name (As appropriate): ____

Witness signature (As appropriate): ___________________ Date: ________________

Study team member’s statement: I, the undersigned, have explained to the participant in a language that s/he understands; the procedures to be followed in the study, the risks and benefits involved, and the obligations of the study team.

___________________________ ___________________________ _______________

Name Signature Date

For peer review only

Informed Consent Agreement for religious leaders

Study title: Scale up of community-based injectable contraceptives in Gombe State, Nigeria

SERIAL NUMBER ___________________

Background and purpose: One of the health innovations that have been piloted with significant success around the world, including Nigeria, is the community-based distribution of injectable contraceptives. This study will contribute to providing evidence towards aiding our understanding of the barriers and facilitators to the introduction, dissemination, diffusion and integration of the community-based distribution of injectable contraceptives intervention in Nigeria.

Procedures: You have been selected as one of this study's participants for an in-depth interview. Each interview will last for about 60 minutes. In the interviews and discussions I will ask questions about your knowledge and your role in the implementation or use of injectable contraceptives as well as what you think might be the challenges and things that work well in implementing this program.

Potential Risks: There are no health risks associated with the interviews and discussions.

Potential benefits: The findings of this study will help enhance the scale up of injectable contraceptives in Nigeria thereby helping to promote maternal and child health as well as healthier families.

Reimbursements for "out of pocket" expenses: Transportation costs incurred in order to participate in the study will be reimbursed at the rate of ₦100 per km.

Withdrawal from participation: If you agree to take part you are free to end your participation in this study at any time you want without having to give reasons.

Additional Information: In case you have any question or concern about this study please feel free to contact Dr Oluwaseun Akinyemi, Department of Health Policy and Management, College of Medicine, University of Ibadan Ibadan, Nigeria (Tel. No.: +234 803 502 0136; Email: seunakinyemi@hotmail.com)

Informed consent record: Please read this statement carefully before you sign and if you agree on participating, please fill free to sign this form below to affirm your consent.

I, ________________________________ clearly understand the aims of the project titled **"Scale up of community-based injectable contraceptives in Gombe State, Nigeria"** which has been explained to me by the researcher. I have been given the chance to ask questions and I am satisfied with the answers to all of my questions and I agree to participate in the study. I understand that I may revoke my consent and leave the study at any stage, if I wish so, with no negative consequences.

Participant's name: ____

Participant's signature or thumbprint: ____________________ Date: ______________

Witness Name (As appropriate): ____

Witness signature (As appropriate): ___________________ Date: _________________

Study team member's statement: I, the undersigned, have explained to the participant in a language that s/he understands; the procedures to be followed in the study, the risks and benefits involved, and the obligations of the study team.

___________________________ ___________________________ _______________

Name Signature Date

For peer review only

Informed Consent Agreement for women who are current users of injectable contraceptives

Study title: Scale up of community-based injectable contraceptives in Gombe State, Nigeria

SERIAL NUMBER ___________________

Background and purpose: One of the health innovations that have been piloted with significant success around the world, including Nigeria, is the community-based distribution of injectable contraceptives. This study will contribute to providing evidence towards aiding our understanding of the barriers and facilitators to the introduction, dissemination, diffusion and integration of the community-based distribution of injectable contraceptives intervention in Nigeria.

Procedures: You have been selected as one of this study's participants for a focus group discussion or a questionnaire interview. Each interview or focus group discussion will last for about 60 minutes. In the interviews and discussions I will ask questions about your knowledge and your role in the implementation or use of injectable contraceptives as well as what you think might be the challenges and things that work well in implementing this program.

Potential Risks: There are no health risks associated with the interviews and discussions.

Potential benefits: The findings of this study will help enhance the scale up of injectable contraceptives in Nigeria thereby helping to promote maternal and child health as well as healthier families.

Reimbursements for "out of pocket" expenses: Transportation costs incurred in order to participate in the study will be reimbursed at the rate of ₦100 per km.

Withdrawal from participation: If you agree to take part you are free to end your participation in this study at any time you want without having to give reasons.

Additional Information: In case you have any question or concern about this study please feel free to contact Dr Oluwaseun Akinyemi, Department of Health Policy and Management, College of Medicine, University of Ibadan Ibadan, Nigeria (Tel. No.: +234 803 502 0136; Email: seunakinyemi@hotmail.com)

Informed consent record: Please read this statement carefully before you sign and if you agree on participating, please fill free to sign this form below to affirm your consent.

I, _______________________________ clearly understand the aims of the project titled **"Scale up of community-based injectable contraceptives in Gombe State, Nigeria"** which has been explained to me by the researcher. I have been given the chance to ask questions and I am satisfied with the answers to all of my questions and I agree to participate in the study. I understand

that I may revoke my consent and leave the study at any stage, if I wish so, with no negative consequences.

Participant's name: ____

Participant's signature or thumbprint: ______________________ Date: ______________

Witness Name (As appropriate): ____

Witness signature (As appropriate): ____________________ Date: _________________

Study team member's statement: I, the undersigned, have explained to the participant in a language that s/he understands; the procedures to be followed in the study, the risks and benefits involved, and the obligations of the study team.

____________________________ ____________________________ _______________

Name Signature Date

For peer review only

Informed Consent Agreement for women who do not use injectable contraceptives

Study title: Scale up of community-based injectable contraceptives in Gombe State, Nigeria

SERIAL NUMBER ____________________

Background and purpose: One of the health innovations that have been piloted with significant success around the world, including Nigeria, is the community-based distribution of injectable contraceptives. This study will contribute to providing evidence towards aiding our understanding of the barriers and facilitators to the introduction, dissemination, diffusion and integration of the community-based distribution of injectable contraceptives intervention in Nigeria.

Procedures: You have been selected as one of this study's participants for a focus group discussion. Each focus group discussion will last for about 60 minutes. In the interviews and discussions I will ask questions about your knowledge and your role in the implementation or use of injectable contraceptives as well as what you think might be the challenges and things that work well in implementing this program.

Potential Risks: There are no health risks associated with the interviews and discussions.

Potential benefits: The findings of this study will help enhance the scale up of injectable contraceptives in Nigeria thereby helping to promote maternal and child health as well as healthier families.

Reimbursements for "out of pocket" expenses: Transportation costs incurred in order to participate in the study will be reimbursed at the rate of ₦100 per km.

Withdrawal from participation: If you agree to take part you are free to end your participation in this study at any time you want without having to give reasons.

Additional Information: In case you have any question or concern about this study please feel free to contact Dr Oluwaseun Akinyemi, Department of Health Policy and Management, College of Medicine, University of Ibadan Ibadan, Nigeria (Tel. No.: +234 803 502 0136; Email: seunakinyemi@hotmail.com)

Informed consent record: Please read this statement carefully before you sign and if you agree on participating, please fill free to sign this form below to affirm your consent.

I, ________________________________ clearly understand the aims of the project titled **"Scale up of community-based injectable contraceptives in Gombe State, Nigeria"** which has been explained to me by the researcher. I have been given the chance to ask questions and I am satisfied with the answers to all of my questions and I agree to participate in the study. I understand that I may revoke my consent and leave the study at any stage, if I wish so, with no negative consequences.

For peer review only

Participant’s name: ____

Participant’s signature or thumbprint: ____________________ Date: ______________

Witness Name (As appropriate): ____

Witness signature (As appropriate): ___________________ Date: _________________

Study team member’s statement: I, the undersigned, have explained to the participant in a language that s/he understands; the procedures to be followed in the study, the risks and benefits involved, and the obligations of the study team.

___________________________ ___________________________ _______________

Name Signature Date

For peer review only

Informed Consent Agreement for married men

Study title: Scale up of community-based injectable contraceptives in Gombe State, Nigeria

SERIAL NUMBER ____________________

Background and purpose: One of the health innovations that have been piloted with significant success around the world, including Nigeria, is the community-based distribution of injectable contraceptives. This study will contribute to providing evidence towards aiding our understanding of the barriers and facilitators to the introduction, dissemination, diffusion and integration of the community-based distribution of injectable contraceptives intervention in Nigeria.

Procedures: You have been selected as one of this study's participants for a focus group discussion. Each focus group discussion will last for about 60 minutes. In the interviews and discussions I will ask questions about your knowledge and your role in the implementation or use of injectable contraceptives as well as what you think might be the challenges and things that work well in implementing this program.

Potential Risks: There are no health risks associated with the interviews and discussions.

Potential benefits: The findings of this study will help enhance the scale up of injectable contraceptives in Nigeria thereby helping to promote maternal and child health as well as healthier families.

Reimbursements for "out of pocket" expenses: Transportation costs incurred in order to participate in the study will be reimbursed at the rate of ₦100 per km.

Withdrawal from participation: If you agree to take part you are free to end your participation in this study at any time you want without having to give reasons.

Additional Information: In case you have any question or concern about this study please feel free to contact Dr Oluwaseun Akinyemi, Department of Health Policy and Management, College of Medicine, University of Ibadan Ibadan, Nigeria (Tel. No.: +234 803 502 0136; Email: seunakinyemi@hotmail.com)

Informed consent record: Please read this statement carefully before you sign and if you agree on participating, please fill free to sign this form below to affirm your consent.

I, _________________________________ clearly understand the aims of the project titled **"Scale up of community-based injectable contraceptives in Gombe State, Nigeria"** which has been explained to me by the researcher. I have been given the chance to ask questions and I am satisfied with the answers to all of my questions and I agree to participate in the study. I understand that I may revoke my consent and leave the study at any stage, if I wish so, with no negative consequences.

For peer review only

Participant's name: ____

Participant's signature or thumbprint: ______________________ Date: ________________

Witness Name (As appropriate): ____

Witness signature (As appropriate): ______________________ Date: ___________________

Study team member's statement: I, the undersigned, have explained to the participant in a language that s/he understands; the procedures to be followed in the study, the risks and benefits involved, and the obligations of the study team.

_______________________________ _______________________________ _________________

Name Signature Date

For peer review only

Informed Consent Agreement for unmarried men

Study title: Scale up of community-based injectable contraceptives in Gombe State, Nigeria

SERIAL NUMBER ___________________

Background and purpose: One of the health innovations that have been piloted with significant success around the world, including Nigeria, is the community-based distribution of injectable contraceptives. This study will contribute to providing evidence towards aiding our understanding of the barriers and facilitators to the introduction, dissemination, diffusion and integration of the community-based distribution of injectable contraceptives intervention in Nigeria.

Procedures: You have been selected as one of this study's participants for a focus group discussion. Each focus group discussion will last for about 60 minutes. In the interviews and discussions I will ask questions about your knowledge and your role in the implementation or use of injectable contraceptives as well as what you think might be the challenges and things that work well in implementing this program.

Potential Risks: There are no health risks associated with the interviews and discussions.

Potential benefits: The findings of this study will help enhance the scale up of injectable contraceptives in Nigeria thereby helping to promote maternal and child health as well as healthier families.

Reimbursements for "out of pocket" expenses: Transportation costs incurred in order to participate in the study will be reimbursed at the rate of ₦100 per km.

Withdrawal from participation: If you agree to take part you are free to end your participation in this study at any time you want without having to give reasons.

Additional Information: In case you have any question or concern about this study please feel free to contact Dr Oluwaseun Akinyemi, Department of Health Policy and Management, College of Medicine, University of Ibadan Ibadan, Nigeria (Tel. No.: +234 803 502 0136; Email: seunakinyemi@hotmail.com)

Informed consent record: Please read this statement carefully before you sign and if you agree on participating, please fill free to sign this form below to affirm your consent.

I, _________________________________ clearly understand the aims of the project titled **"Scale up of community-based injectable contraceptives in Gombe State, Nigeria"** which has been explained to me by the researcher. I have been given the chance to ask questions and I am satisfied with the answers to all of my questions and I agree to participate in the study. I understand that I may revoke my consent and leave the study at any stage, if I wish so, with no negative consequences.

For peer review only

Participant’s name: ____

Participant’s signature or thumbprint: ___________________ Date: _____________

Witness Name (As appropriate): ____

Witness signature (As appropriate): __________________ Date: ________________

Study team member’s statement: I, the undersigned, have explained to the participant in a language that s/he understands; the procedures to be followed in the study, the risks and benefits involved, and the obligations of the study team.

__________________________ __________________________ ______________

Name Signature Date

For peer review only

Informed Consent Agreement to audio record interview senior MoH officials

Study title: Scale up of community-based injectable contraceptives in Gombe State, Nigeria

SERIAL NUMBER ___________________

Background and purpose: One of the health innovations that have been piloted with significant success around the world, including Nigeria, is the community-based distribution of injectable contraceptives.

Additional Information: In case you have any question or concern about this study please feel free to contact Dr Oluwaseun Akinyemi, Department of Health Policy and Management, College of Medicine, University of Ibadan Ibadan, Nigeria (Tel. No.: +234 803 502 0136; Email: seunakinyemi@hotmail.com)

Informed consent to record: Please read this statement carefully before you sign and if you agree that the interview / discussion with you should be audio recorded, please fill free to sign this form below to affirm your consent.

I, ______________________________ clearly understand the aims of the project titled **"Scale up of community-based injectable contraceptives in Gombe State, Nigeria"** which has been explained to me by the researcher. I have been given the chance to ask questions and I am satisfied with the answers to all of my questions and I agree to participate in the study. I understand that I may revoke my consent and leave the study at any stage, if I wish so, with no negative consequences. I agree that the interview / discussion with me should be audio recorded. I understand that the recording is for research purposes only.

Participant's name: ____

Participant's signature or thumbprint: ____________________ Date: ______________

Witness Name (As appropriate): ____

Witness signature (As appropriate): ___________________ Date: _________________

Study team member's statement: I, the undersigned, have explained to the participant in a language that s/he understands; the procedures to be followed in the study (including audio recording of the interview), the risks and benefits involved, and the obligations of the study team.

___________________________ ___________________________ _______________

Name Signature Date

For peer review only

Informed Consent Agreement to audio record interview with program managers

Study title: Scale up of community-based injectable contraceptives in Gombe State, Nigeria

SERIAL NUMBER ____________________

Background and purpose: One of the health innovations that have been piloted with significant success around the world, including Nigeria, is the community-based distribution of injectable contraceptives.

Additional Information: In case you have any question or concern about this study please feel free to contact Dr Oluwaseun Akinyemi, Department of Health Policy and Management, College of Medicine, University of Ibadan Ibadan, Nigeria (Tel. No.: +234 803 502 0136; Email: seunakinyemi@hotmail.com)

Informed consent to record: Please read this statement carefully before you sign and if you agree that the interview / discussion with you should be audio recorded, please fill free to sign this form below to affirm your consent.

I, ________________________________ clearly understand the aims of the project titled **"Scale up of community-based injectable contraceptives in Gombe State, Nigeria"** which has been explained to me by the researcher. I have been given the chance to ask questions and I am satisfied with the answers to all of my questions and I agree to participate in the study. I understand that I may revoke my consent and leave the study at any stage, if I wish so, with no negative consequences. I agree that the interview / discussion with me should be audio recorded. I understand that the recording is for research purposes only.

Participant's name: ____

Participant's signature or thumbprint: ____________________ Date: _______________

Witness Name (As appropriate): ____

Witness signature (As appropriate): ____________________ Date: __________________

Study team member's statement: I, the undersigned, have explained to the participant in a language that s/he understands; the procedures to be followed in the study (including audio recording of the interview), the risks and benefits involved, and the obligations of the study team.

_____________________________ _____________________________ ________________

Name Signature Date

For peer review only

Informed Consent Agreement to audio record interview with health workers

Study title: Scale up of community-based injectable contraceptives in Gombe State, Nigeria

SERIAL NUMBER ___________________

Background and purpose: One of the health innovations that have been piloted with significant success around the world, including Nigeria, is the community-based distribution of injectable contraceptives.

Additional Information: In case you have any question or concern about this study please feel free to contact Dr Oluwaseun Akinyemi, Department of Health Policy and Management, College of Medicine, University of Ibadan Ibadan, Nigeria (Tel. No.: +234 803 502 0136; Email: seunakinyemi@hotmail.com)

Informed consent to record: Please read this statement carefully before you sign and if you agree that the interview / discussion with you should be audio recorded, please fill free to sign this form below to affirm your consent.

I, ______________________________ clearly understand the aims of the project titled **"Scale up of community-based injectable contraceptives in Gombe State, Nigeria"** which has been explained to me by the researcher. I have been given the chance to ask questions and I am satisfied with the answers to all of my questions and I agree to participate in the study. I understand that I may revoke my consent and leave the study at any stage, if I wish so, with no negative consequences. I agree that the interview / discussion with me should be audio recorded. I understand that the recording is for research purposes only.

Participant's name: ____

Participant's signature or thumbprint: ____________________ Date: ______________

Witness Name (As appropriate): ____

Witness signature (As appropriate): ___________________ Date: _________________

Study team member's statement: I, the undersigned, have explained to the participant in a language that s/he understands; the procedures to be followed in the study (including audio recording of the interview), the risks and benefits involved, and the obligations of the study team.

___________________________ ___________________________ _______________

Name Signature Date

For peer review only

Informed Consent Agreement to audio record interview with community leaders

Study title: Scale up of community-based injectable contraceptives in Gombe State, Nigeria

SERIAL NUMBER ____________________

Background and purpose: One of the health innovations that have been piloted with significant success around the world, including Nigeria, is the community-based distribution of injectable contraceptives.

Additional Information: In case you have any question or concern about this study please feel free to contact Dr Oluwaseun Akinyemi, Department of Health Policy and Management, College of Medicine, University of Ibadan Ibadan, Nigeria (Tel. No.: +234 803 502 0136; Email: seunakinyemi@hotmail.com)

Informed consent to record: Please read this statement carefully before you sign and if you agree that the interview / discussion with you should be audio recorded, please fill free to sign this form below to affirm your consent.

I, ________________________________ clearly understand the aims of the project titled **"Scale up of community-based injectable contraceptives in Gombe State, Nigeria"** which has been explained to me by the researcher. I have been given the chance to ask questions and I am satisfied with the answers to all of my questions and I agree to participate in the study. I understand that I may revoke my consent and leave the study at any stage, if I wish so, with no negative consequences. I agree that the interview / discussion with me should be audio recorded. I understand that the recording is for research purposes only.

Participant's name: ____

Participant's signature or thumbprint: _____________________ Date: _______________

Witness Name (As appropriate): ____

Witness signature (As appropriate): ____________________ Date: __________________

Study team member's statement: I, the undersigned, have explained to the participant in a language that s/he understands; the procedures to be followed in the study (including audio recording of the interview), the risks and benefits involved, and the obligations of the study team.

_____________________________ _____________________________ ________________

Name Signature Date

For peer review only

Informed Consent Agreement to audio record interview with religious leaders

Study title: Scale up of community-based injectable contraceptives in Gombe State, Nigeria

SERIAL NUMBER ____________________

Background and purpose: One of the health innovations that have been piloted with significant success around the world, including Nigeria, is the community-based distribution of injectable contraceptives.

Additional Information: In case you have any question or concern about this study please feel free to contact Dr Oluwaseun Akinyemi, Department of Health Policy and Management, College of Medicine, University of Ibadan Ibadan, Nigeria (Tel. No.: +234 803 502 0136; Email: seunakinyemi@hotmail.com)

Informed consent to record: Please read this statement carefully before you sign and if you agree that the interview / discussion with you should be audio recorded, please fill free to sign this form below to affirm your consent.

I, ________________________________ clearly understand the aims of the project titled **"Scale up of community-based injectable contraceptives in Gombe State, Nigeria"** which has been explained to me by the researcher. I have been given the chance to ask questions and I am satisfied with the answers to all of my questions and I agree to participate in the study. I understand that I may revoke my consent and leave the study at any stage, if I wish so, with no negative consequences. I agree that the interview / discussion with me should be audio recorded. I understand that the recording is for research purposes only.

Participant's name: ____

Participant's signature or thumbprint: _____________________ Date: _______________

Witness Name (As appropriate): ____

Witness signature (As appropriate): ____________________ Date: __________________

Study team member's statement: I, the undersigned, have explained to the participant in a language that s/he understands; the procedures to be followed in the study (including audio recording of the interview), the risks and benefits involved, and the obligations of the study team.

_____________________________ _____________________________ ________________

Name Signature Date

For peer review only

Informed Consent Agreement to audio record focus group discussion with women who currently use injectable contraceptive

Study title: Scale up of community-based injectable contraceptives in Gombe State, Nigeria

SERIAL NUMBER ___________________

Background and purpose: One of the health innovations that have been piloted with significant success around the world, including Nigeria, is the community-based distribution of injectable contraceptives.

Additional Information: In case you have any question or concern about this study please feel free to contact Dr Oluwaseun Akinyemi, Department of Health Policy and Management, College of Medicine, University of Ibadan Ibadan, Nigeria (Tel. No.: +234 803 502 0136; Email: seunakinyemi@hotmail.com)

Informed consent to record: Please read this statement carefully before you sign and if you agree that the interview / discussion with you should be audio recorded, please fill free to sign this form below to affirm your consent.

I, ______________________________ clearly understand the aims of the project titled **"Scale up of community-based injectable contraceptives in Gombe State, Nigeria"** which has been explained to me by the researcher. I have been given the chance to ask questions and I am satisfied with the answers to all of my questions and I agree to participate in the study. I understand that I may revoke my consent and leave the study at any stage, if I wish so, with no negative consequences. I agree that the interview / discussion with me should be audio recorded. I understand that the recording is for research purposes only.

Participant's name: ____

Participant's signature or thumbprint: ____________________ Date: ______________

Witness Name (As appropriate): ____

Witness signature (As appropriate): ___________________ Date: _________________

Study team member's statement: I, the undersigned, have explained to the participant in a language that s/he understands; the procedures to be followed in the study (including audio recording of the discussion), the risks and benefits involved, and the obligations of the study team.

___________________________ ___________________________ _______________

Name Signature Date

For peer review only

Informed Consent Agreement to audio record focus group discussion with women who do not use injectable contraceptive

Study title: Scale up of community-based injectable contraceptives in Gombe State, Nigeria

SERIAL NUMBER ____________________

Background and purpose: One of the health innovations that have been piloted with significant success around the world, including Nigeria, is the community-based distribution of injectable contraceptives.

Additional Information: In case you have any question or concern about this study please feel free to contact Dr Oluwaseun Akinyemi, Department of Health Policy and Management, College of Medicine, University of Ibadan Ibadan, Nigeria (Tel. No.: +234 803 502 0136; Email: seunakinyemi@hotmail.com)

Informed consent to record: Please read this statement carefully before you sign and if you agree that the interview / discussion with you should be audio recorded, please fill free to sign this form below to affirm your consent.

I, ________________________________ clearly understand the aims of the project titled **"Scale up of community-based injectable contraceptives in Gombe State, Nigeria"** which has been explained to me by the researcher. I have been given the chance to ask questions and I am satisfied with the answers to all of my questions and I agree to participate in the study. I understand that I may revoke my consent and leave the study at any stage, if I wish so, with no negative consequences. I agree that the interview / discussion with me should be audio recorded. I understand that the recording is for research purposes only.

Participant's name: ____

Participant's signature or thumbprint: _____________________ Date: _______________

Witness Name (As appropriate): ____

Witness signature (As appropriate): ____________________ Date: __________________

Study team member's statement: I, the undersigned, have explained to the participant in a language that s/he understands; the procedures to be followed in the study (including audio recording of the discussion), the risks and benefits involved, and the obligations of the study team.

_____________________________ _____________________________ ________________

Name Signature Date

For peer review only

Informed Consent Agreement to audio record focus group discussion with married men

Study title: Scale up of community-based injectable contraceptives in Gombe State, Nigeria

SERIAL NUMBER ____________________

Background and purpose: One of the health innovations that have been piloted with significant success around the world, including Nigeria, is the community-based distribution of injectable contraceptives.

Additional Information: In case you have any question or concern about this study please feel free to contact Dr Oluwaseun Akinyemi, Department of Health Policy and Management, College of Medicine, University of Ibadan Ibadan, Nigeria (Tel. No.: +234 803 502 0136; Email: seunakinyemi@hotmail.com)

Informed consent to record: Please read this statement carefully before you sign and if you agree that the interview / discussion with you should be audio recorded, please fill free to sign this form below to affirm your consent.

I, ________________________________ clearly understand the aims of the project titled **"Scale up of community-based injectable contraceptives in Gombe State, Nigeria"** which has been explained to me by the researcher. I have been given the chance to ask questions and I am satisfied with the answers to all of my questions and I agree to participate in the study. I understand that I may revoke my consent and leave the study at any stage, if I wish so, with no negative consequences. I agree that the interview / discussion with me should be audio recorded. I understand that the recording is for research purposes only.

Participant's name: ____

Participant's signature or thumbprint: _____________________ Date: _______________

Witness Name (As appropriate): ____

Witness signature (As appropriate): ____________________ Date: __________________

Study team member's statement: I, the undersigned, have explained to the participant in a language that s/he understands; the procedures to be followed in the study (including audio recording of the discussion), the risks and benefits involved, and the obligations of the study team.

_____________________________ _____________________________ ________________

Name Signature Date

For peer review only

Informed Consent Agreement to audio record focus group discussion with single/unmarried men

Study title: Scale up of community-based injectable contraceptives in Gombe State, Nigeria

SERIAL NUMBER ___________________

Background and purpose: One of the health innovations that have been piloted with significant success around the world, including Nigeria, is the community-based distribution of injectable contraceptives.

Additional Information: In case you have any question or concern about this study please feel free to contact Dr Oluwaseun Akinyemi, Department of Health Policy and Management, College of Medicine, University of Ibadan Ibadan, Nigeria (Tel. No.: +234 803 502 0136; Email: seunakinyemi@hotmail.com)

Informed consent to record: Please read this statement carefully before you sign and if you agree that the interview / discussion with you should be audio recorded, please fill free to sign this form below to affirm your consent.

I, ______________________________ clearly understand the aims of the project titled **"Scale up of community-based injectable contraceptives in Gombe State, Nigeria"** which has been explained to me by the researcher. I have been given the chance to ask questions and I am satisfied with the answers to all of my questions and I agree to participate in the study. I understand that I may revoke my consent and leave the study at any stage, if I wish so, with no negative consequences. I agree that the interview / discussion with me should be audio recorded. I understand that the recording is for research purposes only.

Participant's name: ____

Participant's signature or thumbprint: ____________________ Date: ______________

Witness Name (As appropriate): ____

Witness signature (As appropriate): ___________________ Date: _________________

Study team member's statement: I, the undersigned, have explained to the participant in a language that s/he understands; the procedures to be followed in the study (including audio recording of the discussion), the risks and benefits involved, and the obligations of the study team.

____________________________ ____________________________ _______________

Name Signature Date

For peer review only

Distress Protocol

Distress Protocol 1: The protocol for managing distress in the context of a research focus group /interview

(Adapted from: Draucker C B, Martsolf D S and Poole C (2009) Developing Distress Protocols for research on Sensitive Topics. *Archives of Psychiatric Nursing* 23 (5) pp 343-350)

Distress

• A participant indicates they are experiencing a high level of stress or emotional distress

OR

• Exhibit behaviours suggestive that the discussion/interview is too stressful such as uncontrolled crying, shaking etc.

Stage 1 Response

• Stop the discussion/interview.

• The researcher (who is a health professional) will offer immediate support

• Assess mental status:

  - Tell me what thoughts you are having?
  - Tell me what you are feeling right now?
  - Do you feel you are able to go on about your day?
  - Do you feel safe?

Review

•If participant feels able to carry on; resume interview/discussion

•If participant is unable to carry on, Go to stage 2

Stage 2 Response

•Remove participant from discussion and accompany to quiet area or discontinue interview

•Encourage the participant to contact their GP or mental health provider

OR

•Offer, with participant consent, for a member of the research team to do so OR

•With participant consent contact a member of the health care team treating them for further advice/support

Follow up

•Follow participant up with courtesy call (if participant consents)

OR

•Encourage the participant to call either if he/she experiences increased distress in the hours/days following the focus group

For peer review only

Distress Protocol 2: The protocol for managing distress in the context of a research focus group /interview management

Adapted from: McCosker,H Barnard, A Gerber, R (2001). Undertaking Sensitive Research: Issues and Strategies for Meeting the Safety Needs of All. Forum: Qualitative Social Research, 2(1)

Pre-data collection

•The researcher would consider the potential physical and psychological impact on the researcher of the participants' description of life experiences.

•The researcher would consider how many interviews could be undertaken in a week.

•The researcher would be aware of the potential for emotional exhaustion.

Data collection stage

• For topics that are potentially sensitive/distressing data collection would be undertaken by two members of the research team.

•Regular scheduled debriefing sessions with the lead researcher.

•Research team would be encouraged to journal their thoughts and feelings which may then become part of fieldwork notes in some research approaches.

Analysis

•Research team would be alerted prior to transcription review of potentially "challenging" or "difficult" interviews

•The research team would have regular scheduled debriefing sessions with the lead researcher

Follow up

•Researcher will access a research mentor (supervisor) if he experiences increased distress in the hours/days following transcription.

For peer review only

Distress Protocol 3: The protocol for managing distress in the context of a research focus group /interview transcription

(Adapted from: Gregory, D Russell, C Phillips, L (1997). Beyond textual perfection: transcribers as vulnerable persons. Qualitative Health Research, 7(2), 294-300.)

Pre-data collection

• The transcriber would be considered in the research proposal, with a clear indication of how s/he will be provided with a "safe" working environment while also maintaining the "quality" of the research.

Ethical review stage

• The transcriber would be included in the ethical clearance process.

• The transcriber would be informed of the nature of the research and the type of data.

Pre-transcription

• The transcriber would be alerted prior to the transcription of potentially "challenging" or "difficult" interviews.

•The transcriber would have regular scheduled debriefing sessions with a named member of the research team.

During Transcription

•Transcriber would have prompt access to an appropriate person for crisis counselling.

•S/he would have a clearly documented termination from the transcription process that includes resolution of personal issues which arose as a consequence of the work.

•Transcriber would be encouraged to journal her/his thoughts and feelings which may then become part of fieldwork notes.

Follow up

•Follow transcriber up with courtesy call (if transcriber consents)

OR

•Encourage the transcriber to call if s/he experiences increased distress in the hours/days following transcription

For peer review only

Reporting checklist for qualitative study.

Based on the SRQR guidelines.

Instructions to authors

Complete this checklist by entering the page numbers from your manuscript where readers will find each of the items listed below.

Your article may not currently address all the items on the checklist. Please modify your text to include the missing information. If you are certain that an item does not apply, please write "n/a" and provide a short explanation.

Upload your completed checklist as an extra file when you submit to a journal.

In your methods section, say that you used the SRQRreporting guidelines, and cite them as:

O'Brien BC, Harris IB, Beckman TJ, Reed DA, Cook DA. Standards for reporting qualitative research: a synthesis of recommendations. Acad Med. 2014;89(9):1245-1251.

		Reporting Item	Page Number
**Title**			
	#1	Concise description of the nature and topic of the study identifying the study as qualitative or indicating the approach (e.g. ethnography, grounded theory) or data collection methods (e.g. interview, focus group) is recommended	1
**Abstract**			
	#2	Summary of the key elements of the study using the abstract format of the intended publication; typically includes background, purpose, methods, results and conclusions	2
**Introduction**			
Problem formulation	#3	Description and signifcance of the problem / phenomenon studied: review of relevant theory and empirical work; problem statement	4-5

For peer review only

Purpose or research question	#4	Purpose of the study and specific objectives or questions	5
**Methods**			
Qualitative approach and research paradigm	#5	Qualitative approach (e.g. ethnography, grounded theory, case study, phenomenolgy, narrative research) and guiding theory if appropriate; identifying the research paradigm (e.g. postpositivist, constructivist / interpretivist) is also recommended; rationale. The rationale should briefly discuss the justification for choosing that theory, approach, method or technique rather than other options available; the assumptions and limitations implicit in those choices and how those choices influence study conclusions and transferability. As appropriate the rationale for several items might be discussed together.	6
Researcher characteristics and reflexivity	#6	Researchers' characteristics that may influence the research, including personal attributes, qualifications / experience, relationship with participants, assumptions and / or presuppositions; potential or actual interaction between researchers' characteristics and the research questions, approach, methods, results and / or transferability	6
Context	#7	Setting / site and salient contextual factors; rationale	5
Sampling strategy	#8	How and why research participants, documents, or events were selected; criteria for deciding when no further sampling was necessary (e.g. sampling saturation); rationale	5-6
Ethical issues pertaining to human subjects	#9	Documentation of approval by an appropriate ethics review board and participant consent, or explanation for lack thereof; other confidentiality and data security issues	7
Data collection methods	#10	Types of data collected; details of data collection procedures including (as appropriate) start and stop dates of data collection and analysis, iterative	5-6

For peer review only

		process, triangulation of sources / methods, and modification of procedures in response to evolving study findings; rationale	
Data collection instruments and technologies	#11	Description of instruments (e.g. interview guides, questionnaires) and devices (e.g. audio recorders) used for data collection; if / how the instruments(s) changed over the course of the study	6
Units of study	#12	Number and relevant characteristics of participants, documents, or events included in the study; level of participation (could be reported in results)	5-6
Data processing	#13	Methods for processing data prior to and during analysis, including transcription, data entry, data management and security, verification of data integrity, data coding, and anonymisation / deidentification of excerpts	6
Data analysis	#14	Process by which inferences, themes, etc. were identified and developed, including the researchers involved in data analysis; usually references a specific paradigm or approach; rationale	6-7
Techniques to enhance trustworthiness	#15	Techniques to enhance trustworthiness and credibility of data analysis (e.g. member checking, audit trail, triangulation); rationale	7
**Results/findings**			
Syntheses and interpretation	#16	Main findings (e.g. interpretations, inferences, and themes); might include development of a theory or model, or integration with prior research or theory	7-12
Links to empirical data	#17	Evidence (e.g. quotes, field notes, text excerpts, photographs) to substantiate analytic findings	7-12
**Discussion**			
Intergration with prior work, implications, transferability and contribution(s) to the field	#18	Short summary of main findings; explanation of how findings and conclusions connect to, support, elaborate on, or challenge conclusions of earlier scholarship; discussion of scope of application /	12-14

For peer review only

		generalizability; identification of unique contributions(s) to scholarship in a discipline or field	
Limitations	#19	Trustworthiness and limitations of findings	14
**Other**			
Conflicts of interest	#20	Potential sources of influence of perceived influence on study conduct and conclusions; how these were managed	3
Funding	#21	Sources of funding and other support; role of funders in data collection, interpretation and reporting	3

None The SRQR checklist is distributed with permission of Wolters Kluwer © 2014 by the Association of American Medical Colleges. This checklist can be completed online using https://www.goodreports.org/, a tool made by the EQUATOR Network in collaboration with Penelope.ai

For peer review only
